# On the Equivalence of Two-Point Basis-Set Extrapolations and Robust Parameterization for Coupled-Cluster and Double-Hybrid DFT Methods

**DOI:** 10.1021/acs.jpca.6c01541

**Published:** 2026-05-27

**Authors:** Mark A. Iron

**Affiliations:** Computational Chemistry Unit, Department of Chemical Research Support, 34976 Weizmann Institute of Science, Rehovot 7610001, Israel

## Abstract

Basis-set extrapolation (BSE) to the complete basis set (CBS) limit is a cornerstone of several high-accuracy thermochemical computational schemes. In this study, we assess some of the approximations that are commonly made when using BSE. While the extrapolation parameters are typically fitted using coupled-cluster methods, there are cases where these same parameters are applied to other computational methods. While there can be significant differences in the fitted parameters, when applied to two benchmark datasets the difference in performance between the coupled-cluster and method-specific fitted parameters is small. While there are a number of schemes for two-basis-set extrapolations to the CBS limit, it is shown that three common extrapolations—exponential, exponential–square root, and inverse power—reduce to a Schwenke-style correction to the larger basis set of the scaled difference between two sequential basis sets (*E*_CBS_ = *E*_*n*_ + *f*·(*E*_*n*_ – *E*_*n*–1_)). Moreover, this reduction means that the differences between the methods is mathematically meaningless, and given the extrapolation parameter of one scheme one can find both the Schwenke-type extrapolation factor *f* and the extrapolation factors of the other two schemes and vice versa. Finally, Schwenke-type two-point extrapolation parameters are given for a selection of basis sets for coupled-cluster and selected double-hybrid density functional theory exchange–correlation functionals.

## Graphical abstract

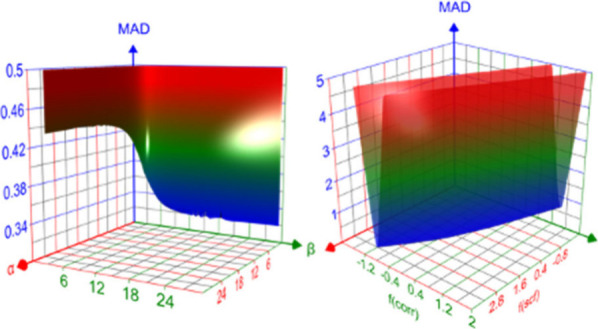

## 1 Introduction

The basis-set approximation (i.e., the use of a set of mathematical functions to describe the wavefunction) is a cornerstone of computational chemistry, in particular for wavefunction theory (WFT) and density functional theory (DFT, also known as Kohn–Sham theory) methods. Ideally, one would like to work in an infinite basis set as then calculations would be exact (within a given computational method). Unfortunately, computers (and their users) tend to not like working with infinity, so finite basis sets are used. This, however, introduces inherent error into the calculations, which can be minimized, at least, by using bigger and bigger basis sets. But even this has its limits as calculations tend to scale rather unfavorably with the basis set size (e.g., Hartree–Fock scales as *N* ^4^ while CCSD­(T) scales as *N* ^7^, where *N* is the number of basis set functions). There are further approximations, such as the resolution of the identity (RI) or localized methods (e.g., the DLPNO approximation in Orca), that can also be used to reduce the computational cost, but these are beyond the scope of this study.

This is where basis-set extrapolation (BSE) comes to the rescue. It was observed that properties (such as the total electronic energy) tend to converge smoothly and monotonically with increasing basis-set size.^1^ One can take advantage of this behavior and use a small set of calculations with increasing basis set size to extrapolate to the infinite basis set, often termed the complete basis set (CBS) limit. This forms the basis for a number of high-accuracy thermochemical schemes (HATS), such as the Weizmann-*n*, HEAT, Gaussian-*n*, and CBS methods (for recent reviews, see refs 2–5 ), which are generally based on either coupled-cluster or Møller–Plesset methods.

A number of extrapolations that have been proposed for BSE, and the underlying equations are discussed hereinafter in section 3.2. A common choice is to use an exponential–square root extrapolation (*E*_*n*_ ≈ *E*_CBS_ + *A*·exp­(−α√*L*))^6^ for the Hartree–Fock energy and an inverse power extrapolation (*E*_*n*_ ≈ *E*_CBS_ + *B*·*n* ^–β^)^7^ for the correlation energy. Neese and Valeev optimized α and β values for two-basis-set extrapolations (2BSE) for various basis set combinations,^8^ and it is these parameters that are commonly used for most applications. While most procedures for determining the 2BSE parameters involve fitting to benchmark energies, such as in Neese and Valeev’s study, Fishman et al. took a novel approach and required the basis set superposition error (BSSE) to vanish at the CBS limit.^9^ Nevertheless, the steep scaling of WFT methods with basis set size will always impose limits on the size of systems that can be studied computationally.

Because of its computational efficiency, DFT is a stalwart of calculations of large systems. In particular, double-hybrid (DH) functionals have been shown to have accuracies approaching that of the significantly more expensive CCSD­(T) at a fraction of the cost,^10^ especially if the various linearization techniques (e.g., RI-MP2, RIJK, RIJCOSX) are used. Chai and Head-Gordon used an inverse power extrapolation (β = 3) for CBS extrapolation of the PT2 part of the ωB97X(2) DH functional.^11^ Chuang and Chen later considered various extrapolation schemes for the various parameterizations of the B2PLYP DH functional;^12,13^ in their study the considered all three extrapolations schemes considered herein and optimized the parameters specifically for each functional. Karton and Martin evaluated basis-set extrapolations with B2GP-PLYP, both conventional and explicitly correlation (i.e., B2GP-PLYP-F12), but took their β extrapolation parameter from CCSD-F12 extrapolations (specifically Hill et al.^14^);^15^ Mehta and Martin followed this up with a more extensive comparison with the GMTKN55 benchmark dataset.^16^ Kraus evaluated the performance of a selection of hybrid and DH functionals and determined appropriate extrapolation parameters (in addition to reevaluating Hartree–Fock CBS extrapolations).^17^ In another example, Dohm et al.^18^ used BSE-extrapolated PWPB95 energies when they amended some of the entries in this author’s MOBH35 benchmark set, which are based on W1-like CBS extrapolations using CCSD­(T) methods.^19^

Despite the widespread use of BSE, there are still some open questions that will be addressed in this study:

- Neese and Valeev stated that they had fit their α and β 2BSE parameters by minimizing the mean absolute deviation (MAD) of the total energy for a set of 21 small molecules.^8^ As discussed in section 3.3, there is reason to believe that this may not be ideal because extrapolation of the Hartree–Fock (to avoid confusion with the molecule hydrogen fluoride, which is a member of our test set (*vide supra*), this quantity will be denoted as SCF, an acronym of self-consistent field) energy should be independent of the correlation treatment and because the SCF energy is 2–3 orders of magnitude larger than the correlation energy.
- Schwenke proposed a 2BSE based on a scaled difference in energy calculated with two consecutive basis sets using the exponential−square root extrapolation for the SCF energy and an inverse power extrapolation for the CCSD­(T) correlation energy.^20^ Is there any advantage to this? As shown in section 3.2, the various 2BSE methods can be reduced to a Schwenke-type extrapolation, making the differences between the different methods less meaningful.
- A number of HATS use 2BSE parameters fit for CCSD­(T) correlation with other correlation methods. Is this justified?
- The CCSD­(T)-fit 2BSE parameters have also been used for DH functionals. Is this a reasonable approach?

## 2 Computational Methods

Most calculations were done using Molpro versions 2022.2 and 2023.2; double-hybrid DFT calculations were done using Molpro 2025.4 ^21–23^ (section 3.6, parameter fitting). The S66 RI-QCISD­(T), RI-MP2 (section 3.5) and double-hybrid DFT (section 3.6) calculations were run using Orca 6.0.1,^24^ while the MB43-16 MP2 (section 3.5) and DSD-PBEP86-D3BJ (section 3.6) calculations were done using Gaussian 16, rev. C.01.^25^

A number of WFT *ab initio* methods were used:

- Hartree–Fock (denoted as SCF);^26,27^

- Coupled-cluster with single and double excitations and a quasiperturbative treatment of the triples contribution (CCSD­(T));^28–36^

- Quadratic configuration interaction with single and double excitations and a triples correction (QCISD­(T));^34,37,38^

- Second-order Møller–Plesset theory (MP2).^39^

All WFT methods used a restricted open-shell Hartree–Fock (ROHF) reference.

A selection of DH DFT exchange–correlation functionals were used:

- Grimme’s original DH functional using the Becke88 (B88) exchange functional^40^ and the Lee–Yang–Parr (LYP) correlation functional^41^ (B2PLYP);^42^
- Goerigk and Grimme’s DH functional based on the Perdew–Wang91 (PW91) exchange functional^43–47^ and the Becke95 (B95) correlation functional^48^ (PWPB95);^49^
- Schwabe and Grimme’s DH functional based on the modified Perdew–Wang (mPW91) exchange functional^50^ (where the mPW91 functional used the build-it LibXC library^51,52^ ) and the LYP correlation functional (mPW2PLYP);^53^
- Kozuch and Martin’s original implementation of a dispersion-corrected (specifically the third version of Grimme and co-workers’ empirical dispersion correction^54^ with Becke–Johnson dampening, that is D3BJ^55,56^ ) DH functional with spin-component-scaled (SCS) MP2-like correlation^101^ with the Perdew–Burke–Ernzerhof (PBE) exchange functional^57^ and the Perdew-86 correlation functional^58^ (DSD-PBEP86);^59^
- Santra et al.’s revised version of the previous (revDSD-PBEP86-D3BJ) and the version with Grimme and co-workers fourth version of their empirical dispersion correction^60,61^ (revDSD-PBEP86-D4);^62^
- Karton et al.’s general purpose reparameterization of B2PLYP (B2GP-PLYP);^63^

DFT calculations used the unrestricted Kohn–Sham (UKS) formalism for open-shell systems and the restricted Kohn–Sham (RKS) formalism for the closed-shell systems. Molrpo’s “high” grid accuracy setting was used. Molpro input files for the DSD double-hybrid functionals were taken from the homepage of Prof. Jan M. L. (Gershom) Martin.^64^

Four families of basis sets were used:

- Dunning’s correlation-consistent basis sets (cc-pV*n*Z and aug-cc-pV*n*Z, *n* = D, T, Q, 5, 6),^65–68^ which include by default the (aug-)­cc-pV­(*n*+d)­Z basis set that includes an extra *d* polarization function on the second-row main group elements (i.e., Al–Ar)^69^ (the aug-cc-pV5Z basis set for Mg was taken from the core–valence basis sets of Iron, Oren, and Martin but with the core–valence functions removed^70^);
- Nesse and Valeev’s atomic natural orbital (ANO) basis sets (ANO-pV*n*Z and aug-ANO-pV*n*Z, *n* = D, T, Q, 5);^8^
- The Weigend and Ahlrichs family of def2 basis sets without (i.e., def2-SVP, def2-TZVPP, and def2-QZVPP) and with (i.e., def2-SVPD, def2-TZVPPD, and def2-QZVPPD) diffuse functions (the corresponding def2-TZVP and def2-QZVP basis sets were not used—for discussion, see Supporting Information (SI) section S1);^71^
- Jensen’s polarization consistent basis sets (pc-*n* and aug-pc-*n*, *n* = 1–4).^72–74^

The ANO-pV*n*Z and aug-ANO-pV*n*Z basis sets were downloaded from the Basis Set Exchange (BSE) site.^75–77^ Note that the version of ANO-pVTZ in the BSE (at the time of writing) and in the Supporting Information of Neese and Valeev’s paper^8^ is actually a copy of ANO-pVDZ (this is a documented bug in the BSE^78^); as recommended by Neese in the bug report, the ANO-pVTZ basis set was obtained from the ANO-pVQZ basis set by removing the outermost function for each angular momentum (the final basis set is provided in selected formats in SI section S7 and attached as separate SI files in Orca, Gaussian, and Molpro formats).^79^ In addition, the ANO-pV5Z and aug-ANO-pV5Z basis sets for hydrogen are identical to their Qζ counterparts in all available sources but were used as is.

Geometries of the BSEF21 ^8^ (section 3.1), S66 ^80,81^ and MB43-16 ^82,83^ (sections 3.5 and 3.6) were taken from the respective publications; some of the BSEF21 geometries and the new geometries in the BSEF74 set (see section 3.1 and SI section S2) were reoptimized at the same level of theory as in the original study (i.e., Becke’s three-parameter hybrid functional^84,85^ using the B88 exchange functional^40^ and the LYP correlation functional^41^ with VWN3 ^86^ as implemented in Gaussian16 with the def2-TZVP basis set—i.e., B3LYP/def2-TZVP—using Gaussian16). One member of MB43-16 has a negative energy of formation; to make statistical analysis simpler, the direction of this reaction was flipped so that all energies are positive.

Parameter fitting was done using the Solver module of Microsoft Excel using the default “GRG Nonlinear” engine. Maple 2024 was used to assist with some of the algebraic derivations.

## 3 Results and Discussion

### 3.1 Basis-Set Extrapolation Fitting Sets

Neese and Valeev used a set of 21 small molecules (which will be denoted here as BSEF21) to fit the parameters for canonical CCSD­(T).^8^ The same procedure will be used in this study. The molecules in the dataset and their electronic states (and the equivalents in the largest nondegenerate Abelian point-group, where applicable) are H_2_^1^Σ_g_^+^ (^1^A_1_), BH_3_^1^A_1_^′^ (^1^A_1_), CH_4_^1^A_1_ (^1^A′), NH_3_^1^A_1_ (^1^A_1_), H_2_O ^1^A_1_, HF ^1^Σ^+^ (^1^A_1_), B_2_^3^Σ_g_^–^ (^3^B_1g_), BC ^4^Σ^–^ (^4^A_2_), BN ^3^Π (^3^A_2_), BO ^2^Σ^+^ (^2^A_1_), BF ^1^Σ^+^ (^1^A_1_), C_2_^1^Σ_g_^+^ (^1^A_g_), CN ^2^Σ^+^ (^2^A_1_), CO ^1^Σ^+^ (^1^A_1_), CF ^2^Π (^2^B_2_ or ^2^B_1_), N_2_^1^Σ_g_^+^ (^1^A_g_), NO ^2^Π (^2^B_2_ or ^2^B_1_), NF ^3^Σ^–^ (^3^A_2_), O_2_^3^Σ_g_^–^ (^3^B_1g_), OF ^2^Π (^2^B_2_ or ^2^B_1_), and F_2_^1^Σ_g_^+^ (^1^A_g_). The geometries were taken from the paper by Neese and Valeev^8^ except as noted in SI section S2, and the structures used are given in Table S3. This is the set used in the initial evaluations of the current study to be consistent with Neese and Valeev’s study.

The second set adds 53 second-row main group elements and the monohydrides: BH ^1^Σ^+^ (^1^A_1_), CH ^2^Π (^2^B_2_ or ^2^B_1_), NH ^3^Σ^–^ (^3^A_2_), OH ^2^Π (^2^B_2_ or ^2^B_1_), Al_2_^3^Σ_g_^–^ (^3^B_1g_), AlH ^1^Σ^+^ (^2^A_1_), AlH_3_^1^A_1_^′^ (^1^A_1_), AlB ^3^Σ^–^ (^3^A_2_), AlC ^4^Σ^–^ (^4^A_2_), AlN ^3^Π (^3^B_2_ or ^3^B_1_), AlO ^2^Σ^+^ (^2^A_1_), AlF ^1^Σ^+^ (^1^A_1_), AlSi ^4^Σ^–^ (^4^A_2_), AlP ^3^Π (^3^B_2_ or ^3^B_1_), AlS ^2^Σ^+^ (^2^A_1_), AlCl ^1^Σ^+^ (^1^A_1_), Si_2_^3^Σ_g_^–^ (^3^B_1g_), SiH ^2^Π (^2^B_2_ or ^2^B_1_), SiH_4_^1^A_1_ (^1^A′), SiB ^4^Σ^–^ (^4^A_2_), SiC ^3^Π (^3^B_2_ or ^3^B_1_), SiN ^2^Σ^+^ (^2^A_1_), SiO ^1^Σ^+^ (^1^A_1_), SiF ^2^Π (^2^B_2_ or ^2^B_1_), SiP ^2^Σ^+^ (^2^A_1_), SiS ^1^Σ^+^ (^1^A_1_), SiCl ^2^Π (^2^B_2_ or ^2^B_1_), P_2_^1^Σ_g_^+^ (^1^A_g_), PH ^3^Σ^–^ (^3^A_2_), PH_3_^1^A_1_ (^1^A_1_), PB ^3^Π (^3^B_2_ or ^3^B_1_), PC ^2^Σ^+^ (^2^A_1_), PN ^1^Σ^+^ (^1^A_1_), PO ^2^Π (^2^B_2_ or ^2^B_1_), PF ^3^Σ^–^ (^3^A_2_), PS ^2^Π (^2^B_2_ or ^2^B_1_), PCl ^3^Σ^–^ (^3^A_2_), S_2_^3^Σ_g_^–^ (^3^B_1g_), SH ^2^Π (^2^B_2_ or ^2^B_1_), H_2_S ^1^A_1_, SB ^2^Σ^+^ (^2^A_1_), SC ^1^Σ^+^ (^1^A_1_), SN ^2^Π (^2^B_2_ or ^2^B_1_), SO ^3^Σ^–^ (^3^A_2_), SF ^2^Π (^2^B_2_ or ^2^B_1_), SCl ^2^Π (^2^B_2_ or ^2^B_1_), Cl_2_^1^Σ_g_^+^ (^1^A_g_), HCl ^1^Σ_g_^+^ (^1^A_g_), BCl ^1^Σ^+^ (^1^A_1_), CCl ^2^Π (^2^B_2_ or ^2^B_1_), ClN ^3^Σ^–^ (^3^A_2_), ClO ^2^Π (^2^B_2_ or ^2^B_1_), and ClF ^1^Σ^+^ (^1^A_1_). The geometries, following Neese and Valeev, were optimized at the B3LYP/def2-TZVP level of theory.^8^ This set is denoted as BSEF74 and is used for final parameter fitting. When fitting the double-hybrid DFT functionals (section 3.6), SiP was problematic—its energy would often not converge—so it was removed from the fitting and evaluations for these functionals, giving the BSEF74* dataset.

In principle, one could also add third-row main group elements or transition metals to the evaluation. However, this limits the availability of basis sets (e.g., the aug-cc-pV6Z basis set—at the time of writing—used in the CBS benchmark extrapolations does not include these elements), in addition to the potential need to account for relativistic effects (either explicitly with a relativistic Hamiltonian, such as Douglass–Kroll–Hess (DHK) or X2C, or using a relativistic effective core potential (RECP)), and thus this is beyond the scope of the current study.

### 3.2 Basis-Set Extrapolation

A number of approaches for extrapolating to the basis-set limit have been proposed, but here we will focus on what is commonly used, where one scheme is used to extrapolate the SCF energies and a second for the correlation energies.

Generally, the SCF energy is extrapolated using an exponential–square root function originally proposed by Zhong et al.:^6^

1 $$E_{n}\approx E_{\mathrm{CBS}}+A\cdot \exp\left(-\alpha \sqrt{L}\right)$$

where *E*_*n*_ and *E*_CBS_ are the energies with the *n-*tuple-ζ basis set and at the CBS limit, *L* is the highest angular momentum of the basis set (which in general equals *n* with Dunning’s cc-pV*n*Z family of basis sets), and *A* and α are parameters. For two basis sets, this can be written in a more convenient form (where *n*_2_ > *n*_1_ hereinafter):

2 $$E_{\mathrm{CBS}}^{\mathrm{SCF}}\left({n_{1},n}_{2}\right)\approx E_{n_{2}}+\frac{E_{n_{2}}-E_{n_{1}}}{\exp\left[\alpha \left(\sqrt{n_{2}}-\sqrt{n_{1}}\right)\right]-1}=E_{n_{2}}+f_{\alpha }\left(n_{1},n_{2},\alpha \right)\cdot \left(E_{n_{2}}-E_{n_{1}}\right)$$

Algebraic manipulation (see SI section S3 for details of all algebra in this section) can give *f*_α_(*n*_1_, *n*_2_, α) in a convenient format:

3 $$f_{\alpha }\left(n_{1},n_{2},\alpha \right)=\frac{1}{\exp\left[\alpha \left(\sqrt{n_{2}}-\sqrt{n_{1}}\right)\right]-1}=\frac{\exp\left(\alpha \sqrt{n_{1}}\right)}{\exp\left(\alpha \sqrt{n_{2}}\right)-\exp\left(\alpha \sqrt{n_{1}}\right)}$$

Correlation energies (*E*_corr_) are typically extrapolated based on Halkier et al.’s inverse-cubic extrapolation (which in its original implementation has β = 3,^7^ but Truhlar and co-workers later showed that smaller values—β_MP2_ = 1.91, β_CCSD_ = 1.94, and β_CCSD(T)_ = 2.02—are better^87,88^ ):

4 $$E_{n}\approx E_{\mathrm{CBS}}+B\cdot n^{-\beta }$$

For two basis sets, this is commonly written as

5 $$E_{\mathrm{CBS}}^{\mathrm{corr}}\left({n_{1},n}_{2}\right)\approx \frac{n_{1}^{\beta }E_{n_{1}}^{\mathrm{corr}}-n_{2}^{\beta }E_{n_{2}}^{\mathrm{corr}}}{n_{1}^{\beta }-n_{2}^{\beta }}$$

This can also be written as a correction based on the difference of energies calculated using two consecutive basis sets:

6 $$E_{\mathrm{CBS}}^{\mathrm{corr}}\left(n_{1},n_{2}\right)\approx E_{n_{2}}+f_{\beta }\left(n_{1},n_{2},\beta \right)\cdot \left(E_{n_{2}}-E_{n_{1}}\right)$$

where

7 $$f_{\beta }\left(n_{1},n_{2},\beta \right)=\frac{n_{1}^{\beta }}{n_{2}^{\beta }-n_{1}^{\beta }}=\frac{n_{2}^{-\beta }}{n_{1}^{-\beta }-n_{2}^{-\beta }}$$

Alternatively, an exponential (geometric) extrapolation has been used:^89^

8 $$E_{n}\approx E_{\mathrm{CBS}}+C\cdot \exp\left(-\gamma \cdot L\right)$$

This can analogously be written as

9 $$E_{\mathrm{CBS}}=E_{n_{2}}+f_{\gamma }\left(\gamma ,n_{1},n_{2}\right)\cdot \left(E_{n_{2}}-E_{n_{1}}\right)$$

where

10 $$f_{\gamma }\left(\gamma ,n_{1},n_{2}\right)=\frac{\exp\left(\gamma \cdot n_{1}\right)}{\exp\left(\gamma \cdot n_{2}\right)-\exp\left(\gamma \cdot n_{1}\right)}$$

Schwenke proposed extrapolating to the CBS limit using the scaled difference of the energies calculated with two consecutive basis sets as a correction factor:^20^

11 $$E_{\mathrm{CBS}}\left(n_{1},n_{2}\right)\approx E_{n_{2}}+f\cdot \left(E_{n_{2}}-E_{n_{1}}\right)$$

­(The original equation proposed by Schwenke was a correction to the smaller basis set; see SI section S3.4 for discussion.) It was noted in 2013 by Ranasinghe and Petersson^90^ (they used λ instead of *f*) that expressing the extrapolations in such a form leads to easier parameter optimization, yet there is still use of the various two-point extrapolations. Spackman and Karton demonstrated that the inverse power extrapolation (eq 4) can be reduced to a Schwenke-like extrapolation,^91^ and Martin further demonstrated that the three extrapolation equations (eqs 2, 5, and 9) can be rearranged to give Schwenke-like extrapolations.^92^

Once the extrapolation method is written in the form of a Schwenke-like equation (i.e., eq 2, 6, or 9), then the differences between the various method become mathematically meaningless. There is a direct monotonic relation between α, β, and γ with the correction function *f* (eqs 3, 7, and 10), as shown in Figure 1. Thus, one can express each parameter in terms of the correction function *f*:

12 $$\alpha =\frac{\ln\left(\frac{f+1}{f}\right)}{\sqrt{n_{2}}-\sqrt{n_{1}}}$$

13 $$\beta =\frac{\ln\left(\frac{f+1}{f}\right)}{\ln\left(n_{2}\right)-\ln\left(n_{1}\right)}$$

14 $$\gamma =\ln\left(\frac{f+1}{f}\right)$$

This means that one can move between extrapolation methods and that if one has one of the parameters, one can get the corresponding other two. The formulas for the interconversion of the three parameters are:

15 $$\alpha =\frac{\ln\left[{\left(\frac{n_{2}}{n_{1}}\right)}^{\beta }\right]}{\sqrt{n_{2}}-\sqrt{n_{1}}}$$

16 $$\beta =\frac{\alpha \left(\sqrt{n_{2}}-\sqrt{n_{1}}\right)}{\ln\left(n_{2}\right)-\ln\left(n_{1}\right)}$$

17 $$\alpha =\frac{\gamma }{\sqrt{n_{2}}-\sqrt{n_{1}}}$$

18 $$\beta =\frac{\gamma }{\ln\left(n_{2}\right)-\ln\left(n_{1}\right)}$$

19 $$\gamma =\alpha \cdot \left(\sqrt{n_{2}}-\sqrt{n_{1}}\right)$$

20 $$\gamma =\beta \cdot \left(\ln\left(n_{2}\right)-\ln\left(n_{1}\right)\right)$$

Therefore, given that α, β, and γ can be expressed in terms of each other, there is only meaning in optimizing the correction factor *f*, and for any component of the total energy one obtains

21 $$E_{\mathrm{CBS}}\approx E_{n_{2}}+f\cdot \left(E_{n_{2}}-E_{n_{1}}\right)$$

**Figure 1:**
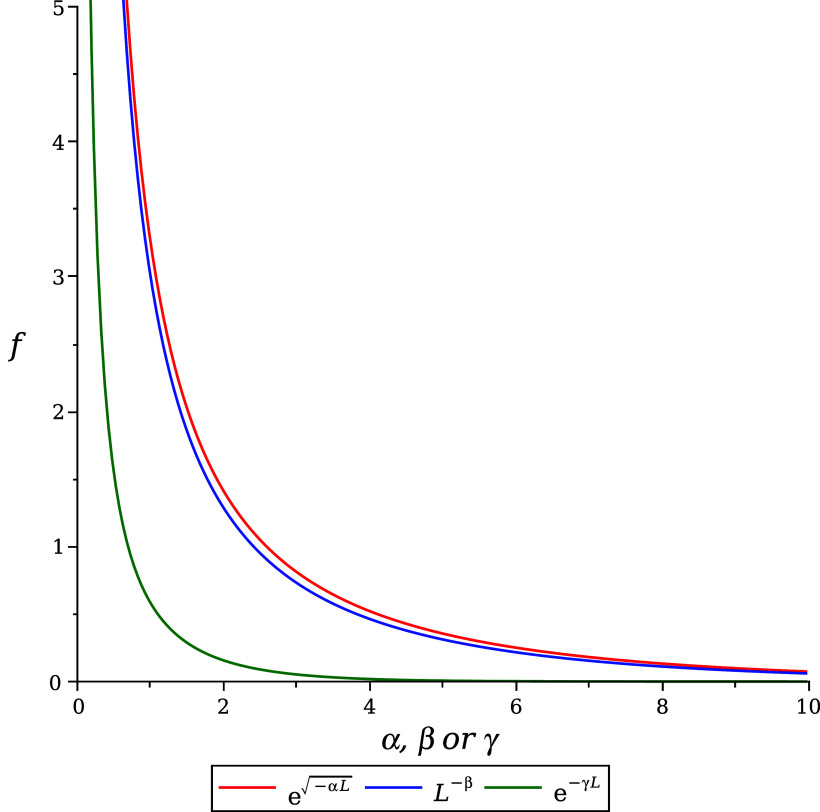
Plot of the correction functions *f* for a {T,Q}­ζ extrapolation as a function of α, β, and γ.

The Schwenke extrapolation parameter is related to the rate of convergence with respect to the basis set. One way to view this is to consider the relation between *f* and β. As shown in eq S7c, $f=\frac{1}{{\left(\frac{n_{2}}{n_{1}}\right)}^{\beta }-1}$ , so for a fixed *n*_2_, a larger β means a faster decay. Given the inverse relation between *f* and β (Figure 1), a smaller *f* correlates to a faster decay. Another way to look at it is to consider the form of the Schwenke extrapolation (eq 21): *f* is directly linked to how much basis-set error remains with basis set *n*_2_ relative to the last basis set increment (i.e., the residual error Δ_*n*_2__ = *E*_CBS_ – *E*_*n*_2__). *f* is related to the number of such increments that are “missing” beyond *n*_2_, so a small *f* means that the last increment already is sufficient to get one close to the CBS limit or fast convergence.

For three consecutive basis sets (e.g., AVTZ, AVQZ, AV5Z) with the exponential extrapolation scheme (eq 8), one can express the CBS limit solely in terms of the energies calculated with each basis set without resorting to optimizable parameters:^93^

$$E_{\mathrm{CBS}}=\frac{E_{n}E_{n+2}-E_{n+1}^{2}}{E_{n}-2E_{n+1}+E_{n+2}}$$ 22

where *n* is the cardinal number of the smallest basis set in the series. (This is the standard Aitken Δ^2^ process, also known as the three-point Aitken extrapolation;^94,95^ see SI section S4 for my algebraic derivation of this equation.) Since there are no parameters to optimize, this has the advantage that a definite and unambiguous answer is obtained. Unfortunately, for the other two extrapolations discussed here, because of the presence of the square root in eq 1 and the power of β in eq 4, analytical extrapolation equations of this type (eq 22) are impossible, even for Maple. One would thus have to fit the three parameters in the relevant extrapolations using energetic data from three or more consecutive basis sets in order to find *E*_CBS_; as will be shown in the next section, this can lead to ambiguity in the results. (For additional discussion, see SI section S5.)

There is a theoretical basis for the commonly used BSE schemes, governed by two distinct asymptotic behaviors. The first was described in initial work by Klopper and Kutzelnigg^96^ and further developed by McKemmish and Gill.^97^ They demonstrated that the basis-set incompleteness error for Gaussian orbital basis sets generally decays as the exponential–square root of the cardinal number (i.e., eq 1). It was shown that this behavior stems from the inability of Gaussian functions to describe the electron–nucleus cusp at *r* = 0 together with the analytic structure of the wavefunction that leads to a convergence that differs from a simple power law. The second asymptotic behavior is related to the convergence of the correlation energy.^96^ An inverse power law convergence was predicted from partial-wave analyses of the electron–electron interaction (i.e., eq 4), where β = 3 for the singlet-coupled (i.e., opposite spin, αβ) pairs and β = 5 for the triplet-coupled (i.e., same spin, αα and ββ) pairs. If one truncates this expansion and assumes that the total correlation energy is dominated by the singlet-coupled contributions, one obtains the oft used *L* ^–3^ extrapolation; this behavior was examined in correlated calculations by Helgaker et al.^1^ and Halkier et al.^7^ However, in practice, the triplet-coupled contributions can be significant, and the more rigorous approach would be to extrapolate each contribution independently; this is beyond the scope of this study.

These theoretical limits, however, are only approximately realized in practice. The derivations assume that the basis set is complete in angular momentum. Commonly used Gaussian basis sets (e.g., Dunning’s correlation-consistent basis set, Jensen’s polarization-consistent basis set, and Weigend and Ahlrichs’s def2 basis set—see Computational Methods for details) are not strictly complete in this sense, and they often suffer from radial incompleteness. This is in addition to the above-mentioned significant contributions from the triplet-coupled contributions. Consequently, practical calculations are often far from the true asymptotic regime, which has led the widespread use of semiempirical extrapolation schemes where the functional form is modivated by theoretical considerations but the parameters are optimized against benchmark data for specific methods and/or basis sets.^1,7^

### 3.3 Evaluating the Fitting Procedures: CCSD­(T)

CCSD­(T) is the unquestioned workhorse of accurate thermochemistry calculations. Neese and Valeev stated that they optimized α and β for canonical CCSD­(T) calculations for various basis set pairs by minimizing the MAD of the total CBS energy (i.e., *E*_tot_ = *E*_SCF_ + *E*_corr_) of the BSEF21 dataset (or more accurately a version thereof; see section 3.1).^8^ These values are commonly used in various extrapolation methods.

The are three major questions that need to be addressed here: (*i*) Is it justified to simultaneously fit the α and β parameters for the SCF and correlation energies? (*ii*) Is it justifiable to consider the CCSD and (T) correlation energies as a single entity? (*iii*) What is the impact of the extrapolation method used (i.e., exponential–square root versus inverse power versus exponential)?

When the SCF and correlation energy parameters are optimized simultaneously by minimizing the MAD of *E*_tot_, the SCF extrapolation becomes dependent on the choice of correlation method (e.g., CCSD­(T), CCSDT, QCISD­(T), MP2, or even none). This is illogical and could lead to extrapolations that are not in line with the physics of the system. Furthermore, there are 2–3 orders of magnitude between the SCF reference energy and the CCSD correlation energy and between the CCSD and (T) correlation energies (see Table 1). The SCF component dominates the total energy, and the correlation contribution can vary between different molecules. A simultaneous parameter optimization will ignore the real extrapolation behavior of each component and focus on what gives the most accurate total energy. This is undesirable.

**Table 1:** Complete Basis Set (CBS) Limit Energies (SCF, Total Correlation, CCSD Correlation, and (T) Correlation, All in Ha) and the Difference in Calculated Correlation Energy (Defined as Δ*E*_CBS_^corr^ *= E*_CBS_^corr^ – *E*_CBS_^CCSD^ – *E*_CBS_^(T)^, in mHa and cal/mol) for Each Member of the BSEF21 Dataset Calculated Using the Three-Point Exponential Extrapolation (eq 22) and the aug-cc-pV*n*Z (*n* = Q, 5, 6) Basis Sets

						Δ*E*_CBS_^corr^	
molecule	spin state	*E*_CBS_^SCF^	*E*_CBS_^corr^	*E*_CBS_^CCSD^	*E*_CBS_^(T)^	mHa	cal/mol
B_2_	^3^Σ_g_^–^ (^3^B_1g_)	–49.091573	–0.215827	–0.197099	–0.018728	0.000	0.19
BC	^4^Σ^–^ (^4^A_2_)	–62.338439	–0.212447	–0.199256	–0.013192	0.001	0.73
BF	^1^Σ^+^ (^1^A_1_)	–124.168666	–0.389302	–0.375981	–0.013326	0.005	3.15
BH_3_	^1^A_1_^′^ (^1^A_1_)	–26.402604	–0.145460	–0.142554	–0.002907	0.001	0.47
BN	^3^Π (^3^A_2_)	–79.016991	–0.279379	–0.264722	–0.014659	0.002	1.30
BO	^2^Σ^+^ (^2^A_1_)	–99.562891	–0.351269	–0.334391	–0.016882	0.004	2.68
C_2_	^1^Σ_g_^+^ (^1^A_g_)	–75.406568	–0.402700	–0.366639	–0.036063	0.002	1.46
CF	^2^Π (^2^B_2_ or ^2^B_1_)	–137.233312	–0.432838	–0.416862	–0.015980	0.005	2.85
CH_4_	^1^A_1_ (^1^A′)	–40.216977	–0.239670	–0.232434	–0.007237	0.001	0.45
CN	^2^Σ^+^ (^2^A_1_)	–92.226844	–0.376526	–0.354525	–0.022003	0.002	1.40
CO	^1^Σ^+^ (^1^A_1_)	–112.791279	–0.413021	–0.393674	–0.019352	0.005	3.14
F_2_	^1^Σ_g_^+^ (^1^A_g_)	–198.775335	–0.618728	–0.596383	–0.022351	0.006	3.83
HF	^1^Σ^+^ (^1^A_1_)	–100.070354	–0.321450	–0.312636	–0.008817	0.003	2.09
H_2_	^1^Σ_g_^+^ (^1^A_g_)	–1.133586	–0.040825	–0.040825			
H_2_O	^1^A_1_ (^1^A_1_)	–76.067031	–0.306854	–0.297007	–0.009851	0.003	1.90
N_2_	^1^Σ_g_^+^ (^1^A_g_)	–108.994385	–0.425603	–0.404695	–0.020912	0.004	2.44
NF	^3^Σ^–^ (^3^A_2_)	–153.842385	–0.476019	–0.458676	–0.017347	0.005	3.13
NH_3_	^1^A_1_ (^1^A_1_)	–56.224867	–0.277820	–0.268538	–0.009284	0.002	1.08
NO	^2^Π (^2^B_2_ or ^2^B_1_)	–129.302103	–0.472274	–0.450210	–0.022069	0.006	3.51
O_2_	^3^Σ_g_^–^ (^3^B_1g_)	–149.668110	–0.529890	–0.506874	–0.023022	0.005	3.20
OF	^2^Π (^2^B_2_ or ^2^B_1_)	–174.204548	–0.548529	–0.528122	–0.020412	0.006	3.68

Many basis-set extrapolation schemes use eq 2 for the SCF energy extrapolations and eq 5 for the correlation components. However, since analytical expressions for these three-point extrapolations cannot be found (*vide supra*), one would need to numerically fit the parameters (i.e., *E*_CBS_ and either A and α or B and β). One thus has three to five equations (one for each *E*_*n*_ if using the DZ through 6Z members of the (aug-)­cc-pV*n*Z basis set family) with three unknowns, and one can fit the parameters by minimizing the MAD or root-mean-square deviation (RMSD) between *E*_*n*_ values obtained from the calculations and the extrapolations. Unfortunately, as discussed in more detail in SI section S5, one obtains significantly different results depending on the fitting conditions—hence the inherent ambiguity of this approach.

Consequently, the CBS energies were calculated for each molecule in the BSEF21 set using the energies calculated using the three-point exponential extrapolation (eq 22) with the quadruple-ζ through sextuple-ζ basis sets in the aug-cc-pV*n*Z series (hereinafter, this extrapolation set will be denoted as {Q,5,6}­ζ, with a similar notation for other CBS extrapolations). This was done both for each energy component separately as well as for the total correlation energy; the resulting *E*_CBS_ energies are summarized in Table 1. Also included in Table 1 are the differences in correlation energies (Δ*E*_CBS_^corr^) with a single β parameter (i.e., E_CBS_^corr^) and with two (i.e., E_CBS_^CCSD^ and E_CBS_^(T)^). These differences are very small—on the order of μHa (or cal/mol). One could simply use a single extrapolation parameter β for the two components of the correlation energy in high-accuracy thermochemistry applications, but in such methods, where every little difference matters, the use of separate β parameters would be advisible given the negligible additional cost. This would be especially relevant in methods like W1 and W2 that extrapolate the (T) contributions using basis sets one size smaller than those used for the SCF and CCSD contributions.^98,99^

From the data in Table 1, one can fit α, β, and/or γ parameters for 2BSEs. There are two schools of thought on how to fit the parameters. One could either independently fit α or β to the *E*_CBS_ for each component or simultaneously fit both parameters in a single fit to the total energy at the complete basis-set limit (*E*_CBS_^tot^ = *E*_CBS_^SCF^ + *E*_CBS_^CCSD(T)^); in their study, Neese and Valeev state that they opted for the second option.^8^ While the latter is likely to give lower errors due to the more flexible nature of the fitting, this potentially introduces unphysicality by adding error compensation into the system (i.e., the larger magnitude of the SCF energies compared to the other two allows for deviations from the best fits for the component energies to improve the overall error). However, this is not necessarily the best fit for the individual components of the energy.

If one plots on a grid the MAD of the extrapolated energy as a function of α_SCF_ and β_CCSD(T)_ for a {T,Q}­ζ extrapolation relative to the extrapolated *E*_CBS_^SCF^ and *E*_CBS_^corr^ in Table 1 (Figure 2A,B), one has a trough of reasonable α_SCF_ and β_CCSD(T)_ values. The bottom of the trough (Figure 2C) is S-shaped with a plateau for each arm of the trough. The global minimum is α_SCF_ = 1.581 and β_CCSD(T)_ = 16.733; the latter is very different from the β = 3 originally proposed by Helgaker et al.,^7^ the β = 2.02 proposed by Truhlar and co-workers,^87,88^ or the value β = 3.05 from Neese and Valeev.^8^ In fact, the global minimum is on one branch of the trough while these other values are all on the other. On the other hand, if one considers each component independently (Figure 2D) one obtains minima at α_SCF_ = 5.771, β_CCSD(T)_ = 3.339, β_CCSD_ = 3.404 and β_(T)_ = 3.361. For comparison, for a {T,Q}­ζ extrapolation with the same aug-cc-pV*n*Z basis sets, Neese and Valeev obtain α_SCF_ = 5.79 and β_CCSD(T)_ = 3.05.^8^

**Figure 2:**
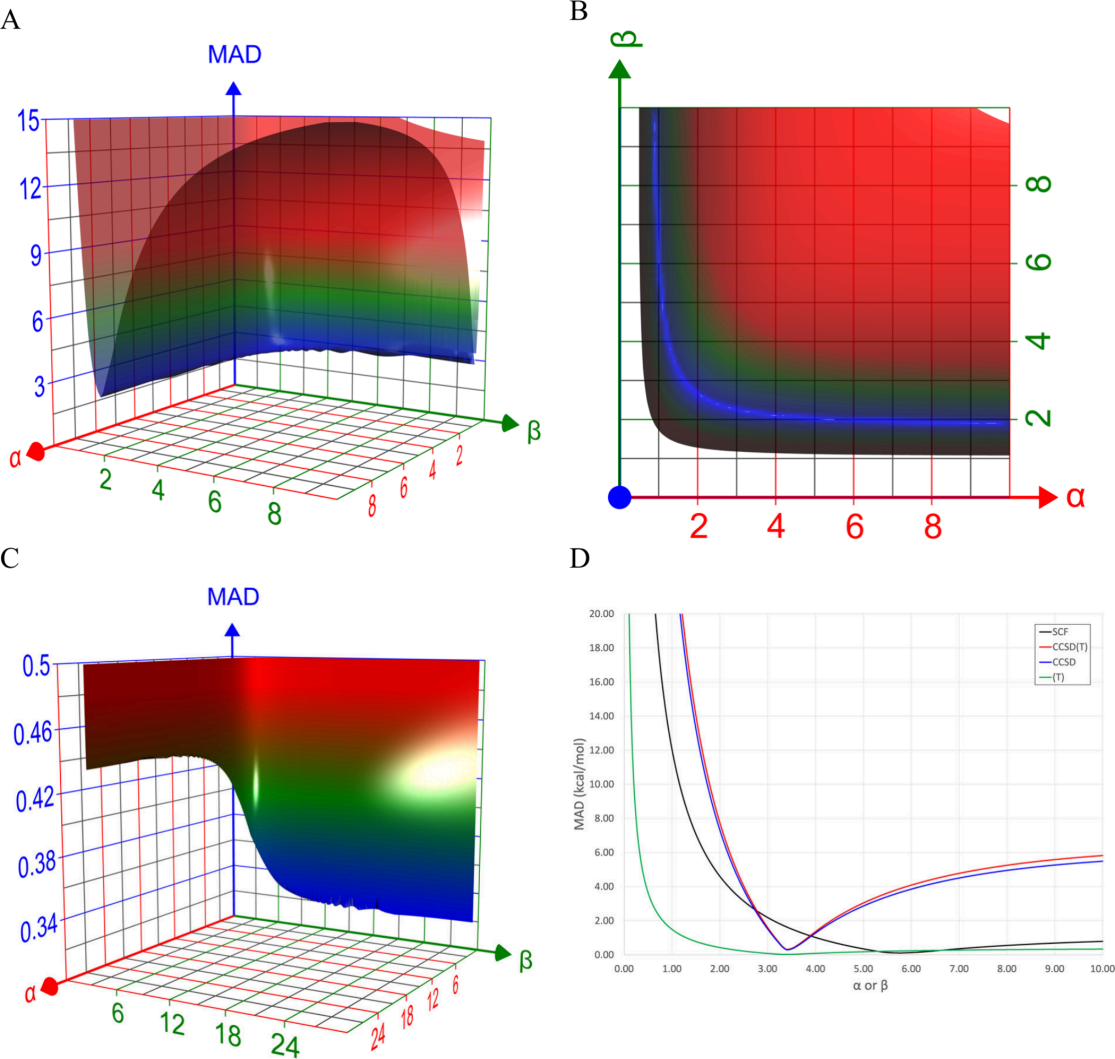
Plot of the MAD (kcal/mol) of the total {T,Q}­ζ extrapolated energies relative to *E*_CBS_ in Table 1. (A) Full MAD surface where α_SCF_ and β_CCSD(T)_ were scanned in the range of 0.1–10.0 in steps of 0.1. (B) Top view showing the contour of the MAD; note the narrow blue trough of low MAD values. (C) Plot of the low MAD regime (≤0.5 kcal/mol) of an extended scan range (up to α_SCF_/β_CCSD(*T*)_ = 30.00) with a finer grid (0.01 spacing); the global minimum with a 0.001 spacing (which results in too many points to plot) corresponds to α_SCF_ = 1.581 and β_CCSD(T)_ = 16.733 with a MAD of 0.32 kcal/mol. (D) Plot at a resolution of 0.001 of the MAD of each component independently as a function of α or β as appropriate, with minima at α_SCF_ = 5.771, β_CCSD(T)_ = 3.339, β_CCSD_ = 3.404, and β_(T)_ = 3.361.

Ranasinghe and Petersson noted that using a Schwenke-type form (eq 11) one has a smoother parameter optimization.^90^ The analogous plot of MAD as a function of *f*_SCF_ and *f*_CCSD(*T*)_ is shown in Figure 3A,B. Now a straight trough is obtained with a smooth contour and a clear minimum. In fact, if one plots for each *f*_SCF_ point on the grid the corresponding *f*_CCSD(T)_ that is a minimum in MAD, then one obtains a straight line (Figure 3C). Moreover, the minimum MAD contour is smooth with a clear minimum (see Figures 3B and S1). Thus, there is a clear set of optimal values, with the global minimum (within the scanned range) at *f*_SCF_ = 1.899 and *f*_CCSD(T)_ = 0.007, which correspond to α_SCF_ ≈ 1.579 and β_CCSD(T)_ ≈ 17.272. Plotting the MAD of each energy component independently as functions of *f* gives V-shaped curves with global minima at *f*_SCF_ = 0.271 and *f*_CCSD(T)_ = 0.603, which correspond to α_SCF_ ≈ 5.768 and β_CCSD(T)_ ≈ 3.399. The concurrent fitting of both *f* parameters again introduces unphysicality to the system, and this is a clear indication, again, that it is recommended to consider each component independent of the others.

**Figure 3:**
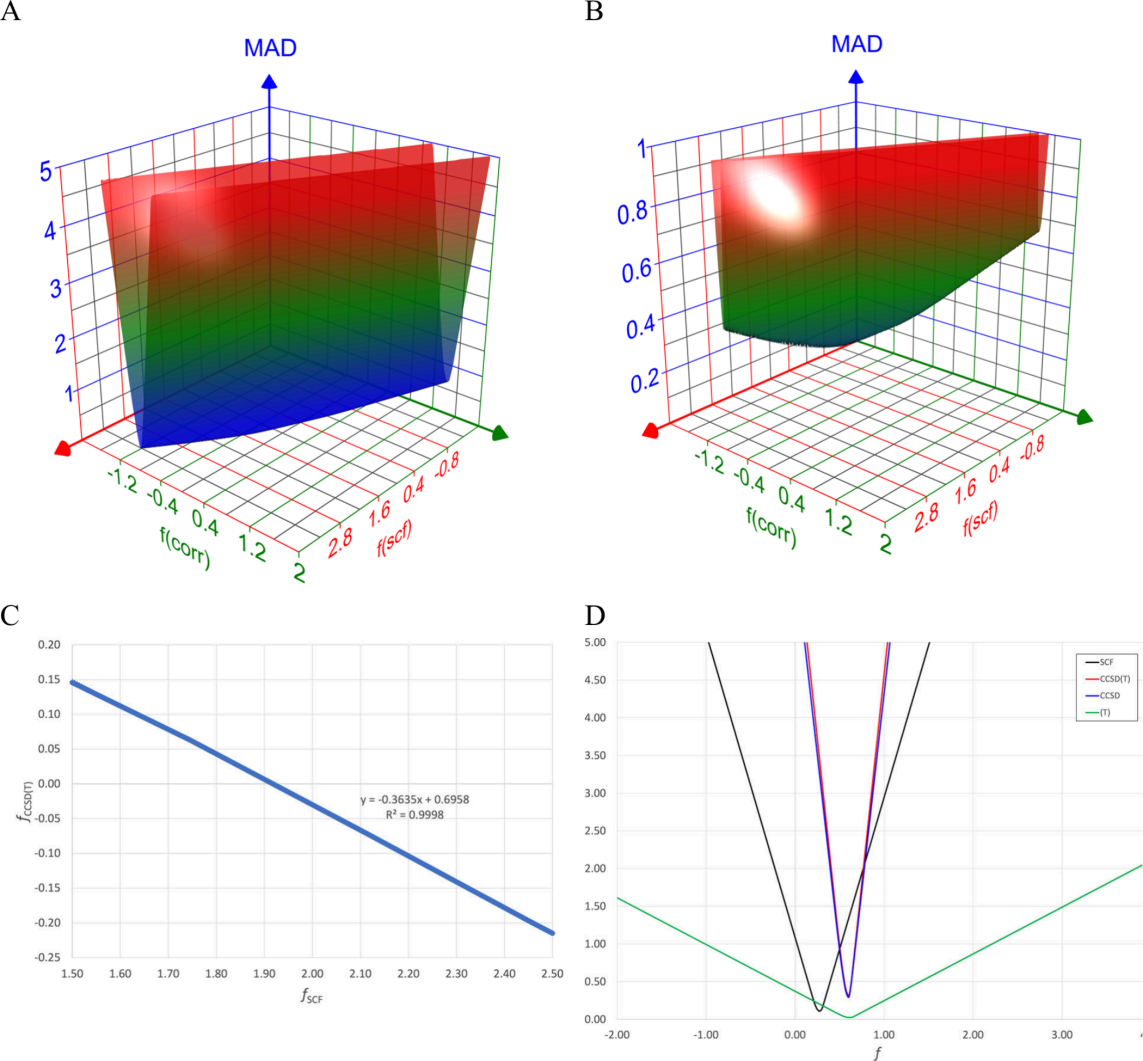
Plot of the MAD (kcal/mol) of the total {T,Q}­ζ-extrapolated energies (i.e., using the aug-cc-pVTZ and aug-cc-pVQZ basis sets) relative to *E*_CBS_ in Table 1. *f*_SCF_ and *f*_CCSD(T)_ were scanned in the range of −2.0 to 4.0 in steps of 0.01. The global minimum corresponds to *f*_SCF_ = 1.89 and *f*_CCSD(T)_ = 0.01 with a MAD of 0.32 kcal/mol; with a finer grid with a 0.001 spacing (which results in too many points to plot), the global minimum corresponds to *f*_SCF_ = 1.899 and *f*_CCSD(T)_ = 0.007 with a MAD of 0.32 kcal/mol. (A) Full MAD surface. (B) Enlargement of the MAD = 0.0–1.0 kcal/mol regime. (C) Trace of the minimum MAD contour of panel A; the MAD along this line is plotted in Figure S1. (D) Plot of MAD of each component as a function of *f* scanned in the range of −2.0 to 4.0 in steps of 0.001 with minima at for *f*_SCF_ = 0.271, *f*_CCSD(T)_ = 0.603, *f*_CCSD_ = 0.601 and *f*_(T)_ = 0.613.

Another observation from Figure 3D (and Figure 2D) is that the (T) correlation energy is weakly dependent on the extrapolation parameter *f*_(T)_ (or β_(T)_)—the plot of the MAD versus the extrapolation parameter is much flatter. This explains the small differences in the CBS values in Table 1 whether the CCSD­(T) correlation energy was extrapolated as a single entity or whether the two components were extrapolated independently (i.e., Δ*E*_CBS_^corr^). This, along with the very close minima, also explains why the CCSD­(T) and CCSD curves almost completely overlap. Thus, as noted above, one could in principle use a single extrapolation parameter *f*_CCSD(T)_, although using separate extrapolation parameters would add zero additional computational cost.

### 3.4 Expanding the Evaluation Set to Include Second-Row Elements

The initial evaluations of the fitting procedures used basically the same set used by Neese and Valeev (i.e., BSEF21) for consistency, but this set includes only first-row elements. For a more balanced evaluation, the fitting set was expanded to include second-row elements (i.e., BSEF74, see section 3.1); the CBS energies for the new systems are listed in Table S6. Using this expanded dataset, the Schwenke-type CBS parameters were reoptimized by minimizing the MAD between the two-parameter extrapolated energies and the {Q,5,6}­ζ CBS limits. The Schwenke parameters for the various components of CCSD­(T) with the aug-cc-pV*n*Z basis sets optimized against each dataset are shown in Table 2. There are small changes in the parameters when the parameters are reoptimized with the bigger dataset, but the changes in the MADs are very small. If one uses the parameters fit with the smaller dataset to evaluate the larger one (denoted as “BSEF74@BSEF21”), there is a slight increase in the MADs, but not enough to invalidate calculations using the former.

**Table 2:** Schwenke-Type Extrapolation Parameters *f* and MAD (kcal/mol) for the Various CCSD­(T) Components for the aug-cc-pV*n*Z Family of Basis Sets Optimized with the BSEF21 and BSEF74 Datasets and the MAD for the BSEF74 Dataset with the BSEF21-Optimized Parameters

	*f*				MAD			
component	{D,T}ζ	{T,Q}ζ	{Q,5}ζ	{5,6}ζ	{D,T}ζ	{T,Q}ζ	{Q,5}ζ	{5,6}ζ
BSEF21								
SCF	0.3481	0.2707	0.1130	0.1202	0.42	0.11	0.01	0.00
CCSD(T)	0.5296	0.6029	0.6794	0.6794	1.23	0.31	0.08	0.03
CCSD	0.5422	0.6016	0.6874	0.6874	1.14	0.29	0.07	0.03
(T)	0.3699	0.6137	0.5565	0.5565	0.05	0.03	0.00	0.00
BSEF74								
SCF	0.3352	0.2688	0.1851	0.2562	0.35	0.08	0.07	0.01
CCSD(T)	0.5168	0.6029	0.6794	0.6794	1.06	0.31	0.08	0.03
CCSD	0.5271	0.6045	0.6902	0.6918	1.02	0.23	0.16	0.07
(T)	0.4111	0.6192	0.5742	0.5816	0.09	0.03	0.01	0.01
BSEF74@BSEF21								
SCF					0.38	0.08	0.09	0.02
CCSD(T)					1.12	0.31	0.08	0.03
CCSD					1.09	0.23	0.16	0.07
(T)					0.13	0.03	0.02	0.01

The relative performances of the various two- and three-point extrapolations are shown in Figure 4. It is not surprising that the spread of errors (i.e., width of the box) decreases as the cardinal numbers of the two-point extrapolations increases (i.e., going from {D,T}­ζ to {Q,5}­ζ the spread of errors significantly decreases). However, the three-point extrapolations, which do not involve any fitted parameters, perform significantly worse than the corresponding two-point extrapolations that do have fit parameters (i.e., compare {D,T,Q}­ζ versus {T,Q}­ζ); the fitting parameter likely introduces a measure of error correction thereby improving the two-parameter fits.

**Figure 4:**
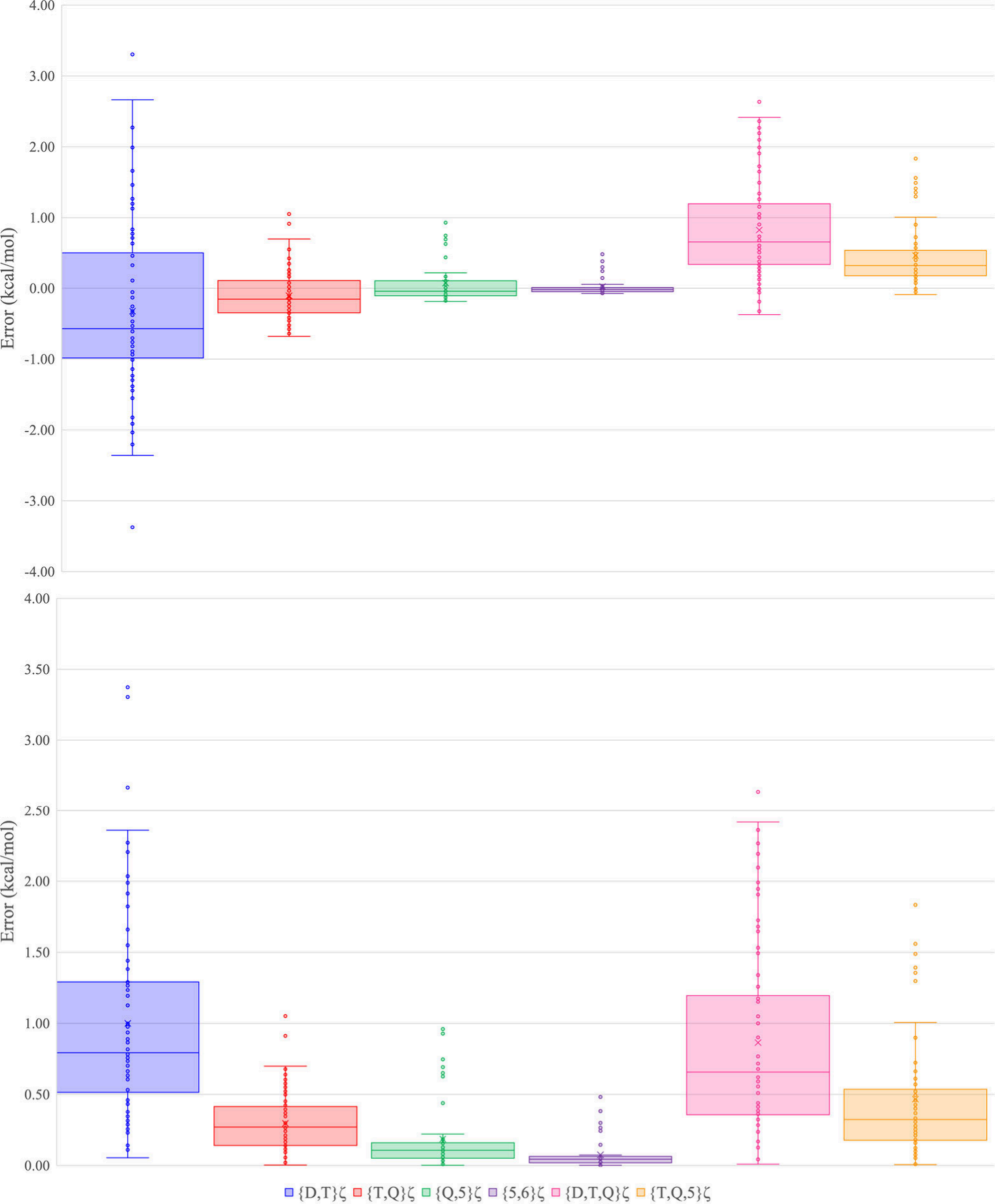
Boxplots showing the performance of each two- and three-basis-set extrapolations of the total energy with the aug-cc-pV*n*Z basis sets relative to the {Q,5,6}­ζ benchmark values for the BSEF74 benchmark dataset. (Top) Signed errors defined as *E*_CBS_^*i*^ – *E*_CBS_^{Q,5,6}ζ^. (Bottom) Absolute errors. Each energy component is summed independently. In each boxplot, the errors (in kcal/mol) are plotted using small circles for each molecule, while the × indicates the average of all values. The box contains the second and third quartiles (i.e., 50% of the data) while the line in the middle of the box is the median. The whiskers (vertical lines extending from the boxes) mark innermost of the smallest/largest value that is within 150% of the interquartile region (i.e., the width of the box).

It was questioned whether the underperformance of the three-point extrapolations was due to the inclusion of a smaller basis relative to the corresponding two-point extrapolation (e.g., comparing {T,Q,5}­ζ and {Q,5}­ζ). From the results in Figure 4, indeed the latter preforms significantly better. However, following this argument, one would expect including a larger basis set in the extrapolation (e.g., comparing {T,Q,5}­ζ and {T,Q}­ζ) would improve the performance, yet the latter still performs better. The hypothesis here is that the better performance with the two-point extrapolations stems from the fitting of the Schwenke fitting parameter *f* that will naturally introduce some measure of error compensation and more evenly splays the errors around 0.0 kcal/mol errors (i.e., in Figure 4 (top) the boxes for the two-point extrapolations are approximately centered around 0.0 kcal/mol while the boxes for the three-point extrapolations are significantly above indicating that the three-point extrapolations systematically underestimate the CBS energies).

The BSE parameter fitting procedure was repeated for a selection of basis sets against the {Q,5,6}­ζ CBS values. The *f*-parameters for the two-point extrapolations to the aug-cc-pV*n*Z CBS limit are given in Tables S7–S9, and, for comparison, the corresponding α, β and γ values are given in Table S10.

### 3.5 Other Correlation Methods: QCISD­(T) and MP2

The next question to consider is whether parameters obtained using coupled-cluster are relevant to other correlation methods. For instance, the compound method files included in the latest release of Orca6 use β obtained from CCSD­(T) for MP2 (e.g., the EP2 extrapolation based on Liakos et al.^100^). However, is there a basis for this practice? To this end, the fitting process was repeated for QCISD­(T) and MP2; the CBS energies for each are given in Table S11 and the extrapolation parameters are given in Table 3. Here, only the QCISD­(T) or MP2 correlation energies are considered since the SCF energy here is (obviously) the same as for CCSD­(T). The MAD as a function of *f*_corr_ are plotted in Figure 5.

**Table 3:** Schwenke *f* Extrapolation Parameters for Two-Basis-Set Extrapolations and Associated Mean Absolute and Signed Deviations (MAD and MSD, Respectively, in kcal/mol) for Dunning’s aug-cc-pV*n*Z and cc-pV*n*Z Basis Sets for Various WFT Methods; See Tables S7–S10 for Other Basis Sets Considered in This Study

	*f*				MAD				MSD^a^			
component	{D,T}ζ	{T,Q}ζ	{Q,5}ζ	{5,6}ζ	{D,T}ζ	{T,Q}ζ	{Q,5}ζ	{5,6}ζ	{D,T}ζ	{T,Q}ζ	{Q,5}ζ	{5,6}ζ
aug-cc-pV*n*Z												
SCF	0.3352	0.2688	0.1841	0.2562	0.35	0.07	0.07	0.01	–0.03	–0.01	0.03	0.00
CCSD(T)	0.5183	0.6029	0.6794	0.6794	1.06	0.31	0.08	0.03	–0.32	–0.19	–0.03	–0.01
CCSD	0.5271	0.6045	0.6918	0.6922	1.03	0.23	0.17	0.07	–0.28	–0.09	0.04	0.02
(T)	0.4111	0.6210	0.5759	0.5830	0.10	0.03	0.02	0.01	0.01	–0.01	0.01	0.00
QCISD(T)	0.5169	0.6029	0.6788	0.6788	1.06	0.31	0.08	0.03	–0.32	–0.19	–0.03	–0.01
QCISD	0.5266	0.6042	0.6897	0.6914	1.04	0.23	0.17	0.07	–0.32	–0.09	0.04	0.02
(T) of QCISD(T)	0.4115	0.6167	0.5860	0.5883	0.10	0.03	0.02	0.01	0.02	–0.01	0.01	0.00
MP2	0.6738	0.7461	0.8163	0.8163	1.20	0.28	0.06	0.03	–0.16	–0.11	–0.02	–0.01
SCF+MP2^b^	0.5561	0.6389	0.7102	0.7183	1.37	0.78	0.51	0.23	0.29	0.31	0.20	0.09
cc-pV*n*Z												
SCF	0.3201	0.2774	0.2087	0.3086	0.34	0.11	0.08	0.02	0.01	0.00	0.04	0.00
CCSD(T)	0.5322	0.6165	0.6956	0.7479	1.15	0.37	0.07	0.04	–0.24	–0.17	–0.03	–0.02
CCSD	0.5418	0.6208	0.7132	0.7610	1.10	0.27	0.19	0.07	–0.42	–0.11	0.08	0.02
(T)	0.4962	0.5995	0.6254	0.6958	0.32	0.22	0.22	0.21	–0.22	–0.20	–0.19	–0.20
QCISD(T)	0.5315	0.6164	0.6953	0.7472	1.15	0.37	0.07	0.04	–0.25	–0.17	–0.03	–0.02
QCISD	0.5400	0.6197	0.7125	0.7605	1.11	0.28	0.19	0.07	–0.40	–0.11	0.08	0.02
(T) of QCISD(T)	0.4982	0.6022	0.6363	0.7016	0.15	0.03	0.02	0.01	–0.02	0.00	0.01	0.00
MP2	0.6797	0.7692	0.8548	0.9026	1.09	0.27	0.08	0.04	–0.14	–0.08	–0.02	–0.01
SCF+MP2^b^	0.5579	0.6675	0.7595	0.8358	1.49	0.80	0.65	0.25	0.07	0.19	0.26	0.05

a Defined as *E*_CBS_^*i*^ – *E*_CBS_^{Q,5,6}ζ^.

b Optimization of a single parameter for the total MP2 energy.

**Figure 5:**
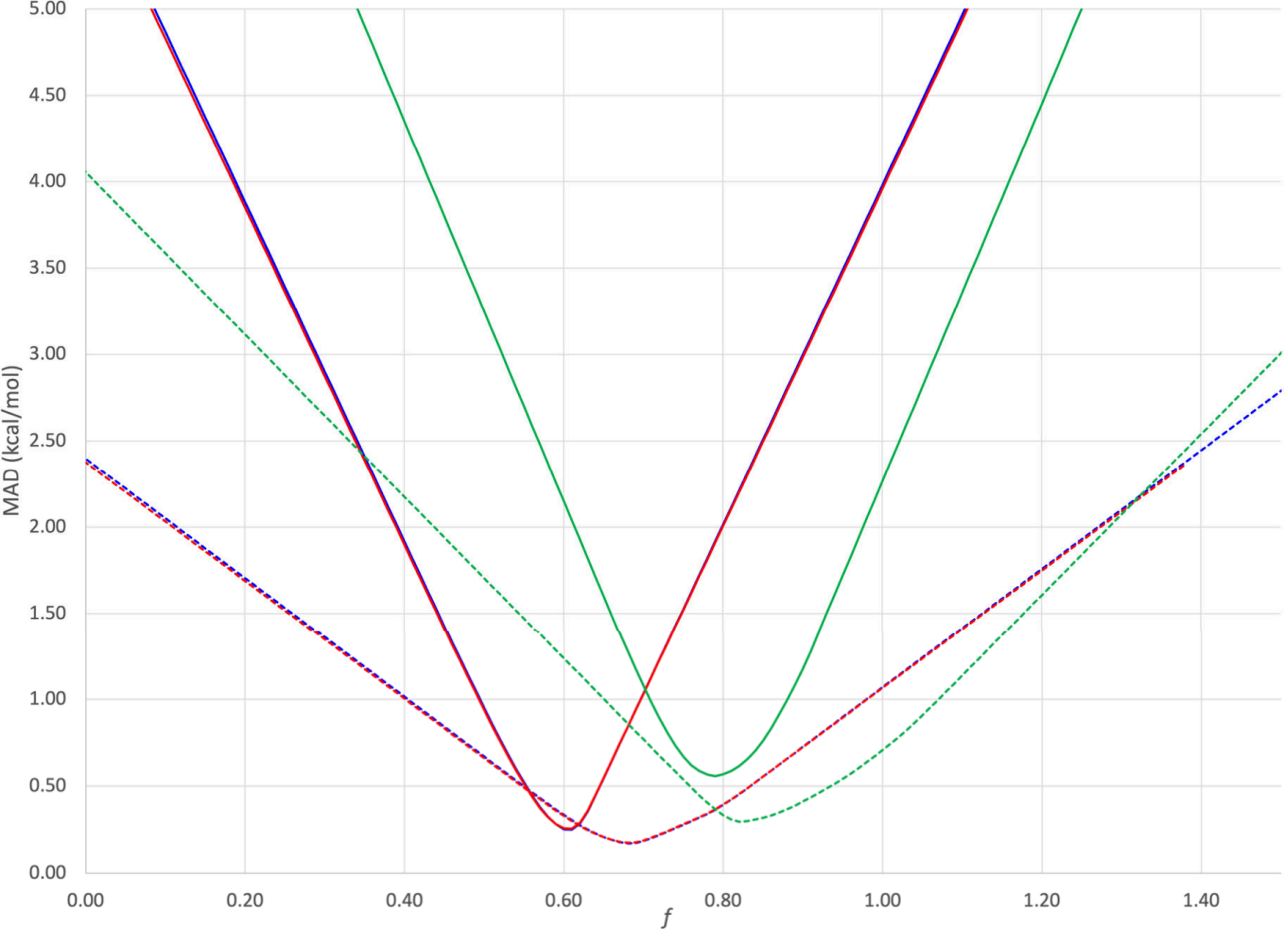
Plot of the MAD as a function of the correlation extrapolation parameter *f*, scanned in the range of −2.0 to 4.0 in steps of 0.01 (only the section around the minima is shown) for the CCSD­(T) (blue), QCISD­(T) (red), and MP2 (green) correlation energies with {T, Q}­ζ (solid lines) and {Q, 5}­ζ (dashed lines) basis-set extrapolations using Dunning’s aug-cc-pV*n*Z basis sets; note that the lines for CCSD­(T) and QCISD­(T) are so close as to be almost visually indistinguishable.

The mean absolute and signed deviations (MAD and MSD) are given in Table 3. Overall, the MADs are reasonably small: the largest is for the SCP+MP2 {D,T}­ζ extrapolation, and with larger basis set pairs they approach zero. The MSDs are generally close to zero, indicating that there is no systematic bias in the errors. The fitting of the *f* parameter against MAD is naturally going to localize the individual errors around zero. The fact that they are (generally) slightly negative implies that the two-basis-set CBS energies slightly overshoot the {Q,5,6}­ζ benchmarks, except for the SCF+MP2 total energy.

It is not surprising that the plots for QCISD­(T) and CCSD­(T) (Figure 5) nearly overlap. The two methods have a related underlying motivation and are the equivalent at the full-CI (FCI) limit. One can safely use parameters fit for one for the other. MP2 on the other hand, based on perturbation theory, behaves quite differently, and the optimal *f*_MP2_ values are significantly higher. One would therefore be advised to reconsider using the same parameters as for the CCSD­(T) correlation.

But really how significant is the choice of parameters? To this end, two benchmark datasets were used: Řezáč et al.’s S66 set of noncovalent interactions^80,81^ and Goerigk et al.’s mindless benchmarking set of 43 16-atom fictitious molecules (MB16-43);^82,83^ both are part of Goerigk et al.’s GMTKN55 database.^82^ The MADs of the CBS values determined with each set of parameters (fit to CCSD­(T) and fit to MP2 correlation energies) relative to the published reference values are given in Table 4. For S66, the differences are negligible: ΔMAD = 0.06 kcal/mol for the {D,T}­ζ extrapolation and even smaller for the bigger basis sets. However, S66 is comprised of well-behaved systems, in contrast to MB16-43, which is a more arduous test (S66 has reference reaction energies in the range of 1.4–19.5 kcal/mol compared to MB16-43’s 19.9–1290.7 kcal/mol). Not surprisingly, MP2 does rather poorly for this dataset (MAD > 20 kcal/mol). Moreover, there is significant sensitivity to the choice of parameter set used, but whether this is a deficiency of the extrapolations or MP2 is unclear.

**Table 4:** Evaluation of MP2 Basis-Set Extrapolations over the S66 and MB16-43 Benchmark Sets Using MP2- and CCSD­(T)-Optimized Schwenke SCF and Correlation CBS *f* Parameters Optimized against Either the BSEF74 or BSEF21 Benchmark Dataset

	optimized against BSEF74			optimized against BSEF21		
CBS	MP2-optimized	CCSD(T)-optimized	ΔMAD	MP2-optimized	CCSD(T)-optimized	ΔMAD
S66						
{D,T}ζ	2.13	2.19	0.06	2.14	2.18	0.04
{T,Q}ζ	1.79	1.84	0.05	1.79	1.84	0.05
{Q,5}ζ	1.76	1.78	0.02	1.76	1.78	0.02
MB16-43						
{D,T}ζ	23.43	23.24	–0.19	29.23	27.51	–1.72
{T,Q}ζ	61.89	56.05	–5.84	60.14	54.36	–5.27
{Q,5}ζ	49.89	22.05	–27.84	57.36	51.29	–6.07

### 3.6 Double Hybrid Functionals

It is established that MP2 is generally not a method of choice for accurate thermochemical calculations, even surpassed by many DFT functionals (*cf.* WTMAD2 values for the GMTKN55 benchmark set^82^ of 6.91 kcal/mol for MP2 and 5.35 kcal/mol for SCS-MP2 compared to 6.37, 4.79, 3.29, 3.10, and 2.19 kcal/mol for B97M-V, M06-2X, ωB97M-V, DSD-PBEP86, and ωB97M(2), respectively^10^). On the other hand, double-hybrid (DH) functionals, which include an MP2-like correlation component, have been shown to have accuracies even approaching that of coupled-cluster methods.^10^ There are occasions where the CCSD­(T)-optimized parameters were used to extrapolate energies from a DH functional, such as Dohm et al.’s corrections^18^ to the author’s MOBH35 benchmark set.^19^ Thus it was decided to evaluate how a typical DH functional, DSD-PBEP86,^59^ fares on these benchmark tests. (Technical note: the D3BJ empirical dispersion correction was omitted during the basis-set extrapolations since it depends solely on the geometry and is independent of the basis set used.)

There are two approaches one could consider for extrapolating double-hybrid functionals to the basis set limit: extrapolate the Kohn–Sham (*E*_KS_^DH^) and MP2-like (*E*_MP2_^DH^) components separately, in analogy to a typical MP2 calculation, or extrapolate the final total energy (*E*_tot_^DH^). *A priori* there does not seem to be a basis for choosing one option over the other. The CBS energies of the BSEF74* set were determined in the same manner as for the WFT methods (i.e., a {Q,5,6}­ζ extrapolation using eq 22with Dunning’s aug-cc-pVnZ basis sets) for both *E*_tot_^DH^ and its two components *E*_KS_^DH^ and *E*_MP2_^DH^. The CBS energies are given in Table S12 and the corresponding fitted two-level (Schwenke) CBS extrapolation parameters and associated MADs in Table 5. The boxplot in Figure 6 shows that the errors decrease with increasing basis set size, with the {Q,5}­ζ extrapolations having all errors below 2 kcal/mol. Again, the three-point extrapolations have larger error spreads than the corresponding two-point extrapolations.

**Table 5:** Schwenke *f* Extrapolation Parameters for Two-Basis-Set Extrapolations and Associated Mean Absolute and Signed Deviations (MAD and MSD, Respectively, in kcal/mol) for Dunning’s aug-cc-pV*n*Z Basis Sets for the Kohn–Sham, MP2-like Correlation and Total Double-Hybrid DFT Energy for Selected Double-Hybrid Functionals; see Tables S19–S25 for All Other Basis Sets Considered in This Study

		*f*				MAD				MSD^a^			
functional	component	{D,T}ζ	{T,Q}ζ	{Q,5}ζ	{5,6}ζ	{D,T}ζ	{T,Q}ζ	{Q,5}ζ	{5,6}ζ	{D,T}ζ	{T,Q}ζ	{Q,5}ζ	{5,6}ζ
B2PLYP	KS	0.3727	0.3784	0.1955	0.2119	0.43	0.30	0.05	0.01	0.11	0.04	–0.01	0.00
	MP2	0.6263	0.7383	0.7883	0.7903	0.34	0.12	0.05	0.03	–0.05	0.02	0.03	0.01
	KS+MP2	0.4714	0.5410	0.4606	0.4625	0.72	0.51	0.13	0.04	0.00	0.02	0.01	0.01
B2GP-PLYP	KS	0.3607	0.3297	0.1907	0.2265	0.35	0.16	0.05	0.01	0.01	0.02	0.01	0.00
	MP2	0.6387	0.7433	0.7961	0.7961	0.45	0.16	0.07	0.03	–0.05	0.04	0.04	0.02
	KS+MP2	0.4800	0.5361	0.5226	0.5282	0.73	0.47	0.19	0.07	0.08	0.13	0.07	0.03
mPW2PLYP	KS	0.3852	0.4134	0.1941	0.1952	0.53	0.43	0.05	0.01	0.15	0.07	–0.01	0.00
	MP2	0.6180	0.7351	0.7837	0.7886	0.31	0.11	0.05	0.02	–0.05	0.02	0.02	0.01
	KS+MP2	0.4709	0.5523	0.4192	0.4342	0.79	0.61	0.12	0.04	0.05	0.03	0.02	0.00
PWPB95	KS	0.3829	0.3778	0.1867	0.1901	0.50	0.31	0.03	0.01	0.07	0.05	0.00	0.00
	MP2	0.7035	0.7777	0.8239	0.8239	0.75	0.20	0.08	0.04	–0.08	0.01	0.04	0.02
	KS+MP2	0.5804	0.6499	0.6502	0.6576	1.46	0.84	0.34	0.13	–0.38	0.04	–0.04	–0.01
DSD-PBEP86^b^	KS	0.3528	0.3028	0.1870	0.2388	0.35	0.08	0.06	0.01	–0.01	0.00	0.02	0.00
	MP2	0.6902	0.7705	0.8215	0.8215	0.87	0.27	0.12	0.06	–0.18	0.03	0.05	0.03
	KS+MP2	0.5574	0.6225	0.6637	0.6751	1.18	0.68	0.38	0.16	–0.10	0.18	0.15	0.06
revDSD-PBEP86-D3BJ^b^	KS	0.3529	0.3032	0.1869	0.2388	0.35	0.08	0.06	0.01	–0.01	0.00	0.02	0.00
	MP2	0.7146	0.7849	0.8396	0.8400	0.93	0.28	0.13	0.06	–0.20	0.02	0.05	0.03
	KS+MP2	0.5733	0.6376	0.6820	0.6938	1.26	0.71	0.40	0.17	–0.07	0.18	0.16	0.06
revDSD-PBEP86-D4^b^	KS	0.3529	0.3033	0.1866	0.2390	0.35	0.08	0.06	0.01	–0.01	0.00	0.02	0.00
	MP2	0.7175	0.7866	0.8404	0.8407	0.94	0.28	0.13	0.06	–0.20	0.02	0.06	0.03
	KS+MP2	0.5752	0.6391	0.6838	0.6957	1.27	0.71	0.40	0.18	–0.06	0.19	0.17	0.06

a Defined as *E*_CBS_^*i*^ – *E*_CBS_^{Q,5,6}ζ^.

b The associated dispersion corrections were not included in the basis-set extrapolations since they are solely dependent on the geometry.

**Figure 6:**
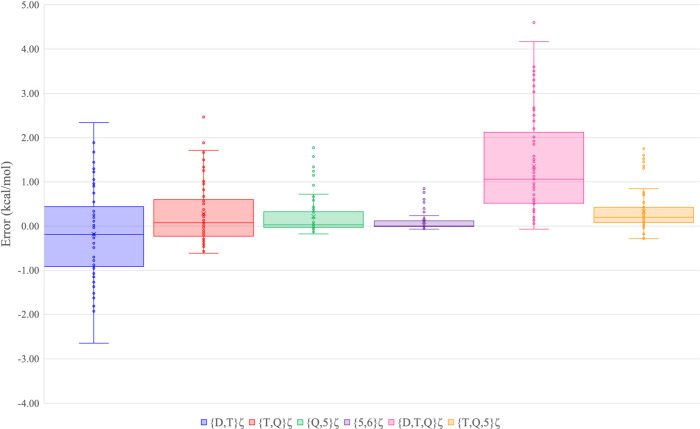
Boxplots showing the performance (MSD) of each two- and three-basis-set extrapolation of the total energy with DSD-PBEP86 and the aug-cc-pV*n*Z basis sets relative to the {Q,5,6}­ζ benchmark values for the BSEF74 benchmark dataset. See Figure 4 for a description of the boxplots.

For both the S66 and MB16-43 datasets, there is significant improvement when using a double-hybrid functional over MP2. There is a very slight advantage of using two parameters (Table 6, ΔMAD < 1 kcal/mol for a {T,Q}­ζ CBS extrapolation). Given the (semi)­empirical nature of DFT functionals (including double-hybrids), it is very reasonable to use a single parameter; however, as noted above, using two would add some increased accuracy at zero additional computational cost. Likewise, the advantage of using DH- over CCSD­(T)-optimized parameters is surprisingly negligible (Table 7).

**Table 6:** Evaluation of DSD-PBEP86 Basis-Set Extrapolations over the S66 and MB16-43 Benchmark Sets Using Schwenke *f* CBS Parameters Optimized for *E*_tot_ and Independently for *E*_KS_ and *E*_MP2_

CBS	*E*_tot_	*E*_KS_ + *E*_MP2_	ΔMAD
S66			
{D,T}ζ	0.83	0.91	0.08
{T,Q}ζ	0.76	0.77	0.01
{Q,5}ζ	0.95	0.95	0.00
MB16-43			
{D,T}ζ	7.37	5.70	–1.67
{T,Q}ζ	4.89	4.68	–0.21
{Q,5}ζ	5.27	5.27	0.00

**Table 7:** Evaluation of DSD-PBEP86 Basis-Set Extrapolations over the S66 and MB16-43 Benchmark Sets Using Schwenke *f* CBS Parameters Optimized for DSD-PBEP86 (*E*_KS_ and *E*_MP2_ Optimized Independently) and for CCSD­(T)

CBS	DSD-optimized	CCSD(T)-optimized	ΔMAD
S66			
{D,T}ζ	0.91	0.95	0.04
{T,Q}ζ	0.77	0.80	0.03
{Q,5}ζ	0.95	0.95	0.00
MB16-43			
{D,T}ζ	5.70	4.87	0.83
{T,Q}ζ	4.68	4.75	–0.07
{Q,5}ζ	5.27	5.23	0.04

These CBS fittings were repeated for other selected DH functionals: B2PLYP, B2GP-PLYP, mPW2PLYP, PWPB95, revDSD-PBEP86-D3BJ, and revDSD-PBEP86-D4. The resulting extrapolation parameters are listed in Table 5 for aug-cc-pV*n*Z and in Tables S19–S25 for other families of basis sets, while the *f* and related α, β, and γ parameters are given in Table S26.

## 4 Conclusions

While basis-set extrapolation is common practice, there were a number of open questions regarding its use, which this study attempted to address. It was found that simultaneously optimizing parameters for both the Hartree–Fock (SCF) and CCSD­(T) correlation contributions leads to values that are inconsistent with the behavior observed when each parameter is optimized independently. Likewise, the practice of using a single parameter for the CCSD­(T) correlation energy, rather than one for each component (i.e., CCSD and (T)), is reasonable given the very small differences in energy. Likewise, the consequences of using extrapolation parameters optimized for CCSD­(T) for other correlation schemes, such as QCISD­(T) or MP2, does not introduce any significant error. Commonly used extrapolation schemes often take their parameters from the study by Neese and Valeev;^8^ while these might not be the ideal values, the evaluations against the S66 and MB16-43 benchmark sets show that the resulting differences are small. Finally, the use of a Schwenke-type extrapolation is recommended, especially for fitting the extrapolation parameters; the three other extrapolation schemes considered in this study (exponential–square root, inverse power, and exponential) can all be expressed in terms of a Schwenke-type extrapolation. Furthermore, it was shown that for extrapolations of two consecutive basis sets, since all extrapolation schemes reduce to a Schwenke-type extrapolation, the different extrapolation schemes are mathematically equivalent and given one extrapolation parameter one can find the other two. Double-hybrid DFT functionals were also considered; the CCSD­(T)-optimized parameters that have been used still give reasonable extrapolations, and there is generally only a small advantage to optimizing a single parameter for the total energy or two parameters, one each for the Kohn–Sham and MP2-like energies.

The BSEF74 benchmark set is comprised primarily of diatomics. While they cover a range of electronic states, one has to ask whether a larger reference set would give different results, especially considering the differences between extrapolation methods can be small. The question of how to expand the reference dataset is tricky. If one were to add the S66 test set, then one would be concerned that the fitting favors closed-shell organic systems. One could add additional datasets but would always be worried whether the reference set is balanced—until one reaches GMTKN55. The problem then becomes computational resource limitations: the larger reference set means more energies to calculate, the larger molecules mean more expensive calculations (which do not scale linearly!), and at some point the aug-cc-pV6Z energy calculations are going to become far too expensive to calculate. In such a situation, one would be forced to rely on either using aug-cc-pV5Z for the upper basis set in the extrapolations (until it too becomes too expensive) or resort to methods like resolution of the identity (RI) to make the calculations more feasible (but this introduces a whole new dimension of complexity—what is the source of the observed small differences, the extrapolation or the RI approximation or potentially both?). After all this additional cost and effort, the most likely scenario is that the conclusions of this study will not change. In the end, the use of the BSEF74 reference dataset likely provides a nice balance of cost and return.

In summary, while the extrapolation parameters commonly used may not be ideal, the resulting errors are very small, meaning that there is no real reason to reevaluate current benchmark values. That being said, moving forward one would be advised to consider using the revised parameters from this study. This becomes more important as one starts considering high-accuracy thermochemistry where every small error is significant.

## Supplementary Material









## Notes

Discussion on the omission of def2-TZVP and def2-TZVPD from this study, geometries and electronic properties of the BSEF74 test set, derivations of the Schwenke-type equations for each extrapolation method, derivation of the equation for the exponential extrapolation to the CBS limit for three consecutive basis sets, further discussion on the extrapolations to the CBS limit, plot of the MAD along the trace of the contour of the MAD surface as a function of *f*_SCF_ and *f*_CCSD(T)_ (Figure S1), tables of the CBS energies for the methods considered (Tables S6 and S11–S18), tables of the Schwenke *f* extrapolation parameters for various methods (Tables S7–S10 and S19–S26), and the corrected ANO-cc-pVTZ basis set in Molpro, Gaussian, and Orca formats (PDF)
Corrected ANO-cc-pVTZ basis set in Orca format (TXT)
Corrected ANO-cc-pVTZ basis set in Gaussian format (TXT)
Corrected ANO-cc-pVTZ basis set in Molpro format (TXT)
