# Supplementary material for: On the Equivalence of Two-Point Basis-Set Extrapolations and Robust Parameterization for Coupled-Cluster and Double-Hybrid DFT Methods
Source: J Phys Chem A. 2026 May 27;130(23):4433–49. doi: 10.1021/acs.jpca.6c01541 (PMC13267079; doi:10.1021/acs.jpca.6c01541)
Supplement: Supplementary file 1 [file jp6c01541_si_001.pdf]

On the Equivalence of Two-Point Basis-Set Extrapolations and Robust Parameterization for Coupled Cluster and Double-Hybrid DFT Methods

*Mark A. Iron**

Computational Chemistry Unit, Department of Chemical Research Support,

Weizmann Institute of Science, Rehovot 7610001, Israel.

Supporting Information

Table of Contents

S1 Discussion on the Omission of def2-TZVP and def2-TZVPD from this Study 3

S2 Geometries of the BSEF21 and BSEF74 Datasets 5

S3 Derivations of the Schwenke-type Equations for Each Extrapolation Method 15

S3.1 Exponential Extrapolations 15

S3.2 Exponential-Square Root Extrapolation 15

S3.3 Inverse Power Extrapolation 16

S3.4 Form of the Schwenke-type Extrapolation 17

S3.5 Derivations of the Interconversion Equations for α, β and γ and their *f* Functions 17

S4 Derivation of the Equation for the Exponential Extrapolation to the CBS Limit for Three Consecutive Basis Sets 19

S5 Further Discussion on the Extrapolations to the Complete Basis Set Limit 21

S6 Additional Tables and Figures 23

S7 The Corrected ANO-cc-pVTZ Basis Set 89

S7.1 MOLPRO Format 89

S7.2 GAUSSIAN Format 96

S7.3 ORCA Format 130

S8 References 166

S1 Discussion on the Omission of def2-TZVP and def2-TZVPD from this Study

There are five members of the def2 family, specifically def2-SVP, def2-TZVP, def2-TZVPP, def2-QZVP and def2-QZVPP, and their diffuse (def2D) couterparts. Initially, extraolations all consecutive pairs was considered, but this led to extrapolation issues with the MH_n ($n \geq 2$) molecules in the dataset (*vide infra*, Section S2), in particular with the correlation energies. For instance, the changes in CCSD(T) correlation energies with increasing basis sets are given in Table S1 for two well-behaved systems (N_2 and O_2, although most other non-hydrogen containing species behave in a similar fashion) and three pathelogical cases (H_2, AlH_3 and SiH_4). For the first two, the differences between conseutive def2-*n*ZVP basis sets decreases with increasing basis set sizes, meaning the energies converge to some values. However, for the latter three systems, the differences increase, meaning that the energies actually diverge, and one cannot use them for extrapolations to the CBS limit, especially the three-point exponential extrapolation. This divergent behaviour is not observed with the def2-*n*ZVPP basis sets. The difference between def-TZVP and def2-TZVPP (and the Qζ counterparts) is the addition of another set of polarization functions on hydrogen atoms (Table S2), while they are identical for the second-row elements. The def2-SVP–def2-TZVPP–def2-QZVPP progression has consistently increasing polarization functions, which is missing from the smaller basis sets. Therefore, only this set of larger basis sets is included in this study.

Table S1. Changes in CCSD(T) correlation energies (in Ha) between def2-TZVP/def2-TZVPP and def2-SVP $\left(\Delta E_{DZ\rightarrow TZ}^{CCSD(T)}\right)$ and between def2-QZVP/def2-QZVPP and def2-TZVP/def2-TZVPP $\left(\Delta E_{TZ\rightarrow QZ}^{CCSD(T)}\right)$ and the difference of differences $\left(\Delta\Delta E^{CCSD(T)}\right)$ for select molecules.

Molecule	def2-*n*ZVP			def2-*n*ZVPP		
	$\Delta E_{DZ\rightarrow TZ}^{CCSD(T)}$	$\Delta E_{TZ\rightarrow QZ}^{CCSD(T)}$	$\Delta\Delta E^{CCSD(T)}$	$\Delta E_{DZ\rightarrow TZ}^{CCSD(T)}$	$\Delta E_{TZ\rightarrow QZ}^{CCSD(T)}$	$\Delta\Delta E^{CCSD(T)}$
N_2	-0.0659	-0.0232	0.0427	-0.0659	-0.0232	0.0427
O_2	-0.0972	-0.0338	0.0634	-0.0972	-0.0338	0.0634
H_2	-0.013	-0.046	-0.0033	-00049	-0.0009	0.0040
AlH_3	-0.0109	-0.0128	-0.0019	-0.0190	-0.0047	0.0143
SiH_4	-0.0179	-0.0182	-0.0013	-0.0659	-0.0232	0.0427

Table S2. Set of polarization functions on hydrogen in the def2-family of basis sets.

Basis Set	Polarization Functions	Basis Set	Polarization Functions
def2-SVP	2s1p		
def2-TZVP	3s1p	def2-TZVPP	3s2p1d
def2-QZVP	4s3p2d1f	def2-QZVPP	4s3p2d1f

S2 Geometries of the BSEF21 and BSEF74 Datasets

Neese and Valeev reported geometries for the molecules in the BSEF21 test set.[S1] For the most part these were the geometries used in this study, with the following exceptions:

- The geometry for BH_3 appears to be misreported, specifically the reported bond distance of 1.1898 a.u. would be more appropriate if the units were Ångstroms.
- For BN, Neese and Valeev considered the "$^3\Sigma$" excited state but noted that this is an excited state; the geometry of the $^3\Pi$ ($^3B_1$ or $^3B_2$) ground state was optimized here at the same B3LYP/def2-TZVP level used by Neese and Valeev giving $R_{B\text{–}N}$ = 2.78367 a.u. (*c.f.* r_e = 1.281 Å = 2.421 a.u. in Huber and Herzberg[S2]). At the CCSD(T)/aug-cc-pV6Z level of theory (this study), the $^3B_1$ state (which is the same in energy as the $^3B_2$ state) is 20.3 kcal/mol lower in energy than the $^3A_2$ state (which would correspond to the $^3\Sigma^-$ state).
- For B_2, experiments indicate a $^3\Sigma_g^-$ ground state[S3] while earlier CI calculations predicted a $^5\Sigma_u^-$ ground state slightly below the $^3\Sigma_g^-$ state,[S4] although the CCSD(T)/aug-cc-pV6Z calculations in this study here indicate the triplet state is 3.55 kcal/mol more stable than the quintet, where the geometries of each state were optimized at the B3LYP/def2-TZVP level of theory following Neese and Valeev's protocol. The optimized geometry for the $^3\Sigma_g^-$ ($^3B_{1g}$) electronic state is $R_{B\text{–}B}$ = 3.04749 a.u. (*c.f.* r_e = 1.590 Å = 3.005 a.u. in Huber and Herzberg[S2]); this is the state used in this study. For the $^5\Sigma_u^-$ (^5A_u) state, the optimized $R_{B\text{–}B}$ = 2.86674 a.u., while the $^3\Pi_u$ state has $R_{B\text{–}B}$ = 3.29247 a.u.
- For BC, B3LYP/def2-TZVP geometry optimization gives $R_{B\text{–}C}$ = 2.80259 a.u. compared to Neese and Valeev's $R_{B\text{–}C}$ = 2.554910 a.u.

- For CN, B3LYP/def2-TZVP geometry optimization gives $R_{C\text{–}N}$ = 2.19682 a.u. compared to Neese and Valeev's $R_{C\text{–}N}$ = 2.319042 a.u. (*c.f.* r_e = 1.1718 Å = 2.2144 a.u. in Huber and Herzberg[S2]).

The new geometries that extend the BSEF21 set to BSEF74 were likewise optimized at the B3LYP/def2-TZVP level of theory following Neese and Valeev.[S1] The final geometric parameters are given in Table S3.

Table S3. Geometric parameters used of all molecules in the BSEF74 Fitting Set (the first twenty-one up to the horizontal line correspond to the BSEF21 set).

Molecule	Spin state	r (Å)	∡ (°)
B_2	$^3\Sigma_g^-$ ($^3B_{1g}$)	1.612663	
BC	$^4\Sigma^-$ ($^4A_2$)	1.483067	
BF	$^1\Sigma^+$ ($^1A_1$)	1.265104	
BH_3	$^1A_1'$ ($^1A_1$)	1.189800	120.0000 (Cartesian coordinates below)
BN	$^3\Pi$ ($^3B_2$ or $^3B_1$)	1.320706	
BO	$^2\Sigma^+$ ($^2A_1$)	1.202366	
C_2	$^1\Sigma_g^+$ (^1A_g)	1.247400	
CF	$^2\Pi$ ($^2B_2$ or $^2B_1$)	1.275788	
CH_4	$^1A_1$ ($^1A'$)	1.089565	(Cartesian coordinates below)
CN	$^2\Sigma^+$ ($^2A_1$)	1.162507	
CO	$^1\Sigma^+$ ($^1A_1$)	1.125076	
F_2	$^1\Sigma_g^+$ (^1A_g)	1.396860	
HF	$^1\Sigma^+$ ($^1A_1$)	0.924724	
H_2	$^1\Sigma_g^+$ (^1A_g)	0.744246	
H_2O	$^1A_1$ ($^1A_1$)	0.962970	105.2370 (Cartesian coordinates below)
N_2	$^1\Sigma_g^+$ (^1A_g)	1.091084	
NF	$^3\Sigma^-$ ($^3A_2$)	1.318248	
NH_3	$^1A_1$ ($^1A_1$)	1.013898	(Cartesian coordinates below)

Molecule	Spin state	r (Å)	∡ (°)
NO	$^2\Pi$ ($^2B_2$ or $^2B_1$)	1.145144	
O_2	$^3\Sigma_g^-$ ($^3B_{1g}$)	1.204554	
OF	$^2\Pi$ ($^2B_2$ or $^2B_1$)	1.349436	
Al_2	$^3\Sigma_g^-$ ($^3B_{1g}$)	2.502784	
AlB	$^3\Sigma^-$ ($^3A_2$)	2.044779	
AlCl	$^1\Sigma^+$ ($^1A_1$)	2.160425	
AlC	$^4\Sigma^-$ ($^4A_2$)	1.970810	
AlF	$^1\Sigma^+$ ($^1A_1$)	1.671555	
AlH_3	$^1A_1'$ ($^1A_1$)	1.580309	120.0000 (Cartesian coordinates below)
AlH	$^1\Sigma^+$ ($^1A_1$)	1.661431	
AlN	$^3\Pi$ ($^3B_2$ or $^3B_1$)	1.792438	
AlO	$^2\Sigma^+$ ($^2A_1$)	1.627148	
AlP	$^3\Pi$ ($^3B_2$ or $^3B_1$)	2.223280	
AlSi	$^4\Sigma^-$ ($^4A_2$)	2.431500	
AlS	$^2\Sigma^+$ ($^2A_1$)	2.044966	
BCl	$^1\Sigma^+$ ($^1A_1$)	1.721523	
BH	$^1\Sigma^+$ ($^1A_1$)	1.232957	
CCl	$^2\Pi$ ($^2B_2$ or $^2B_1$)	1.275788	
CH	$^2\Pi$ ($^2B_2$ or $^2B_1$)	1.275788	
Cl_2	$^1\Sigma_g^+$ (^1A_g)	2.013632	
ClF	$^1\Sigma^+$ ($^1A_1$)	1.640522	
ClN	$^3\Sigma^-$ ($^3A_2$)	1.619789	
ClO	$^2\Pi$ ($^2B_2$ or $^2B_1$)	1.275788	
H_2S	$^1A_1$	1.342597	92.5570 (Cartesian coordinates below)
HCl	$^1\Sigma^+$ ($^1A_1$)	1.281908	
NH	$^3\Sigma^-$ ($^3A_2$)	1.041776	
OH	$^2\Pi$ ($^2B_2$ or $^2B_1$)	1.275788	
P_2	$^1\Sigma_g^+$ (^1A_g)	1.889538	

Molecule	Spin state	r (Å)	∡ (°)
PB	$^3\Pi$ ($^3B_2$ or $^3B_1$)	1.741103	
PCl	$^3\Sigma^-$ ($^3A_2$)	2.034806	
PC	$^2\Sigma^+$ ($^2A_1$)	1.552827	
PF	$^3\Sigma^-$ ($^3A_2$)	1.603654	
PH_3	$^1A_1$ ($^1A_1$)	1.419163	93.3798 (Cartesian coordinates below)
PH	$^3\Sigma^-$ ($^3A_2$)	1.428454	
PN	$^1\Sigma^+$ ($^1A_1$)	1.482568	
PO	$^2\Pi$ ($^2B_2$ or $^2B_1$)	1.275788	
PS	$^2\Pi$ ($^2B_2$ or $^2B_1$)	1.275788	
S_2	$^3\Sigma_g^-$ ($^3B_{1g}$)	1.903736	
SB	$^2\Sigma^+$ ($^2A_1$)	1.610582	
SCl	$^2\Pi$ ($^2B_2$ or $^2B_1$)	1.275788	
SC	$^1\Sigma^+$ ($^1A_1$)	1.531948	
SF	$^2\Pi$ ($^2B_2$ or $^2B_1$)	1.275788	
SH	$^2\Pi$ ($^2B_2$ or $^2B_1$)	1.275788	
Si_2	$^3\Sigma_g^-$ ($^3B_{1g}$)	2.268102	
SiB	$^4\Sigma^-$ ($^4A_2$)	1.912650	
SiCl	$^2\Pi$ ($^2B_2$ or $^2B_1$)	1.275788	
SiC	$^3\Pi$ ($^3B_2$ or $^3B_1$)	1.713771	
SiF	$^2\Pi$ ($^2B_2$ or $^2B_1$)	1.275788	
SiH_4	$^1A_1$ ($^1A'$)	1.480448	109.4712 (Cartesian coordinates below)
SiH	$^2\Pi$ ($^2B_2$ or $^2B_1$)	1.275788	
SiN	$^2\Sigma^+$ ($^2A_1$)	1.567466	
SiO	$^1\Sigma^+$ ($^1A_1$)	1.513135	
SiP	$^2\Sigma^+$ ($^2A_1$)	1.982109	
SiS	$^1\Sigma^+$ ($^1A_1$)	1.937883	
SN	$^2\Pi$ ($^2B_2$ or $^2B_1$)	1.275788	
SO	$^3\Sigma^-$ ($^3A_2$)	1.489687	

For convenience, here are the Cartesian coordinates (in Å) of selected molecules:

BH_3:

```
4
 Coordinates BH3
   B    -0.000000    -0.000000    0.000000
   H     0.000000     1.189800   -0.000000
   H    -1.030397    -0.594900   -0.000000
   H     1.030397    -0.594900   -0.000000
```

CH_4:

```
5
 Coordinates CH4
   C    0.000000    0.000000    0.000000
   H    0.000000    0.000000    1.089565
   H    0.000000   -1.027252   -0.363188
   H   -0.889626    0.513626   -0.363188
   H    0.889626    0.513626   -0.363188
```

NH_3:

```
4
 Coordinates NH3
   N    0.000000    0.000000    0.000000
   H    1.013898    0.000000    0.000000
   H   -0.301711    0.967967    0.000000
   H   -0.301711   -0.410106   -0.876797
```

H_2O:

```
3
 Coordinates H2O
   O    0.000000    0.000000   -0.058639
   H    0.000000   -0.765186    0.525999
   H    0.000000    0.765186    0.525999
```

AlH_3:

```
4
 Coordinates AlH3
   Al    0.000000    0.000000    0.000000
   H     0.000000    1.580309    0.000000
   H     1.368588   -0.790155    0.000000
   H    -1.368588   -0.790155   -0.000000
```

SiH_4:

```
5
 Coordinates SiH4
   Si    0.000000    0.000000    0.000000
   H     0.854737    0.854737    0.854737
   H    -0.854737   -0.854737    0.854737
   H    -0.854737    0.854737   -0.854737
   H     0.854737   -0.854737   -0.854737
```

PH_3:

```
4
 Coordinates PH3
   P     0.000000    0.000000    0.128256
   H     0.000000    1.192409   -0.641279
   H     1.032656   -0.596204   -0.641279
   H    -1.032656   -0.596204   -0.641279
```

H_2S:

```
3
 Coordinates H2S
   S    -0.000000    0.000000    0.103105
   H     0.000000    0.970305   -0.824836
   H    -0.000000   -0.970305   -0.824836
```

In some cases, two states were found with the same overall symmetry. In all cases, care was taken to make sure the lower energy state was used. To avoid confusion, the final α and β occupations are given in Table S4.

Table S4. The α and β orbital occupancies (only α is given for closed-shell systems) of the molecules in the BSEF74 dataset, divided by the largest nondegenerate Abelian symmetry of the molecule, and the number of electrons (n_{elec}), overall symmertry (symm) and number of unpaired electrons (m_s).

D_{2h}	n_{elec}	symm	m_s	α spin								β spin							
				a_g	b_{3u}	b_{2u}	b_{1g}	b_{1u}	b_{2g}	b_{3g}	a_u	a_g	b_{3u}	b_{2u}	b_{1g}	b_{1u}	b_{2g}	b_{3g}	a_u
Al_2	26	b_{1g}	2	4	2	2	0	4	1	1	0	4	1	1	0	4	1	1	0
B_2	10	b_{1g}	2	2	1	1	0	2	0	0	0	2	0	0	0	2	0	0	0
C_2	12	a_g	0	2	1	1	0	2	0	0	0								
Cl_2	34	a_g	0	5	2	2	0	4	2	2	0								
F_2	18	a_g	0	3	1	1	0	2	1	1	0								
H_2	2	a_g	0	1	0	0	0	0	0	0	0								
N_2	14	a_g	0	3	1	1	0	2	0	0	0								
O_2	16	b_{1g}	2	3	1	1	0	2	1	1	0	3	1	1	0	2	0	0	0
P_2	30	a_g	0	5	2	2	0	4	1	1	0								
S_2	32	b_{2g}	2	5	2	2	0	4	2	2	0	5	1	1	0	4	1	1	0
Si_2	28	b_{1g}	2	5	2	2	0	4	1	1	0	5	2	2	0	4	1	1	0
C_{2v}	n_{elec}	symm	m_s	a_1	b_1	b_2	a_2					a_1	b_1	b_2	a_2				
AlB	18	a_2	2	6	2	2	0					6	1	1	0				
AlC	19	a_2	3	7	2	2	0					6	1	1	0				
AlCl	30	a_1	0	9	3	3	0												
AlF	22	a_1	0	7	2	2	0												

AlH_3	16	a_1	0	5	2	1	0				
AlH	14	a_1	0	5	1	1	0				
AlN	20	b_1	2	7	2	2	0	6	1	2	0
AlO	21	a_1	1	7	2	2	0	6	2	2	0
AlP	28	b_1	2	9	3	3	0	8	2	3	0
AlSi	27	a_2	3	9	3	3	0	8	2	2	0
AlS	29	a_1	1	9	3	3	0	8	3	3	0
BC	11	a_2	3	5	1	1	0	4	0	0	0
BCl	22	a_1	0	7	2	2	0				
BF	14	a_1	0	5	1	1	0				
BH_3	8	a_1	0	3	1	0	0				
BH	6	a_1	0	3	0	0	0				
BN	12	b_1	2	5	1	1	0	4	0	1	0
BO	13	a_1	1	5	1	1	0	4	1	1	0
CCl	23	b_1	1	7	3	2	0	7	2	2	0
CF	15	b_1	1	5	2	1	0	5	1	1	0
CH	7	b_1	1	3	1	0	0	3	0	0	0
CN	13	a_1	1	5	1	1	0	4	1	1	0
CO	14	a_1	0	5	1	1	0				
ClF	26	a_1	0	7	3	3	0				
ClN	24	a_2	2	7	3	3	0	7	2	2	0
ClO	25	b_1	1	7	3	3	0	7	2	3	0
H_2O	10	a_1	0	3	1	1	0				
H_2S	18	a_1	0	5	2	2	0				
HCl	18	a_1	0	5	2	2	0				

HF	10	a_1	0	3	1	1	0				
NF	16	a_2	2	5	2	2	0	5	1	1	0
NH	8	a_2	2	3	1	1	0	3	0	0	0
NO	15	b_1	1	5	2	1	0	5	1	1	0
OF	17	b_1	1	5	2	2	0	5	1	2	0
OH	9	b_1	1	3	1	1	0	3	0	1	0
PB	20	b_1	2	7	2	2	0	6	1	2	0
PCl	32	a_2	2	9	4	4	0	9	3	3	0
PC	21	a_1	1	7	2	2	0	6	2	2	0
PF	24	a_2	2	7	3	3	0	7	2	2	0
PH	16	a_2	2	5	2	2	0	5	1	1	0
PN	22	a_1	0	7	2	2	0				
PO	23	b_1	1	7	3	2	0	7	2	2	0
PS	31	b_1	1	9	4	3	0	9	3	3	0
SB	21	a_1	1	7	2	2	0	6	2	2	0
SC	22	a_1	0	7	2	2	0				
SCl	33	b_1	1	9	4	4	0	9	3	4	0
SF	25	b_1	1	7	3	3	0	7	2	3	0
SH	17	b_1	1	5	2	2	0	5	1	2	0
SiB	19	a_2	3	7	2	2	0	6	1	1	0
SiCl	31	b_1	1	9	4	3	0	9	3	3	0
SiC	20	b_1	2	7	2	2	0	6	1	2	0
SiF	23	b_1	1	7	3	2	0	7	2	2	0
SiH	15	b_1	1	5	2	1	0	5	1	1	0
SiO	22	a_1	0	7	2	2	0				

SiN	21	a_1	1	7	2	2	0	6	2	2	0
SiP	29	a_1	1	9	3	3	0	8	3	3	0
SN	23	b_1	1	7	3	2	0	7	2	2	0
SO	24	a_2	2	7	3	3	0	7	2	2	0
SiS	30	a_1	0	9	3	3	0				
**D_2**	**n_{elec}**	**symm**	**m_s**	**a_1**	**b_3**	**b_2**	**b_1**	**a_1**	**b_3**	**b_2**	**b_1**
CH_4	10	a_1	0	4	1						
SiH_4	18	a_1	0	3	2	2	2				
**C_s**	**n_{elec}**	**symm**	**m_s**	**a’**	**a”**			**a’**	**a”**		
NH_3	10	a’	0	4	1						
PH_3	18	a’	0	7	2						

S3 Derivations of the Schwenke-type Equations for Each Extrapolation Method

S3.1 Exponential Extrapolations

This extrapolation is of the type:

$$E_n = E_{CSB} + C \cdot \exp\,(-\gamma \cdot n) \tag{S1}$$

With two consecutive basis sets (V*x*Z and V*y*Z, *i.e.*, $y = x + 1$ – for ease of notation, we will switch here from the (n_1, n_2) used in the main part of this paper), one thus has two equations:

$$E_x = E_{CSB} + C \cdot \exp\,(-\gamma \cdot x) \tag{S1a}$$

$$E_y = E_{CSB} + C \cdot \exp\,(-\gamma \cdot y) \tag{S1b}$$

Subtracting these two equations, we get:

$$E_y - E_x = C \cdot (\exp(-\gamma \cdot y) - \exp(-\gamma \cdot x))$$

$$C = \frac{E_y - E_x}{\exp(-\gamma \cdot y) - \exp(-\gamma \cdot x)} \tag{S2}$$

Taking Eq. S1b, isolating E_{CBS} substituting for *C*:

$$E_{CBS} = E_y - \frac{E_y - E_x}{\exp(-\gamma \cdot y) - \exp(-\gamma \cdot x)} \cdot \exp(-\gamma \cdot y)$$

$$E_{CBS} = E_y + \frac{\exp(-\gamma \cdot y)}{\exp(-\gamma \cdot x) - \exp(-\gamma \cdot y)} \cdot \left(E_y - E_x\right) = E_y + f_\gamma \cdot \left(E_y - E_x\right)$$

This is almost the expression for f_β in the main text. Removing the minus sign from the exponentials gives:

$$f_\gamma = \frac{\frac{1}{\exp(\gamma \cdot y)}}{\frac{1}{\exp(\gamma \cdot x)} - \frac{1}{\exp(\gamma \cdot y)}}$$

$$f_\gamma = \frac{\frac{1}{\exp(\gamma \cdot y)}}{\frac{\exp(\gamma \cdot y) - \exp(\gamma \cdot x)}{\exp(\gamma \cdot x) \cdot \exp(\gamma \cdot y)}} = \frac{\exp(\gamma \cdot x)}{\exp(\gamma \cdot y) - \exp(\gamma \cdot x)} \tag{S3}$$

S3.2 Exponential-Square Root Extrapolation

The derivation for f_α is the same for f_γ in the previous section with the replacement of *C* with *A* and $\gamma \cdot x$ with $\alpha\sqrt{x}$ (and likewise with y). As noted in the main text, f_α often appears in a slightly different form:

$$f_\alpha = \frac{1}{\exp\left(\alpha\sqrt{y} - \alpha\sqrt{x}\right) - 1} \tag{S4}$$

If we define $\varepsilon_i = \exp(\alpha\sqrt{i})$ and note that $\exp(A - B) = \frac{\exp(A)}{\exp(B)}$, we get:

$$f_\alpha = \frac{1}{\frac{\exp(\alpha\sqrt{y})}{\exp(\alpha\sqrt{x})}-1} = \frac{1}{\frac{\varepsilon_y}{\varepsilon_x}-1} \cdot \left(\frac{\varepsilon_x}{\varepsilon_x}\right) = \frac{\varepsilon_x}{\frac{\varepsilon_y}{\varepsilon_x}\varepsilon_x - \varepsilon_x} = \frac{\varepsilon_x}{\varepsilon_y - \varepsilon_x} = \frac{\exp(\alpha\sqrt{x})}{\exp(\alpha\sqrt{y}) - \exp(\alpha\sqrt{x})} \quad \text{(S5)}$$

which is the form in the main text.

S3.3 Inverse Power Extrapolation

This is of the form:

$$E_n = E_{CBS} + B \cdot n^{-\beta} \quad \text{(S6)}$$

Thus, with two basis sets we have two equations:

$$E_x = E_{CBS} + B \cdot x^{-\beta} \quad \text{(S6a)}$$

$$E_y = E_{CBS} + B \cdot y^{-\beta} \quad \text{(S6b)}$$

Again, subtracting gives us:

$$E_y - E_x = B \cdot \left(y^{-\beta} - x^{-\beta}\right)$$

$$B = \frac{E_y - E_x}{y^{-\beta} - x^{-\beta}}$$

And back into Eq. S6a:

$$E_{CBS} = E_y - \frac{E_y - E_x}{y^{-\beta} - x^{-\beta}} \cdot y^{-\beta} = E_y + \frac{y^{-\beta}}{x^{-\beta} - y^{-\beta}} \cdot \left(E_y - E_x\right)$$

Or:

$$f_\beta = \frac{y^{-\beta}}{x^{-\beta} - y^{-\beta}} \quad \text{(S7a)}$$

To get the form with a positive exponent:

$$f_\beta = \frac{y^{-\beta}}{\frac{1}{x^\beta} - \frac{1}{y^\beta}} = \frac{y^{-\beta}}{\frac{y^\beta - x^\beta}{x^\beta y^\beta}} = \frac{y^{-\beta} x^\beta y^\beta}{x^\beta - y^\beta} = \frac{x^\beta}{y^\beta - x^\beta} \quad \text{(S7b)}$$

Similarly,

$$f_\beta = \frac{x^\beta}{y^\beta - x^\beta} \cdot \frac{x^{-\beta}}{x^{-\beta}} = \frac{1}{\left(\frac{y}{x}\right)^\beta - 1} \quad \text{(S7c)}$$

S3.4 Form of the Schwenke-type Extrapolation

In his original paper, Schwenke proposed using the scaled difference of two consecutive basis sets as a correction for the energy of the small basis set for the CBS limit (see Eq. 8 in Schwenke's paper),[S5] that is:

$$E_{CBS} \approx E_x + f \cdot \left(E_y - E_x\right) \tag{S8}$$

However, E_y should be closer to the CBS limit, so it would be more logical to use a correction for E_y rather than E_x since the former's correction should be smaller than the latter's. If, for instance in the derivation of the Schwenke form of the exponential extrapolation (Section S3.1), one were to substitute into Eq. S1a rather than Eq. S1b, one would receive an equation in this form (albeit with a slightly different f_Y).

S3.5 Derivations of the Interconversion Equations for α, β and γ and their *f* Functions

These equations (Eq. 12-14 in the main text) were initially derived using MAPLE 2024, and the derivations here were then reverse engineered from the known answers.

Given Eq. S4, one can flip it to get:

$$\exp\left(\alpha \cdot (\sqrt{n_2} - \sqrt{n_1})\right) - 1 = \frac{1}{f}$$

$$\exp\left(\alpha \cdot (\sqrt{n_2} - \sqrt{n_1})\right) = \frac{1}{f} + 1 = \frac{f+1}{f}$$

$$\alpha \cdot (\sqrt{n_2} - \sqrt{n_1}) = \ln\left(\frac{f+1}{f}\right)$$

$$\alpha = \frac{\ln\left(\frac{f+1}{f}\right)}{\sqrt{n_2} - \sqrt{n_1}} \tag{S9}$$

One can find the analogous conversion for β by manipulating it algebraically to a similar format:

$$f = \frac{n_1^{\beta}}{n_2^{\beta} - n_1^{\beta}} \cdot \frac{n_1^{-\beta}}{n_1^{-\beta}} = \frac{1}{n_2^{\beta} n_1^{-\beta} - 1}$$

As before flipping gives:

$$n_2^{\beta} n_1^{-\beta} = \left(\frac{n_2}{n_1}\right)^{\beta} = \frac{f+1}{f}$$

$$ln\left(\frac{n_2}{n_1}\right)^{\beta} = \ln\left(\frac{f+1}{f}\right)$$

$$\beta = \frac{\ln\left(\frac{f+1}{f}\right)}{\ln\left(\frac{n_2}{n_1}\right)} = \frac{\ln\left(\frac{f+1}{f}\right)}{\ln(n_2)-\ln(n_1)} \qquad \text{(S10)}$$

Similarly for γ:

$$f = \frac{\exp(\gamma \cdot n_1)}{\exp(\gamma \cdot n_2)-\exp(\gamma \cdot n_1)} \cdot \frac{\exp(-\gamma \cdot n_1)}{\exp(-\gamma \cdot n_1)} = \frac{1}{\exp(-\cdot n_2)\exp(-\gamma \cdot n_1)-1}$$

$$\frac{\exp(\gamma \cdot n_2)}{\exp(\gamma \cdot n_1)} = \frac{f+1}{f}$$

$$\ln(exp(\gamma \cdot n_2)) - \ln(exp(\gamma \cdot n_1)) = \gamma(n_2 - n_1) = \ln\left(\frac{f+1}{f}\right)$$

However, since we are working with consecutive basis sets, $n_2 = n_1 + 1 \Rightarrow n_2 - n_1 = 1$. Therefore,

$$\gamma = \ln\left(\frac{f+1}{f}\right) \qquad \text{(S11)}$$

The equations that interconvert between α, β and γ have in common $\ln\left(\frac{f+1}{f}\right)$ terms. Isolating this component in Eqs. S9 and S10 and equating gives:

$$\alpha(\sqrt{n_2} - \sqrt{n_1}) = \beta(\ln(n_2) - \ln(n_1)) = \ln\left(\left(\frac{n_2}{n_1}\right)^{\beta}\right) = \gamma \qquad \text{(S12)}$$

Thus one trivially arrives from here to the equations in the main paper (Eqs. 15-20).

S4 Derivation of the Equation for the Exponential Extrapolation to the CBS Limit for Three Consecutive Basis Sets

While these equations have been long reported, I was not able to find a full derivation, only the equations. Thus, for the curious, I am including my derivations here. The exponential extrapolation, as noted in the main text is:

$$E_n = E_{CBS} + C \cdot \exp(-\gamma \cdot L) \tag{S13}$$

For three sequential basis sets (VxZ, VyZ and VzZ, *i.e.*, $z = x + 2,\ y = x + 1$) one has the following three equations:

$$E_x = E_{CBS} + C \cdot \exp(-\gamma \cdot x) \tag{S14}$$

$$E_y = E_{CBS} + C \cdot \exp(-\gamma \cdot y) \tag{S15}$$

$$E_z = E_{CBS} + C \cdot \exp(-\alpha\gamma \cdot z) \tag{S16}$$

Combining the first two and defining $\theta = \exp(\gamma)$, one gets:

$$E_x - C\theta^{-x} = E_y - C\theta^{-y}$$

$$C = \frac{E_x - E_y}{\theta^{-x} - \theta^{-y}} \tag{S17}$$

Taking likewise the last two equations above (*i.e.*, S15 and S16):

$$E_y - E_z = C(\theta^{-y} - \theta^{-z})$$

Inserting the value of *C* and rearranging gives:

$$\frac{E_y - E_z}{E_x - E_y} = \frac{\theta^{-y} - \theta^{-z}}{\theta^{-x} - \theta^{-y}}$$

Taking the reciprocal of both sides and noting the relation between *x*, *y* and *z*:

$$\frac{E_x - E_y}{E_y - E_z} = \frac{\theta^{-x} - \theta^{-(x+1)}}{\theta^{-(x+1)} - \theta^{-(x+2)}}$$

$$\frac{E_x - E_y}{E_y - E_z} = \frac{\theta^{-x} - \theta^{-(x+1)}}{\theta^{-(x+1)} - \theta^{-(x+2)}} \cdot \frac{\theta^{x+1}}{\theta^{x+1}} = \frac{\theta^{-x+x+1} - \theta^{-x-1+x+1}}{\theta^{-x-1+x+1} - \theta^{-x-2+x+1}} = \frac{\theta - 1}{1 - \theta^{-1}}$$

Reverting θ gives:

$$\frac{E_x - E_y}{E_y - E_z} = \frac{\exp(\gamma) - 1}{1 - \exp(-\gamma)} \cdot \frac{\exp(\gamma)}{\exp(\gamma)} = \frac{\exp(\gamma)(\exp(\gamma) - 1)}{\exp(\gamma) - \exp(-\gamma) \cdot \exp(\gamma)} = \frac{\exp(\gamma)(\exp(\gamma) - 1)}{\exp(\gamma) - 1} = \exp(\gamma)$$

Thus,

$$\frac{E_x - E_y}{E_y - E_z} = \exp(\gamma) = \theta \tag{S18}$$

Or:

$$\gamma = \ln\left(\frac{E_x - E_y}{E_y - E_z}\right) \tag{S19}$$

Now if we go back to our equation for *C* (Eq. S17):

$$C = \frac{E_x - E_y}{\left(\frac{E_y - E_z}{E_x - E_y}\right)^x - \left(\frac{E_y - E_z}{E_x - E_y}\right)^{x+1}}$$

If we start with $x = 2$, then:

$$C = \frac{E_x - E_y}{\frac{(E_y - E_z)^2}{(E_x - E_y)^2} - \frac{(E_y - E_z)^3}{(E_x - E_y)^3}} = \frac{(E_x - E_y)\cdot(E_x - E_y)^3}{(E_y - E_z)^2(E_x - E_y) - (E_y - E_z)^3} = \frac{(E_x - E_y)^4}{(E_y - E_z)^2\cdot(E_x - E_y - E_y + E_z)}$$

$$C = \frac{(E_x - E_y)^4}{(E_y - E_z)^2(E_x - 2E_y + E_z)} \tag{S20}$$

Repeating for $x = 3$, one will get:

$$C = \frac{(E_x - E_y)^5}{(E_y - E_z)^3(E_x - 2E_y + E_z)} \tag{S21}$$

Following this pattern for any general *x*:

$$C = \frac{(E_x - E_y)^{x+2}}{(E_y - E_z)^x(E_x - 2E_y + E_z)} \tag{S22}$$

Finally, taking the equation for E_x:

$$E_{CBS} = E_x - C \cdot \exp(-\gamma \cdot x) = E_x - C \cdot \theta^{-x}$$

$$E_{CBS} = E_x - \left(\frac{(E_x - E_y)^{x+2}}{(E_y - E_z)^x(E_x - 2E_y + E_z)}\right)\left(\frac{E_x - E_y}{E_y - E_z}\right)^{-x} = E_x - \left(\frac{(E_x - E_y)^{x+2}(E_x - E_y)^{-x}}{(E_y - E_z)^x(E_y - E_z)^{-x}(E_x - 2E_y + E_z)}\right)$$

$$E_{CBS} = E_x - \frac{(E_x - E_y)^2}{E_x - 2E_y + E_z} = \frac{E_x^2 - 2E_xE_y + E_xE_z - E_x^2 + 2E_xE_y - E_y^2}{E_x - 2E_y + E_z}$$

$$E_{CBS} = \frac{E_xE_z - E_y^2}{E_x - 2E_y + E_z} \tag{S23}$$

Quod erat demonstrandum.

S5 Further Discussion on the Extrapolations to the Complete Basis Set Limit

Three extrapolation schemes are considered in this study: exponential–square root (Eq. 1), inverse power (Eq. 4) and exponential (Eq. 8). Each has three parameters: E_{CBS}, {α, β or γ} and {A, B, or C}. Thus, with the energies with three basis sets one, can build a set of three equations with three unknowns. As shown in Section S4, one can derive an equation for the exponential extrapolations for three consecutive basis sets (Eq. 21 in the main text or Eq. S23 here). However, the presence of the power or square root in the other two extrapolation schemes makes it impossible to find analogous analytical expressions for these two methods. One thus needs to use some fitting procedure.

There are three factors that affect the fitting that are considered here: which basis sets are included, the initial guess of the three parameters, and the function being minimized. With the Dunning basis sets used, there are three sets of basis sets one could consider: {D,T,Q,5,6}ζ, {T,Q,5,6}ζ or {Q,5,6}ζ. When fitting the extrapolation parameters, one thus could minimize either the MAD or RMDS between E_n obtained from the extrapolation equation (Eq. 1, 4 or 8) and from the explicit calculation.

Initially, the extrapolation parameters were fit using the SOLVER function in MICROSOFT EXCEL. The results of several set of fitting runs are given in Table S5, and there is a clear variability in the results and no clear indication which set is best. Just because a given set has a low MAD/RMSD does not mean that this is the best set, rather that this set is closest to the exponential {Q,5,6}ζ values. For this reason, as noted in the main text, it was chosen to use the {Q,5,6}ζ exponential extrapolation since this gives unambiguous results.

Table S5. Dependence of the extrapolation parameters α (for SCF energy) and β (for the correlation energy) on the fitting conditions: shown are the mean absolute deviation (MAD), mean signed deviation (MSD) and standard deviation (SD) in mHa of each parameterization relative to the {Q,5,6}ζ exponential extrapolation (Eq. 21) for various sets of included basis sets in the aug-cc-pVnZ family of basis sets and fitting conditions.[a]

	SCF			CCSD(T)			CCSD			(T)		
	MAD	MSD	SD	MAD	MSD	SD	MAD	MSD	SD	MAD	MSD	SD
ΔE(DTQ56) (1)	2.331	-2.331	2.030	15.627	-15.627	20.266	13.052	-13.052	17.159	0.453	-0.453	0.397
ΔE(DTQ56) (2)	1.856	-1.856	0.751	28.963	-28.963	24.494	24.559	-24.559	22.699	1.462	-1.462	1.047
ΔE(DTQ56) (3)	2.793	-2.793	2.280	2.809	0.115	1.985	2.770	2.210	1.745	0.250	0.250	0.109
ΔE(DTQ56) (4)	1.457	-1.456	0.800	4.831	-4.831	3.197	4.765	-4.765	3.190	0.141	-0.141	0.062
ΔE(DTQ56) (5)	0.977	-0.976	0.542	4.831	-4.831	3.196	4.765	-4.765	3.190	0.155	-0.123	0.068
ΔE(TQ56) (1)	1.420	-1.420	1.110	8.860	-8.858	8.993	8.216	-8.214	8.111	0.346	-0.346	0.184
ΔE(TQ56) (2)	1.714	-1.714	1.222	25.010	-25.010	15.315	24.320	-24.320	14.701	1.137	-1.137	0.485
ΔE(TQ56) (3)	1.693	-1.693	1.279	0.791	0.102	0.712	0.786	0.240	0.523	0.167	0.167	0.071
ΔE(TQ56) (4)	1.457	-1.456	0.800	4.831	-4.831	3.197	4.765	-4.765	3.190	0.141	-0.141	0.062
ΔE(TQ56) (5)	1.285	-1.284	1.219	4.832	-4.832	3.199	4.767	-4.767	3.191	0.155	-0.123	0.068
ΔE(Q56) (1)	1.293	-1.293	0.897	6.307	-6.306	5.032	5.151	-5.149	4.564	0.275	-0.275	0.153
ΔE(Q56) (2)	1.954	-1.954	2.188	14.555	-14.555	8.573	13.852	-13.852	8.665	0.682	-0.682	0.300
ΔE(Q56) (3)	1.351	-1.351	1.258	0.633	-0.625	0.601	0.533	-0.519	0.490	0.034	0.033	0.019
ΔE(Q56) (4)	1.351	-1.351	1.258	0.633	-0.625	0.601	0.533	-0.519	0.490	0.034	0.033	0.019

[a] The fittings used the basis sets with *n* as in the parentheses with the following starting conditions:

(1) initial attempt at fitting, minimizing MAD with initial E_{CBS} from some preliminary extrapolations, A = 4, B = 0.2 (CCSD(T) and CCSD) or 0.02 (for (T)), α = 4 and β = 5;
(2) minimizing MAD with initial E_{CBS}, A, B, α and β values taken from {Q,5,6}ζ exponential extrapolation;
(3) minimizing MAD with initial $E_{CBS} = E_{aug-cc-pV6Z}$, A = B = 4, α =β = 3;
(4) minimizing RMSD with initial E_{CBS}, A, B, α and β values taken from {Q,5,6}ζ exponential extrapolation;
(5) minimizing RMSD with initial $E_{CBS} = E_{aug-cc-pV6Z}$, A = B = 4, α =β = 3.

S6 Additional Tables and Figures

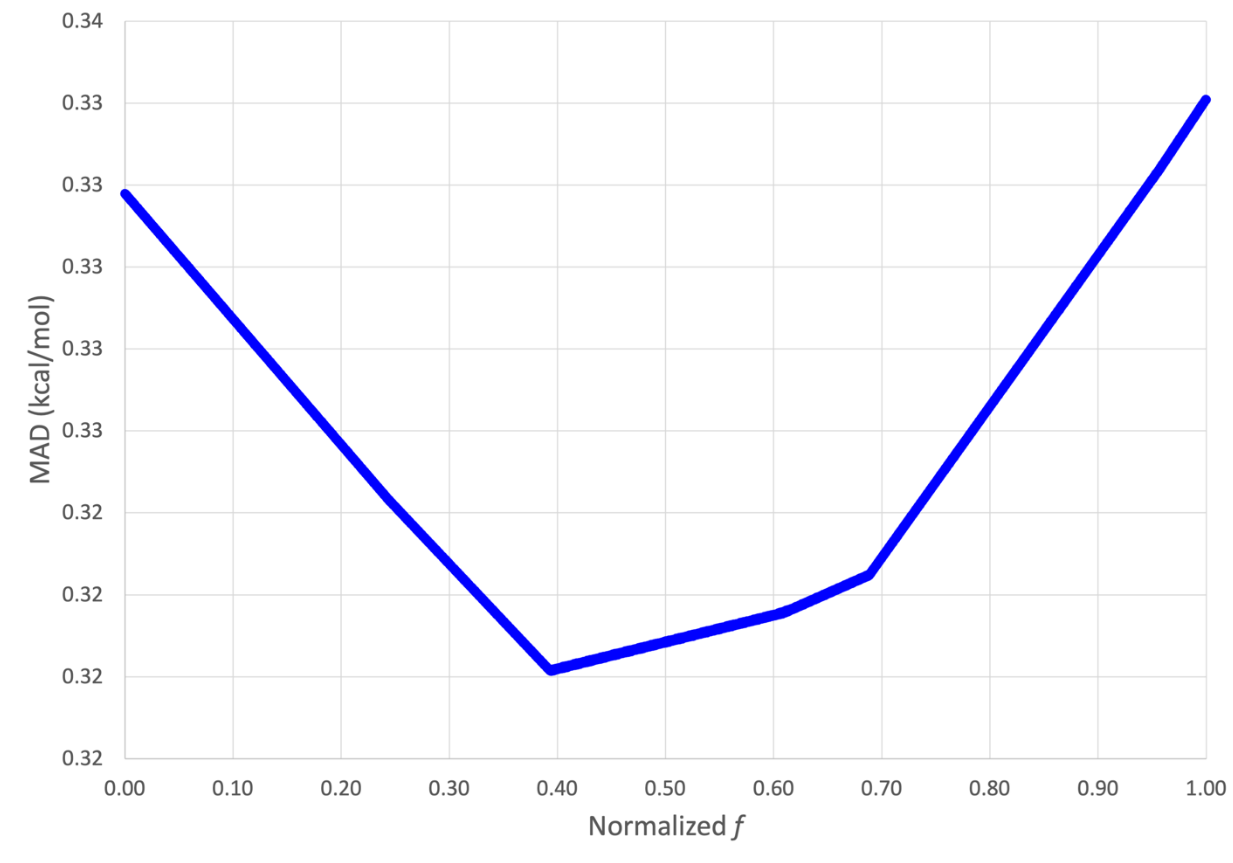

Figure S1. Plot of the MAD (kcal/mol) along the trace of the contour of the MAD surface as a function of f_{SCF} and $f_{CCSD(T)}$. f_{SCF} parameter was scanned in the range of 1.5-2.5 and the $f_{CCSD(T)}$ was found that corresponds to the minimum MAD for each f_{SCF}. The x-axis is the normalized distance along the resulting line (see Figure 3C in main text), that is $N = \frac{r_i}{r}$ where r is the total length of the line and r_i is the distance of point i from the starting point.

Table S6. Complete basis set-limit (CBS) energies (SCF, total correlations, CCSD correlation and (T) correlation, all in Ha) and the difference in calculated correlation energy (defined as $\Delta E_{CBS}^{corr} = E_{CBS}^{corr} - E_{CBS}^{CCSD} - E_{CBS}^{(T)}$, in mHa and cal/mol) for each new member of the BSEF74 dataset calculated using the three-point exponential extrapolation (Eq. 21) and the aug-cc-pVnZ (n = Q, 5, 6) basis sets.

**Molecule**	**Spin state**	E_{CBS}^{SCF}	E_{CBS}^{corr}	E_{CBS}^{CCSD}	$E_{CBS}^{(T)}$	ΔE_{CBS}^{corr}	
						**mHa**	**cal/mol**
Al_2	$^3\Sigma_g^-$ ($^3B_{1g}$)	-483.773052	-0.147832	-0.136677	-0.011155	0.0006	0.39
AlB	$^3\Sigma^-$ ($^3A_2$)	-266.438116	-0.168798	-0.156663	-0.012135	0.0001	0.04
AlCl	$^1\Sigma^+$ ($^1A_1$)	-701.518236	-0.317162	-0.302563	-0.014601	0.0029	1.85
AlC	$^4\Sigma^-$ ($^4A_2$)	-279.669147	-0.179796	-0.171397	-0.008399	0.0004	0.25
AlF	$^1\Sigma^+$ ($^1A_1$)	-341.487925	-0.373570	-0.361469	-0.012105	0.0049	3.10
AlH_3	$^1A_1'$ ($^1A_1$)	-243.649794	-0.125635	-0.123811	-0.001825	0.0005	0.29
AlH	$^1\Sigma^+$ ($^1A_1$)	-242.464589	-0.087559	-0.085895	-0.001664	0.0000	0.02
AlN	$^3\Pi$ ($^3B_2$ or $^3B_1$)	-296.298755	-0.258033	-0.245515	-0.012520	0.0017	1.08
AlO	$^2\Sigma^+$ ($^2A_1$)	-316.774693	-0.358852	-0.336659	-0.022198	0.0056	3.52
AlP	$^3\Pi$ ($^3B_2$ or $^3B_1$)	-582.617480	-0.230438	-0.215414	-0.015024	0.0001	0.04
AlSi	$^4\Sigma^-$ ($^4A_2$)	-530.798280	-0.169570	-0.158814	-0.010756	0.0003	0.17
AlS	$^2\Sigma^+$ ($^2A_1$)	-639.483240	-0.282253	-0.263642	-0.018613	0.0010	0.65
BCl	$^1\Sigma^+$ ($^1A_1$)	-484.162544	-0.340632	-0.323683	-0.016953	0.0035	2.19
BH	$^1\Sigma^+$ ($^1A_1$)	-25.131630	-0.105227	-0.102914	-0.002314	0.0003	0.20
CCl	$^2\Pi$ ($^2B_2$ or $^2B_1$)	-497.262316	-0.387386	-0.367381	-0.020009	0.0036	2.26
CH	$^2\Pi$ ($^2B_2$ or $^2B_1$)	-38.279814	-0.142844	-0.138799	-0.004045	0.0008	0.52
Cl_2	$^1\Sigma_g^+$ (^1A_g)	-919.010275	-0.496171	-0.472127	-0.024051	0.0074	4.64
ClF	$^1\Sigma^+$ ($^1A_1$)	-558.919364	-0.552514	-0.530386	-0.022134	0.0051	3.21
ClN	$^3\Sigma^-$ ($^3A_2$)	-513.906891	-0.431177	-0.410620	-0.020560	0.0031	1.95
ClO	$^2\Pi$ ($^2B_2$ or $^2B_1$)	-534.311548	-0.500456	-0.477834	-0.022626	0.0035	2.23
H_2S	$^1A_1$	-398.720151	-0.246588	-0.236415	-0.010175	0.0014	0.89
HCl	$^1\Sigma^+$ ($^1A_1$)	-460.112735	-0.264128	-0.253429	-0.010702	0.0030	1.91
NH	$^3\Sigma^-$ ($^3A_2$)	-54.978290	-0.182896	-0.177885	-0.005013	0.0017	1.06
OH	$^2\Pi$ ($^2B_2$ or $^2B_1$)	-75.422658	-0.251212	-0.244278	-0.006937	0.0028	1.76

P_2	$^1\Sigma_g^+$ (^1A_g)	-681.500602	-0.346393	-0.321463	-0.024931	0.0013	0.84
PB	$^3\Pi$ ($^3B_2$ or $^3B_1$)	-365.313807	-0.242133	-0.226347	-0.015787	0.0007	0.44
PCl	$^3\Sigma^-$ ($^3A_2$)	-800.273367	-0.387318	-0.367676	-0.019645	0.0032	2.02
PC	$^2\Sigma^+$ ($^2A_1$)	-378.486309	-0.332621	-0.309302	-0.023321	0.0014	0.86
PF	$^3\Sigma^-$ ($^3A_2$)	-440.233584	-0.437156	-0.420780	-0.016380	0.0039	2.44
PH_3	$^1A_1$ ($^1A_1$)	-342.494688	-0.221580	-0.213456	-0.008125	0.0015	0.92
PH	$^3\Sigma^-$ ($^3A_2$)	-341.295007	-0.153104	-0.147175	-0.005929	0.0003	0.16
PN	$^1\Sigma^+$ ($^1A_1$)	-395.189452	-0.402006	-0.375980	-0.026029	0.0028	1.78
PO	$^2\Pi$ ($^2B_2$ or $^2B_1$)	-415.631204	-0.430758	-0.408281	-0.022481	0.0048	3.00
PS	$^2\Pi$ ($^2B_2$ or $^2B_1$)	-738.293528	-0.377655	-0.353287	-0.024369	0.0014	0.87
S_2	$^3\Sigma_g^-$ ($^3B_{1g}$)	-795.093368	-0.420200	-0.394643	-0.025559	0.0020	1.28
SB	$^2\Sigma^+$ ($^2A_1$)	-422.192270	-0.296974	-0.278049	-0.018926	0.0008	0.51
SCl	$^2\Pi$ ($^2B_2$ or $^2B_1$)	-857.043636	-0.445267	-0.422641	-0.022630	0.0044	2.74
SC	$^1\Sigma^+$ ($^1A_1$)	-435.362588	-0.372864	-0.348498	-0.024368	0.0016	1.00
SF	$^2\Pi$ ($^2B_2$ or $^2B_1$)	-496.980146	-0.495704	-0.476183	-0.019525	0.0043	2.68
SH	$^2\Pi$ ($^2B_2$ or $^2B_1$)	-398.104985	-0.208898	-0.200541	-0.008359	0.0011	0.72
Si_2	$^3\Sigma_g^-$ ($^3B_{1g}$)	-577.780663	-0.219351	-0.205888	-0.013463	0.0001	0.07
SiB	$^4\Sigma^-$ ($^4A_2$)	-313.477679	-0.183669	-0.171977	-0.011692	0.0001	0.08
SiCl	$^2\Pi$ ($^2B_2$ or $^2B_1$)	-748.457112	-0.351784	-0.334420	-0.017368	0.0029	1.82
SiC	$^3\Pi$ ($^3B_2$ or $^3B_1$)	-326.630170	-0.259547	-0.243099	-0.016448	0.0007	0.43
SiF	$^2\Pi$ ($^2B_2$ or $^2B_1$)	-388.428476	-0.404203	-0.389943	-0.014264	0.0038	2.40
SiH_4	$^1A_1$ ($^1A'$)	-291.268645	-0.188413	-0.183919	-0.004496	0.0016	0.98
SiH	$^2\Pi$ ($^2B_2$ or $^2B_1$)	-289.437791	-0.119363	-0.115514	-0.003850	0.0000	0.03
SiN	$^2\Sigma^+$ ($^2A_1$)	-343.297630	-0.342030	-0.320325	-0.021708	0.0029	1.81
SiO	$^1\Sigma^+$ ($^1A_1$)	-363.855047	-0.393422	-0.372878	-0.020549	0.0058	3.67
SiP	$^2\Sigma^+$ ($^2A_1$)	-629.602422	-0.303123	-0.279281	-0.023843	0.0001	0.07
SiS	$^1\Sigma^+$ ($^1A_1$)	-686.516092	-0.331916	-0.310774	-0.021144	0.0012	0.72
SN	$^2\Pi$ ($^2B_2$ or $^2B_1$)	-451.949859	-0.434201	-0.408193	-0.026010	0.0020	1.27
SO	$^3\Sigma^-$ ($^3A_2$)	-472.403925	-0.474201	-0.449859	-0.024346	0.0036	2.27

Table S7. Schwenke *f* extrapolation parameters for two basis-set extrapolations and associated mean absolute and signed deviations (MAD and MSD, respectively, in kcal/mol) for Neese and Valeev's aug-ANO-V*n*Z and ANO-pV*n*Z basis sets for various WFT methods.

**Component**	***f***			**MAD**			**MSD**[a]		
	**{D,T}**	**{T,Q}**	**{Q,5}**	**{D,T}**	**{T,Q}**	**{Q,5}**	**{D,T}**	**{T,Q}**	**{Q,5}**
				aug-ANO-pV*n*Z					
SCF	0.3206	0.7130	1.7095	0.28	0.11	0.15	0.06	0.03	0.10
CCSD(T)	0.5023	0.6286	0.7709	0.95	0.17	0.21	-0.38	0.09	0.16
CCSD	0.5095	0.6234	1.0255	0.92	0.15	0.62	-0.39	0.02	0.26
(T)	0.4277	0.6314	1.2823	0.05	0.02	0.06	0.00	0.01	0.01
				ANO-pV*n*Z					
SCF	0.2853	0.8132	0.8825	0.39	0.32	0.12	0.12	0.07	0.04
CCSD(T)	0.5328	0.6169	0.7941	1.29	0.25	0.29	-0.64	-0.11	0.13
CCSD	0.5411	0.6248	0.8101	1.26	0.25	0.32	-0.50	-0.09	0.20
(T)	0.4152	0.5692	0.7518	0.11	0.05	0.03	0.01	0.01	0.02

[a] Defined as $E_{CBS}^{i} - E_{CBS}^{\{Q,5,6\}\zeta}$.

Table S8. Schwenke *f* extrapolation parameters for two basis-set extrapolations and and associated mean absolute and signed deviations (MAD and MSD, respectively, in kcal/mol) for the def2 basis sets for various WFT methods.

**Component**	***f***		**MAD**		**MSD**[a]	
	**{SVP, TZVPP}**	**{TZVPP, QZVPP}**	**{SVP, TZVPP}**	**{TZVPP, QZVPP}**	**{SVP, TZVPP}**	**{TZVPP, QZVPP}**
			with diffuse functions			
SCF	0.0702	0.0502	1.45	0.14	-0.37	0.07
CCSD(T)	5.5928	0.5604	15.09	7.79	13.44	8.27
CCSD	0.6019	0.6416	1.36	0.56	-0.16	-0.10
(T)	0.5377	0.5855	0.10	0.03	0.04	0.00
			without diffuse functions			
SCF	0.0708	0.0550	1.48	0.18	-0.32	0.10
CCSD(T)	4.9199	0.5406	16.23	7.36	14.02	8.50
CCSD	0.5027	0.6151	2.06	0.58	0.36	-0.10
(T)	0.4852	0.5464	0.12	0.03	0.03	0.00

[a] Defined as $E_{CBS}^{i} - E_{CBS}^{\{Q,5,6\}\zeta}$.

Table S9. Schwenke *f* extrapolation parameters for two basis-set extrapolations and and associated mean absolute and signed deviations (MAD and MSD, respectively, in kcal/mol) for Jensen's aug-pc-*n* and pc-*n* basis sets[a] for various WFT methods.

**Component**	***f***			**MAD**			**MSD**[b]		
	**{1,2}**	**{2,3}**	**{3,4}**	**{1,2}**	**{2,3}**	**{3,4}**	**{1,2}**	**{2,3}**	**{3,4}**
				aug-pc-*n*					
SCF	0.1999	0.8140	5.8134	3.72	2.17	2.20	1.76	0.21	0.28
CCSD(T)	0.7719	0.4056	0.6864	1.35	0.15	0.16	-0.48	-0.09	0.08
CCSD	0.7777	0.4738	0.7936	1.31	0.94	0.29	-0.49	0.03	0.04
(T)	0.7082	0.4527	0.7388	0.17	0.09	0.02	0.01	0.00	0.00
				pc-*n*					
SCF	0.2025	0.7175	5.3957	4.43	1.97	2.06	2.44	-0.10	0.08
CCSD(T)	0.8000	0.3839	0.6738	2.52	0.25	0.17	0.04	-0.16	0.08
CCSD	0.7941	0.4121	0.7590	2.15	0.80	0.25	0.11	0.17	0.02
(T)	0.7925	0.3883	0.6485	0.47	0.10	0.02	0.13	0.00	0.00

[a] For the pc-*n* basis sets, n = 1 corresponds to a double-ζ basis set, etc. [b] Defined as $E_{CBS}^{i} - E_{CBS}^{\{Q,5,6\}\zeta}$.

Table S10. Schwenke *f* extrapolation parameters for two basis-set extrapolations and corresponding α, β and γ parameters for wavefunction theory (WFT) methods with the various basis sets considered in this study.

**Component**	**CBS pair**	***f***	**α**	**β**	**γ**
		aug-cc-pV*n*Z			
SCF	{D,T}	0.3352	4.3485	3.4087	1.3821
	{T,Q}	0.2688	5.7921	5.3948	1.5520
	{Q,5}	0.1841	7.8847	8.3414	1.8613
	{5,6}	0.2562	7.4488	8.7194	1.5897
CCSD(T)	{D,T}	0.5183	3.3816	2.6508	1.0748
	{T,Q}	0.6029	3.6493	3.3990	0.9778
	{Q,5}	0.6794	3.8337	4.0558	0.9050
	{5,6}	0.6794	4.2405	4.9639	0.9050
CCSD	{D,T}	0.5271	3.3469	2.6236	1.0638
	{T,Q}	0.6045	3.6429	3.3931	0.9761
	{Q,5}	0.6918	3.7880	4.0074	0.8942
	{5,6}	0.6922	4.1886	4.9031	0.8939
(T)	{D,T}	0.4111	3.8802	3.0416	1.2333
	{T,Q}	0.6210	3.5808	3.3351	0.9595
	{Q,5}	0.5759	4.2643	4.5113	1.0067
	{5,6}	0.5830	4.6802	5.4786	0.9989

Component	CBS pair	*f*	α	β	γ
QCISD(T)	{D,T}	0.5169	3.3872	2.6552	1.0766
	{T,Q}	0.6029	3.6494	3.3991	0.9778
	{Q,5}	0.6788	3.8359	4.0581	0.9055
	{5,6}	0.6788	4.2429	4.9667	0.9055
QCISD	{D,T}	0.5266	3.3487	2.6250	1.0643
	{T,Q}	0.6042	3.6444	3.3944	0.9765
	{Q,5}	0.6897	3.7957	4.0155	0.8960
	{5,6}	0.6914	4.1916	4.9066	0.8946
(T) of QCISD(T)	{D,T}	0.4115	3.8780	3.0399	1.2326
	{T,Q}	0.6167	3.5969	3.3502	0.9638
	{Q,5}	0.5860	4.2175	4.4618	0.9956
	{5,6}	0.5883	4.6537	5.4476	0.9932
MP2	{D,T}	0.6738	2.8629	2.2442	0.9099
	{T,Q}	0.7461	3.1734	2.9557	0.8503
	{Q,5}	0.8163	3.3878	3.5840	0.7998
	{5,6}	0.8163	3.7473	4.3865	0.7998
SCF+MP2	{D,T}	0.5561	3.2373	2.5377	1.0289
	{T,Q}	0.6389	3.5157	3.2745	0.9420
	{Q,5}	0.7102	3.7227	3.9383	0.8788
	{5,6}	0.7183	4.0867	4.7838	0.8722

**Component**	**CBS pair**	***f***	**α**	**β**	**γ**
		cc-pV*n*Z			
SCF	{D,T}	0.3201	4.4577	3.4943	1.4168
	{T,Q}	0.2774	5.6992	5.3083	1.5271
	{Q,5}	0.2087	7.4408	7.8717	1.7565
	{5,6}	0.3086	6.7692	7.9239	1.4447
CCSD(T)	{D,T}	0.5322	3.3272	2.6081	1.0575
	{T,Q}	0.6165	3.5976	3.3508	0.9640
	{Q,5}	0.6956	3.7745	3.9932	0.8910
	{5,6}	0.7479	3.9777	4.6562	0.8489
CCSD	{D,T}	0.5418	3.2904	2.5793	1.0458
	{T,Q}	0.6208	3.5814	3.3357	0.9596
	{Q,5}	0.7132	3.7124	3.9275	0.8764
	{5,6}	0.7610	3.9313	4.6019	0.8390
(T)	{D,T}	0.4962	3.4725	2.7220	1.1037
	{T,Q}	0.5995	3.6624	3.4111	0.9813
	{Q,5}	0.6254	4.0459	4.2802	0.9551
	{5,6}	0.6958	4.1743	4.8863	0.8909
CCSD(T)	{D,T}	0.5315	3.3298	2.6102	1.0583
	{T,Q}	0.6164	3.5979	3.3511	0.9641
	{Q,5}	0.6953	3.7753	3.9940	0.8912

Component	CBS pair	f	α	β	γ
	{5,6}	0.7472	3.9800	4.6589	0.8494
CCSD	{D,T}	0.5400	3.2971	2.5846	1.0479
	{T,Q}	0.6197	3.5854	3.3395	0.9607
	{Q,5}	0.7125	3.7147	3.9298	0.8769
	{5,6}	0.7605	3.9329	4.6037	0.8394
(T)	{D,T}	0.4982	3.4639	2.7153	1.1010
	{T,Q}	0.6022	3.6520	3.4015	0.9785
	{Q,5}	0.6363	4.0009	4.2327	0.9445
	{5,6}	0.7016	4.1514	4.8595	0.8860
MP2	{D,T}	0.6797	2.8465	2.2313	0.9047
	{T,Q}	0.7692	3.1087	2.8954	0.8330
	{Q,5}	0.8548	3.2816	3.4717	0.7747
	{5,6}	0.9026	3.4939	4.0899	0.7457
SCF+MP2	{D,T}	0.5579	3.2310	2.5327	1.0269
	{T,Q}	0.6675	3.4170	3.1826	0.9156
	{Q,5}	0.7595	3.5587	3.7648	0.8401
	{5,6}	0.8358	3.6869	4.3159	0.7869
		aug-ANO-pV*n*Z			
SCF	{D,T}	0.3206	4.4537	3.4912	1.4156
	{T,Q}	0.7130	3.2712	3.0468	0.8765

Component	CBS pair	*f*	α	β	γ
	{Q,5}	1.7095	1.9510	2.0640	0.4606
CCSD(T)	{D,T}	0.5023	3.4468	2.7019	1.0955
	{T,Q}	0.6286	3.5527	3.3090	0.9519
	{Q,5}	0.7709	3.5230	3.7270	0.8317
CCSD	{D,T}	0.5095	3.4171	2.6786	1.0861
	{T,Q}	0.6234	3.5717	3.3267	0.9570
	{Q,5}	1.0255	2.8831	3.0501	0.6806
(T)	{D,T}	0.4277	3.7923	2.9727	1.2053
	{T,Q}	0.6314	3.5428	3.2998	0.9493
	{Q,5}	1.2823	2.4422	2.5837	0.5765
		ANO-pV*n*Z			
SCF	{D,T}	0.2853	4.7357	3.7122	1.5052
	{T,Q}	0.8132	2.9926	2.7874	0.8019
	{Q,5}	0.8825	3.2092	3.3951	0.7576
CCSD(T)	{D,T}	0.5328	3.3246	2.6061	1.0567
	{T,Q}	0.6169	3.5961	3.3494	0.9636
	{Q,5}	0.7941	3.4526	3.6526	0.8151
CCSD	{D,T}	0.5411	3.2929	2.5812	1.0466
	{T,Q}	0.6248	3.5667	3.3221	0.9557
	{Q,5}	0.8101	3.4057	3.6030	0.8040

Component	CBS pair	*f*	α	β	γ
(T)	{D,T}	0.4152	3.8584	3.0245	1.2263
	{T,Q}	0.5692	3.7848	3.5252	1.0141
	{Q,5}	0.7518	3.5835	3.7911	0.8460
	def2 with diffuse functions (def2D)				
SCF	{SVP,TZVPP}	0.0702	8.5720	6.7195	2.7245
	{TZVPP,QZVPP}	0.0502	11.3487	10.5702	3.0409
CCSD(T)	{SVP,TZVPP}	1.6977	1.4571	1.1422	0.4631
	{TZVPP,QZVPP}	0.5604	3.8216	3.5595	1.0240
CCSD	{SVP,TZVPP}	0.6019	3.0797	2.4141	0.9788
	{TZVPP,QZVPP}	0.6416	3.5061	3.2656	0.9394
(T)	{SVP,TZVPP}	0.5377	3.3061	2.5916	1.0508
	{TZVPP,QZVPP}	0.5855	3.7176	3.4626	0.9961
SCF	{SVP,TZVPP}	0.0702	8.5720	6.7195	2.7245
	{TZVPP,QZVPP}	0.0502	11.3487	10.5702	3.0409
CCSD(T)	{SVP,TZVPP}	1.6977	1.4571	1.1422	0.4631
	{TZVPP,QZVPP}	0.5604	3.8216	3.5595	1.0240
CCSD	{SVP,TZVPP}	0.6019	3.0797	2.4141	0.9788
	{TZVPP,QZVPP}	0.6416	3.5061	3.2656	0.9394
(T)	{SVP,TZVPP}	0.5377	3.3061	2.5916	1.0508
	{TZVPP,QZVPP}	0.5855	3.7176	3.4626	0.9961

Component	CBS pair	*f*	α	β	γ
def2 without diffuse functions					
SCF	{SVP,TZVPP}	0.0708	8.5468	6.6997	2.7165
	{TZVPP,QZVPP}	0.0550	11.0228	10.2667	2.9535
CCSD(T)	{SVP,TZVPP}	1.6458	1.4937	1.1709	0.4748
	{TZVPP,QZVPP}	0.5406	3.9084	3.6403	1.0472
CCSD	{SVP,TZVPP}	0.5027	3.4452	2.7007	1.0950
	{TZVPP,QZVPP}	0.6151	3.6026	3.3555	0.9653
(T)	{SVP,TZVPP}	0.4852	3.5198	2.7591	1.1187
	{TZVPP,QZVPP}	0.5464	3.8827	3.6164	1.0404
aug-pc-*n*					
SCF	{1,2}	0.1999	5.6393	4.4206	1.7924
	{2,3}	0.8140	2.9906	2.7855	0.8013
	{3,4}	5.8134	0.6724	0.7113	0.1587
CCSD(T)	{1,2}	0.7719	2.6144	2.0493	0.8309
	{2,3}	0.4056	4.6384	4.3203	1.2429
	{3,4}	0.6864	3.8079	4.0284	0.8989
CCSD	{1,2}	0.7777	2.6011	2.0389	0.8267
	{2,3}	0.4738	4.2349	3.9445	1.1347
	{3,4}	0.7936	3.4541	3.6541	0.8154
(T)	{1,2}	0.7082	2.7701	2.1715	0.8805

**Component**	**CBS pair**	***f***	**α**	**β**	**γ**
	{2,3}	0.4527	4.3515	4.0531	1.1660
	{3,4}	0.7388	3.6256	3.8356	0.8559
		pc-*n*			
SCF	{1,2}	0.2025	5.6044	4.3932	1.7813
	{2,3}	0.7175	3.2574	3.0340	0.8728
	{3,4}	5.3957	0.7202	0.7620	0.1700
CCSD(T)	{1,2}	0.8000	2.5515	2.0001	0.8110
	{2,3}	0.3839	4.7858	4.4575	1.2824
	{3,4}	0.6738	3.8546	4.0778	0.9099
CCSD	{1,2}	0.7941	2.5644	2.0102	0.8151
	{2,3}	0.4121	4.5965	4.2812	1.2316
	{3,4}	0.7590	3.5603	3.7665	0.8405
(T)	{1,2}	0.7925	2.5679	2.0129	0.8162
	{2,3}	0.3883	4.7549	4.4287	1.2741
	{3,4}	0.6485	3.9520	4.1809	0.9329

Table S11. Complete basis set-limit (CBS) correlation energies (total QCISD(T), QCCSD, (T) and MP2, all in Ha) and the difference in calculated correlation energy (defined as $\Delta E^{corr}_{CBS} = E^{corr}_{CBS} - E^{CCSD}_{CBS} - E^{(T)}_{CBS}$, in mHa and cal/mol) for each member of the BSEF74 calculated using the three-point exponential extrapolation (Eq. S23) and the aug-cc-pV*n*Z (*n* = Q, 5, 6) basis sets.

Molecule	Spin state	E^{corr}_{CBS}	E^{QCISD}_{CBS}	$E^{(T)}_{CBS}$		ΔE^{MP2}_{CBS}
				mHa	cal/mol	
Al_2	-0.148358	-0.137248	-0.011111	0.001	0.41	-0.120824
AlB	-0.170527	-0.158494	-0.012033	0.000	0.00	-0.132190
AlC	-0.180296	-0.171972	-0.008324	0.000	0.25	-0.146957
AlCl	-0.317230	-0.302740	-0.014493	0.003	1.84	-0.286687
AlF	-0.374019	-0.362470	-0.011553	0.004	2.75	-0.361552
AlH	-0.087576	-0.085940	-0.001636	0.000	0.00	-0.069136
AlH_3	-0.125649	-0.123847	-0.001803	0.000	0.27	-0.104680
AlN	-0.259911	-0.247974	-0.011939	0.003	1.60	-0.219980
AlO	-0.361247	-0.342231	-0.019018	0.002	1.46	-0.354560
AlP	-0.231230	-0.216153	-0.015077	0.000	0.04	-0.200156
AlS	-0.283065	-0.264713	-0.018353	0.001	0.39	-0.255747
AlSi	-0.169949	-0.159206	-0.010744	0.000	0.23	-0.139221
B_2	-0.217310	-0.199567	-0.017743	0.000	0.13	-0.177141
BC	-0.213984	-0.200980	-0.013005	0.001	0.60	-0.176463
BCl	-0.340892	-0.324194	-0.016701	0.003	1.96	-0.304589
BF	-0.389762	-0.377043	-0.012723	0.004	2.68	-0.372514
BH	-0.105249	-0.102975	-0.002274	0.000	0.18	-0.081519
BH_3	-0.145488	-0.142643	-0.002846	0.001	0.44	-0.125119
BN	-0.280661	-0.266764	-0.013898	0.002	1.09	-0.256059
BO	-0.352404	-0.336733	-0.015675	0.003	2.11	-0.342807
C_2	-0.404452	-0.371994	-0.032460	0.002	1.21	-0.384136
CCl	-0.388336	-0.368838	-0.019501	0.003	1.86	-0.348974
CF	-0.433783	-0.418706	-0.015081	0.004	2.51	-0.413319
CH	-0.143001	-0.138955	-0.004047	0.001	0.52	-0.119207

Molecule	Spin state	E_{CBS}^{corr}	E_{CBS}^{QCISD}	$E_{CBS}^{(T)}$ mHa	$E_{CBS}^{(T)}$ cal/mol	ΔE_{CBS}^{MP2}
CH_4	-0.239756	-0.232750	-0.007007	0.001	0.48	-0.218076
Cl_2	-0.496324	-0.472473	-0.023859	0.008	4.82	-0.454620
ClF	-0.553061	-0.531571	-0.021496	0.006	3.55	-0.526448
ClN	-0.432839	-0.412994	-0.019847	0.002	1.33	-0.391445
ClO	-0.501900	-0.480680	-0.021222	0.002	1.37	-0.460157
CN	-0.378416	-0.359847	-0.018570	0.001	0.83	-0.357229
CO	-0.413769	-0.395495	-0.018279	0.004	2.65	-0.400384
F_2	-0.619193	-0.597525	-0.021674	0.006	3.63	-0.604953
H_2	-0.040825	-0.040825				-0.034129
H_2O	-0.307041	-0.297437	-0.009607	0.003	1.85	-0.298706
H_2S	-0.246651	-0.236613	-0.010040	0.001	0.87	-0.219762
HCl	-0.264149	-0.253524	-0.010627	0.003	2.02	-0.240813
HF	-0.321672	-0.313133	-0.008543	0.003	1.95	-0.317548
N_2	-0.426117	-0.405916	-0.020205	0.004	2.27	-0.416220
NF	-0.477472	-0.461370	-0.016105	0.004	2.66	-0.456050
NH	-0.183081	-0.178076	-0.005007	0.002	1.08	-0.164313
NH_3	-0.277934	-0.268874	-0.009062	0.002	1.13	-0.262996
NO	-0.473212	-0.452201	-0.021016	0.005	3.16	-0.461937
O_2	-0.530699	-0.507416	-0.023287	0.005	3.06	-0.535394
OF	-0.550125	-0.531647	-0.018483	0.005	3.26	-0.526735
OH	-0.251400	-0.244577	-0.006826	0.003	1.71	-0.237032
P_2	-0.346788	-0.322193	-0.024596	0.001	0.77	-0.316957
PB	-0.242951	-0.227501	-0.015451	0.001	0.44	-0.209888
PC	-0.334228	-0.311706	-0.022522	0.001	0.78	-0.304713
PCl	-0.387835	-0.368352	-0.019485	0.003	1.77	-0.349992
PF	-0.437965	-0.422253	-0.015715	0.004	2.17	-0.417778
PH	-0.153297	-0.147314	-0.005983	0.000	0.13	-0.130267

Molecule	Spin state	E_{CBS}^{corr}	E_{CBS}^{QCISD}	$E_{CBS}^{(T)}$		ΔE_{CBS}^{MP2}
				mHa	cal/mol	
PH_3	-0.221681	-0.213727	-0.007955	0.001	0.84	-0.191741
PN	-0.402896	-0.377716	-0.025182	0.002	1.52	-0.388467
PO	-0.432088	-0.410765	-0.021326	0.003	2.09	-0.414854
PS	-0.360120	-0.336221	-0.023900	0.001	0.88	-0.343491
S_2	-0.420695	-0.394936	-0.025761	0.002	1.26	-0.392439
SB	-0.297849	-0.279501	-0.018349	0.001	0.47	-0.268863
SC	-0.373731	-0.350565	-0.023167	0.001	0.82	-0.343846
SCl	-0.445848	-0.423489	-0.022363	0.004	2.68	-0.403681
SF	-0.496610	-0.477894	-0.018720	0.004	2.58	-0.471321
SH	-0.209039	-0.200663	-0.008377	0.001	0.71	-0.183769
Si_2	-0.219551	-0.206104	-0.013447	0.000	0.07	-0.184734
SiB	-0.184181	-0.172522	-0.011659	0.000	0.08	-0.150812
SiC	-0.261210	-0.244997	-0.016214	0.001	0.39	-0.222667
SiCl	-0.352161	-0.334908	-0.017256	0.003	1.65	-0.316954
SiF	-0.404922	-0.391224	-0.013702	0.003	2.11	-0.387444
SiH	-0.119555	-0.115679	-0.003876	0.000	0.01	-0.097096
SiH_4	-0.188469	-0.184068	-0.004402	0.002	0.94	-0.159898
SiN	-0.344520	-0.324654	-0.019869	0.003	1.58	-0.311674
SiO	-0.394589	-0.375365	-0.019228	0.004	2.30	-0.382148
SiP	-0.304135	-0.280487	-0.023648	0.000	0.07	-0.276793
SiS	-0.332412	-0.311673	-0.020740	0.001	0.52	-0.300709
SN	-0.435327	-0.410093	-0.025236	0.002	1.13	-0.411256
SO	-0.475470	-0.451695	-0.023779	0.003	2.15	-0.460999

Table S12. Complete basis set-limit (CBS) energies for the DSD-PBEP86-D3BJ double-hybrid DFT functional components (Kohn–Sham energy, MP2-like component and total DH energy, all in Ha) and the difference in calculated total energy (defined as $\Delta E_{CBS} = E_{CBS}^{tot} - E_{CBS}^{KS} - E_{CBS}^{\mathrm{MP2}}$, in mHa and cal/mol) for each member of the BSEF74* calculated using the three-point exponential extrapolation (Eq. S23) and the aug-cc-pVnZ (n = Q, 5, 6) basis sets.

Molecule	E_{CBS}^{KS}	E_{CBS}^{MP2}	E_{CBS}^{tot}	$E_{CBS}^{KS} + E_{CBS}^{MP2}$	ΔE_{CBS}	
					mHa	cal/mol
Al_2	-484.184215	-0.111409	-484.295473	-484.295624	-0.150	-94.38
AlB	-266.709900	-0.103131	-266.812897	-266.813031	-0.134	-83.89
AlC	-702.005910	-0.147498	-702.153005	-702.153408	-0.403	-252.78
AlCl	-279.938410	-0.135738	-280.073961	-280.074148	-0.187	-117.58
AlF	-341.842582	-0.180817	-342.022892	-342.023399	-0.507	-318.18
AlH	-243.875333	-0.059684	-243.934938	-243.935017	-0.079	-49.73
AlH_3	-242.673002	-0.040324	-242.713255	-242.713326	-0.071	-44.58
AlN	-296.606894	-0.201471	-296.808094	-296.808365	-0.271	-170.21
AlO	-317.126806	-0.295948	-317.422360	-317.422754	-0.393	-246.69
AlP	-583.071918	-0.177874	-583.249557	-583.249792	-0.235	-147.36
AlS	-531.220671	-0.129171	-531.349668	-531.349842	-0.175	-109.68
AlSi	-639.957227	-0.233276	-640.190205	-640.190503	-0.298	-187.04
B_2	-49.214915	-0.170483	-49.385302	-49.385398	-0.096	-60.37
BC	-484.504476	-0.157354	-484.661532	-484.661830	-0.298	-187.07
BCl	-62.467293	-0.162933	-62.630054	-62.630226	-0.172	-108.15
BF	-124.375376	-0.186411	-124.561163	-124.561787	-0.624	-391.52
BH	-26.484748	-0.068611	-26.553251	-26.553359	-0.108	-67.76
BH_3	-25.190657	-0.046865	-25.237456	-25.237522	-0.066	-41.27
BN	-79.178674	-0.229705	-79.408115	-79.408378	-0.263	-165.13
BO	-99.754875	-0.304486	-100.058866	-100.059360	-0.494	-310.15
C_2	-75.595890	-0.208478	-75.804177	-75.804368	-0.190	-119.53
CCl	-497.632567	-0.314815	-497.946996	-497.947382	-0.386	-242.37
CF	-137.466950	-0.363709	-137.829841	-137.830659	-0.818	-513.53
CH	-40.343907	-0.113361	-40.457080	-40.457268	-0.188	-118.03

Molecule	E_{CBS}^{KS}	E_{CBS}^{MP2}	E_{CBS}^{tot}	$E_{CBS}^{KS} + E_{CBS}^{MP2}$	ΔE_{CBS}	
					mHa	cal/mol
CH_4	-38.362638	-0.110081	-38.472588	-38.472719	-0.132	-82.55
Cl_2	-919.585359	-0.230871	-919.815729	-919.816231	-0.501	-314.62
ClF	-559.361984	-0.263820	-559.624942	-559.625804	-0.862	-541.12
ClN	-514.303078	-0.343865	-514.646437	-514.646943	-0.506	-317.80
ClO	-534.735478	-0.413403	-535.148141	-535.148881	-0.740	-464.34
CN	-92.429970	-0.310093	-92.739739	-92.740063	-0.324	-203.38
CO	-113.008457	-0.202164	-113.210181	-113.210621	-0.440	-275.91
F_2	-199.089502	-0.300884	-199.389298	-199.390386	-1.088	-682.70
H_2	-100.232954	-0.155635	-100.388062	-100.388589	-0.527	-330.51
H_2O	-76.223227	-0.148686	-76.371591	-76.371913	-0.322	-201.93
H_2S	-1.150827	-0.019672	-1.170473	-1.170499	-0.026	-16.48
HCl	-399.006666	-0.114330	-399.120848	-399.120996	-0.149	-93.35
HF	-460.407470	-0.122460	-460.529693	-460.529930	-0.237	-148.84
N_2	-109.216441	-0.210432	-109.426517	-109.426873	-0.356	-223.56
NF	-154.101439	-0.393879	-154.494432	-154.495318	-0.886	-556.28
NH	-56.368679	-0.133167	-56.501634	-56.501846	-0.212	-133.16
NH_3	-55.084905	-0.139852	-55.224555	-55.224757	-0.203	-127.14
NO	-129.554057	-0.404647	-129.958099	-129.958704	-0.605	-379.86
O_2	-149.959478	-0.433854	-150.392545	-150.393332	-0.786	-493.38
OF	-174.493128	-0.464727	-174.956777	-174.957856	-1.079	-677.15
OH	-75.558701	-0.206606	-75.764926	-75.765307	-0.382	-239.42
P_2	-681.999578	-0.166198	-682.165603	-682.165777	-0.174	-108.93
PB	-365.616057	-0.189053	-365.804940	-365.805109	-0.169	-106.24
PC	-800.803820	-0.310149	-801.113504	-801.113969	-0.465	-291.55
PCl	-378.833046	-0.239392	-379.072257	-379.072437	-0.180	-113.23
PF	-440.628137	-0.363861	-440.991221	-440.991998	-0.777	-487.74
PH	-342.768725	-0.102786	-342.871404	-342.871512	-0.107	-67.40

Molecule	E_{CBS}^{KS}	E_{CBS}^{MP2}	E_{CBS}^{tot}	$E_{CBS}^{KS} + E_{CBS}^{MP2}$	ΔE_{CBS}	
					mHa	cal/mol
PH_3	-341.543732	-0.113869	-341.657461	-341.657601	-0.140	-87.64
PN	-395.554513	-0.200996	-395.755304	-395.755510	-0.206	-129.38
PO	-416.018180	-0.369841	-416.387549	-416.388022	-0.473	-296.83
PS	-738.815335	-0.308586	-739.123655	-739.123921	-0.266	-167.19
S_2	-795.643635	-0.333107	-795.976401	-795.976742	-0.340	-213.38
SB	-422.518165	-0.243883	-422.761835	-422.762048	-0.213	-133.56
SC	-857.599953	-0.362017	-857.961495	-857.961970	-0.475	-298.27
SCl	-435.717407	-0.178953	-435.896152	-435.896360	-0.208	-130.53
SF	-497.401432	-0.415714	-497.816295	-497.817147	-0.851	-534.20
SH	-398.378807	-0.164352	-398.542973	-398.543159	-0.187	-117.25
Si_2	-578.227508	-0.169936	-578.397267	-578.397444	-0.176	-110.73
SiB	-313.750806	-0.138277	-313.888966	-313.889083	-0.117	-73.61
SiC	-748.967243	-0.285341	-749.252164	-749.252583	-0.419	-262.80
SiCl	-326.942744	-0.184011	-327.126603	-327.126755	-0.152	-95.47
SiF	-388.803805	-0.341072	-389.144189	-389.144876	-0.687	-431.35
SiH	-291.525077	-0.088454	-291.613477	-291.613531	-0.054	-33.83
SiH_4	-289.666898	-0.091144	-289.757953	-289.758042	-0.089	-55.97
SiN	-343.642576	-0.273348	-343.915688	-343.915924	-0.237	-148.41
SiO	-364.217474	-0.195224	-364.412375	-364.412698	-0.323	-202.37
SiS	-687.010153	-0.156857	-687.166810	-687.167010	-0.200	-125.23
SN	-452.342219	-0.348636	-452.690534	-452.690855	-0.321	-201.62
SO	-472.821293	-0.388372	-473.209111	-473.209665	-0.555	-348.13

Table S13. Complete basis set-limit (CBS) energies for the B2PLYP double-hybrid DFT functional components (Kohn–Sham energy, MP2-like component and total DH energy, all in Ha) and the difference in calculated total energy (defined as $\Delta E_{CBS} = E_{CBS}^{tot} - E_{CBS}^{KS} - E_{CBS}^{\mathrm{MP2}}$, in mHa and cal/mol) for each member of the BSEF74* calculated using the three-point exponential extrapolation (Eq. S23) and the aug-cc-pVnZ (n = Q, 5, 6) basis sets.

Molecule	E_{CBS}^{KS}	E_{CBS}^{MP2}	E_{CBS}^{tot}	$E_{CBS}^{KS} + E_{CBS}^{MP2}$	ΔE_{CBS}	
					mHa	cal/mol
Al_2	-484.516728	-0.041720	-484.558291	-484.558448	-0.157	-98.55
AlB	-266.917116	-0.043059	-266.960064	-266.960174	-0.111	-69.51
AlC	-702.399533	-0.093751	-702.492743	-702.493284	-0.541	-339.77
AlCl	-280.159451	-0.051285	-280.210592	-280.210736	-0.144	-90.40
AlF	-342.135901	-0.115348	-342.250861	-342.251249	-0.388	-243.64
AlH	-244.057037	-0.034267	-244.091215	-244.091304	-0.089	-55.55
AlH_3	-242.847064	-0.023523	-242.870495	-242.870587	-0.091	-57.32
AlN	-296.854604	-0.075058	-296.929464	-296.929662	-0.198	-124.32
AlO	-317.403905	-0.112160	-317.515803	-317.516066	-0.263	-164.99
AlP	-583.431670	-0.065115	-583.496548	-583.496785	-0.237	-148.41
AlS	-531.561838	-0.047932	-531.609614	-531.609770	-0.156	-97.62
AlSi	-640.334756	-0.085238	-640.419645	-640.419994	-0.350	-219.42
B_2	-49.309986	-0.065457	-49.375386	-49.375443	-0.057	-35.76
BC	-484.776484	-0.100629	-484.876731	-484.877113	-0.382	-239.64
BCl	-62.570109	-0.063040	-62.633053	-62.633149	-0.097	-60.57
BF	-124.545458	-0.118986	-124.664037	-124.664443	-0.406	-254.90
BH	-26.546457	-0.040285	-26.586667	-26.586742	-0.076	-47.41
BH_3	-25.242641	-0.027492	-25.270085	-25.270133	-0.048	-30.04
BN	-79.304879	-0.085148	-79.389874	-79.390028	-0.153	-96.20
BO	-99.905347	-0.112380	-100.017456	-100.017727	-0.271	-169.89
C_2	-75.737452	-0.142235	-75.879561	-75.879687	-0.126	-79.33
CCl	-497.918859	-0.114193	-498.032669	-498.033052	-0.383	-240.08
CF	-137.649784	-0.131063	-137.780415	-137.780847	-0.433	-271.42
CH	-40.426329	-0.069808	-40.496005	-40.496137	-0.132	-82.99

Molecule	E_{CBS}^{KS}	E_{CBS}^{MP2}	E_{CBS}^{tot}	$E_{CBS}^{KS} + E_{CBS}^{MP2}$	ΔE_{CBS}	
					mHa	cal/mol
CH_4	-38.426924	-0.037513	-38.464362	-38.464437	-0.076	-47.55
Cl_2	-920.034997	-0.147605	-920.181897	-920.182603	-0.706	-442.73
ClF	-559.710577	-0.168825	-559.878723	-559.879402	-0.679	-425.99
ClN	-514.603339	-0.126621	-514.729532	-514.729960	-0.428	-268.49
ClO	-535.062004	-0.151471	-535.212935	-535.213475	-0.540	-338.55
CN	-92.576935	-0.120778	-92.697528	-92.697713	-0.185	-116.13
CO	-113.177508	-0.131185	-113.308401	-113.308693	-0.292	-183.17
F_2	-199.337060	-0.192679	-199.529042	-199.529739	-0.697	-437.55
H_2	-100.358820	-0.099028	-100.457505	-100.457849	-0.344	-215.59
H_2O	-76.335781	-0.094651	-76.430215	-76.430432	-0.216	-135.72
H_2S	-1.160456	-0.010857	-1.171294	-1.171312	-0.019	-11.67
HCl	-399.224859	-0.071585	-399.296230	-399.296444	-0.214	-134.38
HF	-460.635667	-0.077359	-460.712685	-460.713026	-0.341	-213.78
N_2	-109.384790	-0.137296	-109.521847	-109.522086	-0.240	-150.36
NF	-154.297555	-0.143936	-154.441026	-154.441491	-0.465	-291.64
NH	-56.465784	-0.083946	-56.549576	-56.549730	-0.154	-96.48
NH_3	-55.161168	-0.049944	-55.211001	-55.211112	-0.111	-69.74
NO	-129.740218	-0.148239	-129.888126	-129.888456	-0.330	-207.27
O_2	-150.163671	-0.161605	-150.324860	-150.325275	-0.415	-260.57
OF	-174.715839	-0.168835	-174.884110	-174.884675	-0.565	-354.47
OH	-75.660450	-0.073066	-75.733310	-75.733517	-0.206	-129.58
P_2	-682.394202	-0.109004	-682.502937	-682.503206	-0.269	-168.50
PB	-365.853259	-0.070893	-365.923985	-365.924152	-0.167	-104.49
PC	-801.216540	-0.112679	-801.328687	-801.329220	-0.532	-333.97
PCl	-379.088393	-0.098439	-379.186665	-379.186832	-0.167	-104.77
PF	-440.939211	-0.131755	-441.070498	-441.070966	-0.467	-293.29
PH	-342.976927	-0.063021	-343.039812	-343.039948	-0.136	-85.30

Molecule	E_{CBS}^{KS}	E_{CBS}^{MP2}	E_{CBS}^{tot}	$E_{CBS}^{KS} + E_{CBS}^{MP2}$	ΔE_{CBS}	
					mHa	cal/mol
PH_3	-341.734953	-0.040554	-341.775380	-341.775508	-0.128	-80.23
PN	-395.840533	-0.133013	-395.973349	-395.973546	-0.197	-123.66
PO	-416.319490	-0.135585	-416.454760	-416.455075	-0.314	-197.28
PS	-739.221083	-0.113589	-739.334312	-739.334672	-0.361	-226.30
S_2	-796.061750	-0.122801	-796.184097	-796.184551	-0.454	-284.88
SB	-422.772788	-0.090260	-422.862807	-422.863049	-0.242	-151.84
SC	-858.032030	-0.130727	-858.162156	-858.162757	-0.601	-377.12
SCl	-435.995137	-0.117308	-436.112194	-436.112445	-0.252	-157.87
SF	-497.731708	-0.149673	-497.880825	-497.881381	-0.556	-348.99
SH	-398.589132	-0.057876	-398.646778	-398.647008	-0.229	-143.93
Si_2	-578.585622	-0.062464	-578.647907	-578.648086	-0.179	-112.12
SiB	-313.970180	-0.052837	-314.022916	-314.023017	-0.101	-63.10
SiC	-749.370485	-0.102419	-749.472413	-749.472905	-0.491	-308.35
SiCl	-327.181304	-0.073413	-327.254592	-327.254716	-0.124	-78.05
SiF	-389.106010	-0.122340	-389.227947	-389.228350	-0.402	-252.48
SiH	-291.723133	-0.052199	-291.775267	-291.775332	-0.064	-40.45
SiH_4	-289.849934	-0.030984	-289.880835	-289.880917	-0.082	-51.69
SiN	-343.909267	-0.105691	-344.014798	-344.014959	-0.160	-100.66
SiO	-364.508714	-0.127598	-364.636069	-364.636312	-0.243	-152.45
SiS	-687.403889	-0.101749	-687.505326	-687.505638	-0.311	-195.46
SN	-452.637094	-0.133720	-452.770525	-452.770813	-0.289	-181.17
SO	-473.133715	-0.143823	-473.277141	-473.277538	-0.397	-249.31

Table S14. Complete basis set-limit (CBS) energies for the B2GP-PLYP double-hybrid DFT functional components (Kohn–Sham energy, MP2-like component and total DH energy, all in Ha) and the difference in calculated total energy (defined as $\Delta E_{CBS} = E_{CBS}^{tot} - E_{CBS}^{KS} - E_{CBS}^{\text{MP2}}$, in mHa and cal/mol) for each member of the BSEF74*calculated using the three-point exponential extrapolation (Eq. S23) and the aug-cc-pV*n*Z (*n* = Q, 5, 6) basis sets.

Molecule	E_{CBS}^{KS}	E_{CBS}^{MP2}	E_{CBS}^{tot}	$E_{CBS}^{KS} + E_{CBS}^{MP2}$	ΔE_{CBS}	
					mHa	cal/mol
Al_2	-484.423108	-0.051166	-484.474131	-484.474274	-0.143	-89.56
AlB	-266.858208	-0.051389	-266.909494	-266.909597	-0.103	-64.73
AlC	-702.290661	-0.118500	-702.408672	-702.409160	-0.488	-306.18
AlCl	-280.097660	-0.063510	-280.161027	-280.161170	-0.143	-90.03
AlF	-342.051593	-0.146785	-342.197946	-342.198378	-0.432	-271.24
AlH	-244.007677	-0.043309	-244.050907	-244.050986	-0.079	-49.36
AlH_3	-242.799487	-0.029384	-242.828790	-242.828871	-0.081	-51.09
AlN	-296.783708	-0.092556	-296.876062	-296.876264	-0.202	-126.79
AlO	-317.321623	-0.137582	-317.458925	-317.459205	-0.280	-175.66
AlP	-583.330975	-0.080800	-583.411560	-583.411775	-0.215	-134.94
AlS	-531.466564	-0.059298	-531.525713	-531.525862	-0.149	-93.24
AlSi	-640.228743	-0.106566	-640.335008	-640.335309	-0.301	-188.99
B_2	-49.280135	-0.079269	-49.359340	-49.359404	-0.064	-40.12
BC	-484.700593	-0.126769	-484.827014	-484.827362	-0.348	-218.39
BCl	-62.539428	-0.077707	-62.617021	-62.617134	-0.113	-70.96
BF	-124.495191	-0.151354	-124.646054	-124.646545	-0.490	-307.68
BH	-26.529970	-0.051180	-26.581061	-26.581150	-0.090	-56.20
BH_3	-25.229227	-0.034402	-25.263575	-25.263629	-0.054	-33.91
BN	-79.266602	-0.106438	-79.372860	-79.373040	-0.180	-113.08
BO	-99.858810	-0.141562	-100.000047	-100.000371	-0.325	-203.68
C_2	-75.688553	-0.173097	-75.861501	-75.861650	-0.148	-93.16
CCl	-497.837076	-0.143440	-497.980153	-497.980516	-0.363	-227.79
CF	-137.594069	-0.166341	-137.759887	-137.760410	-0.524	-328.64
CH	-40.400746	-0.088854	-40.489443	-40.489600	-0.157	-98.46

Molecule	E_{CBS}^{KS}	E_{CBS}^{MP2}	E_{CBS}^{tot}	$E_{CBS}^{KS} + E_{CBS}^{MP2}$	ΔE_{CBS}	
					mHa	cal/mol
CH_4	-38.409057	-0.047350	-38.456318	-38.456407	-0.089	-55.83
Cl_2	-919.908157	-0.187003	-920.094527	-920.095160	-0.634	-397.58
ClF	-559.608000	-0.214584	-559.821831	-559.822584	-0.753	-472.49
ClN	-514.516471	-0.158969	-514.675011	-514.675440	-0.429	-269.16
ClO	-534.965807	-0.190031	-535.155265	-535.155838	-0.573	-359.83
CN	-92.529279	-0.148018	-92.677075	-92.677297	-0.222	-139.45
CO	-113.124604	-0.165719	-113.289974	-113.290323	-0.349	-219.13
F_2	-199.257463	-0.245337	-199.501956	-199.502800	-0.844	-529.92
H_2	-100.319544	-0.126886	-100.446015	-100.446430	-0.415	-260.32
H_2O	-76.299426	-0.120739	-76.419907	-76.420165	-0.258	-162.12
H_2S	-1.157232	-0.013835	-1.171045	-1.171067	-0.022	-13.91
HCl	-399.162900	-0.090626	-399.253345	-399.253526	-0.180	-113.08
HF	-460.571569	-0.098317	-460.669585	-460.669885	-0.301	-188.70
N_2	-109.330751	-0.173136	-109.503602	-109.503887	-0.285	-178.66
NF	-154.236469	-0.182409	-154.418316	-154.418878	-0.563	-353.10
NH	-56.434393	-0.106885	-56.541103	-56.541278	-0.175	-109.56
NH_3	-55.138729	-0.063550	-55.202146	-55.202278	-0.132	-83.03
NO	-129.679870	-0.187122	-129.866592	-129.866992	-0.400	-250.82
O_2	-150.096738	-0.204339	-150.300574	-150.301076	-0.503	-315.35
OF	-174.644891	-0.213524	-174.857733	-174.858416	-0.682	-428.22
OH	-75.629415	-0.093321	-75.722487	-75.722735	-0.248	-155.73
P_2	-682.281632	-0.135751	-682.417168	-682.417384	-0.216	-135.56
PB	-365.786135	-0.087876	-365.873861	-365.874011	-0.150	-94.02
PC	-801.101071	-0.142040	-801.242630	-801.243111	-0.482	-302.37
PCl	-379.013681	-0.117635	-379.131169	-379.131316	-0.147	-92.26
PF	-440.849072	-0.167291	-441.015839	-441.016363	-0.524	-328.60
PH	-342.918091	-0.079595	-342.997567	-342.997685	-0.118	-74.24

Molecule	E_{CBS}^{KS}	E_{CBS}^{MP2}	E_{CBS}^{tot}	$E_{CBS}^{KS} + E_{CBS}^{MP2}$	ΔE_{CBS}	
					mHa	cal/mol
PH_3	-341.681981	-0.051011	-341.732873	-341.732993	-0.119	-74.83
PN	-395.754862	-0.165768	-395.920452	-395.920630	-0.178	-111.47
PO	-416.230075	-0.170356	-416.400098	-416.400431	-0.333	-208.70
PS	-739.105778	-0.141925	-739.247407	-739.247703	-0.296	-185.87
S_2	-795.942734	-0.154173	-796.096527	-796.096906	-0.379	-237.90
SB	-422.700521	-0.112797	-422.813109	-422.813318	-0.209	-131.19
SC	-857.910360	-0.164804	-858.074637	-858.075165	-0.527	-330.90
SCl	-435.914133	-0.146429	-436.060343	-436.060561	-0.218	-136.97
SF	-497.635242	-0.189848	-497.824481	-497.825089	-0.608	-381.57
SH	-398.530490	-0.073181	-398.603472	-398.603671	-0.199	-124.71
Si_2	-578.485284	-0.077634	-578.562753	-578.562917	-0.164	-102.92
SiB	-313.908347	-0.065311	-313.973563	-313.973658	-0.095	-59.74
SiC	-749.258302	-0.129159	-749.387013	-749.387461	-0.448	-280.97
SiCl	-327.112845	-0.088343	-327.201069	-327.201188	-0.119	-74.65
SiF	-389.018936	-0.155440	-389.173918	-389.174377	-0.459	-287.72
SiH	-291.668773	-0.066050	-291.734769	-291.734823	-0.054	-33.96
SiH_4	-289.799658	-0.038766	-289.838346	-289.838424	-0.078	-48.94
SiN	-343.830562	-0.129137	-343.959532	-343.959698	-0.166	-104.38
SiO	-364.422650	-0.160159	-364.582545	-364.582809	-0.264	-165.40
SiS	-687.292969	-0.127303	-687.420017	-687.420272	-0.255	-160.01
SN	-452.549398	-0.164495	-452.713625	-452.713893	-0.268	-168.18
SO	-473.040694	-0.181080	-473.221363	-473.221774	-0.411	-258.14

Table S15. Complete basis set-limit (CBS) energies for the mPW2PLYP double-hybrid DFT functional components (Kohn–Sham energy, MP2-like component and total DH energy, all in Ha) and the difference in calculated total energy (defined as $\Delta E_{CBS} = E_{CBS}^{tot} - E_{CBS}^{KS} - E_{CBS}^{\mathrm{MP2}}$, in mHa and cal/mol) for each member of the BSEF74* calculated using the three-point exponential extrapolation (Eq. S23) and the aug-cc-pVnZ (n = Q, 5, 6) basis sets.

Molecule	E_{CBS}^{KS}	E_{CBS}^{MP2}	E_{CBS}^{tot}	$E_{CBS}^{KS} + E_{CBS}^{MP2}$	ΔE_{CBS}	
					mHa	cal/mol
Al_2	-484.544114	-0.040991	-484.584933	-484.585105	-0.172	-107.80
AlB	-266.932742	-0.043113	-266.975738	-266.975855	-0.117	-73.54
AlC	-702.430670	-0.090172	-702.520257	-702.520842	-0.585	-367.05
AlCl	-280.175510	-0.050095	-280.225454	-280.225605	-0.151	-94.86
AlF	-342.161150	-0.110481	-342.271242	-342.271631	-0.389	-243.90
AlH	-244.068241	-0.032964	-244.101107	-244.101206	-0.098	-61.54
AlH_3	-242.859565	-0.022819	-242.882283	-242.882384	-0.101	-63.61
AlN	-296.874882	-0.073619	-296.948297	-296.948501	-0.204	-128.21
AlO	-317.429846	-0.109949	-317.539533	-317.539795	-0.262	-164.29
AlP	-583.460895	-0.063471	-583.524113	-583.524366	-0.253	-158.77
AlS	-531.589197	-0.046814	-531.635847	-531.636011	-0.164	-102.86
AlSi	-640.365737	-0.082677	-640.448029	-640.448414	-0.385	-241.36
B_2	-49.316837	-0.065080	-49.381864	-49.381917	-0.053	-33.40
BC	-484.796651	-0.097062	-484.893301	-484.893714	-0.413	-258.90
BCl	-62.576258	-0.061673	-62.637840	-62.637931	-0.091	-57.41
BF	-124.559009	-0.113985	-124.672616	-124.672994	-0.378	-237.39
BH	-26.546737	-0.038601	-26.585268	-26.585338	-0.071	-44.32
BH_3	-25.243318	-0.026662	-25.269934	-25.269979	-0.046	-28.57
BN	-79.314170	-0.082650	-79.396674	-79.396820	-0.146	-91.67
BO	-99.918252	-0.108444	-100.026440	-100.026696	-0.256	-160.70
C_2	-75.753404	-0.140870	-75.894158	-75.894275	-0.116	-72.99
CCl	-497.941573	-0.110377	-498.051544	-498.051950	-0.406	-254.60
CF	-137.665500	-0.125761	-137.790859	-137.791261	-0.401	-251.80
CH	-40.429854	-0.066808	-40.496537	-40.496663	-0.126	-78.98

Molecule	E_{CBS}^{KS}	E_{CBS}^{MP2}	E_{CBS}^{tot}	$E_{CBS}^{KS} + E_{CBS}^{MP2}$	ΔE_{CBS}	
					mHa	cal/mol
CH_4	-38.429195	-0.036137	-38.465261	-38.465332	-0.072	-44.91
Cl_2	-920.072219	-0.141729	-920.213181	-920.213948	-0.767	-481.24
ClF	-559.742051	-0.161779	-559.903161	-559.903830	-0.669	-419.85
ClN	-514.628133	-0.122422	-514.750113	-514.750555	-0.442	-277.35
ClO	-535.091230	-0.146520	-535.237207	-535.237749	-0.542	-340.08
CN	-92.590881	-0.118469	-92.709180	-92.709350	-0.170	-106.78
CO	-113.192660	-0.126274	-113.318663	-113.318933	-0.270	-169.35
F_2	-199.363533	-0.184468	-199.547356	-199.548001	-0.645	-404.87
H_2	-100.370174	-0.094370	-100.464218	-100.464543	-0.325	-203.95
H_2O	-76.345706	-0.090485	-76.435987	-76.436192	-0.204	-128.24
H_2S	-1.159726	-0.010374	-1.170082	-1.170100	-0.018	-11.03
HCl	-399.241417	-0.068757	-399.309934	-399.310174	-0.240	-150.54
HF	-460.653247	-0.074097	-460.726969	-460.727344	-0.375	-235.48
N_2	-109.400583	-0.132313	-109.532673	-109.532896	-0.224	-140.54
NF	-154.315510	-0.138260	-154.453341	-154.453770	-0.430	-269.62
NH	-56.472833	-0.080345	-56.553029	-56.553177	-0.148	-93.11
NH_3	-55.165151	-0.047814	-55.212860	-55.212965	-0.104	-65.57
NO	-129.758810	-0.142753	-129.901258	-129.901563	-0.304	-190.90
O_2	-150.185177	-0.155429	-150.340226	-150.340606	-0.381	-238.98
OF	-174.738549	-0.162503	-174.900530	-174.901051	-0.521	-327.10
OH	-75.668204	-0.069782	-75.737793	-75.737986	-0.194	-121.49
P_2	-682.427985	-0.105984	-682.533668	-682.533969	-0.301	-188.73
PB	-365.871152	-0.069116	-365.940089	-365.940268	-0.179	-112.42
PC	-801.249743	-0.108577	-801.357744	-801.358319	-0.575	-360.83
PCl	-379.110083	-0.097788	-379.207688	-379.207872	-0.183	-115.12
PF	-440.966005	-0.126351	-441.091895	-441.092356	-0.461	-289.45
PH	-342.991738	-0.060640	-343.052226	-343.052378	-0.152	-95.68

Molecule	E_{CBS}^{KS}	E_{CBS}^{MP2}	E_{CBS}^{tot}	$E_{CBS}^{KS} + E_{CBS}^{MP2}$	ΔE_{CBS}	
					mHa	cal/mol
PH_3	-341.748775	-0.039119	-341.787757	-341.787893	-0.136	-85.64
PN	-395.867054	-0.129316	-395.996157	-395.996371	-0.214	-134.12
PO	-416.347094	-0.131032	-416.477806	-416.478126	-0.320	-200.82
PS	-739.255374	-0.110123	-739.365093	-739.365497	-0.404	-253.59
S_2	-796.097103	-0.118664	-796.215261	-796.215767	-0.506	-317.22
SB	-422.792319	-0.087542	-422.879593	-422.879861	-0.268	-168.05
SC	-858.067608	-0.125979	-858.192929	-858.193587	-0.659	-413.38
SCl	-436.018621	-0.113901	-436.132244	-436.132522	-0.278	-174.54
SF	-497.760931	-0.143653	-497.904027	-497.904583	-0.556	-349.01
SH	-398.604899	-0.055633	-398.660280	-398.660533	-0.253	-158.61
Si_2	-578.614627	-0.060802	-578.675238	-578.675429	-0.191	-119.78
SiB	-313.986286	-0.051637	-314.037817	-314.037923	-0.106	-66.38
SiC	-749.402703	-0.098676	-749.500848	-749.501379	-0.530	-332.86
SiCl	-327.200434	-0.072847	-327.273150	-327.273282	-0.132	-82.78
SiF	-389.131971	-0.117285	-389.248864	-389.249256	-0.393	-246.39
SiH	-291.735168	-0.050163	-291.785259	-291.785331	-0.072	-45.06
SiH_4	-289.863132	-0.030012	-289.893056	-289.893144	-0.088	-54.91
SiN	-343.933175	-0.104174	-344.037187	-344.037350	-0.163	-102.03
SiO	-364.535389	-0.123451	-364.658598	-364.658839	-0.242	-151.57
SiS	-687.436547	-0.098609	-687.534806	-687.535155	-0.349	-218.97
SN	-452.663786	-0.130200	-452.793674	-452.793986	-0.312	-195.85
SO	-473.162304	-0.138741	-473.300637	-473.301044	-0.407	-255.36

Table S16. Complete basis set-limit (CBS) energies for the PWPB95 double-hybrid DFT functional components (Kohn–Sham energy, MP2-like component and total DH energy, all in Ha) and the difference in calculated total energy (defined as $\Delta E_{CBS} = E_{CBS}^{tot} - E_{CBS}^{KS} - E_{CBS}^{\mathrm{MP2}}$, in mHa and cal/mol) for each member of the BSEF74* calculated using the three-point exponential extrapolation (Eq. S23) and the aug-cc-pVnZ (n = Q, 5, 6) basis sets.

Molecule	E_{CBS}^{KS}	E_{CBS}^{MP2}	E_{CBS}^{tot}	$E_{CBS}^{KS} + E_{CBS}^{MP2}$	ΔE_{CBS}	
					mHa	cal/mol
Al_2	-484.527899	-0.124679	-484.652320	-484.652578	-0.258	-161.94
AlB	-266.915023	-0.124094	-267.038907	-267.039117	-0.210	-131.52
AlC	-702.431312	-0.075023	-702.505780	-702.506335	-0.555	-348.37
AlCl	-280.155303	-0.146101	-280.301173	-280.301404	-0.231	-144.92
AlF	-342.131742	-0.089677	-342.221043	-342.221418	-0.375	-235.43
AlH	-244.051271	-0.033156	-244.084333	-244.084427	-0.094	-58.76
AlH_3	-242.845449	-0.022881	-242.868266	-242.868330	-0.064	-40.15
AlN	-296.847659	-0.221529	-297.068869	-297.069188	-0.319	-200.03
AlO	-317.400433	-0.320773	-317.720728	-317.721206	-0.478	-299.89
AlP	-583.451044	-0.196027	-583.646555	-583.647071	-0.516	-324.03
AlS	-531.576809	-0.140895	-531.717366	-531.717703	-0.337	-211.75
AlSi	-640.362667	-0.249591	-640.611507	-640.612258	-0.751	-471.32
B_2	-49.299048	-0.190287	-49.489218	-49.489334	-0.116	-72.62
BC	-484.798820	-0.080373	-484.878744	-484.879193	-0.449	-281.60
BCl	-62.555519	-0.179283	-62.734630	-62.734802	-0.172	-107.96
BF	-124.531528	-0.092587	-124.623690	-124.624114	-0.425	-266.59
BH	-26.529178	-0.036897	-26.565994	-26.566075	-0.081	-50.82
BH_3	-25.229090	-0.026375	-25.255406	-25.255465	-0.060	-37.37
BN	-79.287658	-0.245040	-79.532436	-79.532698	-0.262	-164.29
BO	-99.891483	-0.315824	-100.206811	-100.207307	-0.497	-311.57
C_2	-75.732586	-0.110934	-75.843397	-75.843520	-0.123	-77.31
CCl	-497.940245	-0.333987	-498.273497	-498.274233	-0.736	-461.54
CF	-137.635508	-0.376104	-138.010829	-138.011612	-0.783	-491.35
CH	-40.414856	-0.058182	-40.472921	-40.473038	-0.117	-73.18

Molecule	E_{CBS}^{KS}	E_{CBS}^{MP2}	E_{CBS}^{tot}	$E_{CBS}^{KS} + E_{CBS}^{MP2}$	ΔE_{CBS}	
					mHa	cal/mol
CH_4	-38.411262	-0.122489	-38.533614	-38.533751	-0.137	-85.85
Cl_2	-920.094505	-0.115930	-920.209564	-920.210434	-0.871	-546.34
ClF	-559.735003	-0.130938	-559.865198	-559.865941	-0.744	-466.61
ClN	-514.625134	-0.360885	-514.985193	-514.986019	-0.826	-518.11
ClO	-535.084839	-0.435333	-535.519170	-535.520172	-1.003	-629.12
CN	-92.566526	-0.345352	-92.911585	-92.911878	-0.293	-184.01
CO	-113.167001	-0.100945	-113.267653	-113.267946	-0.293	-183.65
F_2	-199.327340	-0.148575	-199.475213	-199.475915	-0.703	-440.86
H_2	-100.352056	-0.075761	-100.427461	-100.427817	-0.356	-223.25
H_2O	-76.327918	-0.073385	-76.401083	-76.401303	-0.220	-138.12
H_2S	-1.155581	-0.011005	-1.166566	-1.166586	-0.019	-12.13
HCl	-399.244868	-0.058677	-399.303282	-399.303546	-0.263	-165.23
HF	-460.662712	-0.061586	-460.723900	-460.724299	-0.399	-250.49
N_2	-109.376413	-0.105182	-109.481360	-109.481595	-0.234	-147.00
NF	-154.284152	-0.403871	-154.687182	-154.688024	-0.842	-528.17
NH	-56.456714	-0.066780	-56.523355	-56.523494	-0.140	-87.69
NH_3	-55.147549	-0.145529	-55.292884	-55.293078	-0.194	-121.76
NO	-129.730490	-0.418846	-130.148783	-130.149337	-0.553	-347.24
O_2	-150.153974	-0.437652	-150.590899	-150.591627	-0.728	-457.00
OF	-174.702833	-0.481527	-175.183366	-175.184360	-0.994	-623.67
OH	-75.647704	-0.213605	-75.860948	-75.861310	-0.361	-226.84
P_2	-682.431081	-0.085905	-682.516625	-682.516987	-0.361	-226.83
PB	-365.861399	-0.207777	-366.068776	-366.069176	-0.400	-251.22
PC	-801.260743	-0.326367	-801.585941	-801.587110	-1.170	-733.99
PCl	-379.100017	-0.286493	-379.386110	-379.386510	-0.399	-250.51
PF	-440.947004	-0.374853	-441.320881	-441.321857	-0.976	-612.40
PH	-342.988332	-0.054136	-343.042301	-343.042468	-0.168	-105.20

Molecule	E_{CBS}^{KS}	E_{CBS}^{MP2}	E_{CBS}^{tot}	$E_{CBS}^{KS} + E_{CBS}^{MP2}$	ΔE_{CBS}	
					mHa	cal/mol
PH_3	-341.744984	-0.123866	-341.868501	-341.868850	-0.349	-218.81
PN	-395.853504	-0.102457	-395.955717	-395.955961	-0.244	-153.00
PO	-416.329107	-0.388048	-416.716480	-416.717155	-0.676	-423.91
PS	-739.261994	-0.331806	-739.592928	-739.593800	-0.873	-547.52
S_2	-796.108868	-0.349099	-796.456910	-796.457967	-1.058	-663.71
SB	-422.789636	-0.260403	-423.049495	-423.050039	-0.545	-341.74
SC	-858.083292	-0.382115	-858.464100	-858.465407	-1.307	-820.22
SCl	-436.014286	-0.091693	-436.105669	-436.105978	-0.309	-194.03
SF	-497.746699	-0.431244	-498.176824	-498.177943	-1.119	-701.95
SH	-398.605813	-0.176587	-398.781879	-398.782400	-0.520	-326.50
Si_2	-578.604579	-0.185192	-578.789345	-578.789771	-0.426	-267.29
SiB	-313.973616	-0.149315	-314.122690	-314.122931	-0.241	-151.24
SiC	-749.407454	-0.302872	-749.709315	-749.710326	-1.012	-634.75
SiCl	-327.183100	-0.215816	-327.398648	-327.398916	-0.268	-168.33
SiF	-389.106466	-0.353476	-389.459122	-389.459942	-0.820	-514.69
SiH	-291.723013	-0.047863	-291.770790	-291.770875	-0.086	-53.78
SiH_4	-289.852534	-0.104674	-289.957012	-289.957208	-0.196	-123.17
SiN	-343.912519	-0.305829	-344.217999	-344.218348	-0.349	-219.24
SiO	-364.512173	-0.098489	-364.610392	-364.610662	-0.270	-169.43
SiS	-687.438640	-0.080735	-687.518986	-687.519375	-0.389	-243.91
SN	-452.655230	-0.384545	-453.039169	-453.039775	-0.606	-380.23
SO	-473.150784	-0.400615	-473.550584	-473.551399	-0.815	-511.45

Table S17. Complete basis set-limit (CBS) energies for the rev-DSD-PBEP86-D3BJ double-hybrid DFT functional components (Kohn–Sham energy, MP2-like component and total DH energy, all in Ha) and the difference in calculated total energy (defined as $\Delta E_{CBS} = E_{CBS}^{tot} - E_{CBS}^{KS} - E_{CBS}^{MP2}$, in mHa and cal/mol) for each member of the BSEF74* calculated using the three-point exponential extrapolation (Eq. S23) and the aug-cc-pVnZ (n = Q, 5, 6) basis sets.

Molecule	E_{CBS}^{KS}	E_{CBS}^{MP2}	E_{CBS}^{tot}	$E_{CBS}^{KS} + E_{CBS}^{MP2}$	ΔE_{CBS}	
					mHa	cal/mol
Al_2	-484.173328	-0.112986	-484.286153	-484.286314	-0.160	-100.70
AlB	-266.703076	-0.101888	-266.804829	-266.804964	-0.134	-84.33
AlC	-701.992498	-0.152739	-702.144812	-702.145237	-0.426	-267.22
AlCl	-279.931136	-0.135823	-280.066758	-280.066959	-0.201	-126.10
AlF	-341.833436	-0.185911	-342.018802	-342.019347	-0.545	-342.09
AlH	-243.869017	-0.065409	-243.934341	-243.934426	-0.084	-52.98
AlH_3	-242.667312	-0.044178	-242.711415	-242.711491	-0.076	-47.52
AlN	-296.598993	-0.203836	-296.802541	-296.802829	-0.289	-181.11
AlO	-317.118218	-0.296379	-317.414178	-317.414597	-0.419	-262.94
AlP	-583.059833	-0.180782	-583.240362	-583.240615	-0.253	-158.99
AlS	-531.209269	-0.130462	-531.339541	-531.339731	-0.190	-119.12
AlSi	-639.944430	-0.235571	-640.179684	-640.180002	-0.318	-199.71
B_2	-49.211966	-0.171912	-49.383775	-49.383878	-0.103	-64.40
BC	-484.495034	-0.162761	-484.657479	-484.657795	-0.316	-198.24
BCl	-62.463949	-0.162023	-62.625788	-62.625973	-0.185	-115.87
BF	-124.370195	-0.191643	-124.561169	-124.561838	-0.669	-419.80
BH	-26.482343	-0.074044	-26.556273	-26.556387	-0.114	-71.66
BH_3	-25.188962	-0.051144	-25.240036	-25.240105	-0.069	-43.51
BN	-79.174711	-0.230629	-79.405061	-79.405340	-0.278	-174.68
BO	-99.750272	-0.304282	-100.054030	-100.054554	-0.524	-328.65
C_2	-75.591750	-0.213725	-75.805271	-75.805475	-0.204	-128.03
CCl	-497.622658	-0.318122	-497.940372	-497.940780	-0.408	-256.02
CF	-137.461313	-0.366041	-137.826489	-137.827354	-0.865	-543.00
CH	-40.340494	-0.119012	-40.459307	-40.459506	-0.199	-124.98

Molecule	E_{CBS}^{KS}	E_{CBS}^{MP2}	E_{CBS}^{tot}	$E_{CBS}^{KS} + E_{CBS}^{MP2}$	ΔE_{CBS}	
					mHa	cal/mol
CH_4	-38.360491	-0.115026	-38.475380	-38.475518	-0.138	-86.64
Cl_2	-919.569533	-0.237822	-919.806820	-919.807355	-0.535	-335.95
ClF	-559.350442	-0.270948	-559.620469	-559.621390	-0.921	-578.09
ClN	-514.292700	-0.345001	-514.637164	-514.637701	-0.537	-336.91
ClO	-534.724490	-0.415910	-535.139619	-535.140400	-0.782	-490.43
CN	-92.425377	-0.309841	-92.734874	-92.735218	-0.344	-215.86
CO	-113.003225	-0.207010	-113.209765	-113.210235	-0.471	-295.43
F_2	-199.082260	-0.308649	-199.389744	-199.390909	-1.165	-731.19
H_2	-100.229016	-0.159477	-100.387929	-100.388493	-0.564	-353.70
H_2O	-76.219443	-0.152890	-76.371990	-76.372333	-0.343	-215.12
H_2S	-1.150340	-0.021886	-1.172198	-1.172226	-0.028	-17.26
HCl	-398.998760	-0.119275	-399.117873	-399.118035	-0.161	-101.29
HF	-460.399293	-0.126745	-460.525787	-460.526039	-0.252	-157.93
N_2	-109.211188	-0.215192	-109.425999	-109.426380	-0.381	-239.09
NF	-154.095353	-0.393946	-154.488359	-154.489299	-0.940	-589.69
NH	-56.365059	-0.137942	-56.502776	-56.503001	-0.225	-141.12
NH_3	-55.082273	-0.141431	-55.223489	-55.223703	-0.215	-134.62
NO	-129.548330	-0.405254	-129.952943	-129.953584	-0.641	-402.11
O_2	-149.953280	-0.429448	-150.381892	-150.382728	-0.836	-524.76
OF	-174.486459	-0.467082	-174.952400	-174.953541	-1.141	-715.93
OH	-75.555419	-0.209418	-75.764435	-75.764837	-0.402	-252.41
P_2	-681.986147	-0.171018	-682.156972	-682.157164	-0.192	-120.56
PB	-365.607973	-0.190426	-365.798215	-365.798399	-0.184	-115.16
PC	-800.789333	-0.312514	-801.101351	-801.101847	-0.497	-311.79
PCl	-378.824420	-0.239035	-379.063258	-379.063455	-0.198	-123.94
PF	-440.617943	-0.365182	-440.982295	-440.983125	-0.830	-520.83
PH	-342.761122	-0.108672	-342.869676	-342.869794	-0.118	-74.02

Molecule	E_{CBS}^{KS}	E_{CBS}^{MP2}	E_{CBS}^{tot}	$E_{CBS}^{KS} + E_{CBS}^{MP2}$	ΔE_{CBS}	
					mHa	cal/mol
PH_3	-341.536980	-0.116457	-341.653283	-341.653437	-0.154	-96.46
PN	-395.545197	-0.205584	-395.750557	-395.750780	-0.223	-139.99
PO	-416.008388	-0.371412	-416.379292	-416.379800	-0.508	-318.57
PS	-738.801346	-0.311368	-739.112425	-739.112714	-0.289	-181.63
S_2	-795.629061	-0.333742	-795.962434	-795.962803	-0.369	-231.40
SB	-422.509377	-0.245095	-422.754244	-422.754472	-0.228	-143.25
SC	-857.584786	-0.365887	-857.950166	-857.950673	-0.507	-318.36
SCl	-435.708019	-0.183804	-435.891597	-435.891822	-0.225	-141.24
SF	-497.390560	-0.418812	-497.808467	-497.809372	-0.905	-567.79
SH	-398.371348	-0.168274	-398.539421	-398.539622	-0.201	-125.90
Si_2	-578.215466	-0.172171	-578.387446	-578.387637	-0.191	-120.09
SiB	-313.743402	-0.137730	-313.881003	-313.881132	-0.128	-80.54
SiC	-748.953310	-0.289313	-749.242181	-749.242623	-0.442	-277.62
SiCl	-326.934809	-0.184763	-327.119407	-327.119572	-0.165	-103.79
SiF	-388.794155	-0.343950	-389.137375	-389.138105	-0.731	-458.41
SiH	-291.517814	-0.095383	-291.613138	-291.613198	-0.059	-37.10
SiH_4	-289.660697	-0.096017	-289.756618	-289.756714	-0.096	-60.24
SiN	-343.633977	-0.274132	-343.907855	-343.908109	-0.254	-159.61
SiO	-364.208234	-0.199947	-364.407831	-364.408181	-0.350	-219.87
SiS	-686.996722	-0.161789	-687.158293	-687.158511	-0.218	-136.53
SN	-452.332353	-0.349838	-452.681846	-452.682191	-0.345	-216.62
SO	-472.810918	-0.387076	-473.197399	-473.197993	-0.595	-373.24

Table S18. Complete basis set-limit (CBS) energies for the rev-DSD-PBEP86-D4 double-hybrid DFT functional components (Kohn–Sham energy, MP2-like component and total DH energy, all in Ha) and the difference in calculated total energy (defined as $\Delta E_{CBS} = E_{CBS}^{tot} - E_{CBS}^{KS} - E_{CBS}^{\text{MP2}}$, in mHa and cal/mol) for each member of the BSEF74* calculated using the three-point exponential extrapolation (Eq. S23) and the aug-cc-pVnZ (n = Q, 5, 6) basis sets.

Molecule	E_{CBS}^{KS}	E_{CBS}^{MP2}	E_{CBS}^{tot}	$E_{CBS}^{KS} + E_{CBS}^{MP2}$	ΔE_{CBS}	
					mHa	cal/mol
Al_2	-484.164325	-0.112974	-484.277138	-484.277300	-0.162	-101.62
AlB	-266.697434	-0.101494	-266.798792	-266.798928	-0.135	-84.97
AlC	-701.981407	-0.155102	-702.136079	-702.136510	-0.431	-270.40
AlCl	-279.925121	-0.135595	-280.060513	-280.060716	-0.202	-127.04
AlF	-341.825874	-0.188638	-342.013959	-342.014511	-0.552	-346.39
AlH	-243.863794	-0.066848	-243.930557	-243.930642	-0.086	-53.67
AlH_3	-242.662607	-0.045147	-242.707678	-242.707754	-0.077	-48.17
AlN	-296.592460	-0.203768	-296.795938	-296.796228	-0.291	-182.30
AlO	-317.111116	-0.295925	-317.406619	-317.407041	-0.422	-264.85
AlP	-583.049839	-0.180828	-583.230412	-583.230668	-0.256	-160.66
AlS	-531.199840	-0.130401	-531.330049	-531.330241	-0.192	-120.41
AlSi	-639.933848	-0.235476	-640.169003	-640.169324	-0.321	-201.51
B_2	-49.209527	-0.171810	-49.381234	-49.381337	-0.103	-64.73
BC	-484.487226	-0.165257	-484.652163	-484.652483	-0.320	-200.58
BCl	-62.461185	-0.161598	-62.622597	-62.622783	-0.186	-116.64
BF	-124.365910	-0.194450	-124.559683	-124.560360	-0.677	-424.89
BH	-26.480355	-0.075544	-26.555783	-26.555898	-0.115	-72.40
BH_3	-25.187560	-0.052243	-25.239734	-25.239804	-0.070	-43.88
BN	-79.171435	-0.230350	-79.401505	-79.401785	-0.280	-175.63
BO	-99.746466	-0.303770	-100.049710	-100.050237	-0.527	-330.53
C_2	-75.588326	-0.216779	-75.804899	-75.805105	-0.206	-129.29
CCl	-497.614464	-0.318013	-497.932066	-497.932477	-0.411	-258.00
CF	-137.456652	-0.365756	-137.821538	-137.822408	-0.870	-545.94
CH	-40.337672	-0.121048	-40.458518	-40.458720	-0.201	-126.31

Molecule	E_{CBS}^{KS}	E_{CBS}^{MP2}	E_{CBS}^{tot}	$E_{CBS}^{KS} + E_{CBS}^{MP2}$	ΔE_{CBS}	
					mHa	cal/mol
CH_4	-38.358716	-0.115433	-38.474010	-38.474149	-0.139	-87.01
Cl_2	-919.556446	-0.241355	-919.797259	-919.797801	-0.543	-340.43
ClF	-559.340898	-0.274881	-559.614847	-559.615779	-0.932	-584.81
ClN	-514.284117	-0.344583	-514.628160	-514.628701	-0.541	-339.42
ClO	-534.715404	-0.415553	-535.130171	-535.130958	-0.787	-493.71
CN	-92.421580	-0.309177	-92.730411	-92.730757	-0.346	-217.17
CO	-112.998899	-0.209943	-113.208365	-113.208841	-0.476	-298.76
F_2	-199.076271	-0.313094	-199.388185	-199.389364	-1.179	-740.03
H_2	-100.225759	-0.161755	-100.386944	-100.387515	-0.570	-357.95
H_2O	-76.216313	-0.155137	-76.371104	-76.371451	-0.347	-217.46
H_2S	-1.149938	-0.022404	-1.172314	-1.172342	-0.028	-17.44
HCl	-398.992222	-0.121224	-399.113283	-399.113446	-0.164	-102.71
HF	-460.392532	-0.128700	-460.520977	-460.521232	-0.255	-159.93
N_2	-109.206843	-0.218205	-109.424663	-109.425048	-0.385	-241.65
NF	-154.090320	-0.393344	-154.482719	-154.483664	-0.945	-592.93
NH	-56.362065	-0.140088	-56.501926	-56.502153	-0.227	-142.55
NH_3	-55.080096	-0.141398	-55.221278	-55.221493	-0.216	-135.36
NO	-129.543595	-0.404704	-129.947655	-129.948299	-0.644	-404.26
O_2	-149.948155	-0.428264	-150.375577	-150.376419	-0.842	-528.06
OF	-174.480945	-0.466647	-174.946444	-174.947591	-1.147	-719.73
OH	-75.552706	-0.209436	-75.761737	-75.762142	-0.404	-253.67
P_2	-681.975040	-0.173533	-682.148378	-682.148573	-0.195	-122.39
PB	-365.601288	-0.190228	-365.791331	-365.791517	-0.185	-116.25
PC	-800.777354	-0.312297	-801.089150	-801.089652	-0.502	-314.95
PCl	-378.817288	-0.238411	-379.055500	-379.055700	-0.199	-125.14
PF	-440.609513	-0.364770	-440.973448	-440.974284	-0.836	-524.40
PH	-342.754835	-0.110615	-342.865331	-342.865450	-0.120	-75.03

Molecule	E_{CBS}^{KS}	E_{CBS}^{MP2}	E_{CBS}^{tot}	$E_{CBS}^{KS} + E_{CBS}^{MP2}$	ΔE_{CBS}	
					mHa	cal/mol
PH_3	-341.531396	-0.116572	-341.647813	-341.647969	-0.156	-97.59
PN	-395.537493	-0.208457	-395.745725	-395.745949	-0.224	-140.68
PO	-416.000290	-0.371027	-416.370806	-416.371317	-0.511	-320.88
PS	-738.789779	-0.311167	-739.100654	-739.100946	-0.293	-183.64
S_2	-795.617011	-0.333290	-795.949927	-795.950300	-0.373	-233.93
SB	-422.502111	-0.244818	-422.746699	-422.746929	-0.231	-144.70
SC	-857.572244	-0.365770	-857.937502	-857.938015	-0.513	-321.65
SCl	-435.700256	-0.186468	-435.886496	-435.886724	-0.228	-143.04
SF	-497.381570	-0.418532	-497.799192	-497.800102	-0.911	-571.55
SH	-398.365180	-0.168473	-398.533450	-398.533653	-0.203	-127.24
Si_2	-578.205509	-0.172154	-578.377470	-578.377663	-0.193	-121.24
SiB	-313.737280	-0.137418	-313.874568	-313.874697	-0.130	-81.28
SiC	-748.941790	-0.289327	-749.230671	-749.231117	-0.446	-279.92
SiCl	-326.928248	-0.184439	-327.112520	-327.112687	-0.167	-104.68
SiF	-388.786176	-0.343762	-389.129203	-389.129938	-0.735	-461.29
SiH	-291.511809	-0.097305	-291.609054	-291.609114	-0.060	-37.67
SiH_4	-289.655569	-0.096437	-289.751909	-289.752006	-0.097	-60.70
SiN	-343.626867	-0.273690	-343.900301	-343.900557	-0.256	-160.69
SiO	-364.200593	-0.202788	-364.403026	-364.403381	-0.355	-222.73
SiS	-686.985616	-0.164215	-687.149611	-687.149831	-0.221	-138.45
SN	-452.324194	-0.349329	-452.673175	-452.673523	-0.348	-218.33
SO	-472.802338	-0.386318	-473.188056	-473.188655	-0.599	-376.07

Table S19. Schwenke *f* extrapolation parameters for two basis-set extrapolations and associated mean absolute and signed deviations (MAD and MSD, respectively, in kcal/mol) for Dunning's cc-pV*n*Z for the Kohn–Sham, MP2-like correlation and total double-hybrid DFT energy for selected double-hybrid functionals.

Functional	Component	*f*				MAD				MSD[a]			
		{D,T}	{T,Q}	{Q,5}	{5,6}	{D,T}	{T,Q}	{Q,5}	{5,6}	{D,T}	{T,Q}	{Q,5}	{5,6}
B2PLYP	KS	0.3520	0.3807	0.2298	0.2607	0.49	0.29	0.06	0.01	0.15	0.05	-0.01	0.00
	MP2	0.6329	0.7745	0.8371	0.8917	0.27	0.12	0.06	0.03	-0.05	0.00	0.04	0.01
	KS+MP2	0.4538	0.5426	0.5044	0.5724	0.81	0.51	0.17	0.05	0.16	0.11	0.03	0.01
B2GP-PLYP	KS	0.3415	0.3417	0.2141	0.2622	0.39	0.17	0.06	0.01	0.05	0.01	0.02	0.00
	MP2	0.6458	0.7838	0.8507	0.9009	0.37	0.15	0.08	0.04	-0.07	0.01	0.05	0.02
	KS+MP2	0.4710	0.5562	0.5694	0.6535	0.81	0.47	0.25	0.08	0.08	0.10	0.10	0.01
mPW2PLYP	KS	0.3627	0.4151	0.2274	0.2549	0.60	0.42	0.06	0.01	0.22	0.10	-0.01	0.00
	MP2	0.6248	0.7712	0.8292	0.8847	0.25	0.11	0.06	0.02	-0.04	0.00	0.04	0.01
	KS+MP2	0.4541	0.5525	0.4757	0.5382	0.88	0.60	0.15	0.03	0.19	0.13	0.01	0.01
PWPB95	KS	0.3617	0.3819	0.2186	0.2600	0.56	0.31	0.04	0.01	0.13	0.08	0.00	0.00
	MP2	0.7136	0.8327	0.8888	0.9333	0.62	0.23	0.10	0.05	-0.07	-0.06	0.05	0.02
	KS+MP2	0.5672	0.6718	0.7083	0.7636	1.87	0.88	0.42	0.14	-0.22	0.03	-0.06	-0.02
DSD-PBEP86[b]	KS	0.3347	0.3187	0.2122	0.2817	0.37	0.10	0.07	0.01	0.04	-0.02	0.03	0.00
	MP2	0.6971	0.8213	0.8807	0.9295	0.72	0.28	0.14	0.06	-0.17	-0.04	0.08	0.03
	KS+MP2	0.5461	0.6467	0.7284	0.7951	1.37	0.71	0.49	0.18	0.08	0.15	0.12	0.03
revDSD-PBEP86-D3BJ[b]	KS	0.3349	0.3189	0.2124	0.2810	0.37	0.10	0.07	0.01	0.04	-0.02	0.03	0.00

Functional	Component	*f*				MAD				MSD[a]			
		**{D,T}**	**{T,Q}**	**{Q,5}**	**{5,6}**	**{D,T}**	**{T,Q}**	**{Q,5}**	**{5,6}**	**{D,T}**	**{T,Q}**	**{Q,5}**	**{5,6}**
	MP2	0.7222	0.8395	0.8944	0.9446	0.78	0.30	0.15	0.07	-0.16	-0.06	0.09	0.03
	KS+MP2	0.5632	0.6625	0.7464	0.8142	1.46	0.74	0.51	0.19	0.09	0.16	0.14	0.03
revDSD-PBEP86-D4[b]	KS	0.3351	0.3190	0.2125	0.2799	0.37	0.10	0.07	0.01	0.04	-0.02	0.03	0.00
	MP2	0.7248	0.8416	0.8963	0.9463	0.79	0.30	0.15	0.07	-0.16	-0.06	0.10	0.03
	KS+MP2	0.5651	0.6641	0.7480	0.8161	1.47	0.74	0.52	0.19	0.10	0.17	0.15	0.03

[a] Defined as $E_{CBS}^{i} - E_{CBS}^{\{Q,5,6\}\zeta}$. [b] The associated dispersion corrections were not included in the basis set extrapolations since they are solely dependent on the geometry.

Table S20. Schwenke *f* extrapolation parameters for two basis-set extrapolations and associated mean absolute and signed deviations (MAD and MSD, respectively, in kcal/mol) for Neese and Valeev's aug-ANO-pV*n*Z for the Kohn–Sham, MP2-like correlation and total double-hybrid DFT energy for selected double-hybrid functionals.

Functional	Component	*f*			MAD			MSD[a]		
		{D,T}	{T,Q}	{Q,5}	{D,T}	{T,Q}	{Q,5}	{D,T}	{T,Q}	{Q,5}
B2PLYP	KS	0.5620	0.8175	1.2289	0.53	0.13	0.12	0.03	0.02	9.84
	MP2	0.6471	0.7924	1.1424	0.26	0.08	0.24	-0.04	0.04	0.14
	KS+MP2	0.6137	0.7674	1.1209	0.58	0.19	0.40	-0.09	0.01	10.04
B2GP-PLYP	KS	0.4408	0.7748	1.3354	0.42	0.12	0.12	0.08	0.03	9.85
	MP2	0.6554	0.8009	1.1399	0.34	0.10	0.32	-0.04	0.05	0.19
	KS+MP2	0.6014	0.7677	1.1998	0.55	0.23	0.54	-0.09	0.04	10.08
mPW2PLYP	KS	0.6801	0.8401	1.1186	0.62	0.14	0.14	-0.02	0.00	9.83
	MP2	0.6447	0.7890	1.1021	0.24	0.07	0.23	-0.05	0.04	0.14
	KS+MP2	0.6383	0.7669	1.0829	0.64	0.19	0.36	-0.10	0.00	10.02
PWPB95	KS	0.5736	0.7434	1.0311	0.52	0.10	0.12	0.04	0.02	9.79
	MP2	0.7117	0.8346	1.1511	0.61	0.10	0.46	-0.15	0.01	0.25
	KS+MP2	0.6678	0.7897	1.1803	0.75	0.26	0.76	0.04	0.06	10.10
DSD-PBEP86[b]	KS	0.3786	0.7354	1.6498	0.34	0.11	0.15	0.06	0.03	9.82
	MP2	0.6966	0.8246	1.1631	0.69	0.17	0.57	-0.15	0.07	0.32
	KS+MP2	0.6365	0.8005	1.2658	0.80	0.36	1.04	0.03	0.07	10.24
revDSD-PBEP86-D3BJ[b]	KS	0.3791	0.7357	-1.0018	0.34	0.11	0.44	0.06	0.03	10.18

Functional	Component	*f*			MAD			MSD[a]		
		{D,T}	{T,Q}	{Q,5}	{D,T}	{T,Q}	{Q,5}	{D,T}	{T,Q}	{Q,5}
	MP2	0.7169	0.8368	1.1773	0.76	0.17	0.62	-0.17	0.07	0.34
	KS+MP2	0.6541	0.8107	-1.0212	0.86	0.38	5.82	0.04	0.08	15.60
revDSD-PBEP86-D4[b]	KS	0.3796	0.7356	1.6402	0.34	0.11	0.15	0.06	0.03	9.81
	MP2	0.7191	0.8381	1.1789	0.77	0.17	0.63	-0.17	0.08	0.35
	KS+MP2	0.6557	0.8119	1.2763	0.87	0.39	1.12	0.05	0.09	10.29

[a] Defined as $E_{CBS}^{i} - E_{CBS}^{\{Q,5,6\}\zeta}$. [b] The associated dispersion corrections were not included in the basis set extrapolations since they are solely dependent on the geometry.

Table S21. Schwenke *f* extrapolation parameters for two basis-set extrapolations and associated mean absolute and signed deviations (MAD and MSD, respectively, in kcal/mol) for Neese and Valeev's ANO-pV*n*Z for the Kohn–Sham, MP2-like correlation and total double-hybrid DFT energy for selected double-hybrid functionals.

Functional	Component	*f*			MAD			MSD[a]		
		{D,T}	{T,Q}	{Q,5}	{D,T}	{T,Q}	{Q,5}	{D,T}	{T,Q}	{Q,5}
B2PLYP	KS	0.4472	1.4406	1.0356	0.64	0.57	0.15	0.07	-0.12	9.85
	MP2	0.6869	0.7679	0.9409	0.37	0.10	0.12	-0.13	0.02	0.09
	KS+MP2	0.5847	0.9061	0.9362	0.76	0.64	0.28	-0.03	-0.13	9.97
B2GP-PLYP	KS	0.3886	1.1946	0.9767	0.49	0.47	0.14	0.07	-0.05	9.84
	MP2	0.6981	0.7791	0.9442	0.49	0.14	0.15	-0.16	0.03	0.12
	KS+MP2	0.5847	0.8647	0.9325	0.71	0.59	0.32	0.02	-0.19	9.99
mPW2PLYP	KS	0.4846	1.6484	1.0579	0.79	0.66	0.17	0.11	-0.14	9.82
	MP2	0.6797	0.7621	0.9393	0.35	0.10	0.11	-0.12	0.02	0.08
	KS+MP2	0.5852	0.9683	0.9492	0.88	0.72	0.28	0.07	-0.16	9.93
PWPB95	KS	0.4442	1.5527	0.9732	0.66	0.60	0.12	0.11	-0.07	9.79
	MP2	0.7465	0.8185	0.9873	0.81	0.19	0.15	-0.21	-0.01	0.08
	KS+MP2	0.6713	0.8810	0.9327	1.06	0.75	0.37	0.00	-0.06	10.04
DSD-PBEP86[b]	KS	0.3438	1.0643	0.9354	0.42	0.38	0.12	0.10	-0.05	9.78
	MP2	0.7396	0.8101	0.9733	0.94	0.25	0.23	-0.31	0.03	0.16
	KS+MP2	0.6459	0.8343	0.9550	0.99	0.69	0.50	0.08	-0.09	10.09
revDSD-PBEP86-D3BJ[b]	KS	0.3443	1.0674	0.9340	0.42	0.38	0.12	0.10	-0.05	9.78

Functional	Component	*f*			MAD			MSD[a]		
		{D,T}	{T,Q}	{Q,5}	{D,T}	{T,Q}	{Q,5}	{D,T}	{T,Q}	{Q,5}
	MP2	0.7592	0.8249	0.9882	1.00	0.27	0.25	-0.33	0.02	0.17
	KS+MP2	0.6625	0.8503	0.9680	1.06	0.72	0.53	0.11	-0.13	10.12
revDSD-PBEP86-D4[b]	KS	0.3446	1.0699	0.9331	0.42	0.38	0.12	0.10	-0.05	9.77
	MP2	0.7614	0.8266	0.9899	1.01	0.27	0.26	-0.33	0.02	0.18
	KS+MP2	0.6647	0.8526	0.9693	1.07	0.72	0.53	0.10	-0.14	10.12

[a] Defined as $E_{CBS}^{i} - E_{CBS}^{\{Q,5,6\}\zeta}$. [b] The associated dispersion corrections were not included in the basis set extrapolations since they are solely dependent on the geometry.

Table S22. Schwenke *f* extrapolation parameters for two basis-set extrapolations and associated mean absolute and signed deviations (MAD and MSD, respectively, in kcal/mol) for the def2 family of basis sets including diffuse functions (*i.e.*, def2D) for the Kohn–Sham, MP2-like correlation and total DFT energy for selected double-hybrid functionals.

**Functional**	**Component**	***f***		**MAD**		**MSD**[a]	
		**{SVPD, TZVPPD}**	**{TZVPPD, QZVPPD}**	**{SVPD, TZVPPD}**	**{TZVPPD, QZVPPD}**	**{SVPD, TZVPPD}**	**{TZVPPD, QZVPPD}**
B2PLYP	KS	0.0759	0.0362	1.38	0.20	-0.42	0.12
	MP2	0.7525	0.7842	0.23	0.11	0.00	-0.01
	KS+MP2	0.1266	0.2807	0.83	1.00	-0.23	0.18
B2GP-PLYP	KS	0.0739	0.0425	1.39	0.18	-0.43	0.10
	MP2	0.7722	0.7936	0.31	0.14	-0.04	-0.02
	KS+MP2	0.1403	0.3433	0.93	1.12	-0.28	0.15
mPW2PLYP	KS	0.0777	0.0342	1.37	0.22	-0.42	0.12
	MP2	0.7394	0.7772	0.22	0.11	0.02	-0.01
	KS+MP2	0.1245	0.2605	0.82	0.99	-0.19	0.19
PWPB95	KS	0.0748	0.0277	1.33	0.19	-0.40	0.11
	MP2	0.8461	0.8281	0.58	0.25	-0.09	-0.05
	KS+MP2	0.2145	0.4948	5.35	1.94	-2.94	-0.26
DSD-PBEP86[b]	KS	0.0724	0.0466	1.37	0.16	-0.40	0.09
	MP2	0.8193	0.8273	0.64	0.27	-0.03	-0.07
	KS+MP2	0.2023	0.5025	3.48	1.63	-0.81	0.10

Functional	Component	*f*		MAD		MSD[a]	
		{SVPD, TZVPPD}	{TZVPPD, QZVPPD}	{SVPD, TZVPPD}	{TZVPPD, QZVPPD}	{SVPD, TZVPPD}	{TZVPPD, QZVPPD}
revDSD-PBEP86-D3BJ[b]	KS	0.0724	0.0466	1.37	0.16	-0.40	0.09
	MP2	0.8422	0.8455	0.69	0.29	-0.01	-0.09
	KS+MP2	0.2089	0.5223	3.61	1.68	-0.72	0.09
revDSD-PBEP86-D4[b]	KS	0.0724	0.0466	1.37	0.17	-0.40	0.09
	MP2	0.8448	0.8476	0.70	0.29	-0.01	-0.09
	KS+MP2	0.2095	0.5241	3.58	1.68	-0.66	0.10

[a] Defined as $E_{CBS}^{i} - E_{CBS}^{\{Q,5,6\}\zeta}$. [b] The associated dispersion corrections were not included in the basis set extrapolations since they are solely dependent on the geometry.

Table S23. Schwenke *f* extrapolation parameters for two basis-set extrapolations and associated mean absolute and signed deviations (MAD and MSD, respectively, in kcal/mol) for the def2 family of basis sets without diffuse functions (*i.e.*, def2D) for the Kohn–Sham, MP2-like correlation and total DFT energy for selected double-hybrid functionals.

Functional	Component	*f*		MAD		MSD[a]	
		{SVP, TZVPP}	{TZVPP, QZVPP}	{SVP, TZVPP}	{TZVPP, QZVPP}	{SVP, TZVPP}	{TZVPP, QZVPP}
B2PLYP	KS	0.0766	0.0471	1.40	0.26	-0.36	0.13
	MP2	0.6213	0.7550	0.34	0.11	0.06	-0.04
	KS+MP2	0.1269	0.2831	0.88	1.06	-0.27	0.15
B2GP-PLYP	KS	0.0744	0.0525	1.41	0.23	-0.36	0.11
	MP2	0.6375	0.7653	0.41	0.14	0.04	-0.04
	KS+MP2	0.1405	0.3488	0.94	1.16	-0.39	0.03
mPW2PLYP	KS	0.0789	0.0445	1.39	0.29	-0.37	0.16
	MP2	0.6106	0.7474	0.34	0.10	0.08	-0.03
	KS+MP2	0.1258	0.2599	0.88	1.06	-0.28	0.22
PWPB95	KS	0.0761	0.0396	1.34	0.25	-0.38	0.13
	MP2	0.7146	0.7937	0.74	0.25	-0.09	-0.04
	KS+MP2	0.2106	0.4754	5.35	1.98	-3.06	-0.13
DSD-PBEP86[b]	KS	0.0735	0.0559	1.39	0.21	-0.40	0.10
	MP2	0.6921	0.7954	0.78	0.26	-0.11	-0.09
	KS+MP2	0.1972	0.4825	3.48	1.67	-0.74	0.21

Functional	Component	f		MAD		MSD[a]	
		{SVP, TZVPP}	{TZVPP, QZVPP}	{SVP, TZVPP}	{TZVPP, QZVPP}	{SVP, TZVPP}	{TZVPP, QZVPP}
revDSD-PBEP86-D3BJ[b]	KS	0.0737	0.0559	4.04	2.94	2.29	2.79
	MP2	0.7218	0.8127	0.82	0.28	-0.21	-0.09
	KS+MP2	0.2049	0.5019	6.85	4.95	2.38	3.40
revDSD-PBEP86-D4[b]	KS	0.0736	0.0559	1.39	0.21	-0.40	0.10
	MP2	0.7253	0.8147	0.83	0.28	-0.22	-0.09
	KS+MP2	0.2054	0.5038	3.57	1.72	-0.77	0.22

[a] Defined as $E_{CBS}^{i} - E_{CBS}^{\{Q,5,6\}\zeta}$. [b] The associated dispersion corrections were not included in the basis set extrapolations since they are solely dependent on the geometry.

Table S24. Schwenke *f* extrapolation parameters for two basis-set extrapolations and associated mean absolute and signed deviations (MAD and MSD, respectively, in kcal/mol) for Jensen's aug-pc-*n* basis sets for the Kohn–Sham, MP2-like correlation and total double-hybrid DFT energy for selected double-hybrid functionals.

Functional	Component	f			MAD			MSD[a]		
		{1,2}	{2,3}	{3,4}	{1,2}	{2,3}	{3,4}	{1,2}	{2,3}	{3,4}
B2PLYP	KS	0.1278	0.2876	2.9448	1.69	0.50	0.46	0.85	0.00	-0.02
	MP2	0.9221	0.5493	0.9214	0.47	0.30	0.12	-0.01	0.14	0.05
	KS+MP2	0.2958	0.4382	1.1462	2.69	0.84	0.63	1.22	0.14	0.04
B2GP-PLYP	KS	0.1413	0.4124	4.1227	2.12	0.84	0.79	1.09	0.04	-0.01
	MP2	0.9381	0.5550	0.9270	0.62	0.40	0.15	-0.01	0.20	0.07

	KS+MP2	0.3505	0.5063	1.3004	3.32	1.36	1.05	1.26	0.24	0.07
mPW2PLYP	KS	0.1195	0.1812	1.7892	1.42	0.30	0.26	0.68	0.05	0.02
	MP2	0.9117	0.5430	0.9128	0.43	0.28	0.11	-0.02	0.12	0.05
	KS+MP2	0.2811	0.3928	0.9749	2.40	0.60	0.40	1.05	0.12	0.05
PWPB95	KS	0.1285	0.2718	2.3666	1.71	0.51	0.40	0.92	0.01	-0.02
	MP2	1.0049	0.6098	0.9667	1.08	0.62	0.24	0.28	0.22	0.08
	KS+MP2	0.4972	0.5532	1.0630	5.17	1.47	0.78	-0.11	0.01	-0.01
DSD-PBEP86[b]	KS	0.1573	0.5293	4.7062	2.55	1.17	1.15	1.28	0.05	0.06
	MP2	0.9950	0.5899	0.9603	1.17	0.74	0.28	0.01	0.36	0.11
	KS+MP2	0.4991	0.5876	1.2601	5.06	2.34	1.65	1.17	0.48	0.15
revDSD-PBEP86-D3BJ[b]	KS	0.1570	0.5281	4.7023	2.55	1.17	1.14	1.28	0.05	0.06
	MP2	1.0194	0.6077	0.9766	1.26	0.79	0.30	0.01	0.38	0.12
	KS+MP2	0.5083	0.6034	1.2527	5.20	2.41	1.68	1.49	0.48	0.17
revDSD-PBEP86-D4[b]	KS	0.1569	0.5272	4.6990	2.54	1.17	1.14	1.28	0.05	0.06
	MP2	1.0226	0.6097	0.9785	1.28	0.80	0.30	0.00	0.39	0.12
	KS+MP2	0.5096	0.6050	1.2515	5.20	2.41	1.68	1.53	0.48	0.17

[a] Defined as $E_{CBS}^{i} - E_{CBS}^{\{Q,5,6\}\zeta}$. [b] The associated dispersion corrections were not included in the basis set extrapolations since they are solely dependent on the geometry.

Table S25. Schwenke *f* extrapolation parameters for two basis-set extrapolations and associated mean absolute and signed deviations (MAD and MSD, respectively, in kcal/mol) for Jensen's pc-*n* basis sets for the Kohn–Sham, MP2-like correlation and total double-hybrid DFT energy for selected double-hybrid functionals.

Functional	Component	*f*			MAD			MSD[a]		
		{1,2}	{2,3}	{3,4}	{1,2}	{2,3}	{3,4}	{1,2}	{2,3}	{3,4}
B2PLYP	KS	0.1433	0.2464	2.6467	2.30	0.47	0.43	1.14	-0.02	-0.10
	MP2	0.9316	0.4850	0.8881	1.00	0.22	0.09	0.16	0.12	0.04
	KS+MP2	0.3166	0.3922	1.1071	3.96	0.70	0.57	1.67	0.01	-0.05
B2GP-PLYP	KS	0.1572	0.3431	3.6917	2.77	0.77	0.74	1.38	0.02	-0.13
	MP2	0.9446	0.4952	0.8961	1.26	0.29	0.12	0.22	0.17	0.06
	KS+MP2	0.3623	0.4505	1.2631	4.75	1.13	0.97	2.09	0.10	-0.05
mPW2PLYP	KS	0.1355	0.1730	1.5191	2.02	0.28	0.25	0.95	-0.01	-0.01
	MP2	0.9196	0.4782	0.8769	0.97	0.21	0.08	0.17	0.11	0.04
	KS+MP2	0.3008	0.3457	0.9413	3.67	0.50	0.35	1.55	0.08	-0.03
PWPB95	KS	0.1436	0.2341	2.2617	2.32	0.48	0.38	1.21	-0.01	-0.13
	MP2	1.0129	0.5363	0.9464	1.95	0.46	0.17	0.57	0.23	0.03
	KS+MP2	0.5068	0.4904	1.0134	6.97	1.19	0.69	0.74	-0.01	-0.03
DSD-PBEP86[b]	KS	0.1712	0.4517	4.3710	3.24	1.08	1.07	1.65	-0.05	-0.13
	MP2	0.9980	0.5284	0.9439	2.23	0.53	0.21	0.51	0.30	0.05
	KS+MP2	0.5074	0.5357	1.2339	7.06	1.91	1.53	2.31	0.10	-0.04

revDSD-PBEP86-D3BJ[b]	KS	0.1711	0.4505	4.3623	3.23	1.07	1.06	1.65	-0.05	-0.13
	MP2	1.0231	0.5456	0.9573	2.31	0.58	0.23	0.45	0.33	0.07
	KS+MP2	0.5219	0.5478	1.2277	7.19	1.97	1.55	2.30	0.17	-0.01
revDSD-PBEP86-D4[b]	KS	0.1710	0.4495	4.3550	3.23	1.07	1.06	1.65	-0.05	-0.13
	MP2	1.0261	0.5475	0.9588	2.33	0.58	0.23	0.44	0.34	0.07
	KS+MP2	0.5236	0.5490	1.2265	7.19	1.98	1.55	2.32	0.19	-0.01

[a] Defined as $E_{CBS}^{i} - E_{CBS}^{\{Q,5,6\}\zeta}$. [b] The associated dispersion corrections were not included in the basis set extrapolations since they are solely dependent on the geometry.

Table S26. Schwenke *f* extrapolation parameters for two basis-set extrapolations and corresponding α, β and γ parameters for wavefunction theory (WFT) methods for the various basis sets and double hybrid DFT functionals considered in this study.

	Component	CBS pair	*f*	α	β	γ
			aug-cc-pV*n*Z			
B2PLYP	KS	{D,T}	0.3727	4.1020	3.2155	1.3038
		{T,Q}	0.3784	4.8244	4.4934	1.2927
		{Q,5}	0.1955	7.6711	8.1154	1.8109
		{5,6}	0.2119	8.1709	9.5647	1.7438
	MP2	{D,T}	0.6263	3.0023	2.3535	0.9542
		{T,Q}	0.7383	3.1958	2.9766	0.8563
		{Q,5}	0.7883	3.4700	3.6709	0.8191
		{5,6}	0.7903	3.8314	4.4849	0.8177
	KS+MP2	{D,T}	0.4714	3.5812	2.8072	1.1382
		{T,Q}	0.5410	3.9068	3.6388	1.0468
		{Q,5}	0.4606	4.8890	5.1722	1.1541
		{5,6}	0.4625	5.3943	6.3145	1.1513
B2GP-PLYP	KS	{D,T}	0.3607	4.1774	3.2746	1.3277
		{T,Q}	0.3297	5.2045	4.8475	1.3945
		{Q,5}	0.1907	7.7596	8.2091	1.8318
		{5,6}	0.2265	7.9151	9.2653	1.6893
	MP2	{D,T}	0.6387	2.9645	2.3238	0.9422
		{T,Q}	0.7433	3.1812	2.9630	0.8524
		{Q,5}	0.7961	3.4467	3.6463	0.8137
		{5,6}	0.7961	3.8124	4.4628	0.8137
	KS+MP2	{D,T}	0.4800	3.5429	2.7772	1.1261
		{T,Q}	0.5361	3.9288	3.6593	1.0527
		{Q,5}	0.5226	4.5297	4.7920	1.0693
		{5,6}	0.5282	4.9777	5.8268	1.0624
mPW2PLYP	KS	{D,T}	0.3852	4.0266	3.1563	1.2798
		{T,Q}	0.4134	4.5879	4.2732	1.2293
		{Q,5}	0.1941	7.6960	8.1417	1.8168
		{5,6}	0.1952	8.4912	9.9396	1.8122
	MP2	{D,T}	0.6180	3.0282	2.3737	0.9625
		{T,Q}	0.7351	3.2051	2.9853	0.8588

		{Q,5}	0.7837	3.4837	3.6855	0.8224
		{5,6}	0.7886	3.8372	4.4918	0.8190
	KS+MP2	{D,T}	0.4709	3.5837	2.8092	1.1390
		{T,Q}	0.5523	3.8565	3.5920	1.0333
		{Q,5}	0.4192	5.1656	5.4648	1.2194
		{5,6}	0.4342	5.5989	6.5540	1.1949
PWPB95	KS	{D,T}	0.3829	4.0403	3.1671	1.2842
		{T,Q}	0.3778	4.8285	4.4973	1.2938
		{Q,5}	0.1867	7.8340	8.2878	1.8494
		{5,6}	0.1901	8.5941	10.0601	1.8342
	MP2	{D,T}	0.7035	2.7825	2.1811	0.8844
		{T,Q}	0.7777	3.0854	2.8738	0.8267
		{Q,5}	0.8239	3.3663	3.5613	0.7947
		{5,6}	0.8239	3.7235	4.3587	0.7947
	KS+MP2	{D,T}	0.5804	3.1516	2.4705	1.0017
		{T,Q}	0.6499	3.4770	3.2385	0.9317
		{Q,5}	0.6502	3.9452	4.1737	0.9313
		{5,6}	0.6576	4.3321	5.0711	0.9246
DSD-PBEP86	KS	{D,T}	0.3528	4.2289	3.3149	1.3441
		{T,Q}	0.3028	5.4457	5.0721	1.4592
		{Q,5}	0.1870	7.8279	8.2813	1.8479
		{5,6}	0.2388	7.7144	9.0303	1.6464
	MP2	{D,T}	0.6902	2.8178	2.2088	0.8956
		{T,Q}	0.7705	3.1050	2.8920	0.8320
		{Q,5}	0.8215	3.3731	3.5685	0.7963
		{5,6}	0.8215	3.7310	4.3674	0.7963
	KS+MP2	{D,T}	0.5574	3.2329	2.5342	1.0275
		{T,Q}	0.6225	3.5754	3.3301	0.9580
		{Q,5}	0.6637	3.8927	4.1182	0.9189
		{5,6}	0.6751	4.2580	4.9844	0.9088
revDSD-PBEP86-D3BJ	KS	{D,T}	0.3529	4.2282	3.3144	1.3439
		{T,Q}	0.3032	5.4425	5.0691	1.4583
		{Q,5}	0.1869	7.8297	8.2832	1.8484
		{5,6}	0.2388	7.7134	9.0292	1.6462

	MP2	{D,T}	0.7146	2.7538	2.1586	0.8752
		{T,Q}	0.7849	3.0660	2.8557	0.8215
		{Q,5}	0.8396	3.3227	3.5151	0.7844
		{5,6}	0.8400	3.6740	4.3007	0.7841
	KS+MP2	{D,T}	0.5733	3.1761	2.4897	1.0095
		{T,Q}	0.6376	3.5205	3.2790	0.9433
		{Q,5}	0.6820	3.8240	4.0455	0.9027
		{5,6}	0.6938	4.1822	4.8956	0.8926
revDSD-PBEP86-D4	KS	{D,T}	0.3529	4.2277	3.3140	1.3437
		{T,Q}	0.3033	5.4407	5.0675	1.4578
		{Q,5}	0.1866	7.8368	8.2907	1.8500
		{5,6}	0.2390	7.7107	9.0260	1.6456
	MP2	{D,T}	0.7175	2.7463	2.1528	0.8729
		{T,Q}	0.7866	3.0616	2.8516	0.8204
		{Q,5}	0.8404	3.3204	3.5127	0.7838
		{5,6}	0.8407	3.6720	4.2983	0.7837
	KS+MP2	{D,T}	0.5752	3.1696	2.4846	1.0074
		{T,Q}	0.6391	3.5149	3.2738	0.9418
		{Q,5}	0.6838	3.8171	4.0382	0.9011
		{5,6}	0.6957	4.1745	4.8865	0.8909
			cc-pV*n*Z			
B2PLYP	KS	{D,T}	0.3520	4.2343	3.3192	1.3458
		{T,Q}	0.3807	4.8080	4.4783	1.2883
		{Q,5}	0.2298	7.1054	7.5169	1.6774
		{5,6}	0.2607	7.3847	8.6443	1.5760
	MP2	{D,T}	0.6329	2.9821	2.3376	0.9478
		{T,Q}	0.7745	3.0940	2.8818	0.8290
		{Q,5}	0.8371	3.3296	3.5224	0.7860
		{5,6}	0.8917	3.5241	4.1253	0.7521
	KS+MP2	{D,T}	0.4538	3.6630	2.8714	1.1643
		{T,Q}	0.5426	3.8996	3.6321	1.0449
		{Q,5}	0.5044	4.6289	4.8970	1.0927
		{5,6}	0.5724	4.7347	5.5424	1.0105
B2GP-PLYP	KS	{D,T}	0.3415	4.3048	3.3745	1.3682
		{T,Q}	0.3417	5.1042	4.7541	1.3677

		{Q,5}	0.2141	7.3513	7.7771	1.7354
		{5,6}	0.2622	7.3627	8.6186	1.5714
	MP2	{D,T}	0.6458	2.9432	2.3071	0.9355
		{T,Q}	0.7838	3.0690	2.8585	0.8223
		{Q,5}	0.8507	3.2926	3.4833	0.7773
		{5,6}	0.9009	3.4987	4.0955	0.7467
	KS+MP2	{D,T}	0.4710	3.5833	2.8089	1.1389
		{T,Q}	0.5562	3.8397	3.5763	1.0288
		{Q,5}	0.5694	4.2949	4.5437	1.0139
		{5,6}	0.6535	4.3497	5.0917	0.9283
mPW2PLYP	KS	{D,T}	0.3627	4.1647	3.2647	1.3237
		{T,Q}	0.4151	4.5772	4.2632	1.2264
		{Q,5}	0.2274	7.1413	7.5549	1.6858
		{5,6}	0.2549	7.4682	8.7421	1.5939
	MP2	{D,T}	0.6248	3.0070	2.3572	0.9557
		{T,Q}	0.7712	3.1030	2.8901	0.8314
		{Q,5}	0.8292	3.3514	3.5455	0.7912
		{5,6}	0.8847	3.5435	4.1479	0.7563
	KS+MP2	{D,T}	0.4541	3.6619	2.8705	1.1639
		{T,Q}	0.5525	3.8560	3.5915	1.0332
		{Q,5}	0.4757	4.7955	5.0732	1.1321
		{5,6}	0.5382	4.9202	5.7595	1.0501
PWPB95	KS	{D,T}	0.3617	4.1706	3.2693	1.3256
		{T,Q}	0.3819	4.7999	4.4707	1.2861
		{Q,5}	0.2186	7.2783	7.6999	1.7182
		{5,6}	0.2600	7.3941	8.6554	1.5781
	MP2	{D,T}	0.7136	2.7563	2.1606	0.8760
		{T,Q}	0.8327	2.9442	2.7422	0.7889
		{Q,5}	0.8888	3.1933	3.3783	0.7538
		{5,6}	0.9333	3.4124	3.9945	0.7283
	KS+MP2	{D,T}	0.5672	3.1977	2.5067	1.0164
		{T,Q}	0.6718	3.4026	3.1692	0.9117
		{Q,5}	0.7083	3.7294	3.9454	0.8804
		{5,6}	0.7636	3.9222	4.5912	0.8371

DSD-PBEP86	KS	{D,T}	0.3347	4.3523	3.4117	1.3833
		{T,Q}	0.3187	5.3000	4.9365	1.4201
		{Q,5}	0.2122	7.3822	7.8097	1.7427
		{5,6}	0.2817	7.0997	8.3107	1.5152
	MP2	{D,T}	0.6971	2.7994	2.1944	0.8898
		{T,Q}	0.8213	2.9723	2.7685	0.7964
		{Q,5}	0.8807	3.2139	3.4001	0.7587
		{5,6}	0.9295	3.4221	4.0058	0.7303
	KS+MP2	{D,T}	0.5461	3.2743	2.5667	1.0407
		{T,Q}	0.6467	3.4880	3.2487	0.9346
		{Q,5}	0.7284	3.6603	3.8723	0.8641
		{5,6}	0.7951	3.8156	4.4665	0.8143
revDSD-PBEP86-D3BJ	KS	{D,T}	0.3349	4.3507	3.4105	1.3828
		{T,Q}	0.3189	5.2986	4.9352	1.4198
		{Q,5}	0.2124	7.3785	7.8058	1.7418
		{5,6}	0.2810	7.1084	8.3210	1.5171
	MP2	{D,T}	0.7222	2.7342	2.1433	0.8690
		{T,Q}	0.8395	2.9275	2.7267	0.7844
		{Q,5}	0.8944	3.1792	3.3634	0.7505
		{5,6}	0.9446	3.3833	3.9604	0.7221
	KS+MP2	{D,T}	0.5632	3.2119	2.5178	1.0209
		{T,Q}	0.6625	3.4336	3.1981	0.9200
		{Q,5}	0.7464	3.6010	3.8095	0.8501
		{5,6}	0.8142	3.7542	4.3946	0.8012
revDSD-PBEP86-D4	KS	{D,T}	0.3351	4.3494	3.4095	1.3824
		{T,Q}	0.3190	5.2973	4.9339	1.4194
		{Q,5}	0.2125	7.3779	7.8052	1.7417
		{5,6}	0.2799	7.1221	8.3370	1.5200
	MP2	{D,T}	0.7248	2.7278	2.1382	0.8670
		{T,Q}	0.8416	2.9224	2.7220	0.7831
		{Q,5}	0.8963	3.1744	3.3583	0.7494
		{5,6}	0.9463	3.3790	3.9554	0.7212
	KS+MP2	{D,T}	0.5651	3.2053	2.5125	1.0188
		{T,Q}	0.6641	3.4283	3.1932	0.9186
		{Q,5}	0.7480	3.5956	3.8038	0.8488

		{5,6}	0.8161	3.7481	4.3874	0.7999
		aug-ANO-pV*n*Z				
B2PLYP	KS	{D,T}	0.5620	3.2162	2.5212	1.0222
		{T,Q}	0.8175	2.9818	2.7773	0.7990
		{Q,5}	1.2289	2.5221	2.6681	0.5954
	MP2	{D,T}	0.6471	2.9394	2.3041	0.9342
		{T,Q}	0.7924	3.0462	2.8373	0.8162
		{Q,5}	1.1424	2.6637	2.8179	0.6288
	KS+MP2	{D,T}	0.6137	3.0418	2.3844	0.9668
		{T,Q}	0.7674	3.1134	2.8998	0.8342
		{Q,5}	1.1209	2.7013	2.8578	0.6377
B2GP-PLYP	KS	{D,T}	0.4408	3.7262	2.9209	1.1843
		{T,Q}	0.7748	3.0933	2.8811	0.8288
		{Q,5}	1.3354	2.3677	2.5048	0.5589
	MP2	{D,T}	0.6554	2.9151	2.2851	0.9265
		{T,Q}	0.8009	3.0241	2.8167	0.8103
		{Q,5}	1.1399	2.6680	2.8225	0.6298
	KS+MP2	{D,T}	0.6014	3.0815	2.4155	0.9794
		{T,Q}	0.7677	3.1126	2.8991	0.8340
		{Q,5}	1.1998	2.5679	2.7167	0.6062
mPW2PLYP	KS	{D,T}	0.6801	2.8454	2.2304	0.9044
		{T,Q}	0.8401	2.9262	2.7254	0.7841
		{Q,5}	1.1186	2.7055	2.8622	0.6387
	MP2	{D,T}	0.6447	2.9466	2.3098	0.9365
		{T,Q}	0.7890	3.0551	2.8456	0.8186
		{Q,5}	1.1021	2.7353	2.8937	0.6457
	KS+MP2	{D,T}	0.6383	2.9656	2.3247	0.9426
		{T,Q}	0.7669	3.1149	2.9012	0.8346
		{Q,5}	1.0829	2.7709	2.9313	0.6541
PWPB95	KS	{D,T}	0.5736	3.1753	2.4891	1.0092
		{T,Q}	0.7434	3.1811	2.9629	0.8524
		{Q,5}	1.0311	2.8718	3.0381	0.6779
	MP2	{D,T}	0.7117	2.7611	2.1644	0.8776
		{T,Q}	0.8346	2.9396	2.7379	0.7877
		{Q,5}	1.1511	2.6486	2.8020	0.6253

	KS+MP2	{D,T}	0.6678	2.8798	2.2574	0.9153
		{T,Q}	0.7897	3.0534	2.8440	0.8182
		{Q,5}	1.1803	2.5997	2.7502	0.6137
DSD-PBEP86	KS	{D,T}	0.3786	4.0659	3.1872	1.2923
		{T,Q}	0.7354	3.2043	2.9845	0.8586
		{Q,5}	1.6498	2.0072	2.1235	0.4738
	MP2	{D,T}	0.6966	2.8008	2.1955	0.8902
		{T,Q}	0.8246	2.9640	2.7607	0.7942
		{Q,5}	1.1631	2.6283	2.7806	0.6205
	KS+MP2	{D,T}	0.6365	2.9710	2.3290	0.9443
		{T,Q}	0.8005	3.0251	2.8176	0.8106
		{Q,5}	1.2658	2.4663	2.6092	0.5822
revDSD-PBEP86-D3BJ	KS	{D,T}	0.3791	4.0628	3.1848	1.2913
		{T,Q}	0.7357	3.2034	2.9837	0.8584
		{Q,5}	-1.0018	-26.7380	-28.2866	-6.3120
	MP2	{D,T}	0.7169	2.7478	2.1540	0.8734
		{T,Q}	0.8368	2.9342	2.7330	0.7862
		{Q,5}	1.1773	2.6046	2.7555	0.6149
	KS+MP2	{D,T}	0.6541	2.9190	2.2882	0.9278
		{T,Q}	0.8107	2.9991	2.7933	0.8036
		{Q,5}	-1.0212	-16.4128	-17.3634	-3.8745
revDSD-PBEP86-D4	KS	{D,T}	0.3796	4.0601	3.1827	1.2905
		{T,Q}	0.7356	3.2036	2.9839	0.8584
		{Q,5}	1.6402	2.0166	2.1334	0.4760
	MP2	{D,T}	0.7191	2.7422	2.1495	0.8716
		{T,Q}	0.8381	2.9309	2.7298	0.7853
		{Q,5}	1.1789	2.6019	2.7526	0.6142
	KS+MP2	{D,T}	0.6557	2.9142	2.2844	0.9262
		{T,Q}	0.8119	2.9958	2.7903	0.8027
		{Q,5}	1.2763	2.4509	2.5928	0.5786
			ANO-pV*n*Z			
B2PLYP	KS	{D,T}	0.4472	3.6947	2.8962	1.1743
		{T,Q}	1.4406	1.9675	1.8325	0.5272
		{Q,5}	1.0356	2.8629	3.0287	0.6758

	MP2	{D,T}	0.6869	2.8267	2.2158	0.8984
		{T,Q}	0.7679	3.1122	2.8987	0.8339
		{Q,5}	0.9409	3.0673	3.2450	0.7241
	KS+MP2	{D,T}	0.5847	3.1371	2.4591	0.9971
		{T,Q}	0.9061	2.7754	2.5850	0.7437
		{Q,5}	0.9362	3.0782	3.2565	0.7267
B2GP-PLYP	KS	{D,T}	0.3886	4.0067	3.1408	1.2735
		{T,Q}	1.1946	2.2699	2.1142	0.6082
		{Q,5}	0.9767	2.9865	3.1594	0.7050
	MP2	{D,T}	0.6981	2.7967	2.1923	0.8889
		{T,Q}	0.7791	3.0816	2.8702	0.8257
		{Q,5}	0.9442	3.0597	3.2369	0.7223
	KS+MP2	{D,T}	0.5847	3.1368	2.4589	0.9970
		{T,Q}	0.8647	2.8680	2.6713	0.7685
		{Q,5}	0.9325	3.0869	3.2657	0.7287
mPW2PLYP	KS	{D,T}	0.4846	3.5226	2.7613	1.1196
		{T,Q}	1.6484	1.7695	1.6482	0.4741
		{Q,5}	1.0579	2.8187	2.9819	0.6654
	MP2	{D,T}	0.6797	2.8466	2.2314	0.9047
		{T,Q}	0.7621	3.1282	2.9136	0.8382
		{Q,5}	0.9393	3.0709	3.2488	0.7249
	KS+MP2	{D,T}	0.5852	3.1352	2.4576	0.9965
		{T,Q}	0.9683	2.6475	2.4659	0.7094
		{Q,5}	0.9492	3.0481	3.2246	0.7196
PWPB95	KS	{D,T}	0.4442	3.7097	2.9079	1.1791
		{T,Q}	1.5527	1.8554	1.7281	0.4971
		{Q,5}	0.9732	2.9941	3.1675	0.7068
	MP2	{D,T}	0.7465	2.6743	2.0964	0.8500
		{T,Q}	0.8185	2.9793	2.7749	0.7983
		{Q,5}	0.9873	2.9633	3.1349	0.6995
	KS+MP2	{D,T}	0.6713	2.8698	2.2496	0.9121
		{T,Q}	0.8810	2.8309	2.6367	0.7585
		{Q,5}	0.9327	3.0862	3.2650	0.7286
DSD-PBEP86	KS	{D,T}	0.3438	4.2889	3.3620	1.3632
		{T,Q}	1.0643	2.4724	2.3028	0.6625

		{Q,5}	0.9354	3.0799	3.2583	0.7271
	MP2	{D,T}	0.7396	2.6910	2.1094	0.8553
		{T,Q}	0.8101	3.0004	2.7946	0.8040
		{Q,5}	0.9733	2.9940	3.1674	0.7068
	KS+MP2	{D,T}	0.6459	2.9430	2.3070	0.9354
		{T,Q}	0.8343	2.9402	2.7385	0.7878
		{Q,5}	0.9550	3.0348	3.2105	0.7164
revDSD-PBEP86-D3BJ	KS	{D,T}	0.3443	4.2859	3.3596	1.3622
		{T,Q}	1.0674	2.4672	2.2979	0.6611
		{Q,5}	0.9340	3.0834	3.2620	0.7279
	MP2	{D,T}	0.7592	2.6441	2.0726	0.8404
		{T,Q}	0.8249	2.9633	2.7600	0.7940
		{Q,5}	0.9882	2.9613	3.1329	0.6991
	KS+MP2	{D,T}	0.6625	2.8946	2.2690	0.9200
		{T,Q}	0.8503	2.9017	2.7026	0.7775
		{Q,5}	0.9680	3.0056	3.1797	0.7095
revDSD-PBEP86-D4	KS	{D,T}	0.3446	4.2835	3.3578	1.3615
		{T,Q}	1.0699	2.4629	2.2939	0.6599
		{Q,5}	0.9331	3.0854	3.2641	0.7284
	MP2	{D,T}	0.7614	2.6389	2.0686	0.8387
		{T,Q}	0.8266	2.9591	2.7562	0.7929
		{Q,5}	0.9899	2.9577	3.1290	0.6982
	KS+MP2	{D,T}	0.6647	2.8885	2.2643	0.9181
		{T,Q}	0.8526	2.8963	2.6976	0.7761
		{Q,5}	0.9693	3.0028	3.1767	0.7089
def2D						
B2PLYP	KS	{SVP,TZVPP}	0.0759	8.3421	6.5392	2.6514
		{TZVPP,QZVPP}	0.0362	12.5209	11.6621	3.3550
	MP2	{SVP,TZVPP}	0.7525	2.6599	2.0851	0.8454
		{TZVPP,QZVPP}	0.7842	3.0680	2.8575	0.8221
	KS+MP2	{SVP,TZVPP}	0.1266	6.8770	5.3908	2.1858
		{TZVP,QZVP}	0.2807	5.6645	5.2760	1.5178
B2GP-PLYP	KS	{SVP,TZVPP}	0.0739	8.4188	6.5994	2.6758
		{TZVPP,QZVPP}	0.0425	11.9460	11.1266	3.2009
	MP2	{SVP,TZVPP}	0.7722	2.6137	2.0488	0.8307

		{TZVPP,QZVPP}	0.7936	3.0431	2.8343	0.8154
	KS+MP2	{SVP,TZVPP}	0.1403	6.5920	5.1673	2.0952
		{TZVP,QZVP}	0.3433	5.0915	4.7422	1.3643
mPW2PLYP	KS	{SVP,TZVPP}	0.0777	8.2721	6.4844	2.6292
		{TZVPP,QZVPP}	0.0342	12.7251	11.8523	3.4097
	MP2	{SVP,TZVPP}	0.7394	2.6916	2.1099	0.8555
		{TZVPP,QZVPP}	0.7772	3.0868	2.8751	0.8271
	KS+MP2	{SVP,TZVPP}	0.1245	6.9233	5.4271	2.2005
		{TZVP,QZVP}	0.2605	5.8841	5.4805	1.5766
PWPB95	KS	{SVP,TZVPP}	0.0748	8.3849	6.5727	2.6650
		{TZVPP,QZVPP}	0.0277	13.4923	12.5669	3.6153
	MP2	{SVP,TZVPP}	0.8461	2.4547	1.9242	0.7802
		{TZVPP,QZVPP}	0.8281	2.9553	2.7526	0.7919
	KS+MP2	{SVP,TZVPP}	0.2145	5.4545	4.2757	1.7337
		{TZVP,QZVP}	0.4948	4.1259	3.8429	1.1055
DSD-PBEP86	KS	{SVP,TZVPP}	0.0724	8.4820	6.6489	2.6959
		{TZVPP,QZVPP}	0.0466	11.6115	10.8150	3.1113
	MP2	{SVP,TZVPP}	0.8193	2.5100	1.9675	0.7978
		{TZVPP,QZVPP}	0.8273	2.9575	2.7546	0.7925
	KS+MP2	{SVP,TZVPP}	0.2023	5.6073	4.3955	1.7822
		{TZVP,QZVP}	0.5025	4.0876	3.8072	1.0953
revDSD-PBEP86-D3BJ	KS	{SVP,TZVPP}	0.0724	8.4814	6.6484	2.6957
		{TZVPP,QZVPP}	0.0466	11.6137	10.8170	3.1119
	MP2	{SVP,TZVPP}	0.8422	2.4626	1.9304	0.7827
		{TZVPP,QZVPP}	0.8455	2.9131	2.7133	0.7806
	KS+MP2	{SVP,TZVPP}	0.2089	5.5234	4.3297	1.7556
		{TZVP,QZVP}	0.5223	3.9925	3.7186	1.0698
revDSD-PBEP86-D4	KS	{SVP,TZVPP}	0.0724	8.4808	6.6480	2.6955
		{TZVPP,QZVPP}	0.0466	11.6156	10.8189	3.1124
	MP2	{SVP,TZVPP}	0.8448	2.4572	1.9262	0.7810
		{TZVPP,QZVPP}	0.8476	2.9081	2.7086	0.7792
	KS+MP2	{SVP,TZVPP}	0.2095	5.5162	4.3241	1.7533
		{TZVP,QZVP}	0.5241	3.9837	3.7104	1.0674

		def2				
B2PLYP	KS	{SVP,TZVPP}	0.0766	8.3136	6.5169	2.6424
		{TZVPP,QZVPP}	0.0471	11.5756	10.7816	3.1017
	MP2	{SVP,TZVPP}	0.6213	3.0177	2.3655	0.9591
		{TZVPP,QZVPP}	0.7550	3.1480	2.9320	0.8435
	KS+MP2	{SVP,TZVPP}	0.1269	6.8702	5.3854	2.1836
		{TZVP,QZVP}	0.2831	5.6401	5.2532	1.5113
B2GP-PLYP	KS	{SVP,TZVPP}	0.0744	8.3989	6.5837	2.6695
		{TZVPP,QZVPP}	0.0525	11.1869	10.4195	2.9975
	MP2	{SVP,TZVPP}	0.6375	2.9680	2.3266	0.9433
		{TZVPP,QZVPP}	0.7653	3.1193	2.9053	0.8358
	KS+MP2	{SVP,TZVPP}	0.1405	6.5890	5.1650	2.0942
		{TZVP,QZVP}	0.3488	5.0475	4.7013	1.3525
mPW2PLYP	KS	{SVP,TZVPP}	0.0789	8.2303	6.4516	2.6159
		{TZVPP,QZVPP}	0.0445	11.7739	10.9663	3.1548
	MP2	{SVP,TZVPP}	0.6106	3.0516	2.3921	0.9699
		{TZVPP,QZVPP}	0.7474	3.1695	2.9521	0.8493
	KS+MP2	{SVP,TZVPP}	0.1258	6.8945	5.4045	2.1913
		{TZVP,QZVP}	0.2599	5.8909	5.4869	1.5785
PWPB95	KS	{SVP,TZVPP}	0.0761	8.3329	6.5320	2.6485
		{TZVPP,QZVPP}	0.0396	12.1946	11.3582	3.2675
	MP2	{SVP,TZVPP}	0.7146	2.7535	2.1584	0.8752
		{TZVPP,QZVPP}	0.7937	3.0428	2.8341	0.8153
	KS+MP2	{SVP,TZVPP}	0.2106	5.5023	4.3132	1.7488
		{TZVP,QZVP}	0.4754	4.2268	3.9369	1.1326
DSD-PBEP86	KS	{SVP,TZVPP}	0.0735	8.4347	6.6119	2.6809
		{TZVPP,QZVPP}	0.0559	10.9673	10.2150	2.9387
	MP2	{SVP,TZVPP}	0.6921	2.8127	2.2049	0.8940
		{TZVPP,QZVPP}	0.7954	3.0385	2.8301	0.8142
	KS+MP2	{SVP,TZVPP}	0.1972	5.6742	4.4479	1.8035
		{TZVP,QZVP}	0.4825	4.1890	3.9017	1.1224
revDSD-PBEP86-D3BJ	KS	{SVP,TZVPP}	0.0737	8.4288	6.6072	2.6790
		{TZVPP,QZVPP}	0.0559	10.9653	10.2132	2.9382
	MP2	{SVP,TZVPP}	0.7218	2.7353	2.1442	0.8694
		{TZVPP,QZVPP}	0.8127	2.9938	2.7885	0.8022

	KS+MP2	{SVP,TZVPP}	0.2049	5.5746	4.3699	1.7718
		{TZVP,QZVP}	0.5019	4.0906	3.8100	1.0961
revDSD-PBEP86-D4	KS	{SVP,TZVPP}	0.0736	8.4322	6.6099	2.6801
		{TZVPP,QZVPP}	0.0559	10.9641	10.2121	2.9378
	MP2	{SVP,TZVPP}	0.7253	2.7265	2.1373	0.8666
		{TZVPP,QZVPP}	0.8147	2.9888	2.7838	0.8009
	KS+MP2	{SVP,TZVPP}	0.2054	5.5675	4.3642	1.7695
		{TZVP,QZVP}	0.5038	4.0815	3.8015	1.0936
			aug-pc-*n*			
B2PLYP	KS	{1,2}	0.1278	6.8510	5.3704	2.1775
		{2,3}	0.2876	5.5940	5.2103	1.4989
		{3,4}	2.9448	1.2385	1.3102	0.2924
	MP2	{1,2}	0.9221	2.3110	1.8116	0.7345
		{2,3}	0.5493	3.8698	3.6043	1.0369
		{3,4}	0.9214	3.1133	3.2936	0.7349
	KS+MP2	{1,2}	0.2958	4.6477	3.6433	1.4772
		{2,3}	0.4382	4.4357	4.1314	1.1885
		{3,4}	1.1462	2.6571	2.8110	0.6273
B2GP-PLYP	KS	{1,2}	0.1413	6.5722	5.1519	2.0889
		{2,3}	0.4124	4.5941	4.2790	1.2310
		{3,4}	4.1227	0.9200	0.9733	0.2172
	MP2	{1,2}	0.9381	2.2830	1.7896	0.7256
		{2,3}	0.5550	3.8448	3.5811	1.0302
		{3,4}	0.9270	3.0997	3.2793	0.7317
	KS+MP2	{1,2}	0.3505	4.2441	3.3269	1.3489
		{2,3}	0.5063	4.0692	3.7901	1.0903
		{3,4}	1.3004	2.4163	2.5562	0.5704
mPW2PLYP	KS	{1,2}	0.1195	7.0389	5.5177	2.2372
		{2,3}	0.1812	6.9957	6.5159	1.8745
		{3,4}	1.7892	1.8808	1.9897	0.4440
	MP2	{1,2}	0.9117	2.3296	1.8261	0.7404
		{2,3}	0.5430	3.8975	3.6302	1.0443
		{3,4}	0.9128	3.1339	3.3154	0.7398
	KS+MP2	{1,2}	0.2811	4.7722	3.7408	1.5168
		{2,3}	0.3928	4.7242	4.4002	1.2658

		{3,4}	0.9749	2.9904	3.1636	0.7059
PWPB95	KS	{1,2}	0.1285	6.8353	5.3581	2.1725
		{2,3}	0.2718	5.7587	5.3637	1.5430
		{3,4}	2.3666	1.4930	1.5795	0.3524
	MP2	{1,2}	1.0049	2.1732	1.7035	0.6907
		{2,3}	0.6098	3.6227	3.3742	0.9707
		{3,4}	0.9667	3.0085	3.1828	0.7102
	KS+MP2	{1,2}	0.4972	3.4685	2.7189	1.1024
		{2,3}	0.5532	3.8526	3.5883	1.0323
		{3,4}	1.0630	2.8089	2.9715	0.6631
DSD-PBEP86	KS	{1,2}	0.1573	6.2797	4.9225	1.9959
		{2,3}	0.5293	3.9599	3.6883	1.0610
		{3,4}	4.7062	0.8162	0.8635	0.1927
	MP2	{1,2}	0.9950	2.1887	1.7157	0.6957
		{2,3}	0.5899	3.7001	3.4463	0.9914
		{3,4}	0.9603	3.0230	3.1980	0.7136
	KS+MP2	{1,2}	0.4991	3.4603	2.7125	1.0998
		{2,3}	0.5876	3.7093	3.4549	0.9939
		{3,4}	1.2601	2.4748	2.6181	0.5842
revDSD-PBEP86-D3BJ	KS	{1,2}	0.1570	6.2833	4.9254	1.9971
		{2,3}	0.5281	3.9652	3.6932	1.0625
		{3,4}	4.7023	0.8168	0.8641	0.1928
	MP2	{1,2}	1.0194	2.1508	1.6859	0.6836
		{2,3}	0.6077	3.6308	3.3817	0.9729
		{3,4}	0.9766	2.9866	3.1596	0.7050
	KS+MP2	{1,2}	0.5083	3.4222	2.6826	1.0877
		{2,3}	0.6034	3.6473	3.3971	0.9773
		{3,4}	1.2527	2.4858	2.6298	0.5868
revDSD-PBEP86-D4	KS	{1,2}	0.1569	6.2863	4.9277	1.9980
		{2,3}	0.5272	3.9696	3.6973	1.0637
		{3,4}	4.6990	0.8173	0.8646	0.1929
	MP2	{1,2}	1.0226	2.1459	1.6821	0.6821
		{2,3}	0.6097	3.6232	3.3746	0.9708
		{3,4}	0.9785	2.9826	3.1554	0.7041
	KS+MP2	{1,2}	0.5096	3.4166	2.6782	1.0859

		{2,3}	0.6050	3.6411	3.3913	0.9756
		{3,4}	1.2515	2.4877	2.6318	0.5873
			pc-*n*			
B2PLYP	KS	{1,2}	0.1433	6.5347	5.1224	2.0770
		{2,3}	0.2464	6.0497	5.6348	1.6210
		{3,4}	2.6467	1.3577	1.4364	0.3205
	MP2	{1,2}	0.9316	2.2943	1.7985	0.7292
		{2,3}	0.4850	4.1762	3.8897	1.1190
		{3,4}	0.8881	3.1951	3.3801	0.7543
	KS+MP2	{1,2}	0.3166	4.4839	3.5148	1.4251
		{2,3}	0.3922	4.7281	4.4038	1.2669
		{3,4}	1.1071	2.7262	2.8841	0.6436
B2PLYP	KS	{1,2}	0.1572	6.2807	4.9233	1.9962
		{2,3}	0.3431	5.0930	4.7437	1.3647
		{3,4}	3.6917	1.0154	1.0742	0.2397
	MP2	{1,2}	0.9446	2.2717	1.7807	0.7220
		{2,3}	0.4952	4.1244	3.8415	1.1051
		{3,4}	0.8961	3.1750	3.3589	0.7495
	KS+MP2	{1,2}	0.3623	4.1673	3.2667	1.3245
		{2,3}	0.4505	4.3640	4.0647	1.1693
		{3,4}	1.2631	2.4703	2.6134	0.5832
B2PLYP	KS	{1,2}	0.1355	6.6882	5.2428	2.1258
		{2,3}	0.1730	7.1438	6.6538	1.9142
		{3,4}	1.5191	2.1425	2.2666	0.5058
	MP2	{1,2}	0.9196	2.3154	1.8150	0.7359
		{2,3}	0.4782	4.2120	3.9231	1.1286
		{3,4}	0.8769	3.2236	3.4103	0.7610
	KS+MP2	{1,2}	0.3008	4.6068	3.6112	1.4642
		{2,3}	0.3457	5.0722	4.7243	1.3591
		{3,4}	0.9413	3.0662	3.2438	0.7238
PWPB95	KS	{1,2}	0.1436	6.5288	5.1178	2.0751
		{2,3}	0.2341	6.2046	5.7790	1.6625
		{3,4}	2.2617	1.5509	1.6408	0.3661
	MP2	{1,2}	1.0129	2.1607	1.6937	0.6867
		{2,3}	0.5363	3.9278	3.6583	1.0524

		{3,4}	0.9464	3.0545	3.2315	0.7211
	KS+MP2	{1,2}	0.5068	3.4283	2.6874	1.0896
		{2,3}	0.4904	4.1482	3.8637	1.1115
		{3,4}	1.0134	2.9081	3.0766	0.6865
DSD-PBEP86	KS	{1,2}	0.1712	6.0496	4.7422	1.9228
		{2,3}	0.4517	4.3569	4.0580	1.1674
		{3,4}	4.3710	0.8727	0.9233	0.2060
	MP2	{1,2}	0.9980	2.1840	1.7120	0.6942
		{2,3}	0.5284	3.9639	3.6920	1.0621
		{3,4}	0.9439	3.0602	3.2374	0.7224
	KS+MP2	{1,2}	0.5074	3.4257	2.6853	1.0888
		{2,3}	0.5357	3.9303	3.6607	1.0531
		{3,4}	1.2339	2.5143	2.6600	0.5936
revDSD-PBEP86-D3BJ	KS	{1,2}	0.1711	6.0514	4.7436	1.9233
		{2,3}	0.4505	4.3638	4.0645	1.1693
		{3,4}	4.3623	0.8743	0.9249	0.2064
	MP2	{1,2}	1.0231	2.1451	1.6815	0.6818
		{2,3}	0.5456	3.8863	3.6197	1.0413
		{3,4}	0.9573	3.0297	3.2052	0.7152
	KS+MP2	{1,2}	0.5219	3.3671	2.6394	1.0702
		{2,3}	0.5478	3.8762	3.6103	1.0386
		{3,4}	1.2277	2.5240	2.6702	0.5958
revDSD-PBEP86-D4	KS	{1,2}	0.1710	6.0528	4.7447	1.9238
		{2,3}	0.4495	4.3696	4.0698	1.1708
		{3,4}	4.3550	0.8756	0.9263	0.2067
	MP2	{1,2}	1.0261	2.1406	1.6780	0.6804
		{2,3}	0.5475	3.8777	3.6117	1.0390
		{3,4}	0.9588	3.0263	3.2015	0.7144
	KS+MP2	{1,2}	0.5236	3.3607	2.6344	1.0681
		{2,3}	0.5490	3.8713	3.6058	1.0373
		{3,4}	1.2265	2.5258	2.6721	0.5963

S7 The Corrected ANO-cc-pVTZ Basis Set

S7.1 MOLPRO Format

```
! ANO basis sets from atomic MR-ACPF based on cc-pV6Z primitives
!
! H-Al: Neese, F.; Valeev, E.F J. Chem. Theo. Comp., 2010, submitted for publication
! ORIGINAL FILE WAS BOGUS COPY OF aug-pVDZ: corrected Jan 27, 2012 by Gershom Martin
! how: copied ano-pVQZ, stripped off top angular momentum plus last ANO of each remaining angular
momentum
!
spherical
basis={
S,H,402.009900,60.241960,13.732170,3.904505,1.282709,0.465544,0.181120,0.072791
c,1.8,-0.0004429508,-0.0032507467,-0.0181152250,-0.0636329998,-0.2221636410,-0.4041047831,-
0.3481081094,-0.0927213251
c,1.8,-0.0005857917,-0.0020963443,-0.0304299021,-0.0288272025,-0.7526945634,-
0.5303060000,0.9524676196,0.1944104297
c,1.8,-0.0006083150,-0.0052483500,-0.0254171128,-0.2376406403,-1.7096583473,2.6789654303,-
1.0333863147,-0.2486237019
P,H,9.880000,3.950000,1.580000,0.630000,0.250000,0.100000
c,1.6,0.0055312240,0.0053232428,0.1503847471,0.5829443939,0.3399470552,0.0317494051
c,1.6,-0.0280600947,0.0343704220,-0.8940158660,-0.2472687692,0.8487826893,0.0228203559
D,H,4.000000,1.600000,0.640000,0.260000
c,1.4,0.0022011030,-0.2782524806,-0.6650428296,-0.1748476192
S,He,4785.000000,717.000000,163.200000,46.260000,15.100000,5.437000,2.088000,0.829700,0.336600,0.
136900
c,1.10,0.0000603202,0.0004684960,0.0024501467,0.0101553835,0.0350340333,0.0992797341,0.2173871303
,0.3472682167,0.3445578174,0.1180260053
c,1.10,0.0000888731,0.0006940214,0.0036433971,0.0157462582,0.0614009646,0.2414755099,0.6119641323
,0.1961329442,-0.7241642195,-0.3382814814
c,1.10,0.0001287054,0.0011477678,0.0050983801,0.0306322873,0.1396159611,0.8577184034,0.0948064849
,-1.7250507963,0.6542597771,0.5294200911
P,He,0.387000,0.984000,2.498000,6.342000,16.104000
c,1.5,0.2887442887,0.5361342449,0.2659020077,0.0400702083,0.0090385832
c,1.5,-0.6544173279,-0.2752308724,0.7993083208,0.2671653126,0.0310059145
D,He,0.747000,1.910000,4.886000,12.498000
c,1.4,0.4658429191,0.5373435960,0.1309421923,0.0071350958
S,Li,70681.000000,13594.000000,3100.400000,826.460000,253.760000,88.451000,34.493000,14.831000,6.
929900,3.467800,1.831600,1.006300,0.566780,0.322540,0.182770,0.101640,0.054666,0.028025,0.013497
c,1.19,0.0000053236,0.0000323747,0.0001879603,0.0008712219,0.0033836700,0.0110494789,0.0305955979
,0.0707341149,0.1345557163,0.2069615916,0.2591085719,0.2468661567,0.1501162290,0.0448272557,0.003
5755287,0.0003154609,0.0005366220,0.0004574244,0.0000471593
c,1.19,0.0000008577,0.0000052160,0.0000302866,0.0001404588,0.0005463525,0.0017918661,0.0050120074
,0.0118375809,0.0233643106,0.0381867406,0.0536789047,0.0683347487,0.0749471861,0.0592482250,-
0.0260749945,-0.2220432444,-0.4734515860,-0.3620754465,-0.0407343934
c,1.19,0.0000080423,0.0000490032,0.0002842400,0.0013265029,0.0052097294,0.0179882238,0.0571974540
,0.1782581630,0.4298970134,0.4508658275,-0.0371352880,-0.4872120993,-0.4627668553,-0.1601289845,-
0.0116236967,0.0661103743,0.1351321962,0.1047064237,0.0116751457
c,1.19,0.0000113754,0.0000711224,0.0004007287,0.0019573837,0.0076139906,0.0317360413,0.1342152644
,0.5842277718,0.6806090172,-1.0050986104,-
0.9446701880,0.0956130540,0.6974621916,0.3954658405,0.0714809958,-0.0818978750,-0.1598815920,-
0.1282750162,-0.0141083238
P,Li,28.500000,6.640000,1.920000,0.770000,0.320000,0.150000,0.066000,0.025000
c,1.8,0.0189870192,0.3141072757,0.6617276623,0.0886066288,0.1156570486,-
0.0647171322,0.0269408101,-0.0074775714
c,1.8,0.0274718905,0.3090978049,0.3225036171,-0.3787691725,-0.4329780841,-0.2896095144,-
0.1054598002,-0.0091887916
D,Li,1.750000,0.300000,0.110000
c,1.3,1.0034973970,-0.0174235563,0.0214475965
S,Be,139330.000000,26774.000000,6112.000000,1632.900000,503.030000,176.030000,68.939000,29.768000
,13.963000,7.009000,3.709400,2.039200,1.147200,0.650700,0.366590,0.202110,0.107420,0.054231,0.025
619
c,1.19,0.0000048137,0.0000292950,0.0001696534,0.0007837988,0.0030296604,0.0098688962,0.0273158639
,0.0638839975,0.1244755331,0.1997945143,0.2603024986,0.2561166821,0.1602238386,0.0479172446,0.004
0519380,0.0000553253,-0.0000146329,0.0000054228,0.0000000000
c,1.19,0.0000008966,0.0000054566,0.0000316042,0.0001460945,0.0005655948,0.0018504976,0.0051738207
,0.0123588076,0.0250209252,0.0429921324,0.0640053839,0.0837309282,0.0886004427,0.0453339368,-
0.0774503499,-0.2769575079,-0.4532396096,-0.3011584730,-0.0331442139
```

c,1.19,-0.0000013388,-0.0000081449,-0.0000472010,-0.0002181144,-0.0008456379,-0.0027700998,-
0.0078018669,-0.0188623345,-0.0393045895,-0.0686639537,-0.1016445025,-0.1297877375,-
0.1780941635,-0.0475153109,0.7220485366,0.9937821087,-0.3707388758,-0.9022620370,-0.1163652246
c,1.19,-0.0000023477,-0.0000141798,-0.0000829446,-0.0003795818,-0.0014952726,-0.0048605475,-
0.0144204026,-0.0359876745,-0.0871441173,-0.1382935381,-0.1829728831,-0.0817135288,-
0.7363775005,1.7380426984,1.6466592777,-2.5871369088,-0.8177852807,1.2514210442,0.1967978149
P,Be,45.700000,10.600000,3.080000,1.230000,0.510000,0.230000,0.106000,0.040000
c,1.8,0.0005598577,0.0049230936,0.0198821499,0.0394518210,0.1630602437,0.4111578133,0.4429347544,
0.0688676460
c,1.8,0.0008862347,0.0306619409,0.0319215308,0.6318969865,-0.7695651726,-
1.1409907121,1.4919251865,0.0091265087
D,Be,3.350000,0.570000,0.210000
c,1.3,0.0250099508,-0.0916355551,-0.9394094692
S,B,210400.000000,31500.000000,7169.000000,2030.000000,662.500000,239.200000,93.260000,38.640000,
16.780000,7.541000,3.482000,1.618000,0.627000,0.293400,0.131000,0.058150
c,1.16,0.0000058419,0.0000454146,0.0002389029,0.0010079000,0.0036529523,0.0117622375,0.0338828343
,0.0857657026,0.1830564860,0.3066666848,0.3420305417,0.1767395801,0.0161871420,-0.0072524426,-
0.0045661166,-0.0017393129
c,1.16,0.0000011280,0.0000087607,0.0000461618,0.0001945321,0.0007082580,0.0022868566,0.0067020510
,0.0174065585,0.0397538712,0.0754768239,0.1190307837,0.1092470410,-0.1137525839,-0.4401365807,-
0.4644885380,-0.1275880120
c,1.16,-0.0000015351,-0.0000119591,-0.0000627416,-0.0002659788,-0.0009617749,-0.0031373676,-
0.0091280070,-0.0242286332,-0.0553385063,-0.1084827858,-0.1748472341,-
0.1931910158,0.7386485838,0.9645338675,-0.8678854847,-0.5839131756
c,1.16,-0.0000021841,-0.0000156486,-0.0000925055,-0.0003342419,-0.0014757077,-0.0037751752,-
0.0148890871,-0.0286407849,-0.1044732073,-0.1095604096,-0.4185836888,0.1397561796,2.4048770835,-
2.2319225539,-0.8640371769,1.4552578987
P,B,192.500000,45.640000,14.750000,5.503000,2.222000,0.959000,0.431400,0.196900,0.090330,0.040660
c,1.10,0.0001348580,0.0011483171,0.0058428161,0.0210758283,0.0611960100,0.1530710758,0.2943241620
,0.3642704356,0.2585309237,0.0717053089
c,1.10,0.0001038119,0.0010029799,0.0044027503,0.0159590908,0.0140866372,0.2036025057,0.6405610720
,0.0138043328,-0.7239551491,-0.2861547514
c,1.10,0.0003902960,0.0035872388,0.0181614638,0.0780319375,0.2455412103,0.8296400722,-
0.2884445870,-1.1575765444,0.5478083327,0.3678908853
D,B,2.886000,1.267000,0.556000,0.244000,0.107000
c,1.5,0.0124864080,0.0646166325,0.3523640343,0.4918272179,0.2485231797
c,1.5,-0.0449367119,0.2536987512,0.8659247615,-0.4512434011,-0.5802493218
F,B,1.651000,0.800200,0.387800,0.188000
c,1.4,0.0261389620,0.3045935590,0.4978105649,0.3215186188
S,C,312100.000000,46740.000000,10640.000000,3013.000000,982.800000,354.800000,138.400000,57.35000
0,24.920000,11.230000,5.201000,2.426000,0.967300,0.445600,0.197100,0.086350
c,1.16,0.0000056888,0.0000442122,0.0002324717,0.0009813983,0.0035604858,0.0114692478,0.0330825487
,0.0842739202,0.1811835207,0.3058726609,0.3428996624,0.1785203122,0.0174929057,-0.0069381856,-
0.0045895926,-0.0017787010
c,1.16,0.0000011517,0.0000089401,0.0000471067,0.0001985305,0.0007241159,0.0023373031,0.0068692727
,0.0179514067,0.0414871653,0.0795916232,0.1258249776,0.1068228241,-0.1286390810,-0.4398665406,-
0.4462025705,-0.1396137797
c,1.16,0.0000014615,0.0000113996,0.0000596491,0.0002537561,0.0009151876,0.0029997075,0.0086902270
,0.0233513200,0.0532963047,0.1071421338,0.1715711640,0.1810506143,-0.6618969705,-
0.9413749900,0.6896636760,0.7079116403
c,1.16,-0.0000020729,-0.0000152244,-0.0000868378,-0.0003294248,-0.0013722962,-0.0037795404,-
0.0136469384,-0.0290748718,-0.0939954201,-0.1270398885,-0.3903688653,0.0967690209,2.1831711797,-
1.6380587319,-1.3661318212,1.6011180083
P,C,295.200000,69.980000,22.640000,8.485000,3.459000,1.504000,0.678300,0.308700,0.140000,0.061780
c,1.10,0.0001429536,0.0012248907,0.0063589239,0.0235884808,0.0696777731,0.1664464149,0.2933564994
,0.3512017572,0.2563868868,0.0762169165
c,1.10,0.0001641191,0.0015014551,0.0073769191,0.0284317842,0.0674783713,0.2857591239,0.5586074972
,-0.0898519083,-0.6546507650,-0.2604391448
c,1.10,0.0004063047,0.0035967590,0.0197550020,0.0825675839,0.2839301676,0.7770202553,-
0.4435648258,-1.0388445407,0.5735322398,0.3869815823
D,C,4.542000,1.979000,0.862100,0.375600,0.163600
c,1.5,0.0143889048,0.0713989420,0.3570760522,0.4803354126,0.2551176449
c,1.5,-0.0007635462,0.3071489206,0.7744199003,-0.4242215167,-0.5691046088
F,C,2.631000,1.255000,0.598800,0.285700
c,1.4,0.0334914704,0.2983549101,0.5168396500,0.3061330041
S,N,432300.000000,64700.000000,14720.000000,4170.000000,1361.000000,491.200000,191.600000,79.4100
00,34.530000,15.580000,7.232000,3.382000,1.369000,0.624800,0.274700,0.119200
c,1.16,0.0000055936,0.0000435138,0.0002289287,0.0009650171,0.0035021904,0.0112921163,0.0326128265
,0.0832972697,0.1799856581,0.3050035130,0.3411593117,0.1774826966,0.0198840957,-
0.0012466348,0.0010401039,-0.0001265860

c,1.16,0.0000012377,0.0000096146,0.0000507014,0.0002132911,0.0007784594,0.0025133634,0.0073972480
,0.0193531475,0.0449446014,0.0862431945,0.1342509759,0.1060138777,-0.1384170918,-0.4391267599,-
0.4313435809,-0.1507186091
c,1.16,0.0000013948,0.0000109071,0.0000569709,0.0002427563,0.0008723951,0.0028758828,0.0082942767
,0.0225053948,0.0512093507,0.1048497272,0.1644290239,0.1710558715,-0.5974990638,-
0.9102913983,0.5234527691,0.8200521439
c,1.16,-0.0000020720,-0.0000153930,-0.0000865570,-0.0003344672,-0.0013599801,-0.0038661715,-
0.0134702049,-0.0300266728,-0.0925316587,-0.1386151714,-0.3824931664,0.0954870859,2.0469664361,-
1.3008336658,-1.6185133598,1.6426401236
P,N,415.900000,98.610000,31.920000,12.000000,4.919000,2.148000,0.969600,0.439900,0.197800,0.08603
0
c,1.10,0.0001493515,0.0012852780,0.0067482167,0.0254480843,0.0755743643,0.1758280802,0.2962195131
,0.3441099405,0.2503615773,0.0763931220
c,1.10,0.0001951090,0.0017570195,0.0089935233,0.0354017865,0.0970144192,0.3245625207,0.5070185471
,-0.1454680195,-0.6201309743,-0.2427206584
c,1.10,0.0004162692,0.0036425363,0.0208775389,0.0869515693,0.3019545646,0.7481924316,-
0.5366016092,-0.9624028745,0.5979386882,0.3812822948
D,N,6.717000,2.896000,1.249000,0.538000,0.232000
c,1.5,0.0153532025,0.0739377840,0.3611149183,0.4784008261,0.2548775648
c,1.5,0.0133057296,0.3124701443,0.7432037059,-0.3999521968,-0.5773663132
F,N,3.829000,1.795000,0.841000,0.394000
c,1.4,0.0362155054,0.3001021670,0.5301475088,0.2930118022
S,O,570800.000000,85480.000000,19460.000000,5512.000000,1798.000000,648.900000,253.100000,104.900
000,45.650000,20.620000,9.587000,4.493000,1.837000,0.834900,0.365800,0.157000
c,1.16,0.0000055477,0.0000431114,0.0002266878,0.0009564461,0.0034734806,0.0111986722,0.0323902914
,0.0828666917,0.1795999151,0.3052522946,0.3409416044,0.1774628746,0.0204404056,-
0.0011225476,0.0009015503,-0.0001420890
c,1.16,0.0000012619,0.0000097942,0.0000516082,0.0002173760,0.0007936340,0.0025636828,0.0075540649
,0.0198252847,0.0462226113,0.0893623473,0.1383434619,0.1051813067,-0.1491659047,-0.4435386388,-
0.4304217683,-0.1392847630
c,1.16,-0.0000014956,-0.0000116782,-0.0000610049,-0.0002599662,-0.0009359729,-0.0030816635,-
0.0089188306,-0.0242145842,-0.0555592008,-0.1145275935,-0.1828853636,-
0.1753958431,0.7138602082,0.8831041524,-0.7228450116,-0.6629030498
c,1.16,-0.0000021012,-0.0000153046,-0.0000883188,-0.0003296065,-0.0014011800,-0.0037697061,-
0.0140318733,-0.0289525787,-0.0982062901,-0.1307408651,-0.4339939687,0.1774273814,2.1865884409,-
1.8488602248,-1.0626335267,1.4644411549
P,O,525.600000,124.600000,40.340000,15.180000,6.245000,2.732000,1.227000,0.549200,0.241800,0.1025
00
c,1.10,0.0001676631,0.0014434160,0.0075973289,0.0288049894,0.0849419814,0.1899631174,0.2990478650
,0.3320569965,0.2419920846,0.0836337245
c,1.10,0.0002041327,0.0017992162,0.0093549749,0.0361612981,0.1047852150,0.3132249093,0.4464833129
,-0.1162190220,-0.5398121590,-0.3468451765
c,1.10,0.0003397509,0.0030597166,0.0166722601,0.0691106710,0.2246739298,0.6387770416,-
0.2244503728,-0.9381963502,0.1164759037,0.7366445524
D,O,8.253000,3.597000,1.568000,0.684000,0.298000
c,1.5,0.0204005719,0.0941369585,0.3964770909,0.4553601610,0.2204057295
c,1.5,0.0253040956,0.3693832197,0.6505508649,-0.4116458521,-0.5804977663
F,O,5.430000,2.416000,1.075000,0.478000
c,1.4,0.0335228290,0.2884311646,0.5272437798,0.3296607779
S,F,723500.000000,108400.000000,24680.000000,6990.000000,2282.000000,824.600000,321.800000,133.50
0000,58.110000,26.280000,12.240000,5.747000,2.365000,1.071000,0.468100,0.199400
c,1.16,0.0000055553,0.0000431553,0.0002268882,0.0009575524,0.0034684130,0.0111796501,0.0323122990
,0.0827520288,0.1797788209,0.3053815712,0.3399668832,0.1764600805,0.0211818400,0.0002018100,0.002
0122870,0.0001869180
c,1.16,0.0000013026,0.0000101077,0.0000532435,0.0002243797,0.0008168400,0.0026390477,0.0077680664
,0.0204244295,0.0477452371,0.0924737947,0.1419594377,0.1040673447,-0.1571528412,-0.4453843402,-
0.4282969012,-0.1334229001
c,1.16,-0.0000015812,-0.0000123348,-0.0000644826,-0.0002745547,-0.0009873826,-0.0032450794,-
0.0094085579,-0.0255267961,-0.0590490461,-0.1218295932,-0.1971062346,-
0.1743133493,0.8011339277,0.8433478026,-0.8437666826,-0.5565415269
c,1.16,-0.0000022138,-0.0000157194,-0.0000939462,-0.0003342040,-0.0015031547,-0.0037496030,-
0.0152245069,-0.0282133583,-0.1087379605,-0.1214794589,-0.5124105037,0.3089446266,2.3171309573,-
2.4398848768,-0.4179981518,1.2155567456
P,F,660.000000,156.400000,50.640000,19.080000,7.872000,3.449000,1.545000,0.686400,0.298600,0.1245
00
c,1.10,0.0001782292,0.0015363519,0.0081228434,0.0309453269,0.0908038263,0.1976141799,0.3005906180
,0.3264224499,0.2396685264,0.0812040964
c,1.10,0.0002343478,0.0020724260,0.0108206813,0.0423975838,0.1252617416,0.3557554018,0.4260597814
,-0.1962653428,-0.5539365531,-0.2621522640
c,1.10,0.0003963333,0.0033830461,0.0196902218,0.0773666944,0.2753925619,0.6855839940,-
0.4987901060,-0.8786594053,0.4221537048,0.5253050835

```
D,F,10.573000,4.613000,2.013000,0.878000,0.383000
c,1.5,0.0227863167,0.1014701669,0.4106536839,0.4490885351,0.2033548260
c,1.5,0.0338687384,0.3932523552,0.6202921281,-0.4439737872,-0.5562789681
F,F,7.563000,3.330000,1.466000,0.645000
c,1.4,0.0256618573,0.2724334030,0.5429625432,0.3335503239
S,Ne,902400.000000,135100.000000,30750.000000,8710.000000,2842.000000,1026.000000,400.100000,165.
900000,72.210000,32.660000,15.220000,7.149000,2.957000,1.335000,0.581600,0.246300
c,1.16,0.0000055071,0.0000428234,0.0002251425,0.0009501602,0.0034471885,0.0111254471,0.0322056752
,0.0825989133,0.1799056420,0.3060520777,0.3401255836,0.1761682211,0.0210152784,-
0.0005074379,0.0010578574,-0.0000598773
c,1.16,0.0000012963,0.0000100690,0.0000530405,0.0002235171,0.0008150559,0.0026368822,0.0077760083
,0.0204887059,0.0480966546,0.0935683962,0.1433560718,0.1021558762,-0.1629347673,-0.4466761688,-
0.4258625122,-0.1303062944
c,1.16,-0.0000016115,-0.0000125800,-0.0000657939,-0.0002799674,-0.0010092608,-0.0033183047,-
0.0096483669,-0.0261882549,-0.0609198166,-0.1260413906,-0.2054888974,-
0.1719233603,0.8489060997,0.8131694566,-0.9018536884,-0.4999251190
c,1.16,-0.0000022775,-0.0000158878,-0.0000974546,-0.0003344131,-0.0015728981,-0.0037161454,-
0.0160993168,-0.0275130195,-0.1165045331,-0.1142361471,-0.5766119902,0.4176357130,2.3884096204,-
2.8381924646,0.0497789435,1.0151612410
P,Ne,815.600000,193.300000,62.600000,23.610000,9.762000,4.281000,1.915000,0.847600,0.366000,0.151
000
c,1.10,0.0001847705,0.0015941093,0.0084653531,0.0324030611,0.0947184717,0.2026241681,0.3017949700
,0.3231669759,0.2371385895,0.0791176295
c,1.10,0.0002554864,0.0022579163,0.0118809731,0.0469683102,0.1409600178,0.3822895707,0.4049571155
,-0.2487725194,-0.5505559893,-0.2146565483
c,1.10,0.0004493493,0.0036446948,0.0225385482,0.0842160085,0.3254115521,0.7220346106,-
0.7514143816,-0.7615446193,0.6360693889,0.3274811852
D,Ne,13.317000,5.803000,2.529000,1.102000,0.480000
c,1.5,0.0247658998,0.1068765548,0.4205406931,0.4449468742,0.1908712837
c,1.5,0.0402982534,0.4048604855,0.5998920244,-0.4614427842,-0.5440313566
F,Ne,10.356000,4.538000,1.989000,0.871000
c,1.4,0.0150197139,0.2512426801,0.5756220779,0.3197084635
S,Na,1918700.000000,287270.000000,65382.000000,18523.000000,6045.400000,2184.200000,853.110000,35
4.690000,155.330000,71.234000,33.835000,16.349000,8.008200,3.671300,1.711800,0.791470,0.354790,0.
090664,0.044313,0.021948,0.010821
c,1.21,0.0000027088,0.0000210578,0.0001107868,0.0004675326,0.0017013284,0.0055150746,0.0162308700
,0.0431395655,0.1014860232,0.1993943053,0.3087288958,0.3067052305,0.1380881578,0.0228929786,0.013
2950975,0.0136239139,0.0037386766,0.0000744574,-0.0000095217,0.0000213706,0.0000000000
c,1.21,0.0000007485,0.0000058177,0.0000306178,0.0001292259,0.0004709708,0.0015307346,0.0045412540
,0.0122699314,0.0299593063,0.0633214284,0.1142954747,0.1541637857,0.0893386546,-0.1659774106,-
0.4384235088,-0.4291049529,-0.1293167239,0.0004404906,-0.0024793949,0.0009626429,-0.0004041010
c,1.21,0.0000001096,0.0000008513,0.0000044829,0.0000189095,0.0000689893,0.0002241299,0.0006666895
,0.0018044493,0.0044437500,0.0094931411,0.0176175702,0.0247238067,0.0150685595,-0.0326908857,-
0.0905675951,-0.1275950087,-0.1221995181,0.2666159471,0.5622463461,0.2797583349,0.0184296293
c,1.21,-0.0000009121,-0.0000070597,-0.0000373862,-0.0001565746,-0.0005770453,-0.0018576772,-
0.0056416232,-0.0153304856,-0.0400233925,-0.0843610950,-0.1526565277,-0.2121931436,-
0.1082608080,0.9474074884,0.7691961289,-0.9537883808,-
0.5979001026,0.0463503976,0.2186199667,0.0803942641,0.0098346670
c,1.21,0.0000020318,0.0000161964,0.0000822300,0.0003647153,0.0012584616,0.0044759137,0.0128756216
,0.0444201519,0.1249910991,0.2836318908,0.1687893908,0.1879554186,-0.8576041123,-
1.2541324249,1.9793554125,0.4183235618,-1.2659024909,-
0.2173944226,0.4805607442,0.0010385908,0.0403416859
P,Na,3044.200000,720.020000,234.420000,89.666000,36.695000,16.256000,7.659300,3.712500,1.799200,0
.862880,0.401040,0.177590,0.080700,0.036700,0.016700
c,1.15,0.0000281041,0.0002565120,0.0014177652,0.0063916825,0.0231537137,0.0652632255,0.1440680587
,0.2426572379,0.3066501458,0.2855229241,0.1569049195,0.0250223117,0.0009795285,0.0001433108,0.000
0382855
c,1.15,0.0000422160,0.0003890985,0.0022214108,0.0103861947,0.0369679527,0.1020915363,0.2601454501
,0.4512333146,0.1116118230,-0.4765918702,-0.4161525704,-0.0994949247,-0.0117309641,-
0.0039101668,0.0002098260
D,Na,2.350000,0.560000,0.140000
c,1.3,0.8485363766,0.2801219125,-0.0151540850
S,Mg,3199100.000000,479000.000000,109010.000000,30878.000000,10075.000000,3637.900000,1419.500000
,589.350000,257.530000,117.170000,54.762000,26.288000,12.860000,6.260400,2.851400,1.305800,0.5833
10,0.168490,0.085646,0.045077,0.021934
c,1.21,0.0000017950,0.0000139619,0.0000734120,0.0003103614,0.0011295201,0.0036825151,0.0109059255
,0.0295927497,0.0721890156,0.1552649528,0.2727110821,0.3344487713,0.2199168441,0.0496777160,0.002
7601574,0.0008143242,0.0000760996,0.0000236754,-0.0000206013,0.0000110197,-0.0000027094
c,1.21,0.0000004537,0.0000035256,0.0000185673,0.0000783671,0.0002861870,0.0009325269,0.0027886571
,0.0076392459,0.0193013967,0.0438512116,0.0880107485,0.1372739709,0.1369763624,-0.0420379328,-
0.3727875673,-0.5190147633,-0.2112102621,-0.0027817880,-0.0015874092,0.0014257844,-0.0004188622
```

c,1.21,0.0000000875,0.0000006788,0.0000035808,0.0000150794,0.0000552299,0.0001793639,0.0005388577,0.0014701301,0.0037442976,0.0084806778,0.0173277526,0.0271678567,0.0286523167,-0.0104754726,-0.0857137348,-0.1685480845,-0.1679893506,0.2258424187,0.4543273830,0.3902252847,0.0848474620
c,1.21,0.0000000280,0.0000002395,0.0000010945,0.0000055492,0.0000160017,0.0000691337,0.0001458382,0.0005991665,0.0009158278,0.0037456039,0.0033686778,0.0148488419,-0.0029418998,0.0203771606,-0.0755531699,0.0331803053,-0.2102622844,0.8016900862,-2.1344067866,4.2379493487,-3.1742581236
c,1.21,0.0000000647,0.0000004376,0.0000028009,0.0000090621,0.0000457595,0.0000986988,0.0004763893,0.0007145585,0.0035949061,0.0032847741,0.0191881365,0.0022781446,0.0566985070,-0.0772645697,0.0766595026,-0.3929458012,0.3208416733,-2.1814872063,8.0281545753,-8.9223905776,3.1552746770
P,Mg,4157.800000,984.530000,320.090000,121.730000,49.892000,22.542000,10.749000,5.289900,2.599600,1.251800,0.592460,0.269410,0.122500,0.055700,0.025300
c,1.15,0.0000236086,0.0002172468,0.0012060672,0.0056165426,0.0202158577,0.0566094004,0.1300385273,0.2291317315,0.3145207710,0.3051945012,0.1535948983,0.0225671416,0.0011363648,0.0002596972,0.0000137661
c,1.15,0.0000014167,0.0000192918,0.0000735564,0.0004918818,0.0012531758,0.0051729321,0.0075510231,0.0236912477,0.0120432758,0.0526499427,-0.0400496777,0.0831726078,-0.3817442098,0.3581759158,-1.1158128170
D,Mg,3.360000,0.800000,0.200000
c,1.3,0.0216295093,0.0049738185,0.9960022920
S,Al,3652000.000000,546800.000000,124500.000000,35440.000000,11840.000000,4434.000000,1812.000000,791.500000,361.000000,169.500000,81.680000,40.280000,20.250000,10.230000,4.802000,2.339000,1.163000,0.588200,0.231100,0.102700,0.045210
c,1.21,0.0000018637,0.0000144635,0.0000761824,0.0003158235,0.0010973874,0.0033697098,0.0093221881,0.0237993006,0.0568193211,0.1224688474,0.2238975904,0.3134446769,0.2749772585,0.1105733913,0.0119196229,0.0006369322,0.0004487692,-0.0000380120,0.0000593791,0.0000587612,0.0000261452
c,1.21,0.0000004848,0.0000037607,0.0000198230,0.0000821362,0.0002859936,0.0008789728,0.0024496839,0.0063137218,0.0154946400,0.0349794080,0.0708150170,0.1194903762,0.1489285897,0.0590936008,-0.2167547776,-0.4769006098,-0.3764545105,-0.0870507052,-0.0012553997,0.0022755184,0.0005978034
c,1.21,0.0000001107,0.0000008591,0.0000045255,0.0000187694,0.0000652773,0.0002009878,0.0005592223,0.0014463830,0.0035455988,0.0080644174,0.0163827254,0.0282463510,0.0359202031,0.0151042507,-0.0605797083,-0.1508458697,-0.2197750427,-0.0945734630,0.4009096114,0.6098385480,0.1812425049
c,1.21,0.0000001496,0.0000011735,0.0000060873,0.0000257771,0.0000871996,0.0002783642,0.0007395216,0.0020256318,0.0046422363,0.0114436286,0.0211580673,0.0414650571,0.0435211688,0.0324891435,-0.1196504024,-0.1600031964,-0.5198351326,0.1665990524,1.8438321393,-0.8377239912,-0.7637343604
c,1.21,0.0000002061,0.0000017153,0.0000081490,0.0000387797,0.0001118472,0.0004371204,0.0008870741,0.0033529949,0.0051703822,0.0200182998,0.0208212154,0.0817328395,0.0153743546,0.1376098151,-0.4141414401,0.2147209157,-2.0927076649,3.5139060536,-0.8395895195,-2.1906843624,1.8376241172
P,Al,2884.000000,683.200000,222.000000,84.820000,35.810000,16.220000,7.702000,3.741000,1.831000,0.887800,0.398900,0.171800,0.072980,0.030690
c,1.14,0.0000638513,0.0005636181,0.0031720969,0.0132523750,0.0433836653,0.1120603104,0.2180142757,0.3119431736,0.3169439761,0.1789251325,0.0309903761,0.0001206735,0.0006689449,-0.0001263149
c,1.14,0.0000120316,0.0001068310,0.0005990995,0.0025255848,0.0082911461,0.0218958429,0.0430126547,0.0633327606,0.0677298350,0.0417050279,-0.1302636002,-0.4202655134,-0.4498469253,-0.1544401658
c,1.14,0.0000142841,0.0001360566,0.0007115502,0.0032161490,0.0097360249,0.0278786993,0.0498714840,0.0877150859,0.0707128658,0.1575376271,-0.4809779769,-0.8348210970,0.7149805981,0.4861292394
c,1.14,0.0000238482,0.0001942814,0.0011880114,0.0045989411,0.0163337058,0.0382089444,0.0942740455,0.1110426891,0.1908934527,0.1369332131,-1.8722530557,1.6516107117,0.2220423910,-0.8236450391
D,Al,2.214300,0.944900,0.403200,0.172100,0.073430
c,1.5,0.0178097303,0.0104024313,0.2594736718,0.5570493165,0.3064913572
c,1.5,0.0081280932,-0.0780708823,0.8755073302,0.0740558985,-0.9045955390
F,Al,0.875600,0.447200,0.228400,0.116700
c,1.4,-0.0156499578,0.2903045341,0.4536392079,0.3917011935
S,Si,4465000.000000,668500.000000,152200.000000,43300.000000,14410.000000,5394.000000,2212.000000,968.100000,441.200000,207.100000,99.800000,49.240000,24.740000,12.470000,5.795000,2.830000,1.407000,0.699500,0.308300,0.138500,0.061450
c,1.21,0.0000017473,0.0000135737,0.0000714074,0.0002972583,0.0010382833,0.0031746754,0.0087324958,0.0223831560,0.0537277388,0.1166496266,0.2159801909,0.3095683722,0.2839493584,0.1222386085,0.0141879507,0.0002860923,0.0005809654,-0.0001362624,0.0001132324,0.0000583027,0.0000334760
c,1.21,0.0000004656,0.0000036154,0.0000190351,0.0000791894,0.0002772083,0.0008481095,0.0023503240,0.0060769746,0.0149870123,0.0340090641,0.0695329490,0.1191292035,0.1538141850,0.0705550699,-0.2133828413,-0.4923372859,-0.3795188177,-0.0760495636,0.0002918897,0.0022699407,0.0007920933
c,1.21,0.0000001174,0.0000009117,0.0000047979,0.0000199725,0.0000698666,0.0002140131,0.0005925740,0.0015361117,0.0037888605,0.0086522181,0.0177881630,0.0310894097,0.0411817036,0.0198252138,-0.0661548777,-0.1800558890,-0.2586501325,-0.0634512383,0.4458147628,0.5867135228,0.1713355928
c,1.21,0.0000001544,0.0000012097,0.0000062868,0.0000266189,0.0000910384,0.0002872714,0.0007656291,0.0020810559,0.0048593425,0.0118522651,0.0226106642,0.0438278795,0.0504887020,0.0362050687,-0.1270928643,-0.2001256711,-0.5742179324,0.3055779655,1.7987083339,-0.8746987215,-0.7519397942
c,1.21,0.0000002050,0.0000016473,0.0000082479,0.0000367054,0.0001174087,0.0004040679,0.0009612055,0.0030005173,0.0059515591,0.0175729444,0.0267914016,0.0691625792,0.0504674095,0.0844721410,-0.2942164003,-0.1272357349,-1.5982515759,3.5088049039,-1.0992028759,-2.2412805449,1.9071138893

```
P,Si,3572.000000,846.000000,274.800000,105.000000,44.350000,20.080000,9.530000,4.634000,2.280000,
1.116000,0.499100,0.225400,0.100100,0.043320
c,1.14,0.0000598101,0.0005290197,0.0029927264,0.0126203482,0.0418585003,0.1101297267,0.2185746699
,0.3174806034,0.3193308887,0.1714145959,0.0272061913,-0.0003246500,0.0008254369,-0.0001201983
c,1.14,0.0000130489,0.0001153341,0.0006545621,0.0027679638,0.0092817285,0.0248181605,0.0504241847
,0.0744168937,0.0817489341,0.0308043509,-0.1781724925,-0.4297428083,-0.4115302182,-0.1316751465
c,1.14,0.0000157401,0.0001447401,0.0007900959,0.0034753084,0.0111422064,0.0310943218,0.0607776749
,0.0978451850,0.0962138762,0.1239530560,-0.6357752212,-0.6602065294,0.7632107906,0.4370191772
c,1.14,0.0000300647,0.0002019631,0.0015081670,0.0048865849,0.0217118781,0.0416318863,0.1359243112
,0.0944419852,0.3177488798,-0.2729942959,-1.6466097242,1.9401066733,-0.0999669875,-0.7318449301
D,Si,3.238600,1.376700,0.585300,0.248800,0.105800
c,1.5,0.0197751361,0.0150954672,0.2572361186,0.5560472315,0.3081147567
c,1.5,0.0047324796,-0.0963416463,0.8702035508,0.1002739841,-0.9214189416
F,Si,1.351000,0.660000,0.322500,0.157500
c,1.4,-0.0131724073,0.2474597385,0.5293164377,0.3562072296
S,P,5384000.000000,806200.000000,183600.000000,52250.000000,17390.000000,6523.000000,2687.000000,
1178.000000,536.200000,251.500000,121.300000,59.880000,30.050000,15.120000,7.010000,3.441000,1.71
2000,0.833700,0.391200,0.177700,0.079390
c,1.21,0.0000016457,0.0000127815,0.0000672065,0.0002797109,0.0009766998,0.0029683492,0.0081239977
,0.0209200106,0.0505590328,0.1104791033,0.2069569123,0.3047373414,0.2929523106,0.1355607116,0.017
3207816,-0.0000351698,0.0008032620,-0.0002438395,0.0000987220,-0.0000512279,0.0000100911
c,1.21,0.0000004469,0.0000034695,0.0000182569,0.0000759386,0.0002657236,0.0008080453,0.0022273283
,0.0057833199,0.0143437812,0.0327060584,0.0673715266,0.1176471371,0.1572798118,0.0838541752,-
0.1997171854,-0.4986030336,-0.3898171231,-0.0743425810,-0.0007367752,-0.0009261894,0.0000000000
c,1.21,0.0000001233,0.0000009570,0.0000050356,0.0000209489,0.0000732948,0.0002229871,0.0006147004
,0.0015982748,0.0039699165,0.0090937104,0.0188701405,0.0335159167,0.0461471119,0.0256075836,-
0.0673395749,-0.2038094414,-0.2883032752,-0.0407361705,0.4713076887,0.5659660071,0.1702693605
c,1.21,0.0000001526,0.0000011946,0.0000062114,0.0000262588,0.0000899315,0.0002814550,0.0007481310
,0.0020348735,0.0048018560,0.0117025222,0.0226723683,0.0443417480,0.0542824195,0.0413471455,-
0.1274586979,-0.2161431174,-0.5952197935,0.3712623750,1.7272055910,-0.8075089899,-0.8027687020
c,1.21,0.0000002017,0.0000015901,0.0000081822,0.0000350779,0.0001179139,0.0003782505,0.0009740148
,0.0027560811,0.0062251053,0.0160328102,0.0293865348,0.0624183676,0.0694862147,0.0623982686,-
0.2222763088,-0.3277686050,-1.2659961520,3.3580397583,-1.0193956105,-2.4607237219,1.9996496274
P,P,4552.000000,1078.000000,350.100000,133.800000,56.520000,25.580000,12.140000,5.902000,2.910000
,1.435000,0.657000,0.300500,0.134000,0.057830
c,1.14,0.0000519530,0.0004604024,0.0026208164,0.0111872441,0.0378228974,0.1021163975,0.2103137453
,0.3173823797,0.3271640365,0.1776869820,0.0293588218,-0.0000752283,0.0006795891,-0.0001259461
c,1.14,0.0000125053,0.0001106415,0.0006324495,0.0027051340,0.0092564586,0.0254006335,0.0537628786
,0.0825945281,0.0926223095,0.0309116256,-0.1930952447,-0.4363625520,-0.4008800592,-0.1220492925
c,1.14,0.0000155755,0.0001422935,0.0007886088,0.0034835891,0.0115313768,0.0327400156,0.0676387623
,0.1100021755,0.1118379956,0.1082446644,-0.6884915103,-0.5895119846,0.7746977189,0.4128842454
c,1.14,0.0000299529,0.0001996742,0.0015148645,0.0049567751,0.0226188204,0.0452278279,0.1495691160
,0.1160589965,0.3290175161,-0.3924085811,-1.5315937173,1.9633920738,-0.1686624408,-0.7134121876
D,P,4.300800,1.834600,0.782600,0.333900,0.142400
c,1.5,0.0212636418,0.0188050678,0.2647976295,0.5535103472,0.3004340001
c,1.5,-0.0010126574,-0.1034761325,0.8914468081,0.0694034624,-0.9131456221
F,P,1.816000,0.880600,0.427000,0.207000
c,1.4,-0.0126043063,0.2459544396,0.5491262846,0.3363334805
S,S,6297000.000000,943100.000000,214900.000000,61250.000000,20450.000000,7719.000000,3198.000000,
1402.000000,637.200000,298.900000,144.300000,71.210000,35.730000,17.970000,8.341000,4.112000,2.04
5000,0.977000,0.476600,0.218500,0.097590
c,1.21,0.0000015921,0.0000123599,0.0000649159,0.0002693326,0.0009346034,0.0028081493,0.0076736278
,0.0198887182,0.0482561629,0.1057504175,0.2002096787,0.3007047557,0.2986548787,0.1463267075,0.020
1597202,-0.0001027048,0.0011010907,-0.0003101426,-0.0001743228,-0.0004191457,-0.0000912518
c,1.21,0.0000004382,0.0000034001,0.0000178707,0.0000741041,0.0002576639,0.0007746516,0.0021312954
,0.0055689211,0.0138537158,0.0316453111,0.0656714009,0.1163667541,0.1591288761,0.0941285220,-
0.1869455747,-0.5017349200,-0.3954799564,-0.0732558993,-0.0061600338,-0.0086419404,-0.0021011099
c,1.21,0.0000001350,0.0000010473,0.0000055054,0.0000228258,0.0000793897,0.0002386496,0.0006570998
,0.0017175837,0.0042826970,0.0098118258,0.0205253893,0.0368719010,0.0519727886,0.0318049495,-
0.0692984997,-0.2299302040,-0.3169482049,-0.0159749150,0.4957998068,0.5502198982,0.1581023881
c,1.21,0.0000001515,0.0000011864,0.0000061554,0.0000259766,0.0000882303,0.0002737658,0.0007237910
,0.0019883683,0.0046884897,0.0114963962,0.0223354902,0.0445622770,0.0556857997,0.0465569275,-
0.1262555207,-0.2175452587,-0.6283016341,0.4241886575,1.7450512169,-0.8970414287,-0.7683501822
c,1.21,0.0000002175,0.0000017014,0.0000088365,0.0000372408,0.0001267345,0.0003923294,0.0010410141
,0.0028512138,0.0067679204,0.0165725289,0.0326736745,0.0651357654,0.0850063621,0.0624786338,-
0.2344979072,-0.4090533612,-1.2528100846,3.9386872408,-1.9634316309,-1.8355947721,1.7827651294
P,S,5266.000000,1247.000000,405.000000,154.800000,65.380000,29.590000,14.040000,6.824000,3.369000
,1.666000,0.768100,0.350400,0.155600,0.066810
c,1.14,0.0000522613,0.0004635223,0.0026409857,0.0113168621,0.0384702429,0.1043389802,0.2156829406
,0.3252583670,0.3261796946,0.1631472917,0.0238968197,0.0004741483,0.0007366333,0.0000198570
```

```
c,1.14,0.0000134501,0.0001188809,0.0006815660,0.0029222411,0.0100822376,0.0277819341,0.0593004576
,0.0911250782,0.1004947289,0.0122559698,-0.2376634453,-0.4374524061,-0.3652713498,-0.1137546274
c,1.14,0.0000154652,0.0001408791,0.0007845892,0.0034675017,0.0116099085,0.0331206035,0.0693083831
,0.1105846677,0.1084933001,0.0773246928,-0.7015469162,-0.4723029029,0.6913382861,0.4658323810
c,1.14,0.0000261265,0.0001831142,0.0013237518,0.0045596034,0.0199287095,0.0428388835,0.1310204730
,0.1141021004,0.2824872816,-0.3619757334,-1.2922486103,1.4371629676,0.4007309084,-1.0014972170
D,S,5.075500,2.183300,0.939200,0.404000,0.173800
c,1.5,0.0251450138,0.0247353053,0.2922763872,0.5461216262,0.2745860461
c,1.5,-0.0034219833,-0.0685076016,0.9010768772,-0.0118517043,-0.8849727408
F,S,1.322200,0.731900,0.405100,0.224300
c,1.4,0.0748384273,0.4165506000,0.3451685431,0.3002791153
S,Cl,6410000.000000,959600.000000,218300.000000,61810.000000,20140.000000,7264.000000,2832.000000
,1175.000000,512.600000,233.000000,109.500000,52.860000,25.840000,12.170000,6.030000,3.012000,1.5
11000,0.660400,0.292600,0.125400
c,1.20,0.0000018134,0.0000141111,0.0000742369,0.0003141152,0.0011463623,0.0037386909,0.0110940777
,0.0301135997,0.0739105226,0.1582493814,0.2747350951,0.3340364624,0.2175597801,0.0457431155,-
0.0000293391,0.0018350275,-0.0004445327,-0.0000776684,-0.0004587124,-0.0000940595
c,1.20,0.0000005314,0.0000041356,0.0000217541,0.0000921099,0.0003363575,0.0011004694,0.0032848588
,0.0090573809,0.0229428466,0.0525801453,0.1042883588,0.1654622541,0.1540218392,-0.0738403143,-
0.4640154060,-0.5674793085,-0.1905085616,0.1231404358,0.1814151436,0.0572888245
c,1.20,0.0000000030,0.0000000242,0.0000001227,0.0000005444,0.0000018615,0.0000064915,0.0000171666
,0.0000496124,0.0000906845,0.0001672956,-0.0001520201,-0.0011870140,-
0.0035702994,0.0044587752,0.0423049009,0.1729287178,0.0799121551,-0.4305627967,-0.5800768760,-
0.1833322170
c,1.20,0.0000001698,0.0000013020,0.0000069960,0.0000288036,0.0001089500,0.0003415483,0.0010740420
,0.0027892591,0.0076251234,0.0161014859,0.0362533789,0.0504717507,0.0667371854,-0.0617185781,-
0.1249324583,-0.6046474562,-0.1783077760,2.1890408950,-0.8544231506,-0.8253035227
c,1.20,-0.0000002454,-0.0000017038,-0.0000105333,-0.0000358464,-0.0001710464,-0.0003995626,-
0.0017689839,-0.0029990509,-0.0133610420,-0.0149230657,-0.0714207934,-0.0238172309,-
0.2061927692,0.3578970532,-0.3577280432,2.6924701253,-3.9540969440,0.6262331788,2.5843599682,-
1.9044798894
P,Cl,2548.000000,603.700000,195.600000,74.150000,30.940000,13.690000,6.229000,2.878000,1.282000,0
.564100,0.234800,0.093120
c,1.12,0.0002357017,0.0020515799,0.0111543331,0.0439815972,0.1299942320,0.2729591832,0.3836904520
,0.2918694533,0.0704461278,0.0012867770,0.0018296711,0.0000163527
c,1.12,0.0000639499,0.0005569862,0.0030474869,0.0121470785,0.0368757425,0.0796346992,0.1179864705
,0.0871432235,-0.1421012819,-0.4276857233,-0.4434172620,-0.1623961240
c,1.12,0.0000792628,0.0007426379,0.0037825549,0.0163312278,0.0460944074,0.1119987183,0.1292753470
,0.1725511490,-0.4628884187,-0.7987449671,0.6410531198,0.5328110609
c,1.12,0.0001077266,0.0010272250,0.0051645255,0.0228840699,0.0651495253,0.1620792145,0.1608737681
,0.2854448087,-1.7256993596,1.1337070246,0.7415976309,-1.0475106715
D,Cl,0.250000,0.618000,1.529000,3.781000
c,1.4,0.3728907832,0.5736769106,0.1719760944,0.0371180574
c,1.4,-1.0387870410,0.4428723678,0.6549212715,-0.0895377154
F,Cl,0.320000,0.656000,1.345000
c,1.3,0.3304730660,0.5388098289,0.2567793816
S,Ar,9149000.000000,1370000.000000,311900.000000,88650.000000,29330.000000,10930.000000,4480.0000
00,1962.000000,894.100000,419.600000,202.300000,99.840000,50.070000,25.140000,11.810000,5.882000,
2.939000,1.405000,0.696300,0.318800,0.141000
c,1.21,0.0000013427,0.0000104386,0.0000548567,0.0002295828,0.0008103338,0.0024853413,0.0068369173
,0.0176198815,0.0428751718,0.0954852872,0.1850640043,0.2890415368,0.3101655965,0.1721832194,0.028
5227179,-0.0005757381,0.0011812150,-0.0004805349,0.0001888494,-0.0000972509,0.0000174327
c,1.21,0.0000003813,0.0000029633,0.0000155803,0.0000651880,0.0002304322,0.0007074586,0.0019572963
,0.0050856225,0.0126528350,0.0293064513,0.0617712186,0.1125407214,0.1622931602,0.1184119757,-
0.1461475119,-0.4977515049,-0.4342396429,-0.0890745104,-0.0005380500,-0.0020403603,0.0000280176
c,1.21,0.0000001196,0.0000009295,0.0000048884,0.0000204455,0.0000723150,0.0002219097,0.0006147545
,0.0015970984,0.0039865334,0.0092577792,0.0197068769,0.0364106866,0.0543600462,0.0412101400,-
0.0557198596,-0.2389790991,-0.3480660572,-0.0114046784,0.5088914666,0.5484078881,0.1564441373
c,1.21,-0.0000001367,-0.0000010740,-0.0000055565,-0.0000237565,-0.0000816261,-0.0002602096,-
0.0006861311,-0.0018950399,-0.0044077494,-0.0111231815,-0.0215812668,-0.0450890638,-
0.0582069373,-0.0607658119,0.1167192188,0.2034650119,0.7215300226,-0.4385972721,-
1.8449057279,1.0202646083,0.7094666949
c,1.21,0.0000002027,0.0000015917,0.0000082454,0.0000351960,0.0001212004,0.0003853461,0.0010202625
,0.0028076686,0.0065790981,0.0165651285,0.0326566656,0.0682935031,0.0931850677,0.0892905674,-
0.2510363395,-0.3897714652,-1.5121815194,4.5898285938,-2.7289064028,-1.3248076648,1.5940922166
P,Ar,7050.000000,1669.000000,542.100000,207.100000,87.520000,39.610000,18.780000,9.130000,4.51600
0,2.245000,1.065000,0.488500,0.216600,0.092550
c,1.14,0.0000501843,0.0004453650,0.0025479973,0.0110155237,0.0378489764,0.1043551933,0.2193351507
,0.3346153067,0.3267710198,0.1481530147,0.0192586150,0.0005519368,0.0001769246,-0.0000168555
c,1.14,0.0000141163,0.0001249327,0.0007189150,0.0031134821,0.0108614712,0.0305213102,0.0664087472
,0.1043355196,0.1104812265,-0.0132896226,-0.2782215414,-0.4348655739,-0.3375752082,-0.0952338864
```

```
c,1.14,0.0000181269,0.0001604674,0.0009241383,0.0040094773,0.0140247320,0.0396644574,0.0883804352
,0.1325313304,0.1419961135,-0.0015187742,-0.8792271203,-0.2138306098,0.7842254682,0.2866213418
c,1.14,0.0000343626,0.0002030576,0.0017464185,0.0051788717,0.0270262790,0.0507693530,0.1933756399
,0.1015726509,0.4355928575,-0.9855080991,-0.8432102702,2.0212689369,-0.6689038659,-0.4774954241
D,Ar,7.632700,3.287600,1.416000,0.609900,0.262700
c,1.5,0.0279057812,0.0293683713,0.3030943731,0.5422799268,0.2640015406
c,1.5,-0.0032892550,-0.0701912500,0.9235793714,-0.0616176285,-0.8648720787
F,Ar,3.058200,1.529200,0.764700,0.382400
c,1.4,-0.0106008451,0.3045834586,0.5423443185,0.2771750082
}
```

S7.2 GAUSSIAN Format

```
!basis set ano-pVTZ
!
! ANO basis sets from atomic MR-ACPF based on cc-pV6Z primitives
!
! H-Al: Neese, F.; Valeev, E.F J. Chem. Theo. Comp., 2010, submitted for publication
! ORIGINAL FILE WAS BOGUS COPY OF aug-pVDZ: corrected Jan 27, 2012 by Gershom Martin
! how: copied ano-pVQZ, stripped off top angular momentum plus last ANO of each remaining angular
momentum
!
! converted 31/12/2025 by MAI using home-written PERL script
-H
S 8 1.0
4.02009900e+02   -4.42950800e-04
6.02419600e+01   -3.25074670e-03
1.37321700e+01   -1.81152250e-02
3.90450500e+00   -6.36329998e-02
1.28270900e+00   -2.22163641e-01
4.65544000e-01   -4.04104783e-01
1.81120000e-01   -3.48108109e-01
7.27910000e-02   -9.27213251e-02
S 8 1.0
4.02009900e+02   -5.85791700e-04
6.02419600e+01   -2.09634430e-03
1.37321700e+01   -3.04299021e-02
3.90450500e+00   -2.88272025e-02
1.28270900e+00   -7.52694563e-01
4.65544000e-01   -5.30306000e-01
1.81120000e-01   9.52467620e-01
7.27910000e-02   1.94410430e-01
S 8 1.0
4.02009900e+02   -6.08315000e-04
6.02419600e+01   -5.24835000e-03
1.37321700e+01   -2.54171128e-02
3.90450500e+00   -2.37640640e-01
1.28270900e+00   -1.70965835e+00
4.65544000e-01   2.67896543e+00
1.81120000e-01   -1.03338631e+00
7.27910000e-02   -2.48623702e-01
P 6 1.0
9.88000000e+00   5.53122400e-03
3.95000000e+00   5.32324280e-03
1.58000000e+00   1.50384747e-01
6.30000000e-01   5.82944394e-01
2.50000000e-01   3.39947055e-01
1.00000000e-01   3.17494051e-02
P 6 1.0
9.88000000e+00   -2.80600947e-02
3.95000000e+00   3.43704220e-02
1.58000000e+00   -8.94015866e-01
6.30000000e-01   -2.47268769e-01
2.50000000e-01   8.48782689e-01
1.00000000e-01   2.28203559e-02
D 4 1.0
4.00000000e+00   2.20110300e-03
1.60000000e+00   -2.78252481e-01
6.40000000e-01   -6.65042830e-01
2.60000000e-01   -1.74847619e-01
```

```
****
-He
S 10 1.0
4.78500000e+03   6.03202000e-05
7.17000000e+02   4.68496000e-04
1.63200000e+02   2.45014670e-03
4.62600000e+01   1.01553835e-02
1.51000000e+01   3.50340333e-02
5.43700000e+00   9.92797341e-02
2.08800000e+00   2.17387130e-01
8.29700000e-01   3.47268217e-01
3.36600000e-01   3.44557817e-01
1.36900000e-01   1.18026005e-01
S 10 1.0
4.78500000e+03   8.88731000e-05
7.17000000e+02   6.94021400e-04
1.63200000e+02   3.64339710e-03
4.62600000e+01   1.57462582e-02
1.51000000e+01   6.14009646e-02
5.43700000e+00   2.41475510e-01
2.08800000e+00   6.11964132e-01
8.29700000e-01   1.96132944e-01
3.36600000e-01   -7.24164219e-01
1.36900000e-01   -3.38281481e-01
S 10 1.0
4.78500000e+03   1.28705400e-04
7.17000000e+02   1.14776780e-03
1.63200000e+02   5.09838010e-03
4.62600000e+01   3.06322873e-02
1.51000000e+01   1.39615961e-01
5.43700000e+00   8.57718403e-01
2.08800000e+00   9.48064849e-02
8.29700000e-01   -1.72505080e+00
3.36600000e-01   6.54259777e-01
1.36900000e-01   5.29420091e-01
P 5 1.0
3.87000000e-01   2.88744289e-01
9.84000000e-01   5.36134245e-01
2.49800000e+00   2.65902008e-01
6.34200000e+00   4.00702083e-02
1.61040000e+01   9.03858320e-03
P 5 1.0
3.87000000e-01   -6.54417328e-01
9.84000000e-01   -2.75230872e-01
2.49800000e+00   7.99308321e-01
6.34200000e+00   2.67165313e-01
1.61040000e+01   3.10059145e-02
D 4 1.0
7.47000000e-01   4.65842919e-01
1.91000000e+00   5.37343596e-01
4.88600000e+00   1.30942192e-01
1.24980000e+01   7.13509580e-03
****
-Li
S 19 1.0
7.06810000e+04   5.32360000e-06
1.35940000e+04   3.23747000e-05
3.10040000e+03   1.87960300e-04
8.26460000e+02   8.71221900e-04
2.53760000e+02   3.38367000e-03
8.84510000e+01   1.10494789e-02
3.44930000e+01   3.05955979e-02
1.48310000e+01   7.07341149e-02
6.92990000e+00   1.34555716e-01
3.46780000e+00   2.06961592e-01
1.83160000e+00   2.59108572e-01
1.00630000e+00   2.46866157e-01
5.66780000e-01   1.50116229e-01
3.22540000e-01   4.48272557e-02
1.82770000e-01   3.57552870e-03
1.01640000e-01   3.15460900e-04
```

```
5.46660000e-02   5.36622000e-04
2.80250000e-02   4.57424400e-04
1.34970000e-02   4.71593000e-05
S 19 1.0
7.06810000e+04   8.57700000e-07
1.35940000e+04   5.21600000e-06
3.10040000e+03   3.02866000e-05
8.26460000e+02   1.40458800e-04
2.53760000e+02   5.46352500e-04
8.84510000e+01   1.79186610e-03
3.44930000e+01   5.01200740e-03
1.48310000e+01   1.18375809e-02
6.92990000e+00   2.33643106e-02
3.46780000e+00   3.81867406e-02
1.83160000e+00   5.36789047e-02
1.00630000e+00   6.83347487e-02
5.66780000e-01   7.49471861e-02
3.22540000e-01   5.92482250e-02
1.82770000e-01   -2.60749945e-02
1.01640000e-01   -2.22043244e-01
5.46660000e-02   -4.73451586e-01
2.80250000e-02   -3.62075447e-01
1.34970000e-02   -4.07343934e-02
S 19 1.0
7.06810000e+04   8.04230000e-06
1.35940000e+04   4.90032000e-05
3.10040000e+03   2.84240000e-04
8.26460000e+02   1.32650290e-03
2.53760000e+02   5.20972940e-03
8.84510000e+01   1.79882238e-02
3.44930000e+01   5.71974540e-02
1.48310000e+01   1.78258163e-01
6.92990000e+00   4.29897013e-01
3.46780000e+00   4.50865827e-01
1.83160000e+00   -3.71352880e-02
1.00630000e+00   -4.87212099e-01
5.66780000e-01   -4.62766855e-01
3.22540000e-01   -1.60128984e-01
1.82770000e-01   -1.16236967e-02
1.01640000e-01   6.61103743e-02
5.46660000e-02   1.35132196e-01
2.80250000e-02   1.04706424e-01
1.34970000e-02   1.16751457e-02
S 19 1.0
7.06810000e+04   1.13754000e-05
1.35940000e+04   7.11224000e-05
3.10040000e+03   4.00728700e-04
8.26460000e+02   1.95738370e-03
2.53760000e+02   7.61399060e-03
8.84510000e+01   3.17360413e-02
3.44930000e+01   1.34215264e-01
1.48310000e+01   5.84227772e-01
6.92990000e+00   6.80609017e-01
3.46780000e+00   -1.00509861e+00
1.83160000e+00   -9.44670188e-01
1.00630000e+00   9.56130540e-02
5.66780000e-01   6.97462192e-01
3.22540000e-01   3.95465840e-01
1.82770000e-01   7.14809958e-02
1.01640000e-01   -8.18978750e-02
5.46660000e-02   -1.59881592e-01
2.80250000e-02   -1.28275016e-01
1.34970000e-02   -1.41083238e-02
P 8 1.0
2.85000000e+01   1.89870192e-02
6.64000000e+00   3.14107276e-01
1.92000000e+00   6.61727662e-01
7.70000000e-01   8.86066288e-02
3.20000000e-01   1.15657049e-01
1.50000000e-01   -6.47171322e-02
6.60000000e-02   2.69408101e-02
```

```
2.50000000e-02   -7.47757140e-03
P 8 1.0
2.85000000e+01   2.74718905e-02
6.64000000e+00   3.09097805e-01
1.92000000e+00   3.22503617e-01
7.70000000e-01   -3.78769172e-01
3.20000000e-01   -4.32978084e-01
1.50000000e-01   -2.89609514e-01
6.60000000e-02   -1.05459800e-01
2.50000000e-02   -9.18879160e-03
D 3 1.0
1.75000000e+00   1.00349740e+00
3.00000000e-01   -1.74235563e-02
1.10000000e-01   2.14475965e-02
****
-Be
S 19 1.0
1.39330000e+05   4.81370000e-06
2.67740000e+04   2.92950000e-05
6.11200000e+03   1.69653400e-04
1.63290000e+03   7.83798800e-04
5.03030000e+02   3.02966040e-03
1.76030000e+02   9.86889620e-03
6.89390000e+01   2.73158639e-02
2.97680000e+01   6.38839975e-02
1.39630000e+01   1.24475533e-01
7.00900000e+00   1.99794514e-01
3.70940000e+00   2.60302499e-01
2.03920000e+00   2.56116682e-01
1.14720000e+00   1.60223839e-01
6.50700000e-01   4.79172446e-02
3.66590000e-01   4.05193800e-03
2.02110000e-01   5.53253000e-05
1.07420000e-01   -1.46329000e-05
5.42310000e-02   5.42280000e-06
2.56190000e-02   0.00000000e+00
S 19 1.0
1.39330000e+05   8.96600000e-07
2.67740000e+04   5.45660000e-06
6.11200000e+03   3.16042000e-05
1.63290000e+03   1.46094500e-04
5.03030000e+02   5.65594800e-04
1.76030000e+02   1.85049760e-03
6.89390000e+01   5.17382070e-03
2.97680000e+01   1.23588076e-02
1.39630000e+01   2.50209252e-02
7.00900000e+00   4.29921324e-02
3.70940000e+00   6.40053839e-02
2.03920000e+00   8.37309282e-02
1.14720000e+00   8.86004427e-02
6.50700000e-01   4.53339368e-02
3.66590000e-01   -7.74503499e-02
2.02110000e-01   -2.76957508e-01
1.07420000e-01   -4.53239610e-01
5.42310000e-02   -3.01158473e-01
2.56190000e-02   -3.31442139e-02
S 19 1.0
1.39330000e+05   -1.33880000e-06
2.67740000e+04   -8.14490000e-06
6.11200000e+03   -4.72010000e-05
1.63290000e+03   -2.18114400e-04
5.03030000e+02   -8.45637900e-04
1.76030000e+02   -2.77009980e-03
6.89390000e+01   -7.80186690e-03
2.97680000e+01   -1.88623345e-02
1.39630000e+01   -3.93045895e-02
7.00900000e+00   -6.86639537e-02
3.70940000e+00   -1.01644502e-01
2.03920000e+00   -1.29787737e-01
1.14720000e+00   -1.78094164e-01
6.50700000e-01   -4.75153109e-02
```

```
3.66590000e-01   7.22048537e-01
2.02110000e-01   9.93782109e-01
1.07420000e-01   -3.70738876e-01
5.42310000e-02   -9.02262037e-01
2.56190000e-02   -1.16365225e-01
S 19 1.0
1.39330000e+05   -2.34770000e-06
2.67740000e+04   -1.41798000e-05
6.11200000e+03   -8.29446000e-05
1.63290000e+03   -3.79581800e-04
5.03030000e+02   -1.49527260e-03
1.76030000e+02   -4.86054750e-03
6.89390000e+01   -1.44204026e-02
2.97680000e+01   -3.59876745e-02
1.39630000e+01   -8.71441173e-02
7.00900000e+00   -1.38293538e-01
3.70940000e+00   -1.82972883e-01
2.03920000e+00   -8.17135288e-02
1.14720000e+00   -7.36377500e-01
6.50700000e-01   1.73804270e+00
3.66590000e-01   1.64665928e+00
2.02110000e-01   -2.58713691e+00
1.07420000e-01   -8.17785281e-01
5.42310000e-02   1.25142104e+00
2.56190000e-02   1.96797815e-01
P 8 1.0
4.57000000e+01   5.59857700e-04
1.06000000e+01   4.92309360e-03
3.08000000e+00   1.98821499e-02
1.23000000e+00   3.94518210e-02
5.10000000e-01   1.63060244e-01
2.30000000e-01   4.11157813e-01
1.06000000e-01   4.42934754e-01
4.00000000e-02   6.88676460e-02
P 8 1.0
4.57000000e+01   8.86234700e-04
1.06000000e+01   3.06619409e-02
3.08000000e+00   3.19215308e-02
1.23000000e+00   6.31896987e-01
5.10000000e-01   -7.69565173e-01
2.30000000e-01   -1.14099071e+00
1.06000000e-01   1.49192519e+00
4.00000000e-02   9.12650870e-03
D 3 1.0
3.35000000e+00   2.50099508e-02
5.70000000e-01   -9.16355551e-02
2.10000000e-01   -9.39409469e-01
****
-B
S 16 1.0
2.10400000e+05   5.84190000e-06
3.15000000e+04   4.54146000e-05
7.16900000e+03   2.38902900e-04
2.03000000e+03   1.00790000e-03
6.62500000e+02   3.65295230e-03
2.39200000e+02   1.17622375e-02
9.32600000e+01   3.38828343e-02
3.86400000e+01   8.57657026e-02
1.67800000e+01   1.83056486e-01
7.54100000e+00   3.06666685e-01
3.48200000e+00   3.42030542e-01
1.61800000e+00   1.76739580e-01
6.27000000e-01   1.61871420e-02
2.93400000e-01   -7.25244260e-03
1.31000000e-01   -4.56611660e-03
5.81500000e-02   -1.73931290e-03
S 16 1.0
2.10400000e+05   1.12800000e-06
3.15000000e+04   8.76070000e-06
7.16900000e+03   4.61618000e-05
2.03000000e+03   1.94532100e-04
```

```
6.62500000e+02   7.08258000e-04
2.39200000e+02   2.28685660e-03
9.32600000e+01   6.70205100e-03
3.86400000e+01   1.74065585e-02
1.67800000e+01   3.97538712e-02
7.54100000e+00   7.54768239e-02
3.48200000e+00   1.19030784e-01
1.61800000e+00   1.09247041e-01
6.27000000e-01   -1.13752584e-01
2.93400000e-01   -4.40136581e-01
1.31000000e-01   -4.64488538e-01
5.81500000e-02   -1.27588012e-01
S 16 1.0
2.10400000e+05   -1.53510000e-06
3.15000000e+04   -1.19591000e-05
7.16900000e+03   -6.27416000e-05
2.03000000e+03   -2.65978800e-04
6.62500000e+02   -9.61774900e-04
2.39200000e+02   -3.13736760e-03
9.32600000e+01   -9.12800700e-03
3.86400000e+01   -2.42286332e-02
1.67800000e+01   -5.53385063e-02
7.54100000e+00   -1.08482786e-01
3.48200000e+00   -1.74847234e-01
1.61800000e+00   -1.93191016e-01
6.27000000e-01   7.38648584e-01
2.93400000e-01   9.64533868e-01
1.31000000e-01   -8.67885485e-01
5.81500000e-02   -5.83913176e-01
S 16 1.0
2.10400000e+05   -2.18410000e-06
3.15000000e+04   -1.56486000e-05
7.16900000e+03   -9.25055000e-05
2.03000000e+03   -3.34241900e-04
6.62500000e+02   -1.47570770e-03
2.39200000e+02   -3.77517520e-03
9.32600000e+01   -1.48890871e-02
3.86400000e+01   -2.86407849e-02
1.67800000e+01   -1.04473207e-01
7.54100000e+00   -1.09560410e-01
3.48200000e+00   -4.18583689e-01
1.61800000e+00   1.39756180e-01
6.27000000e-01   2.40487708e+00
2.93400000e-01   -2.23192255e+00
1.31000000e-01   -8.64037177e-01
5.81500000e-02   1.45525790e+00
P 10 1.0
1.92500000e+02   1.34858000e-04
4.56400000e+01   1.14831710e-03
1.47500000e+01   5.84281610e-03
5.50300000e+00   2.10758283e-02
2.22200000e+00   6.11960100e-02
9.59000000e-01   1.53071076e-01
4.31400000e-01   2.94324162e-01
1.96900000e-01   3.64270436e-01
9.03300000e-02   2.58530924e-01
4.06600000e-02   7.17053089e-02
P 10 1.0
1.92500000e+02   1.03811900e-04
4.56400000e+01   1.00297990e-03
1.47500000e+01   4.40275030e-03
5.50300000e+00   1.59590908e-02
2.22200000e+00   1.40866372e-02
9.59000000e-01   2.03602506e-01
4.31400000e-01   6.40561072e-01
1.96900000e-01   1.38043328e-02
9.03300000e-02   -7.23955149e-01
4.06600000e-02   -2.86154751e-01
P 10 1.0
1.92500000e+02   3.90296000e-04
4.56400000e+01   3.58723880e-03
```

```
1.47500000e+01   1.81614638e-02
5.50300000e+00   7.80319375e-02
2.22200000e+00   2.45541210e-01
9.59000000e-01   8.29640072e-01
4.31400000e-01   -2.88444587e-01
1.96900000e-01   -1.15757654e+00
9.03300000e-02   5.47808333e-01
4.06600000e-02   3.67890885e-01
D 5 1.0
2.88600000e+00   1.24864080e-02
1.26700000e+00   6.46166325e-02
5.56000000e-01   3.52364034e-01
2.44000000e-01   4.91827218e-01
1.07000000e-01   2.48523180e-01
D 5 1.0
2.88600000e+00   -4.49367119e-02
1.26700000e+00   2.53698751e-01
5.56000000e-01   8.65924761e-01
2.44000000e-01   -4.51243401e-01
1.07000000e-01   -5.80249322e-01
F 4 1.0
1.65100000e+00   2.61389620e-02
8.00200000e-01   3.04593559e-01
3.87800000e-01   4.97810565e-01
1.88000000e-01   3.21518619e-01
****
-C
S 16 1.0
3.12100000e+05   5.68880000e-06
4.67400000e+04   4.42122000e-05
1.06400000e+04   2.32471700e-04
3.01300000e+03   9.81398300e-04
9.82800000e+02   3.56048580e-03
3.54800000e+02   1.14692478e-02
1.38400000e+02   3.30825487e-02
5.73500000e+01   8.42739202e-02
2.49200000e+01   1.81183521e-01
1.12300000e+01   3.05872661e-01
5.20100000e+00   3.42899662e-01
2.42600000e+00   1.78520312e-01
9.67300000e-01   1.74929057e-02
4.45600000e-01   -6.93818560e-03
1.97100000e-01   -4.58959260e-03
8.63500000e-02   -1.77870100e-03
S 16 1.0
3.12100000e+05   1.15170000e-06
4.67400000e+04   8.94010000e-06
1.06400000e+04   4.71067000e-05
3.01300000e+03   1.98530500e-04
9.82800000e+02   7.24115900e-04
3.54800000e+02   2.33730310e-03
1.38400000e+02   6.86927270e-03
5.73500000e+01   1.79514067e-02
2.49200000e+01   4.14871653e-02
1.12300000e+01   7.95916232e-02
5.20100000e+00   1.25824978e-01
2.42600000e+00   1.06822824e-01
9.67300000e-01   -1.28639081e-01
4.45600000e-01   -4.39866541e-01
1.97100000e-01   -4.46202571e-01
8.63500000e-02   -1.39613780e-01
S 16 1.0
3.12100000e+05   1.46150000e-06
4.67400000e+04   1.13996000e-05
1.06400000e+04   5.96491000e-05
3.01300000e+03   2.53756100e-04
9.82800000e+02   9.15187600e-04
3.54800000e+02   2.99970750e-03
1.38400000e+02   8.69022700e-03
5.73500000e+01   2.33513200e-02
2.49200000e+01   5.32963047e-02
```

```
1.12300000e+01   1.07142134e-01
5.20100000e+00   1.71571164e-01
2.42600000e+00   1.81050614e-01
9.67300000e-01   -6.61896970e-01
4.45600000e-01   -9.41374990e-01
1.97100000e-01   6.89663676e-01
8.63500000e-02   7.07911640e-01
S 16 1.0
3.12100000e+05   -2.07290000e-06
4.67400000e+04   -1.52244000e-05
1.06400000e+04   -8.68378000e-05
3.01300000e+03   -3.29424800e-04
9.82800000e+02   -1.37229620e-03
3.54800000e+02   -3.77954040e-03
1.38400000e+02   -1.36469384e-02
5.73500000e+01   -2.90748718e-02
2.49200000e+01   -9.39954201e-02
1.12300000e+01   -1.27039889e-01
5.20100000e+00   -3.90368865e-01
2.42600000e+00   9.67690209e-02
9.67300000e-01   2.18317118e+00
4.45600000e-01   -1.63805873e+00
1.97100000e-01   -1.36613182e+00
8.63500000e-02   1.60111801e+00
P 10 1.0
2.95200000e+02   1.42953600e-04
6.99800000e+01   1.22489070e-03
2.26400000e+01   6.35892390e-03
8.48500000e+00   2.35884808e-02
3.45900000e+00   6.96777731e-02
1.50400000e+00   1.66446415e-01
6.78300000e-01   2.93356499e-01
3.08700000e-01   3.51201757e-01
1.40000000e-01   2.56386887e-01
6.17800000e-02   7.62169165e-02
P 10 1.0
2.95200000e+02   1.64119100e-04
6.99800000e+01   1.50145510e-03
2.26400000e+01   7.37691910e-03
8.48500000e+00   2.84317842e-02
3.45900000e+00   6.74783713e-02
1.50400000e+00   2.85759124e-01
6.78300000e-01   5.58607497e-01
3.08700000e-01   -8.98519083e-02
1.40000000e-01   -6.54650765e-01
6.17800000e-02   -2.60439145e-01
P 10 1.0
2.95200000e+02   4.06304700e-04
6.99800000e+01   3.59675900e-03
2.26400000e+01   1.97550020e-02
8.48500000e+00   8.25675839e-02
3.45900000e+00   2.83930168e-01
1.50400000e+00   7.77020255e-01
6.78300000e-01   -4.43564826e-01
3.08700000e-01   -1.03884454e+00
1.40000000e-01   5.73532240e-01
6.17800000e-02   3.86981582e-01
D 5 1.0
4.54200000e+00   1.43889048e-02
1.97900000e+00   7.13989420e-02
8.62100000e-01   3.57076052e-01
3.75600000e-01   4.80335413e-01
1.63600000e-01   2.55117645e-01
D 5 1.0
4.54200000e+00   -7.63546200e-04
1.97900000e+00   3.07148921e-01
8.62100000e-01   7.74419900e-01
3.75600000e-01   -4.24221517e-01
1.63600000e-01   -5.69104609e-01
F 4 1.0
2.63100000e+00   3.34914704e-02
```

```
1.25500000e+00   2.98354910e-01
5.98800000e-01   5.16839650e-01
2.85700000e-01   3.06133004e-01
****
-N
S 16 1.0
4.32300000e+05   5.59360000e-06
6.47000000e+04   4.35138000e-05
1.47200000e+04   2.28928700e-04
4.17000000e+03   9.65017100e-04
1.36100000e+03   3.50219040e-03
4.91200000e+02   1.12921163e-02
1.91600000e+02   3.26128265e-02
7.94100000e+01   8.32972697e-02
3.45300000e+01   1.79985658e-01
1.55800000e+01   3.05003513e-01
7.23200000e+00   3.41159312e-01
3.38200000e+00   1.77482697e-01
1.36900000e+00   1.98840957e-02
6.24800000e-01   -1.24663480e-03
2.74700000e-01   1.04010390e-03
1.19200000e-01   -1.26586000e-04
S 16 1.0
4.32300000e+05   1.23770000e-06
6.47000000e+04   9.61460000e-06
1.47200000e+04   5.07014000e-05
4.17000000e+03   2.13291100e-04
1.36100000e+03   7.78459400e-04
4.91200000e+02   2.51336340e-03
1.91600000e+02   7.39724800e-03
7.94100000e+01   1.93531475e-02
3.45300000e+01   4.49446014e-02
1.55800000e+01   8.62431945e-02
7.23200000e+00   1.34250976e-01
3.38200000e+00   1.06013878e-01
1.36900000e+00   -1.38417092e-01
6.24800000e-01   -4.39126760e-01
2.74700000e-01   -4.31343581e-01
1.19200000e-01   -1.50718609e-01
S 16 1.0
4.32300000e+05   1.39480000e-06
6.47000000e+04   1.09071000e-05
1.47200000e+04   5.69709000e-05
4.17000000e+03   2.42756300e-04
1.36100000e+03   8.72395100e-04
4.91200000e+02   2.87588280e-03
1.91600000e+02   8.29427670e-03
7.94100000e+01   2.25053948e-02
3.45300000e+01   5.12093507e-02
1.55800000e+01   1.04849727e-01
7.23200000e+00   1.64429024e-01
3.38200000e+00   1.71055872e-01
1.36900000e+00   -5.97499064e-01
6.24800000e-01   -9.10291398e-01
2.74700000e-01   5.23452769e-01
1.19200000e-01   8.20052144e-01
S 16 1.0
4.32300000e+05   -2.07200000e-06
6.47000000e+04   -1.53930000e-05
1.47200000e+04   -8.65570000e-05
4.17000000e+03   -3.34467200e-04
1.36100000e+03   -1.35998010e-03
4.91200000e+02   -3.86617150e-03
1.91600000e+02   -1.34702049e-02
7.94100000e+01   -3.00266728e-02
3.45300000e+01   -9.25316587e-02
1.55800000e+01   -1.38615171e-01
7.23200000e+00   -3.82493166e-01
3.38200000e+00   9.54870859e-02
1.36900000e+00   2.04696644e+00
6.24800000e-01   -1.30083367e+00
```

```
2.74700000e-01   -1.61851336e+00
1.19200000e-01   1.64264012e+00
P 10 1.0
4.15900000e+02   1.49351500e-04
9.86100000e+01   1.28527800e-03
3.19200000e+01   6.74821670e-03
1.20000000e+01   2.54480843e-02
4.91900000e+00   7.55743643e-02
2.14800000e+00   1.75828080e-01
9.69600000e-01   2.96219513e-01
4.39900000e-01   3.44109940e-01
1.97800000e-01   2.50361577e-01
8.60300000e-02   7.63931220e-02
P 10 1.0
4.15900000e+02   1.95109000e-04
9.86100000e+01   1.75701950e-03
3.19200000e+01   8.99352330e-03
1.20000000e+01   3.54017865e-02
4.91900000e+00   9.70144192e-02
2.14800000e+00   3.24562521e-01
9.69600000e-01   5.07018547e-01
4.39900000e-01   -1.45468019e-01
1.97800000e-01   -6.20130974e-01
8.60300000e-02   -2.42720658e-01
P 10 1.0
4.15900000e+02   4.16269200e-04
9.86100000e+01   3.64253630e-03
3.19200000e+01   2.08775389e-02
1.20000000e+01   8.69515693e-02
4.91900000e+00   3.01954565e-01
2.14800000e+00   7.48192432e-01
9.69600000e-01   -5.36601609e-01
4.39900000e-01   -9.62402874e-01
1.97800000e-01   5.97938688e-01
8.60300000e-02   3.81282295e-01
D 5 1.0
6.71700000e+00   1.53532025e-02
2.89600000e+00   7.39377840e-02
1.24900000e+00   3.61114918e-01
5.38000000e-01   4.78400826e-01
2.32000000e-01   2.54877565e-01
D 5 1.0
6.71700000e+00   1.33057296e-02
2.89600000e+00   3.12470144e-01
1.24900000e+00   7.43203706e-01
5.38000000e-01   -3.99952197e-01
2.32000000e-01   -5.77366313e-01
F 4 1.0
3.82900000e+00   3.62155054e-02
1.79500000e+00   3.00102167e-01
8.41000000e-01   5.30147509e-01
3.94000000e-01   2.93011802e-01
****
-O
S 16 1.0
5.70800000e+05   5.54770000e-06
8.54800000e+04   4.31114000e-05
1.94600000e+04   2.26687800e-04
5.51200000e+03   9.56446100e-04
1.79800000e+03   3.47348060e-03
6.48900000e+02   1.11986722e-02
2.53100000e+02   3.23902914e-02
1.04900000e+02   8.28666917e-02
4.56500000e+01   1.79599915e-01
2.06200000e+01   3.05252295e-01
9.58700000e+00   3.40941604e-01
4.49300000e+00   1.77462875e-01
1.83700000e+00   2.04404056e-02
8.34900000e-01   -1.12254760e-03
3.65800000e-01   9.01550300e-04
1.57000000e-01   -1.42089000e-04
```

```
S 16 1.0
5.70800000e+05   1.26190000e-06
8.54800000e+04   9.79420000e-06
1.94600000e+04   5.16082000e-05
5.51200000e+03   2.17376000e-04
1.79800000e+03   7.93634000e-04
6.48900000e+02   2.56368280e-03
2.53100000e+02   7.55406490e-03
1.04900000e+02   1.98252847e-02
4.56500000e+01   4.62226113e-02
2.06200000e+01   8.93623473e-02
9.58700000e+00   1.38343462e-01
4.49300000e+00   1.05181307e-01
1.83700000e+00   -1.49165905e-01
8.34900000e-01   -4.43538639e-01
3.65800000e-01   -4.30421768e-01
1.57000000e-01   -1.39284763e-01
S 16 1.0
5.70800000e+05   -1.49560000e-06
8.54800000e+04   -1.16782000e-05
1.94600000e+04   -6.10049000e-05
5.51200000e+03   -2.59966200e-04
1.79800000e+03   -9.35972900e-04
6.48900000e+02   -3.08166350e-03
2.53100000e+02   -8.91883060e-03
1.04900000e+02   -2.42145842e-02
4.56500000e+01   -5.55592008e-02
2.06200000e+01   -1.14527593e-01
9.58700000e+00   -1.82885364e-01
4.49300000e+00   -1.75395843e-01
1.83700000e+00   7.13860208e-01
8.34900000e-01   8.83104152e-01
3.65800000e-01   -7.22845012e-01
1.57000000e-01   -6.62903050e-01
S 16 1.0
5.70800000e+05   -2.10120000e-06
8.54800000e+04   -1.53046000e-05
1.94600000e+04   -8.83188000e-05
5.51200000e+03   -3.29606500e-04
1.79800000e+03   -1.40118000e-03
6.48900000e+02   -3.76970610e-03
2.53100000e+02   -1.40318733e-02
1.04900000e+02   -2.89525787e-02
4.56500000e+01   -9.82062901e-02
2.06200000e+01   -1.30740865e-01
9.58700000e+00   -4.33993969e-01
4.49300000e+00   1.77427381e-01
1.83700000e+00   2.18658844e+00
8.34900000e-01   -1.84886022e+00
3.65800000e-01   -1.06263353e+00
1.57000000e-01   1.46444115e+00
P 10 1.0
5.25600000e+02   1.67663100e-04
1.24600000e+02   1.44341600e-03
4.03400000e+01   7.59732890e-03
1.51800000e+01   2.88049894e-02
6.24500000e+00   8.49419814e-02
2.73200000e+00   1.89963117e-01
1.22700000e+00   2.99047865e-01
5.49200000e-01   3.32056996e-01
2.41800000e-01   2.41992085e-01
1.02500000e-01   8.36337245e-02
P 10 1.0
5.25600000e+02   2.04132700e-04
1.24600000e+02   1.79921620e-03
4.03400000e+01   9.35497490e-03
1.51800000e+01   3.61612981e-02
6.24500000e+00   1.04785215e-01
2.73200000e+00   3.13224909e-01
1.22700000e+00   4.46483313e-01
5.49200000e-01   -1.16219022e-01
```

```
2.41800000e-01   -5.39812159e-01
1.02500000e-01   -3.46845177e-01
P 10 1.0
5.25600000e+02   3.39750900e-04
1.24600000e+02   3.05971660e-03
4.03400000e+01   1.66722601e-02
1.51800000e+01   6.91106710e-02
6.24500000e+00   2.24673930e-01
2.73200000e+00   6.38777042e-01
1.22700000e+00   -2.24450373e-01
5.49200000e-01   -9.38196350e-01
2.41800000e-01   1.16475904e-01
1.02500000e-01   7.36644552e-01
D 5 1.0
8.25300000e+00   2.04005719e-02
3.59700000e+00   9.41369585e-02
1.56800000e+00   3.96477091e-01
6.84000000e-01   4.55360161e-01
2.98000000e-01   2.20405730e-01
D 5 1.0
8.25300000e+00   2.53040956e-02
3.59700000e+00   3.69383220e-01
1.56800000e+00   6.50550865e-01
6.84000000e-01   -4.11645852e-01
2.98000000e-01   -5.80497766e-01
F 4 1.0
5.43000000e+00   3.35228290e-02
2.41600000e+00   2.88431165e-01
1.07500000e+00   5.27243780e-01
4.78000000e-01   3.29660778e-01
****
-F
S 16 1.0
7.23500000e+05   5.55530000e-06
1.08400000e+05   4.31553000e-05
2.46800000e+04   2.26888200e-04
6.99000000e+03   9.57552400e-04
2.28200000e+03   3.46841300e-03
8.24600000e+02   1.11796501e-02
3.21800000e+02   3.23122990e-02
1.33500000e+02   8.27520288e-02
5.81100000e+01   1.79778821e-01
2.62800000e+01   3.05381571e-01
1.22400000e+01   3.39966883e-01
5.74700000e+00   1.76460081e-01
2.36500000e+00   2.11818400e-02
1.07100000e+00   2.01810000e-04
4.68100000e-01   2.01228700e-03
1.99400000e-01   1.86918000e-04
S 16 1.0
7.23500000e+05   1.30260000e-06
1.08400000e+05   1.01077000e-05
2.46800000e+04   5.32435000e-05
6.99000000e+03   2.24379700e-04
2.28200000e+03   8.16840000e-04
8.24600000e+02   2.63904770e-03
3.21800000e+02   7.76806640e-03
1.33500000e+02   2.04244295e-02
5.81100000e+01   4.77452371e-02
2.62800000e+01   9.24737947e-02
1.22400000e+01   1.41959438e-01
5.74700000e+00   1.04067345e-01
2.36500000e+00   -1.57152841e-01
1.07100000e+00   -4.45384340e-01
4.68100000e-01   -4.28296901e-01
1.99400000e-01   -1.33422900e-01
S 16 1.0
7.23500000e+05   -1.58120000e-06
1.08400000e+05   -1.23348000e-05
2.46800000e+04   -6.44826000e-05
6.99000000e+03   -2.74554700e-04
```

```
2.28200000e+03   -9.87382600e-04
8.24600000e+02   -3.24507940e-03
3.21800000e+02   -9.40855790e-03
1.33500000e+02   -2.55267961e-02
5.81100000e+01   -5.90490461e-02
2.62800000e+01   -1.21829593e-01
1.22400000e+01   -1.97106235e-01
5.74700000e+00   -1.74313349e-01
2.36500000e+00   8.01133928e-01
1.07100000e+00   8.43347803e-01
4.68100000e-01   -8.43766683e-01
1.99400000e-01   -5.56541527e-01
S 16 1.0
7.23500000e+05   -2.21380000e-06
1.08400000e+05   -1.57194000e-05
2.46800000e+04   -9.39462000e-05
6.99000000e+03   -3.34204000e-04
2.28200000e+03   -1.50315470e-03
8.24600000e+02   -3.74960300e-03
3.21800000e+02   -1.52245069e-02
1.33500000e+02   -2.82133583e-02
5.81100000e+01   -1.08737960e-01
2.62800000e+01   -1.21479459e-01
1.22400000e+01   -5.12410504e-01
5.74700000e+00   3.08944627e-01
2.36500000e+00   2.31713096e+00
1.07100000e+00   -2.43988488e+00
4.68100000e-01   -4.17998152e-01
1.99400000e-01   1.21555675e+00
P 10 1.0
6.60000000e+02   1.78229200e-04
1.56400000e+02   1.53635190e-03
5.06400000e+01   8.12284340e-03
1.90800000e+01   3.09453269e-02
7.87200000e+00   9.08038263e-02
3.44900000e+00   1.97614180e-01
1.54500000e+00   3.00590618e-01
6.86400000e-01   3.26422450e-01
2.98600000e-01   2.39668526e-01
1.24500000e-01   8.12040964e-02
P 10 1.0
6.60000000e+02   2.34347800e-04
1.56400000e+02   2.07242600e-03
5.06400000e+01   1.08206813e-02
1.90800000e+01   4.23975838e-02
7.87200000e+00   1.25261742e-01
3.44900000e+00   3.55755402e-01
1.54500000e+00   4.26059781e-01
6.86400000e-01   -1.96265343e-01
2.98600000e-01   -5.53936553e-01
1.24500000e-01   -2.62152264e-01
P 10 1.0
6.60000000e+02   3.96333300e-04
1.56400000e+02   3.38304610e-03
5.06400000e+01   1.96902218e-02
1.90800000e+01   7.73666944e-02
7.87200000e+00   2.75392562e-01
3.44900000e+00   6.85583994e-01
1.54500000e+00   -4.98790106e-01
6.86400000e-01   -8.78659405e-01
2.98600000e-01   4.22153705e-01
1.24500000e-01   5.25305084e-01
D 5 1.0
1.05730000e+01   2.27863167e-02
4.61300000e+00   1.01470167e-01
2.01300000e+00   4.10653684e-01
8.78000000e-01   4.49088535e-01
3.83000000e-01   2.03354826e-01
D 5 1.0
1.05730000e+01   3.38687384e-02
4.61300000e+00   3.93252355e-01
```

```
2.01300000e+00   6.20292128e-01
8.78000000e-01   -4.43973787e-01
3.83000000e-01   -5.56278968e-01
F 4 1.0
7.56300000e+00   2.56618573e-02
3.33000000e+00   2.72433403e-01
1.46600000e+00   5.42962543e-01
6.45000000e-01   3.33550324e-01
****
-Ne
S 16 1.0
9.02400000e+05   5.50710000e-06
1.35100000e+05   4.28234000e-05
3.07500000e+04   2.25142500e-04
8.71000000e+03   9.50160200e-04
2.84200000e+03   3.44718850e-03
1.02600000e+03   1.11254471e-02
4.00100000e+02   3.22056752e-02
1.65900000e+02   8.25989133e-02
7.22100000e+01   1.79905642e-01
3.26600000e+01   3.06052078e-01
1.52200000e+01   3.40125584e-01
7.14900000e+00   1.76168221e-01
2.95700000e+00   2.10152784e-02
1.33500000e+00   -5.07437900e-04
5.81600000e-01   1.05785740e-03
2.46300000e-01   -5.98773000e-05
S 16 1.0
9.02400000e+05   1.29630000e-06
1.35100000e+05   1.00690000e-05
3.07500000e+04   5.30405000e-05
8.71000000e+03   2.23517100e-04
2.84200000e+03   8.15055900e-04
1.02600000e+03   2.63688220e-03
4.00100000e+02   7.77600830e-03
1.65900000e+02   2.04887059e-02
7.22100000e+01   4.80966546e-02
3.26600000e+01   9.35683962e-02
1.52200000e+01   1.43356072e-01
7.14900000e+00   1.02155876e-01
2.95700000e+00   -1.62934767e-01
1.33500000e+00   -4.46676169e-01
5.81600000e-01   -4.25862512e-01
2.46300000e-01   -1.30306294e-01
S 16 1.0
9.02400000e+05   -1.61150000e-06
1.35100000e+05   -1.25800000e-05
3.07500000e+04   -6.57939000e-05
8.71000000e+03   -2.79967400e-04
2.84200000e+03   -1.00926080e-03
1.02600000e+03   -3.31830470e-03
4.00100000e+02   -9.64836690e-03
1.65900000e+02   -2.61882549e-02
7.22100000e+01   -6.09198166e-02
3.26600000e+01   -1.26041391e-01
1.52200000e+01   -2.05488897e-01
7.14900000e+00   -1.71923360e-01
2.95700000e+00   8.48906100e-01
1.33500000e+00   8.13169457e-01
5.81600000e-01   -9.01853688e-01
2.46300000e-01   -4.99925119e-01
S 16 1.0
9.02400000e+05   -2.27750000e-06
1.35100000e+05   -1.58878000e-05
3.07500000e+04   -9.74546000e-05
8.71000000e+03   -3.34413100e-04
2.84200000e+03   -1.57289810e-03
1.02600000e+03   -3.71614540e-03
4.00100000e+02   -1.60993168e-02
1.65900000e+02   -2.75130195e-02
7.22100000e+01   -1.16504533e-01
```

```
3.26600000e+01   -1.14236147e-01
1.52200000e+01   -5.76611990e-01
7.14900000e+00   4.17635713e-01
2.95700000e+00   2.38840962e+00
1.33500000e+00   -2.83819246e+00
5.81600000e-01   4.97789435e-02
2.46300000e-01   1.01516124e+00
P 10 1.0
8.15600000e+02   1.84770500e-04
1.93300000e+02   1.59410930e-03
6.26000000e+01   8.46535310e-03
2.36100000e+01   3.24030611e-02
9.76200000e+00   9.47184717e-02
4.28100000e+00   2.02624168e-01
1.91500000e+00   3.01794970e-01
8.47600000e-01   3.23166976e-01
3.66000000e-01   2.37138589e-01
1.51000000e-01   7.91176295e-02
P 10 1.0
8.15600000e+02   2.55486400e-04
1.93300000e+02   2.25791630e-03
6.26000000e+01   1.18809731e-02
2.36100000e+01   4.69683102e-02
9.76200000e+00   1.40960018e-01
4.28100000e+00   3.82289571e-01
1.91500000e+00   4.04957116e-01
8.47600000e-01   -2.48772519e-01
3.66000000e-01   -5.50555989e-01
1.51000000e-01   -2.14656548e-01
P 10 1.0
8.15600000e+02   4.49349300e-04
1.93300000e+02   3.64469480e-03
6.26000000e+01   2.25385482e-02
2.36100000e+01   8.42160085e-02
9.76200000e+00   3.25411552e-01
4.28100000e+00   7.22034611e-01
1.91500000e+00   -7.51414382e-01
8.47600000e-01   -7.61544619e-01
3.66000000e-01   6.36069389e-01
1.51000000e-01   3.27481185e-01
D 5 1.0
1.33170000e+01   2.47658998e-02
5.80300000e+00   1.06876555e-01
2.52900000e+00   4.20540693e-01
1.10200000e+00   4.44946874e-01
4.80000000e-01   1.90871284e-01
D 5 1.0
1.33170000e+01   4.02982534e-02
5.80300000e+00   4.04860485e-01
2.52900000e+00   5.99892024e-01
1.10200000e+00   -4.61442784e-01
4.80000000e-01   -5.44031357e-01
F 4 1.0
1.03560000e+01   1.50197139e-02
4.53800000e+00   2.51242680e-01
1.98900000e+00   5.75622078e-01
8.71000000e-01   3.19708463e-01
****
-Na
S 21 1.0
1.91870000e+06   2.70880000e-06
2.87270000e+05   2.10578000e-05
6.53820000e+04   1.10786800e-04
1.85230000e+04   4.67532600e-04
6.04540000e+03   1.70132840e-03
2.18420000e+03   5.51507460e-03
8.53110000e+02   1.62308700e-02
3.54690000e+02   4.31395655e-02
1.55330000e+02   1.01486023e-01
7.12340000e+01   1.99394305e-01
3.38350000e+01   3.08728896e-01
```

```
1.63490000e+01   3.06705230e-01
8.00820000e+00   1.38088158e-01
3.67130000e+00   2.28929786e-02
1.71180000e+00   1.32950975e-02
7.91470000e-01   1.36239139e-02
3.54790000e-01   3.73867660e-03
9.06640000e-02   7.44574000e-05
4.43130000e-02   -9.52170000e-06
2.19480000e-02   2.13706000e-05
1.08210000e-02   0.00000000e+00
S 21 1.0
1.91870000e+06   7.48500000e-07
2.87270000e+05   5.81770000e-06
6.53820000e+04   3.06178000e-05
1.85230000e+04   1.29225900e-04
6.04540000e+03   4.70970800e-04
2.18420000e+03   1.53073460e-03
8.53110000e+02   4.54125400e-03
3.54690000e+02   1.22699314e-02
1.55330000e+02   2.99593063e-02
7.12340000e+01   6.33214284e-02
3.38350000e+01   1.14295475e-01
1.63490000e+01   1.54163786e-01
8.00820000e+00   8.93386546e-02
3.67130000e+00   -1.65977411e-01
1.71180000e+00   -4.38423509e-01
7.91470000e-01   -4.29104953e-01
3.54790000e-01   -1.29316724e-01
9.06640000e-02   4.40490600e-04
4.43130000e-02   -2.47939490e-03
2.19480000e-02   9.62642900e-04
1.08210000e-02   -4.04101000e-04
S 21 1.0
1.91870000e+06   1.09600000e-07
2.87270000e+05   8.51300000e-07
6.53820000e+04   4.48290000e-06
1.85230000e+04   1.89095000e-05
6.04540000e+03   6.89893000e-05
2.18420000e+03   2.24129900e-04
8.53110000e+02   6.66689500e-04
3.54690000e+02   1.80444930e-03
1.55330000e+02   4.44375000e-03
7.12340000e+01   9.49314110e-03
3.38350000e+01   1.76175702e-02
1.63490000e+01   2.47238067e-02
8.00820000e+00   1.50685595e-02
3.67130000e+00   -3.26908857e-02
1.71180000e+00   -9.05675951e-02
7.91470000e-01   -1.27595009e-01
3.54790000e-01   -1.22199518e-01
9.06640000e-02   2.66615947e-01
4.43130000e-02   5.62246346e-01
2.19480000e-02   2.79758335e-01
1.08210000e-02   1.84296293e-02
S 21 1.0
1.91870000e+06   -9.12100000e-07
2.87270000e+05   -7.05970000e-06
6.53820000e+04   -3.73862000e-05
1.85230000e+04   -1.56574600e-04
6.04540000e+03   -5.77045300e-04
2.18420000e+03   -1.85767720e-03
8.53110000e+02   -5.64162320e-03
3.54690000e+02   -1.53304856e-02
1.55330000e+02   -4.00233925e-02
7.12340000e+01   -8.43610950e-02
3.38350000e+01   -1.52656528e-01
1.63490000e+01   -2.12193144e-01
8.00820000e+00   -1.08260808e-01
3.67130000e+00   9.47407488e-01
1.71180000e+00   7.69196129e-01
7.91470000e-01   -9.53788381e-01
```

```
3.54790000e-01   -5.97900103e-01
9.06640000e-02   4.63503976e-02
4.43130000e-02   2.18619967e-01
2.19480000e-02   8.03942641e-02
1.08210000e-02   9.83466700e-03
S 21 1.0
1.91870000e+06   2.03180000e-06
2.87270000e+05   1.61964000e-05
6.53820000e+04   8.22300000e-05
1.85230000e+04   3.64715300e-04
6.04540000e+03   1.25846160e-03
2.18420000e+03   4.47591370e-03
8.53110000e+02   1.28756216e-02
3.54690000e+02   4.44201519e-02
1.55330000e+02   1.24991099e-01
7.12340000e+01   2.83631891e-01
3.38350000e+01   1.68789391e-01
1.63490000e+01   1.87955419e-01
8.00820000e+00   -8.57604112e-01
3.67130000e+00   -1.25413242e+00
1.71180000e+00   1.97935541e+00
7.91470000e-01   4.18323562e-01
3.54790000e-01   -1.26590249e+00
9.06640000e-02   -2.17394423e-01
4.43130000e-02   4.80560744e-01
2.19480000e-02   1.03859080e-03
1.08210000e-02   4.03416859e-02
P 15 1.0
3.04420000e+03   2.81041000e-05
7.20020000e+02   2.56512000e-04
2.34420000e+02   1.41776520e-03
8.96660000e+01   6.39168250e-03
3.66950000e+01   2.31537137e-02
1.62560000e+01   6.52632255e-02
7.65930000e+00   1.44068059e-01
3.71250000e+00   2.42657238e-01
1.79920000e+00   3.06650146e-01
8.62880000e-01   2.85522924e-01
4.01040000e-01   1.56904919e-01
1.77590000e-01   2.50223117e-02
8.07000000e-02   9.79528500e-04
3.67000000e-02   1.43310800e-04
1.67000000e-02   3.82855000e-05
P 15 1.0
3.04420000e+03   4.22160000e-05
7.20020000e+02   3.89098500e-04
2.34420000e+02   2.22141080e-03
8.96660000e+01   1.03861947e-02
3.66950000e+01   3.69679527e-02
1.62560000e+01   1.02091536e-01
7.65930000e+00   2.60145450e-01
3.71250000e+00   4.51233315e-01
1.79920000e+00   1.11611823e-01
8.62880000e-01   -4.76591870e-01
4.01040000e-01   -4.16152570e-01
1.77590000e-01   -9.94949247e-02
8.07000000e-02   -1.17309641e-02
3.67000000e-02   -3.91016680e-03
1.67000000e-02   2.09826000e-04
D 3 1.0
2.35000000e+00   8.48536377e-01
5.60000000e-01   2.80121913e-01
1.40000000e-01   -1.51540850e-02
****
-Mg
S 21 1.0
3.19910000e+06   1.79500000e-06
4.79000000e+05   1.39619000e-05
1.09010000e+05   7.34120000e-05
3.08780000e+04   3.10361400e-04
1.00750000e+04   1.12952010e-03
```

3.63790000e+03   3.68251510e-03
1.41950000e+03   1.09059255e-02
5.89350000e+02   2.95927497e-02
2.57530000e+02   7.21890156e-02
1.17170000e+02   1.55264953e-01
5.47620000e+01   2.72711082e-01
2.62880000e+01   3.34448771e-01
1.28600000e+01   2.19916844e-01
6.26040000e+00   4.96777160e-02
2.85140000e+00   2.76015740e-03
1.30580000e+00   8.14324200e-04
5.83310000e-01   7.60996000e-05
1.68490000e-01   2.36754000e-05
8.56460000e-02   -2.06013000e-05
4.50770000e-02   1.10197000e-05
2.19340000e-02   -2.70940000e-06
S 21 1.0
3.19910000e+06   4.53700000e-07
4.79000000e+05   3.52560000e-06
1.09010000e+05   1.85673000e-05
3.08780000e+04   7.83671000e-05
1.00750000e+04   2.86187000e-04
3.63790000e+03   9.32526900e-04
1.41950000e+03   2.78865710e-03
5.89350000e+02   7.63924590e-03
2.57530000e+02   1.93013967e-02
1.17170000e+02   4.38512116e-02
5.47620000e+01   8.80107485e-02
2.62880000e+01   1.37273971e-01
1.28600000e+01   1.36976362e-01
6.26040000e+00   -4.20379328e-02
2.85140000e+00   -3.72787567e-01
1.30580000e+00   -5.19014763e-01
5.83310000e-01   -2.11210262e-01
1.68490000e-01   -2.78178800e-03
8.56460000e-02   -1.58740920e-03
4.50770000e-02   1.42578440e-03
2.19340000e-02   -4.18862200e-04
S 21 1.0
3.19910000e+06   8.75000000e-08
4.79000000e+05   6.78800000e-07
1.09010000e+05   3.58080000e-06
3.08780000e+04   1.50794000e-05
1.00750000e+04   5.52299000e-05
3.63790000e+03   1.79363900e-04
1.41950000e+03   5.38857700e-04
5.89350000e+02   1.47013010e-03
2.57530000e+02   3.74429760e-03
1.17170000e+02   8.48067780e-03
5.47620000e+01   1.73277526e-02
2.62880000e+01   2.71678567e-02
1.28600000e+01   2.86523167e-02
6.26040000e+00   -1.04754726e-02
2.85140000e+00   -8.57137348e-02
1.30580000e+00   -1.68548085e-01
5.83310000e-01   -1.67989351e-01
1.68490000e-01   2.25842419e-01
8.56460000e-02   4.54327383e-01
4.50770000e-02   3.90225285e-01
2.19340000e-02   8.48474620e-02
S 21 1.0
3.19910000e+06   2.80000000e-08
4.79000000e+05   2.39500000e-07
1.09010000e+05   1.09450000e-06
3.08780000e+04   5.54920000e-06
1.00750000e+04   1.60017000e-05
3.63790000e+03   6.91337000e-05
1.41950000e+03   1.45838200e-04
5.89350000e+02   5.99166500e-04
2.57530000e+02   9.15827800e-04
1.17170000e+02   3.74560390e-03

```
5.47620000e+01   3.36867780e-03
2.62880000e+01   1.48488419e-02
1.28600000e+01   -2.94189980e-03
6.26040000e+00   2.03771606e-02
2.85140000e+00   -7.55531699e-02
1.30580000e+00   3.31803053e-02
5.83310000e-01   -2.10262284e-01
1.68490000e-01   8.01690086e-01
8.56460000e-02   -2.13440679e+00
4.50770000e-02   4.23794935e+00
2.19340000e-02   -3.17425812e+00
S 21 1.0
3.19910000e+06   6.47000000e-08
4.79000000e+05   4.37600000e-07
1.09010000e+05   2.80090000e-06
3.08780000e+04   9.06210000e-06
1.00750000e+04   4.57595000e-05
3.63790000e+03   9.86988000e-05
1.41950000e+03   4.76389300e-04
5.89350000e+02   7.14558500e-04
2.57530000e+02   3.59490610e-03
1.17170000e+02   3.28477410e-03
5.47620000e+01   1.91881365e-02
2.62880000e+01   2.27814460e-03
1.28600000e+01   5.66985070e-02
6.26040000e+00   -7.72645697e-02
2.85140000e+00   7.66595026e-02
1.30580000e+00   -3.92945801e-01
5.83310000e-01   3.20841673e-01
1.68490000e-01   -2.18148721e+00
8.56460000e-02   8.02815458e+00
4.50770000e-02   -8.92239058e+00
2.19340000e-02   3.15527468e+00
P 15 1.0
4.15780000e+03   2.36086000e-05
9.84530000e+02   2.17246800e-04
3.20090000e+02   1.20606720e-03
1.21730000e+02   5.61654260e-03
4.98920000e+01   2.02158577e-02
2.25420000e+01   5.66094004e-02
1.07490000e+01   1.30038527e-01
5.28990000e+00   2.29131731e-01
2.59960000e+00   3.14520771e-01
1.25180000e+00   3.05194501e-01
5.92460000e-01   1.53594898e-01
2.69410000e-01   2.25671416e-02
1.22500000e-01   1.13636480e-03
5.57000000e-02   2.59697200e-04
2.53000000e-02   1.37661000e-05
P 15 1.0
4.15780000e+03   1.41670000e-06
9.84530000e+02   1.92918000e-05
3.20090000e+02   7.35564000e-05
1.21730000e+02   4.91881800e-04
4.98920000e+01   1.25317580e-03
2.25420000e+01   5.17293210e-03
1.07490000e+01   7.55102310e-03
5.28990000e+00   2.36912477e-02
2.59960000e+00   1.20432758e-02
1.25180000e+00   5.26499427e-02
5.92460000e-01   -4.00496777e-02
2.69410000e-01   8.31726078e-02
1.22500000e-01   -3.81744210e-01
5.57000000e-02   3.58175916e-01
2.53000000e-02   -1.11581282e+00
D 3 1.0
3.36000000e+00   2.16295093e-02
8.00000000e-01   4.97381850e-03
2.00000000e-01   9.96002292e-01
****
-Al
```

```
S 21 1.0
3.65200000e+06   1.86370000e-06
5.46800000e+05   1.44635000e-05
1.24500000e+05   7.61824000e-05
3.54400000e+04   3.15823500e-04
1.18400000e+04   1.09738740e-03
4.43400000e+03   3.36970980e-03
1.81200000e+03   9.32218810e-03
7.91500000e+02   2.37993006e-02
3.61000000e+02   5.68193211e-02
1.69500000e+02   1.22468847e-01
8.16800000e+01   2.23897590e-01
4.02800000e+01   3.13444677e-01
2.02500000e+01   2.74977258e-01
1.02300000e+01   1.10573391e-01
4.80200000e+00   1.19196229e-02
2.33900000e+00   6.36932200e-04
1.16300000e+00   4.48769200e-04
5.88200000e-01   -3.80120000e-05
2.31100000e-01   5.93791000e-05
1.02700000e-01   5.87612000e-05
4.52100000e-02   2.61452000e-05
S 21 1.0
3.65200000e+06   4.84800000e-07
5.46800000e+05   3.76070000e-06
1.24500000e+05   1.98230000e-05
3.54400000e+04   8.21362000e-05
1.18400000e+04   2.85993600e-04
4.43400000e+03   8.78972800e-04
1.81200000e+03   2.44968390e-03
7.91500000e+02   6.31372180e-03
3.61000000e+02   1.54946400e-02
1.69500000e+02   3.49794080e-02
8.16800000e+01   7.08150170e-02
4.02800000e+01   1.19490376e-01
2.02500000e+01   1.48928590e-01
1.02300000e+01   5.90936008e-02
4.80200000e+00   -2.16754778e-01
2.33900000e+00   -4.76900610e-01
1.16300000e+00   -3.76454511e-01
5.88200000e-01   -8.70507052e-02
2.31100000e-01   -1.25539970e-03
1.02700000e-01   2.27551840e-03
4.52100000e-02   5.97803400e-04
S 21 1.0
3.65200000e+06   1.10700000e-07
5.46800000e+05   8.59100000e-07
1.24500000e+05   4.52550000e-06
3.54400000e+04   1.87694000e-05
1.18400000e+04   6.52773000e-05
4.43400000e+03   2.00987800e-04
1.81200000e+03   5.59222300e-04
7.91500000e+02   1.44638300e-03
3.61000000e+02   3.54559880e-03
1.69500000e+02   8.06441740e-03
8.16800000e+01   1.63827254e-02
4.02800000e+01   2.82463510e-02
2.02500000e+01   3.59202031e-02
1.02300000e+01   1.51042507e-02
4.80200000e+00   -6.05797083e-02
2.33900000e+00   -1.50845870e-01
1.16300000e+00   -2.19775043e-01
5.88200000e-01   -9.45734630e-02
2.31100000e-01   4.00909611e-01
1.02700000e-01   6.09838548e-01
4.52100000e-02   1.81242505e-01
S 21 1.0
3.65200000e+06   1.49600000e-07
5.46800000e+05   1.17350000e-06
1.24500000e+05   6.08730000e-06
3.54400000e+04   2.57771000e-05
```

1.18400000e+04   8.71996000e-05
4.43400000e+03   2.78364200e-04
1.81200000e+03   7.39521600e-04
7.91500000e+02   2.02563180e-03
3.61000000e+02   4.64223630e-03
1.69500000e+02   1.14436286e-02
8.16800000e+01   2.11580673e-02
4.02800000e+01   4.14650571e-02
2.02500000e+01   4.35211688e-02
1.02300000e+01   3.24891435e-02
4.80200000e+00   -1.19650402e-01
2.33900000e+00   -1.60003196e-01
1.16300000e+00   -5.19835133e-01
5.88200000e-01   1.66599052e-01
2.31100000e-01   1.84383214e+00
1.02700000e-01   -8.37723991e-01
4.52100000e-02   -7.63734360e-01
S 21 1.0
3.65200000e+06   2.06100000e-07
5.46800000e+05   1.71530000e-06
1.24500000e+05   8.14900000e-06
3.54400000e+04   3.87797000e-05
1.18400000e+04   1.11847200e-04
4.43400000e+03   4.37120400e-04
1.81200000e+03   8.87074100e-04
7.91500000e+02   3.35299490e-03
3.61000000e+02   5.17038220e-03
1.69500000e+02   2.00182998e-02
8.16800000e+01   2.08212154e-02
4.02800000e+01   8.17328395e-02
2.02500000e+01   1.53743546e-02
1.02300000e+01   1.37609815e-01
4.80200000e+00   -4.14141440e-01
2.33900000e+00   2.14720916e-01
1.16300000e+00   -2.09270766e+00
5.88200000e-01   3.51390605e+00
2.31100000e-01   -8.39589520e-01
1.02700000e-01   -2.19068436e+00
4.52100000e-02   1.83762412e+00
P 14 1.0
2.88400000e+03   6.38513000e-05
6.83200000e+02   5.63618100e-04
2.22000000e+02   3.17209690e-03
8.48200000e+01   1.32523750e-02
3.58100000e+01   4.33836653e-02
1.62200000e+01   1.12060310e-01
7.70200000e+00   2.18014276e-01
3.74100000e+00   3.11943174e-01
1.83100000e+00   3.16943976e-01
8.87800000e-01   1.78925133e-01
3.98900000e-01   3.09903761e-02
1.71800000e-01   1.20673500e-04
7.29800000e-02   6.68944900e-04
3.06900000e-02   -1.26314900e-04
P 14 1.0
2.88400000e+03   1.20316000e-05
6.83200000e+02   1.06831000e-04
2.22000000e+02   5.99099500e-04
8.48200000e+01   2.52558480e-03
3.58100000e+01   8.29114610e-03
1.62200000e+01   2.18958429e-02
7.70200000e+00   4.30126547e-02
3.74100000e+00   6.33327606e-02
1.83100000e+00   6.77298350e-02
8.87800000e-01   4.17050279e-02
3.98900000e-01   -1.30263600e-01
1.71800000e-01   -4.20265513e-01
7.29800000e-02   -4.49846925e-01
3.06900000e-02   -1.54440166e-01
P 14 1.0
2.88400000e+03   1.42841000e-05

```
6.83200000e+02   1.36056600e-04
2.22000000e+02   7.11550200e-04
8.48200000e+01   3.21614900e-03
3.58100000e+01   9.73602490e-03
1.62200000e+01   2.78786993e-02
7.70200000e+00   4.98714840e-02
3.74100000e+00   8.77150859e-02
1.83100000e+00   7.07128658e-02
8.87800000e-01   1.57537627e-01
3.98900000e-01   -4.80977977e-01
1.71800000e-01   -8.34821097e-01
7.29800000e-02   7.14980598e-01
3.06900000e-02   4.86129239e-01
P 14 1.0
2.88400000e+03   2.38482000e-05
6.83200000e+02   1.94281400e-04
2.22000000e+02   1.18801140e-03
8.48200000e+01   4.59894110e-03
3.58100000e+01   1.63337058e-02
1.62200000e+01   3.82089444e-02
7.70200000e+00   9.42740455e-02
3.74100000e+00   1.11042689e-01
1.83100000e+00   1.90893453e-01
8.87800000e-01   1.36933213e-01
3.98900000e-01   -1.87225306e+00
1.71800000e-01   1.65161071e+00
7.29800000e-02   2.22042391e-01
3.06900000e-02   -8.23645039e-01
D 5 1.0
2.21430000e+00   1.78097303e-02
9.44900000e-01   1.04024313e-02
4.03200000e-01   2.59473672e-01
1.72100000e-01   5.57049317e-01
7.34300000e-02   3.06491357e-01
D 5 1.0
2.21430000e+00   8.12809320e-03
9.44900000e-01   -7.80708823e-02
4.03200000e-01   8.75507330e-01
1.72100000e-01   7.40558985e-02
7.34300000e-02   -9.04595539e-01
F 4 1.0
8.75600000e-01   -1.56499578e-02
4.47200000e-01   2.90304534e-01
2.28400000e-01   4.53639208e-01
1.16700000e-01   3.91701193e-01
****
-Si
S 21 1.0
4.46500000e+06   1.74730000e-06
6.68500000e+05   1.35737000e-05
1.52200000e+05   7.14074000e-05
4.33000000e+04   2.97258300e-04
1.44100000e+04   1.03828330e-03
5.39400000e+03   3.17467540e-03
2.21200000e+03   8.73249580e-03
9.68100000e+02   2.23831560e-02
4.41200000e+02   5.37277388e-02
2.07100000e+02   1.16649627e-01
9.98000000e+01   2.15980191e-01
4.92400000e+01   3.09568372e-01
2.47400000e+01   2.83949358e-01
1.24700000e+01   1.22238608e-01
5.79500000e+00   1.41879507e-02
2.83000000e+00   2.86092300e-04
1.40700000e+00   5.80965400e-04
6.99500000e-01   -1.36262400e-04
3.08300000e-01   1.13232400e-04
1.38500000e-01   5.83027000e-05
6.14500000e-02   3.34760000e-05
S 21 1.0
4.46500000e+06   4.65600000e-07
```

```
6.68500000e+05   3.61540000e-06
1.52200000e+05   1.90351000e-05
4.33000000e+04   7.91894000e-05
1.44100000e+04   2.77208300e-04
5.39400000e+03   8.48109500e-04
2.21200000e+03   2.35032400e-03
9.68100000e+02   6.07697460e-03
4.41200000e+02   1.49870123e-02
2.07100000e+02   3.40090641e-02
9.98000000e+01   6.95329490e-02
4.92400000e+01   1.19129204e-01
2.47400000e+01   1.53814185e-01
1.24700000e+01   7.05550699e-02
5.79500000e+00   -2.13382841e-01
2.83000000e+00   -4.92337286e-01
1.40700000e+00   -3.79518818e-01
6.99500000e-01   -7.60495636e-02
3.08300000e-01   2.91889700e-04
1.38500000e-01   2.26994070e-03
6.14500000e-02   7.92093300e-04
S 21 1.0
4.46500000e+06   1.17400000e-07
6.68500000e+05   9.11700000e-07
1.52200000e+05   4.79790000e-06
4.33000000e+04   1.99725000e-05
1.44100000e+04   6.98666000e-05
5.39400000e+03   2.14013100e-04
2.21200000e+03   5.92574000e-04
9.68100000e+02   1.53611170e-03
4.41200000e+02   3.78886050e-03
2.07100000e+02   8.65221810e-03
9.98000000e+01   1.77881630e-02
4.92400000e+01   3.10894097e-02
2.47400000e+01   4.11817036e-02
1.24700000e+01   1.98252138e-02
5.79500000e+00   -6.61548777e-02
2.83000000e+00   -1.80055889e-01
1.40700000e+00   -2.58650133e-01
6.99500000e-01   -6.34512383e-02
3.08300000e-01   4.45814763e-01
1.38500000e-01   5.86713523e-01
6.14500000e-02   1.71335593e-01
S 21 1.0
4.46500000e+06   1.54400000e-07
6.68500000e+05   1.20970000e-06
1.52200000e+05   6.28680000e-06
4.33000000e+04   2.66189000e-05
1.44100000e+04   9.10384000e-05
5.39400000e+03   2.87271400e-04
2.21200000e+03   7.65629100e-04
9.68100000e+02   2.08105590e-03
4.41200000e+02   4.85934250e-03
2.07100000e+02   1.18522651e-02
9.98000000e+01   2.26106642e-02
4.92400000e+01   4.38278795e-02
2.47400000e+01   5.04887020e-02
1.24700000e+01   3.62050687e-02
5.79500000e+00   -1.27092864e-01
2.83000000e+00   -2.00125671e-01
1.40700000e+00   -5.74217932e-01
6.99500000e-01   3.05577965e-01
3.08300000e-01   1.79870833e+00
1.38500000e-01   -8.74698722e-01
6.14500000e-02   -7.51939794e-01
S 21 1.0
4.46500000e+06   2.05000000e-07
6.68500000e+05   1.64730000e-06
1.52200000e+05   8.24790000e-06
4.33000000e+04   3.67054000e-05
1.44100000e+04   1.17408700e-04
5.39400000e+03   4.04067900e-04
```

```
2.21200000e+03   9.61205500e-04
9.68100000e+02   3.00051730e-03
4.41200000e+02   5.95155910e-03
2.07100000e+02   1.75729444e-02
9.98000000e+01   2.67914016e-02
4.92400000e+01   6.91625792e-02
2.47400000e+01   5.04674095e-02
1.24700000e+01   8.44721410e-02
5.79500000e+00   -2.94216400e-01
2.83000000e+00   -1.27235735e-01
1.40700000e+00   -1.59825158e+00
6.99500000e-01   3.50880490e+00
3.08300000e-01   -1.09920288e+00
1.38500000e-01   -2.24128054e+00
6.14500000e-02   1.90711389e+00
P 14 1.0
3.57200000e+03   5.98101000e-05
8.46000000e+02   5.29019700e-04
2.74800000e+02   2.99272640e-03
1.05000000e+02   1.26203482e-02
4.43500000e+01   4.18585003e-02
2.00800000e+01   1.10129727e-01
9.53000000e+00   2.18574670e-01
4.63400000e+00   3.17480603e-01
2.28000000e+00   3.19330889e-01
1.11600000e+00   1.71414596e-01
4.99100000e-01   2.72061913e-02
2.25400000e-01   -3.24650000e-04
1.00100000e-01   8.25436900e-04
4.33200000e-02   -1.20198300e-04
P 14 1.0
3.57200000e+03   1.30489000e-05
8.46000000e+02   1.15334100e-04
2.74800000e+02   6.54562100e-04
1.05000000e+02   2.76796380e-03
4.43500000e+01   9.28172850e-03
2.00800000e+01   2.48181605e-02
9.53000000e+00   5.04241847e-02
4.63400000e+00   7.44168937e-02
2.28000000e+00   8.17489341e-02
1.11600000e+00   3.08043509e-02
4.99100000e-01   -1.78172493e-01
2.25400000e-01   -4.29742808e-01
1.00100000e-01   -4.11530218e-01
4.33200000e-02   -1.31675147e-01
P 14 1.0
3.57200000e+03   1.57401000e-05
8.46000000e+02   1.44740100e-04
2.74800000e+02   7.90095900e-04
1.05000000e+02   3.47530840e-03
4.43500000e+01   1.11422064e-02
2.00800000e+01   3.10943218e-02
9.53000000e+00   6.07776749e-02
4.63400000e+00   9.78451850e-02
2.28000000e+00   9.62138762e-02
1.11600000e+00   1.23953056e-01
4.99100000e-01   -6.35775221e-01
2.25400000e-01   -6.60206529e-01
1.00100000e-01   7.63210791e-01
4.33200000e-02   4.37019177e-01
P 14 1.0
3.57200000e+03   3.00647000e-05
8.46000000e+02   2.01963100e-04
2.74800000e+02   1.50816700e-03
1.05000000e+02   4.88658490e-03
4.43500000e+01   2.17118781e-02
2.00800000e+01   4.16318863e-02
9.53000000e+00   1.35924311e-01
4.63400000e+00   9.44419852e-02
2.28000000e+00   3.17748880e-01
1.11600000e+00   -2.72994296e-01
```

```
4.99100000e-01   -1.64660972e+00
2.25400000e-01   1.94010667e+00
1.00100000e-01   -9.99669875e-02
4.33200000e-02   -7.31844930e-01
D 5 1.0
3.23860000e+00   1.97751361e-02
1.37670000e+00   1.50954672e-02
5.85300000e-01   2.57236119e-01
2.48800000e-01   5.56047232e-01
1.05800000e-01   3.08114757e-01
D 5 1.0
3.23860000e+00   4.73247960e-03
1.37670000e+00   -9.63416463e-02
5.85300000e-01   8.70203551e-01
2.48800000e-01   1.00273984e-01
1.05800000e-01   -9.21418942e-01
F 4 1.0
1.35100000e+00   -1.31724073e-02
6.60000000e-01   2.47459738e-01
3.22500000e-01   5.29316438e-01
1.57500000e-01   3.56207230e-01
****
-P
S 21 1.0
5.38400000e+06   1.64570000e-06
8.06200000e+05   1.27815000e-05
1.83600000e+05   6.72065000e-05
5.22500000e+04   2.79710900e-04
1.73900000e+04   9.76699800e-04
6.52300000e+03   2.96834920e-03
2.68700000e+03   8.12399770e-03
1.17800000e+03   2.09200106e-02
5.36200000e+02   5.05590328e-02
2.51500000e+02   1.10479103e-01
1.21300000e+02   2.06956912e-01
5.98800000e+01   3.04737341e-01
3.00500000e+01   2.92952311e-01
1.51200000e+01   1.35560712e-01
7.01000000e+00   1.73207816e-02
3.44100000e+00   -3.51698000e-05
1.71200000e+00   8.03262000e-04
8.33700000e-01   -2.43839500e-04
3.91200000e-01   9.87220000e-05
1.77700000e-01   -5.12279000e-05
7.93900000e-02   1.00911000e-05
S 21 1.0
5.38400000e+06   4.46900000e-07
8.06200000e+05   3.46950000e-06
1.83600000e+05   1.82569000e-05
5.22500000e+04   7.59386000e-05
1.73900000e+04   2.65723600e-04
6.52300000e+03   8.08045300e-04
2.68700000e+03   2.22732830e-03
1.17800000e+03   5.78331990e-03
5.36200000e+02   1.43437812e-02
2.51500000e+02   3.27060584e-02
1.21300000e+02   6.73715266e-02
5.98800000e+01   1.17647137e-01
3.00500000e+01   1.57279812e-01
1.51200000e+01   8.38541752e-02
7.01000000e+00   -1.99717185e-01
3.44100000e+00   -4.98603034e-01
1.71200000e+00   -3.89817123e-01
8.33700000e-01   -7.43425810e-02
3.91200000e-01   -7.36775200e-04
1.77700000e-01   -9.26189400e-04
7.93900000e-02   0.00000000e+00
S 21 1.0
5.38400000e+06   1.23300000e-07
8.06200000e+05   9.57000000e-07
1.83600000e+05   5.03560000e-06
```

```
5.22500000e+04   2.09489000e-05
1.73900000e+04   7.32948000e-05
6.52300000e+03   2.22987100e-04
2.68700000e+03   6.14700400e-04
1.17800000e+03   1.59827480e-03
5.36200000e+02   3.96991650e-03
2.51500000e+02   9.09371040e-03
1.21300000e+02   1.88701405e-02
5.98800000e+01   3.35159167e-02
3.00500000e+01   4.61471119e-02
1.51200000e+01   2.56075836e-02
7.01000000e+00   -6.73395749e-02
3.44100000e+00   -2.03809441e-01
1.71200000e+00   -2.88303275e-01
8.33700000e-01   -4.07361705e-02
3.91200000e-01   4.71307689e-01
1.77700000e-01   5.65966007e-01
7.93900000e-02   1.70269360e-01
S 21 1.0
5.38400000e+06   1.52600000e-07
8.06200000e+05   1.19460000e-06
1.83600000e+05   6.21140000e-06
5.22500000e+04   2.62588000e-05
1.73900000e+04   8.99315000e-05
6.52300000e+03   2.81455000e-04
2.68700000e+03   7.48131000e-04
1.17800000e+03   2.03487350e-03
5.36200000e+02   4.80185600e-03
2.51500000e+02   1.17025222e-02
1.21300000e+02   2.26723683e-02
5.98800000e+01   4.43417480e-02
3.00500000e+01   5.42824195e-02
1.51200000e+01   4.13471455e-02
7.01000000e+00   -1.27458698e-01
3.44100000e+00   -2.16143117e-01
1.71200000e+00   -5.95219793e-01
8.33700000e-01   3.71262375e-01
3.91200000e-01   1.72720559e+00
1.77700000e-01   -8.07508990e-01
7.93900000e-02   -8.02768702e-01
S 21 1.0
5.38400000e+06   2.01700000e-07
8.06200000e+05   1.59010000e-06
1.83600000e+05   8.18220000e-06
5.22500000e+04   3.50779000e-05
1.73900000e+04   1.17913900e-04
6.52300000e+03   3.78250500e-04
2.68700000e+03   9.74014800e-04
1.17800000e+03   2.75608110e-03
5.36200000e+02   6.22510530e-03
2.51500000e+02   1.60328102e-02
1.21300000e+02   2.93865348e-02
5.98800000e+01   6.24183676e-02
3.00500000e+01   6.94862147e-02
1.51200000e+01   6.23982686e-02
7.01000000e+00   -2.22276309e-01
3.44100000e+00   -3.27768605e-01
1.71200000e+00   -1.26599615e+00
8.33700000e-01   3.35803976e+00
3.91200000e-01   -1.01939561e+00
1.77700000e-01   -2.46072372e+00
7.93900000e-02   1.99964963e+00
P 14 1.0
4.55200000e+03   5.19530000e-05
1.07800000e+03   4.60402400e-04
3.50100000e+02   2.62081640e-03
1.33800000e+02   1.11872441e-02
5.65200000e+01   3.78228974e-02
2.55800000e+01   1.02116397e-01
1.21400000e+01   2.10313745e-01
5.90200000e+00   3.17382380e-01
```

```
2.91000000e+00   3.27164036e-01
1.43500000e+00   1.77686982e-01
6.57000000e-01   2.93588218e-02
3.00500000e-01   -7.52283000e-05
1.34000000e-01   6.79589100e-04
5.78300000e-02   -1.25946100e-04
P 14 1.0
4.55200000e+03   1.25053000e-05
1.07800000e+03   1.10641500e-04
3.50100000e+02   6.32449500e-04
1.33800000e+02   2.70513400e-03
5.65200000e+01   9.25645860e-03
2.55800000e+01   2.54006335e-02
1.21400000e+01   5.37628786e-02
5.90200000e+00   8.25945281e-02
2.91000000e+00   9.26223095e-02
1.43500000e+00   3.09116256e-02
6.57000000e-01   -1.93095245e-01
3.00500000e-01   -4.36362552e-01
1.34000000e-01   -4.00880059e-01
5.78300000e-02   -1.22049293e-01
P 14 1.0
4.55200000e+03   1.55755000e-05
1.07800000e+03   1.42293500e-04
3.50100000e+02   7.88608800e-04
1.33800000e+02   3.48358910e-03
5.65200000e+01   1.15313768e-02
2.55800000e+01   3.27400156e-02
1.21400000e+01   6.76387623e-02
5.90200000e+00   1.10002175e-01
2.91000000e+00   1.11837996e-01
1.43500000e+00   1.08244664e-01
6.57000000e-01   -6.88491510e-01
3.00500000e-01   -5.89511985e-01
1.34000000e-01   7.74697719e-01
5.78300000e-02   4.12884245e-01
P 14 1.0
4.55200000e+03   2.99529000e-05
1.07800000e+03   1.99674200e-04
3.50100000e+02   1.51486450e-03
1.33800000e+02   4.95677510e-03
5.65200000e+01   2.26188204e-02
2.55800000e+01   4.52278279e-02
1.21400000e+01   1.49569116e-01
5.90200000e+00   1.16058996e-01
2.91000000e+00   3.29017516e-01
1.43500000e+00   -3.92408581e-01
6.57000000e-01   -1.53159372e+00
3.00500000e-01   1.96339207e+00
1.34000000e-01   -1.68662441e-01
5.78300000e-02   -7.13412188e-01
D 5 1.0
4.30080000e+00   2.12636418e-02
1.83460000e+00   1.88050678e-02
7.82600000e-01   2.64797630e-01
3.33900000e-01   5.53510347e-01
1.42400000e-01   3.00434000e-01
D 5 1.0
4.30080000e+00   -1.01265740e-03
1.83460000e+00   -1.03476132e-01
7.82600000e-01   8.91446808e-01
3.33900000e-01   6.94034624e-02
1.42400000e-01   -9.13145622e-01
F 4 1.0
1.81600000e+00   -1.26043063e-02
8.80600000e-01   2.45954440e-01
4.27000000e-01   5.49126285e-01
2.07000000e-01   3.36333481e-01
****
-S
S 21 1.0
```

```
6.29700000e+06   1.59210000e-06
9.43100000e+05   1.23599000e-05
2.14900000e+05   6.49159000e-05
6.12500000e+04   2.69332600e-04
2.04500000e+04   9.34603400e-04
7.71900000e+03   2.80814930e-03
3.19800000e+03   7.67362780e-03
1.40200000e+03   1.98887182e-02
6.37200000e+02   4.82561629e-02
2.98900000e+02   1.05750417e-01
1.44300000e+02   2.00209679e-01
7.12100000e+01   3.00704756e-01
3.57300000e+01   2.98654879e-01
1.79700000e+01   1.46326707e-01
8.34100000e+00   2.01597202e-02
4.11200000e+00   -1.02704800e-04
2.04500000e+00   1.10109070e-03
9.77000000e-01   -3.10142600e-04
4.76600000e-01   -1.74322800e-04
2.18500000e-01   -4.19145700e-04
9.75900000e-02   -9.12518000e-05
S 21 1.0
6.29700000e+06   4.38200000e-07
9.43100000e+05   3.40010000e-06
2.14900000e+05   1.78707000e-05
6.12500000e+04   7.41041000e-05
2.04500000e+04   2.57663900e-04
7.71900000e+03   7.74651600e-04
3.19800000e+03   2.13129540e-03
1.40200000e+03   5.56892110e-03
6.37200000e+02   1.38537158e-02
2.98900000e+02   3.16453111e-02
1.44300000e+02   6.56714009e-02
7.12100000e+01   1.16366754e-01
3.57300000e+01   1.59128876e-01
1.79700000e+01   9.41285220e-02
8.34100000e+00   -1.86945575e-01
4.11200000e+00   -5.01734920e-01
2.04500000e+00   -3.95479956e-01
9.77000000e-01   -7.32558993e-02
4.76600000e-01   -6.16003380e-03
2.18500000e-01   -8.64194040e-03
9.75900000e-02   -2.10110990e-03
S 21 1.0
6.29700000e+06   1.35000000e-07
9.43100000e+05   1.04730000e-06
2.14900000e+05   5.50540000e-06
6.12500000e+04   2.28258000e-05
2.04500000e+04   7.93897000e-05
7.71900000e+03   2.38649600e-04
3.19800000e+03   6.57099800e-04
1.40200000e+03   1.71758370e-03
6.37200000e+02   4.28269700e-03
2.98900000e+02   9.81182580e-03
1.44300000e+02   2.05253893e-02
7.12100000e+01   3.68719010e-02
3.57300000e+01   5.19727886e-02
1.79700000e+01   3.18049495e-02
8.34100000e+00   -6.92984997e-02
4.11200000e+00   -2.29930204e-01
2.04500000e+00   -3.16948205e-01
9.77000000e-01   -1.59749150e-02
4.76600000e-01   4.95799807e-01
2.18500000e-01   5.50219898e-01
9.75900000e-02   1.58102388e-01
S 21 1.0
6.29700000e+06   1.51500000e-07
9.43100000e+05   1.18640000e-06
2.14900000e+05   6.15540000e-06
6.12500000e+04   2.59766000e-05
2.04500000e+04   8.82303000e-05
```

```
7.71900000e+03   2.73765800e-04
3.19800000e+03   7.23791000e-04
1.40200000e+03   1.98836830e-03
6.37200000e+02   4.68848970e-03
2.98900000e+02   1.14963962e-02
1.44300000e+02   2.23354902e-02
7.12100000e+01   4.45622770e-02
3.57300000e+01   5.56857997e-02
1.79700000e+01   4.65569275e-02
8.34100000e+00   -1.26255521e-01
4.11200000e+00   -2.17545259e-01
2.04500000e+00   -6.28301634e-01
9.77000000e-01   4.24188658e-01
4.76600000e-01   1.74505122e+00
2.18500000e-01   -8.97041429e-01
9.75900000e-02   -7.68350182e-01
S 21 1.0
6.29700000e+06   2.17500000e-07
9.43100000e+05   1.70140000e-06
2.14900000e+05   8.83650000e-06
6.12500000e+04   3.72408000e-05
2.04500000e+04   1.26734500e-04
7.71900000e+03   3.92329400e-04
3.19800000e+03   1.04101410e-03
1.40200000e+03   2.85121380e-03
6.37200000e+02   6.76792040e-03
2.98900000e+02   1.65725289e-02
1.44300000e+02   3.26736745e-02
7.12100000e+01   6.51357654e-02
3.57300000e+01   8.50063621e-02
1.79700000e+01   6.24786338e-02
8.34100000e+00   -2.34497907e-01
4.11200000e+00   -4.09053361e-01
2.04500000e+00   -1.25281008e+00
9.77000000e-01   3.93868724e+00
4.76600000e-01   -1.96343163e+00
2.18500000e-01   -1.83559477e+00
9.75900000e-02   1.78276513e+00
P 14 1.0
5.26600000e+03   5.22613000e-05
1.24700000e+03   4.63522300e-04
4.05000000e+02   2.64098570e-03
1.54800000e+02   1.13168621e-02
6.53800000e+01   3.84702429e-02
2.95900000e+01   1.04338980e-01
1.40400000e+01   2.15682941e-01
6.82400000e+00   3.25258367e-01
3.36900000e+00   3.26179695e-01
1.66600000e+00   1.63147292e-01
7.68100000e-01   2.38968197e-02
3.50400000e-01   4.74148300e-04
1.55600000e-01   7.36633300e-04
6.68100000e-02   1.98570000e-05
P 14 1.0
5.26600000e+03   1.34501000e-05
1.24700000e+03   1.18880900e-04
4.05000000e+02   6.81566000e-04
1.54800000e+02   2.92224110e-03
6.53800000e+01   1.00822376e-02
2.95900000e+01   2.77819341e-02
1.40400000e+01   5.93004576e-02
6.82400000e+00   9.11250782e-02
3.36900000e+00   1.00494729e-01
1.66600000e+00   1.22559698e-02
7.68100000e-01   -2.37663445e-01
3.50400000e-01   -4.37452406e-01
1.55600000e-01   -3.65271350e-01
6.68100000e-02   -1.13754627e-01
P 14 1.0
5.26600000e+03   1.54652000e-05
1.24700000e+03   1.40879100e-04
```

```
4.05000000e+02   7.84589200e-04
1.54800000e+02   3.46750170e-03
6.53800000e+01   1.16099085e-02
2.95900000e+01   3.31206035e-02
1.40400000e+01   6.93083831e-02
6.82400000e+00   1.10584668e-01
3.36900000e+00   1.08493300e-01
1.66600000e+00   7.73246928e-02
7.68100000e-01   -7.01546916e-01
3.50400000e-01   -4.72302903e-01
1.55600000e-01   6.91338286e-01
6.68100000e-02   4.65832381e-01
P 14 1.0
5.26600000e+03   2.61265000e-05
1.24700000e+03   1.83114200e-04
4.05000000e+02   1.32375180e-03
1.54800000e+02   4.55960340e-03
6.53800000e+01   1.99287095e-02
2.95900000e+01   4.28388835e-02
1.40400000e+01   1.31020473e-01
6.82400000e+00   1.14102100e-01
3.36900000e+00   2.82487282e-01
1.66600000e+00   -3.61975733e-01
7.68100000e-01   -1.29224861e+00
3.50400000e-01   1.43716297e+00
1.55600000e-01   4.00730908e-01
6.68100000e-02   -1.00149722e+00
D 5 1.0
5.07550000e+00   2.51450138e-02
2.18330000e+00   2.47353053e-02
9.39200000e-01   2.92276387e-01
4.04000000e-01   5.46121626e-01
1.73800000e-01   2.74586046e-01
D 5 1.0
5.07550000e+00   -3.42198330e-03
2.18330000e+00   -6.85076016e-02
9.39200000e-01   9.01076877e-01
4.04000000e-01   -1.18517043e-02
1.73800000e-01   -8.84972741e-01
F 4 1.0
1.32220000e+00   7.48384273e-02
7.31900000e-01   4.16550600e-01
4.05100000e-01   3.45168543e-01
2.24300000e-01   3.00279115e-01
****
-Cl
S 20 1.0
6.41000000e+06   1.81340000e-06
9.59600000e+05   1.41111000e-05
2.18300000e+05   7.42369000e-05
6.18100000e+04   3.14115200e-04
2.01400000e+04   1.14636230e-03
7.26400000e+03   3.73869090e-03
2.83200000e+03   1.10940777e-02
1.17500000e+03   3.01135997e-02
5.12600000e+02   7.39105226e-02
2.33000000e+02   1.58249381e-01
1.09500000e+02   2.74735095e-01
5.28600000e+01   3.34036462e-01
2.58400000e+01   2.17559780e-01
1.21700000e+01   4.57431155e-02
6.03000000e+00   -2.93391000e-05
3.01200000e+00   1.83502750e-03
1.51100000e+00   -4.44532700e-04
6.60400000e-01   -7.76684000e-05
2.92600000e-01   -4.58712400e-04
1.25400000e-01   -9.40595000e-05
S 20 1.0
6.41000000e+06   5.31400000e-07
9.59600000e+05   4.13560000e-06
2.18300000e+05   2.17541000e-05
```

```
6.18100000e+04   9.21099000e-05
2.01400000e+04   3.36357500e-04
7.26400000e+03   1.10046940e-03
2.83200000e+03   3.28485880e-03
1.17500000e+03   9.05738090e-03
5.12600000e+02   2.29428466e-02
2.33000000e+02   5.25801453e-02
1.09500000e+02   1.04288359e-01
5.28600000e+01   1.65462254e-01
2.58400000e+01   1.54021839e-01
1.21700000e+01   -7.38403143e-02
6.03000000e+00   -4.64015406e-01
3.01200000e+00   -5.67479308e-01
1.51100000e+00   -1.90508562e-01
6.60400000e-01   1.23140436e-01
2.92600000e-01   1.81415144e-01
1.25400000e-01   5.72888245e-02
S 20 1.0
6.41000000e+06   3.00000000e-09
9.59600000e+05   2.42000000e-08
2.18300000e+05   1.22700000e-07
6.18100000e+04   5.44400000e-07
2.01400000e+04   1.86150000e-06
7.26400000e+03   6.49150000e-06
2.83200000e+03   1.71666000e-05
1.17500000e+03   4.96124000e-05
5.12600000e+02   9.06845000e-05
2.33000000e+02   1.67295600e-04
1.09500000e+02   -1.52020100e-04
5.28600000e+01   -1.18701400e-03
2.58400000e+01   -3.57029940e-03
1.21700000e+01   4.45877520e-03
6.03000000e+00   4.23049009e-02
3.01200000e+00   1.72928718e-01
1.51100000e+00   7.99121551e-02
6.60400000e-01   -4.30562797e-01
2.92600000e-01   -5.80076876e-01
1.25400000e-01   -1.83332217e-01
S 20 1.0
6.41000000e+06   1.69800000e-07
9.59600000e+05   1.30200000e-06
2.18300000e+05   6.99600000e-06
6.18100000e+04   2.88036000e-05
2.01400000e+04   1.08950000e-04
7.26400000e+03   3.41548300e-04
2.83200000e+03   1.07404200e-03
1.17500000e+03   2.78925910e-03
5.12600000e+02   7.62512340e-03
2.33000000e+02   1.61014859e-02
1.09500000e+02   3.62533789e-02
5.28600000e+01   5.04717507e-02
2.58400000e+01   6.67371854e-02
1.21700000e+01   -6.17185781e-02
6.03000000e+00   -1.24932458e-01
3.01200000e+00   -6.04647456e-01
1.51100000e+00   -1.78307776e-01
6.60400000e-01   2.18904089e+00
2.92600000e-01   -8.54423151e-01
1.25400000e-01   -8.25303523e-01
S 20 1.0
6.41000000e+06   -2.45400000e-07
9.59600000e+05   -1.70380000e-06
2.18300000e+05   -1.05333000e-05
6.18100000e+04   -3.58464000e-05
2.01400000e+04   -1.71046400e-04
7.26400000e+03   -3.99562600e-04
2.83200000e+03   -1.76898390e-03
1.17500000e+03   -2.99905090e-03
5.12600000e+02   -1.33610420e-02
2.33000000e+02   -1.49230657e-02
1.09500000e+02   -7.14207934e-02
```

```
5.28600000e+01   -2.38172309e-02
2.58400000e+01   -2.06192769e-01
1.21700000e+01   3.57897053e-01
6.03000000e+00   -3.57728043e-01
3.01200000e+00   2.69247013e+00
1.51100000e+00   -3.95409694e+00
6.60400000e-01   6.26233179e-01
2.92600000e-01   2.58435997e+00
1.25400000e-01   -1.90447989e+00
P 12 1.0
2.54800000e+03   2.35701700e-04
6.03700000e+02   2.05157990e-03
1.95600000e+02   1.11543331e-02
7.41500000e+01   4.39815972e-02
3.09400000e+01   1.29994232e-01
1.36900000e+01   2.72959183e-01
6.22900000e+00   3.83690452e-01
2.87800000e+00   2.91869453e-01
1.28200000e+00   7.04461278e-02
5.64100000e-01   1.28677700e-03
2.34800000e-01   1.82967110e-03
9.31200000e-02   1.63527000e-05
P 12 1.0
2.54800000e+03   6.39499000e-05
6.03700000e+02   5.56986200e-04
1.95600000e+02   3.04748690e-03
7.41500000e+01   1.21470785e-02
3.09400000e+01   3.68757425e-02
1.36900000e+01   7.96346992e-02
6.22900000e+00   1.17986470e-01
2.87800000e+00   8.71432235e-02
1.28200000e+00   -1.42101282e-01
5.64100000e-01   -4.27685723e-01
2.34800000e-01   -4.43417262e-01
9.31200000e-02   -1.62396124e-01
P 12 1.0
2.54800000e+03   7.92628000e-05
6.03700000e+02   7.42637900e-04
1.95600000e+02   3.78255490e-03
7.41500000e+01   1.63312278e-02
3.09400000e+01   4.60944074e-02
1.36900000e+01   1.11998718e-01
6.22900000e+00   1.29275347e-01
2.87800000e+00   1.72551149e-01
1.28200000e+00   -4.62888419e-01
5.64100000e-01   -7.98744967e-01
2.34800000e-01   6.41053120e-01
9.31200000e-02   5.32811061e-01
P 12 1.0
2.54800000e+03   1.07726600e-04
6.03700000e+02   1.02722500e-03
1.95600000e+02   5.16452550e-03
7.41500000e+01   2.28840699e-02
3.09400000e+01   6.51495253e-02
1.36900000e+01   1.62079215e-01
6.22900000e+00   1.60873768e-01
2.87800000e+00   2.85444809e-01
1.28200000e+00   -1.72569936e+00
5.64100000e-01   1.13370702e+00
2.34800000e-01   7.41597631e-01
9.31200000e-02   -1.04751067e+00
D 4 1.0
2.50000000e-01   3.72890783e-01
6.18000000e-01   5.73676911e-01
1.52900000e+00   1.71976094e-01
3.78100000e+00   3.71180574e-02
D 4 1.0
2.50000000e-01   -1.03878704e+00
6.18000000e-01   4.42872368e-01
1.52900000e+00   6.54921272e-01
3.78100000e+00   -8.95377154e-02
```

```
F 3 1.0
3.20000000e-01   3.30473066e-01
6.56000000e-01   5.38809829e-01
1.34500000e+00   2.56779382e-01
****
-Ar
S 21 1.0
9.14900000e+06   1.34270000e-06
1.37000000e+06   1.04386000e-05
3.11900000e+05   5.48567000e-05
8.86500000e+04   2.29582800e-04
2.93300000e+04   8.10333800e-04
1.09300000e+04   2.48534130e-03
4.48000000e+03   6.83691730e-03
1.96200000e+03   1.76198815e-02
8.94100000e+02   4.28751718e-02
4.19600000e+02   9.54852872e-02
2.02300000e+02   1.85064004e-01
9.98400000e+01   2.89041537e-01
5.00700000e+01   3.10165596e-01
2.51400000e+01   1.72183219e-01
1.18100000e+01   2.85227179e-02
5.88200000e+00   -5.75738100e-04
2.93900000e+00   1.18121500e-03
1.40500000e+00   -4.80534900e-04
6.96300000e-01   1.88849400e-04
3.18800000e-01   -9.72509000e-05
1.41000000e-01   1.74327000e-05
S 21 1.0
9.14900000e+06   3.81300000e-07
1.37000000e+06   2.96330000e-06
3.11900000e+05   1.55803000e-05
8.86500000e+04   6.51880000e-05
2.93300000e+04   2.30432200e-04
1.09300000e+04   7.07458600e-04
4.48000000e+03   1.95729630e-03
1.96200000e+03   5.08562250e-03
8.94100000e+02   1.26528350e-02
4.19600000e+02   2.93064513e-02
2.02300000e+02   6.17712186e-02
9.98400000e+01   1.12540721e-01
5.00700000e+01   1.62293160e-01
2.51400000e+01   1.18411976e-01
1.18100000e+01   -1.46147512e-01
5.88200000e+00   -4.97751505e-01
2.93900000e+00   -4.34239643e-01
1.40500000e+00   -8.90745104e-02
6.96300000e-01   -5.38050000e-04
3.18800000e-01   -2.04036030e-03
1.41000000e-01   2.80176000e-05
S 21 1.0
9.14900000e+06   1.19600000e-07
1.37000000e+06   9.29500000e-07
3.11900000e+05   4.88840000e-06
8.86500000e+04   2.04455000e-05
2.93300000e+04   7.23150000e-05
1.09300000e+04   2.21909700e-04
4.48000000e+03   6.14754500e-04
1.96200000e+03   1.59709840e-03
8.94100000e+02   3.98653340e-03
4.19600000e+02   9.25777920e-03
2.02300000e+02   1.97068769e-02
9.98400000e+01   3.64106866e-02
5.00700000e+01   5.43600462e-02
2.51400000e+01   4.12101400e-02
1.18100000e+01   -5.57198596e-02
5.88200000e+00   -2.38979099e-01
2.93900000e+00   -3.48066057e-01
1.40500000e+00   -1.14046784e-02
6.96300000e-01   5.08891467e-01
3.18800000e-01   5.48407888e-01
```

```
1.41000000e-01   1.56444137e-01
S 21 1.0
9.14900000e+06   -1.36700000e-07
1.37000000e+06   -1.07400000e-06
3.11900000e+05   -5.55650000e-06
8.86500000e+04   -2.37565000e-05
2.93300000e+04   -8.16261000e-05
1.09300000e+04   -2.60209600e-04
4.48000000e+03   -6.86131100e-04
1.96200000e+03   -1.89503990e-03
8.94100000e+02   -4.40774940e-03
4.19600000e+02   -1.11231815e-02
2.02300000e+02   -2.15812668e-02
9.98400000e+01   -4.50890638e-02
5.00700000e+01   -5.82069373e-02
2.51400000e+01   -6.07658119e-02
1.18100000e+01   1.16719219e-01
5.88200000e+00   2.03465012e-01
2.93900000e+00   7.21530023e-01
1.40500000e+00   -4.38597272e-01
6.96300000e-01   -1.84490573e+00
3.18800000e-01   1.02026461e+00
1.41000000e-01   7.09466695e-01
S 21 1.0
9.14900000e+06   2.02700000e-07
1.37000000e+06   1.59170000e-06
3.11900000e+05   8.24540000e-06
8.86500000e+04   3.51960000e-05
2.93300000e+04   1.21200400e-04
1.09300000e+04   3.85346100e-04
4.48000000e+03   1.02026250e-03
1.96200000e+03   2.80766860e-03
8.94100000e+02   6.57909810e-03
4.19600000e+02   1.65651285e-02
2.02300000e+02   3.26566656e-02
9.98400000e+01   6.82935031e-02
5.00700000e+01   9.31850677e-02
2.51400000e+01   8.92905674e-02
1.18100000e+01   -2.51036339e-01
5.88200000e+00   -3.89771465e-01
2.93900000e+00   -1.51218152e+00
1.40500000e+00   4.58982859e+00
6.96300000e-01   -2.72890640e+00
3.18800000e-01   -1.32480766e+00
1.41000000e-01   1.59409222e+00
P 14 1.0
7.05000000e+03   5.01843000e-05
1.66900000e+03   4.45365000e-04
5.42100000e+02   2.54799730e-03
2.07100000e+02   1.10155237e-02
8.75200000e+01   3.78489764e-02
3.96100000e+01   1.04355193e-01
1.87800000e+01   2.19335151e-01
9.13000000e+00   3.34615307e-01
4.51600000e+00   3.26771020e-01
2.24500000e+00   1.48153015e-01
1.06500000e+00   1.92586150e-02
4.88500000e-01   5.51936800e-04
2.16600000e-01   1.76924600e-04
9.25500000e-02   -1.68555000e-05
P 14 1.0
7.05000000e+03   1.41163000e-05
1.66900000e+03   1.24932700e-04
5.42100000e+02   7.18915000e-04
2.07100000e+02   3.11348210e-03
8.75200000e+01   1.08614712e-02
3.96100000e+01   3.05213102e-02
1.87800000e+01   6.64087472e-02
9.13000000e+00   1.04335520e-01
4.51600000e+00   1.10481227e-01
2.24500000e+00   -1.32896226e-02
```

```
1.06500000e+00   -2.78221541e-01
4.88500000e-01   -4.34865574e-01
2.16600000e-01   -3.37575208e-01
9.25500000e-02   -9.52338864e-02
P 14 1.0
7.05000000e+03   1.81269000e-05
1.66900000e+03   1.60467400e-04
5.42100000e+02   9.24138300e-04
2.07100000e+02   4.00947730e-03
8.75200000e+01   1.40247320e-02
3.96100000e+01   3.96644574e-02
1.87800000e+01   8.83804352e-02
9.13000000e+00   1.32531330e-01
4.51600000e+00   1.41996113e-01
2.24500000e+00   -1.51877420e-03
1.06500000e+00   -8.79227120e-01
4.88500000e-01   -2.13830610e-01
2.16600000e-01   7.84225468e-01
9.25500000e-02   2.86621342e-01
P 14 1.0
7.05000000e+03   3.43626000e-05
1.66900000e+03   2.03057600e-04
5.42100000e+02   1.74641850e-03
2.07100000e+02   5.17887170e-03
8.75200000e+01   2.70262790e-02
3.96100000e+01   5.07693530e-02
1.87800000e+01   1.93375640e-01
9.13000000e+00   1.01572651e-01
4.51600000e+00   4.35592857e-01
2.24500000e+00   -9.85508099e-01
1.06500000e+00   -8.43210270e-01
4.88500000e-01   2.02126894e+00
2.16600000e-01   -6.68903866e-01
9.25500000e-02   -4.77495424e-01
D 5 1.0
7.63270000e+00   2.79057812e-02
3.28760000e+00   2.93683713e-02
1.41600000e+00   3.03094373e-01
6.09900000e-01   5.42279927e-01
2.62700000e-01   2.64001541e-01
D 5 1.0
7.63270000e+00   -3.28925500e-03
3.28760000e+00   -7.01912500e-02
1.41600000e+00   9.23579371e-01
6.09900000e-01   -6.16176285e-02
2.62700000e-01   -8.64872079e-01
F 4 1.0
3.05820000e+00   -1.06008451e-02
1.52920000e+00   3.04583459e-01
7.64700000e-01   5.42344318e-01
3.82400000e-01   2.77175008e-01
****
```

S7.3 ORCA Format

```
#basis set ano-pVTZ
#
# ANO basis sets from atomic MR-ACPF based on cc-pV6Z primitives
#
# H-Al: Neese, F.; Valeev, E.F J. Chem. Theo. Comp., 2010, submitted for publication
# ORIGINAL FILE WAS BOGUS COPY OF aug-pVDZ: corrected Jan 27, 2012 by Gershom Martin
# how: copied ano-pVQZ, stripped off top angular momentum plus last ANO of each remaining angular
momentum
#
# converted 31/12/2025 by MAI using home-written PERL script
NewGTO H
S 8
  1 4.02009900e+02   -4.42950800e-04
  2 6.02419600e+01   -3.25074670e-03
  3 1.37321700e+01   -1.81152250e-02
  4 3.90450500e+00   -6.36329998e-02
```

```
  5 1.28270900e+00   -2.22163641e-01
  6 4.65544000e-01   -4.04104783e-01
  7 1.81120000e-01   -3.48108109e-01
  8 7.27910000e-02   -9.27213251e-02
S 8
  1 4.02009900e+02   -5.85791700e-04
  2 6.02419600e+01   -2.09634430e-03
  3 1.37321700e+01   -3.04299021e-02
  4 3.90450500e+00   -2.88272025e-02
  5 1.28270900e+00   -7.52694563e-01
  6 4.65544000e-01   -5.30306000e-01
  7 1.81120000e-01   9.52467620e-01
  8 7.27910000e-02   1.94410430e-01
S 8
  1 4.02009900e+02   -6.08315000e-04
  2 6.02419600e+01   -5.24835000e-03
  3 1.37321700e+01   -2.54171128e-02
  4 3.90450500e+00   -2.37640640e-01
  5 1.28270900e+00   -1.70965835e+00
  6 4.65544000e-01   2.67896543e+00
  7 1.81120000e-01   -1.03338631e+00
  8 7.27910000e-02   -2.48623702e-01
P 6
  1 9.88000000e+00   5.53122400e-03
  2 3.95000000e+00   5.32324280e-03
  3 1.58000000e+00   1.50384747e-01
  4 6.30000000e-01   5.82944394e-01
  5 2.50000000e-01   3.39947055e-01
  6 1.00000000e-01   3.17494051e-02
P 6
  1 9.88000000e+00   -2.80600947e-02
  2 3.95000000e+00   3.43704220e-02
  3 1.58000000e+00   -8.94015866e-01
  4 6.30000000e-01   -2.47268769e-01
  5 2.50000000e-01   8.48782689e-01
  6 1.00000000e-01   2.28203559e-02
D 4
  1 4.00000000e+00   2.20110300e-03
  2 1.60000000e+00   -2.78252481e-01
  3 6.40000000e-01   -6.65042830e-01
  4 2.60000000e-01   -1.74847619e-01
end
NewGTO He
S 10
  1 4.78500000e+03   6.03202000e-05
  2 7.17000000e+02   4.68496000e-04
  3 1.63200000e+02   2.45014670e-03
  4 4.62600000e+01   1.01553835e-02
  5 1.51000000e+01   3.50340333e-02
  6 5.43700000e+00   9.92797341e-02
  7 2.08800000e+00   2.17387130e-01
  8 8.29700000e-01   3.47268217e-01
  9 3.36600000e-01   3.44557817e-01
 10 1.36900000e-01   1.18026005e-01
S 10
  1 4.78500000e+03   8.88731000e-05
  2 7.17000000e+02   6.94021400e-04
  3 1.63200000e+02   3.64339710e-03
  4 4.62600000e+01   1.57462582e-02
  5 1.51000000e+01   6.14009646e-02
  6 5.43700000e+00   2.41475510e-01
  7 2.08800000e+00   6.11964132e-01
  8 8.29700000e-01   1.96132944e-01
  9 3.36600000e-01   -7.24164219e-01
 10 1.36900000e-01   -3.38281481e-01
S 10
  1 4.78500000e+03   1.28705400e-04
  2 7.17000000e+02   1.14776780e-03
  3 1.63200000e+02   5.09838010e-03
  4 4.62600000e+01   3.06322873e-02
  5 1.51000000e+01   1.39615961e-01
```

```
  6 5.43700000e+00   8.57718403e-01
  7 2.08800000e+00   9.48064849e-02
  8 8.29700000e-01   -1.72505080e+00
  9 3.36600000e-01   6.54259777e-01
 10 1.36900000e-01   5.29420091e-01
P 5
  1 3.87000000e-01   2.88744289e-01
  2 9.84000000e-01   5.36134245e-01
  3 2.49800000e+00   2.65902008e-01
  4 6.34200000e+00   4.00702083e-02
  5 1.61040000e+01   9.03858320e-03
P 5
  1 3.87000000e-01   -6.54417328e-01
  2 9.84000000e-01   -2.75230872e-01
  3 2.49800000e+00   7.99308321e-01
  4 6.34200000e+00   2.67165313e-01
  5 1.61040000e+01   3.10059145e-02
D 4
  1 7.47000000e-01   4.65842919e-01
  2 1.91000000e+00   5.37343596e-01
  3 4.88600000e+00   1.30942192e-01
  4 1.24980000e+01   7.13509580e-03
end
NewGTO Li
S 19
  1 7.06810000e+04   5.32360000e-06
  2 1.35940000e+04   3.23747000e-05
  3 3.10040000e+03   1.87960300e-04
  4 8.26460000e+02   8.71221900e-04
  5 2.53760000e+02   3.38367000e-03
  6 8.84510000e+01   1.10494789e-02
  7 3.44930000e+01   3.05955979e-02
  8 1.48310000e+01   7.07341149e-02
  9 6.92990000e+00   1.34555716e-01
 10 3.46780000e+00   2.06961592e-01
 11 1.83160000e+00   2.59108572e-01
 12 1.00630000e+00   2.46866157e-01
 13 5.66780000e-01   1.50116229e-01
 14 3.22540000e-01   4.48272557e-02
 15 1.82770000e-01   3.57552870e-03
 16 1.01640000e-01   3.15460900e-04
 17 5.46660000e-02   5.36622000e-04
 18 2.80250000e-02   4.57424400e-04
 19 1.34970000e-02   4.71593000e-05
S 19
  1 7.06810000e+04   8.57700000e-07
  2 1.35940000e+04   5.21600000e-06
  3 3.10040000e+03   3.02866000e-05
  4 8.26460000e+02   1.40458800e-04
  5 2.53760000e+02   5.46352500e-04
  6 8.84510000e+01   1.79186610e-03
  7 3.44930000e+01   5.01200740e-03
  8 1.48310000e+01   1.18375809e-02
  9 6.92990000e+00   2.33643106e-02
 10 3.46780000e+00   3.81867406e-02
 11 1.83160000e+00   5.36789047e-02
 12 1.00630000e+00   6.83347487e-02
 13 5.66780000e-01   7.49471861e-02
 14 3.22540000e-01   5.92482250e-02
 15 1.82770000e-01   -2.60749945e-02
 16 1.01640000e-01   -2.22043244e-01
 17 5.46660000e-02   -4.73451586e-01
 18 2.80250000e-02   -3.62075447e-01
 19 1.34970000e-02   -4.07343934e-02
S 19
  1 7.06810000e+04   8.04230000e-06
  2 1.35940000e+04   4.90032000e-05
  3 3.10040000e+03   2.84240000e-04
  4 8.26460000e+02   1.32650290e-03
  5 2.53760000e+02   5.20972940e-03
  6 8.84510000e+01   1.79882238e-02
```

```
  7 3.44930000e+01   5.71974540e-02
  8 1.48310000e+01   1.78258163e-01
  9 6.92990000e+00   4.29897013e-01
 10 3.46780000e+00   4.50865827e-01
 11 1.83160000e+00   -3.71352880e-02
 12 1.00630000e+00   -4.87212099e-01
 13 5.66780000e-01   -4.62766855e-01
 14 3.22540000e-01   -1.60128984e-01
 15 1.82770000e-01   -1.16236967e-02
 16 1.01640000e-01   6.61103743e-02
 17 5.46660000e-02   1.35132196e-01
 18 2.80250000e-02   1.04706424e-01
 19 1.34970000e-02   1.16751457e-02
S 19
  1 7.06810000e+04   1.13754000e-05
  2 1.35940000e+04   7.11224000e-05
  3 3.10040000e+03   4.00728700e-04
  4 8.26460000e+02   1.95738370e-03
  5 2.53760000e+02   7.61399060e-03
  6 8.84510000e+01   3.17360413e-02
  7 3.44930000e+01   1.34215264e-01
  8 1.48310000e+01   5.84227772e-01
  9 6.92990000e+00   6.80609017e-01
 10 3.46780000e+00   -1.00509861e+00
 11 1.83160000e+00   -9.44670188e-01
 12 1.00630000e+00   9.56130540e-02
 13 5.66780000e-01   6.97462192e-01
 14 3.22540000e-01   3.95465840e-01
 15 1.82770000e-01   7.14809958e-02
 16 1.01640000e-01   -8.18978750e-02
 17 5.46660000e-02   -1.59881592e-01
 18 2.80250000e-02   -1.28275016e-01
 19 1.34970000e-02   -1.41083238e-02
P 8
  1 2.85000000e+01   1.89870192e-02
  2 6.64000000e+00   3.14107276e-01
  3 1.92000000e+00   6.61727662e-01
  4 7.70000000e-01   8.86066288e-02
  5 3.20000000e-01   1.15657049e-01
  6 1.50000000e-01   -6.47171322e-02
  7 6.60000000e-02   2.69408101e-02
  8 2.50000000e-02   -7.47757140e-03
P 8
  1 2.85000000e+01   2.74718905e-02
  2 6.64000000e+00   3.09097805e-01
  3 1.92000000e+00   3.22503617e-01
  4 7.70000000e-01   -3.78769172e-01
  5 3.20000000e-01   -4.32978084e-01
  6 1.50000000e-01   -2.89609514e-01
  7 6.60000000e-02   -1.05459800e-01
  8 2.50000000e-02   -9.18879160e-03
D 3
  1 1.75000000e+00   1.00349740e+00
  2 3.00000000e-01   -1.74235563e-02
  3 1.10000000e-01   2.14475965e-02
end
NewGTO Be
S 19
  1 1.39330000e+05   4.81370000e-06
  2 2.67740000e+04   2.92950000e-05
  3 6.11200000e+03   1.69653400e-04
  4 1.63290000e+03   7.83798800e-04
  5 5.03030000e+02   3.02966040e-03
  6 1.76030000e+02   9.86889620e-03
  7 6.89390000e+01   2.73158639e-02
  8 2.97680000e+01   6.38839975e-02
  9 1.39630000e+01   1.24475533e-01
 10 7.00900000e+00   1.99794514e-01
 11 3.70940000e+00   2.60302499e-01
 12 2.03920000e+00   2.56116682e-01
 13 1.14720000e+00   1.60223839e-01
```

```
 14 6.50700000e-01   4.79172446e-02
 15 3.66590000e-01   4.05193800e-03
 16 2.02110000e-01   5.53253000e-05
 17 1.07420000e-01   -1.46329000e-05
 18 5.42310000e-02   5.42280000e-06
 19 2.56190000e-02   0.00000000e+00
S 19
  1 1.39330000e+05   8.96600000e-07
  2 2.67740000e+04   5.45660000e-06
  3 6.11200000e+03   3.16042000e-05
  4 1.63290000e+03   1.46094500e-04
  5 5.03030000e+02   5.65594800e-04
  6 1.76030000e+02   1.85049760e-03
  7 6.89390000e+01   5.17382070e-03
  8 2.97680000e+01   1.23588076e-02
  9 1.39630000e+01   2.50209252e-02
 10 7.00900000e+00   4.29921324e-02
 11 3.70940000e+00   6.40053839e-02
 12 2.03920000e+00   8.37309282e-02
 13 1.14720000e+00   8.86004427e-02
 14 6.50700000e-01   4.53339368e-02
 15 3.66590000e-01   -7.74503499e-02
 16 2.02110000e-01   -2.76957508e-01
 17 1.07420000e-01   -4.53239610e-01
 18 5.42310000e-02   -3.01158473e-01
 19 2.56190000e-02   -3.31442139e-02
S 19
  1 1.39330000e+05   -1.33880000e-06
  2 2.67740000e+04   -8.14490000e-06
  3 6.11200000e+03   -4.72010000e-05
  4 1.63290000e+03   -2.18114400e-04
  5 5.03030000e+02   -8.45637900e-04
  6 1.76030000e+02   -2.77009980e-03
  7 6.89390000e+01   -7.80186690e-03
  8 2.97680000e+01   -1.88623345e-02
  9 1.39630000e+01   -3.93045895e-02
 10 7.00900000e+00   -6.86639537e-02
 11 3.70940000e+00   -1.01644502e-01
 12 2.03920000e+00   -1.29787737e-01
 13 1.14720000e+00   -1.78094164e-01
 14 6.50700000e-01   -4.75153109e-02
 15 3.66590000e-01   7.22048537e-01
 16 2.02110000e-01   9.93782109e-01
 17 1.07420000e-01   -3.70738876e-01
 18 5.42310000e-02   -9.02262037e-01
 19 2.56190000e-02   -1.16365225e-01
S 19
  1 1.39330000e+05   -2.34770000e-06
  2 2.67740000e+04   -1.41798000e-05
  3 6.11200000e+03   -8.29446000e-05
  4 1.63290000e+03   -3.79581800e-04
  5 5.03030000e+02   -1.49527260e-03
  6 1.76030000e+02   -4.86054750e-03
  7 6.89390000e+01   -1.44204026e-02
  8 2.97680000e+01   -3.59876745e-02
  9 1.39630000e+01   -8.71441173e-02
 10 7.00900000e+00   -1.38293538e-01
 11 3.70940000e+00   -1.82972883e-01
 12 2.03920000e+00   -8.17135288e-02
 13 1.14720000e+00   -7.36377500e-01
 14 6.50700000e-01   1.73804270e+00
 15 3.66590000e-01   1.64665928e+00
 16 2.02110000e-01   -2.58713691e+00
 17 1.07420000e-01   -8.17785281e-01
 18 5.42310000e-02   1.25142104e+00
 19 2.56190000e-02   1.96797815e-01
P 8
  1 4.57000000e+01   5.59857700e-04
  2 1.06000000e+01   4.92309360e-03
  3 3.08000000e+00   1.98821499e-02
  4 1.23000000e+00   3.94518210e-02
```

```
  5 5.10000000e-01   1.63060244e-01
  6 2.30000000e-01   4.11157813e-01
  7 1.06000000e-01   4.42934754e-01
  8 4.00000000e-02   6.88676460e-02
P 8
  1 4.57000000e+01   8.86234700e-04
  2 1.06000000e+01   3.06619409e-02
  3 3.08000000e+00   3.19215308e-02
  4 1.23000000e+00   6.31896987e-01
  5 5.10000000e-01   -7.69565173e-01
  6 2.30000000e-01   -1.14099071e+00
  7 1.06000000e-01   1.49192519e+00
  8 4.00000000e-02   9.12650870e-03
D 3
  1 3.35000000e+00   2.50099508e-02
  2 5.70000000e-01   -9.16355551e-02
  3 2.10000000e-01   -9.39409469e-01
end
NewGTO B
S 16
  1 2.10400000e+05   5.84190000e-06
  2 3.15000000e+04   4.54146000e-05
  3 7.16900000e+03   2.38902900e-04
  4 2.03000000e+03   1.00790000e-03
  5 6.62500000e+02   3.65295230e-03
  6 2.39200000e+02   1.17622375e-02
  7 9.32600000e+01   3.38828343e-02
  8 3.86400000e+01   8.57657026e-02
  9 1.67800000e+01   1.83056486e-01
 10 7.54100000e+00   3.06666685e-01
 11 3.48200000e+00   3.42030542e-01
 12 1.61800000e+00   1.76739580e-01
 13 6.27000000e-01   1.61871420e-02
 14 2.93400000e-01   -7.25244260e-03
 15 1.31000000e-01   -4.56611660e-03
 16 5.81500000e-02   -1.73931290e-03
S 16
  1 2.10400000e+05   1.12800000e-06
  2 3.15000000e+04   8.76070000e-06
  3 7.16900000e+03   4.61618000e-05
  4 2.03000000e+03   1.94532100e-04
  5 6.62500000e+02   7.08258000e-04
  6 2.39200000e+02   2.28685660e-03
  7 9.32600000e+01   6.70205100e-03
  8 3.86400000e+01   1.74065585e-02
  9 1.67800000e+01   3.97538712e-02
 10 7.54100000e+00   7.54768239e-02
 11 3.48200000e+00   1.19030784e-01
 12 1.61800000e+00   1.09247041e-01
 13 6.27000000e-01   -1.13752584e-01
 14 2.93400000e-01   -4.40136581e-01
 15 1.31000000e-01   -4.64488538e-01
 16 5.81500000e-02   -1.27588012e-01
S 16
  1 2.10400000e+05   -1.53510000e-06
  2 3.15000000e+04   -1.19591000e-05
  3 7.16900000e+03   -6.27416000e-05
  4 2.03000000e+03   -2.65978800e-04
  5 6.62500000e+02   -9.61774900e-04
  6 2.39200000e+02   -3.13736760e-03
  7 9.32600000e+01   -9.12800700e-03
  8 3.86400000e+01   -2.42286332e-02
  9 1.67800000e+01   -5.53385063e-02
 10 7.54100000e+00   -1.08482786e-01
 11 3.48200000e+00   -1.74847234e-01
 12 1.61800000e+00   -1.93191016e-01
 13 6.27000000e-01   7.38648584e-01
 14 2.93400000e-01   9.64533868e-01
 15 1.31000000e-01   -8.67885485e-01
 16 5.81500000e-02   -5.83913176e-01
S 16
```

```
  1 2.10400000e+05   -2.18410000e-06
  2 3.15000000e+04   -1.56486000e-05
  3 7.16900000e+03   -9.25055000e-05
  4 2.03000000e+03   -3.34241900e-04
  5 6.62500000e+02   -1.47570770e-03
  6 2.39200000e+02   -3.77517520e-03
  7 9.32600000e+01   -1.48890871e-02
  8 3.86400000e+01   -2.86407849e-02
  9 1.67800000e+01   -1.04473207e-01
 10 7.54100000e+00   -1.09560410e-01
 11 3.48200000e+00   -4.18583689e-01
 12 1.61800000e+00   1.39756180e-01
 13 6.27000000e-01   2.40487708e+00
 14 2.93400000e-01   -2.23192255e+00
 15 1.31000000e-01   -8.64037177e-01
 16 5.81500000e-02   1.45525790e+00
P 10
  1 1.92500000e+02   1.34858000e-04
  2 4.56400000e+01   1.14831710e-03
  3 1.47500000e+01   5.84281610e-03
  4 5.50300000e+00   2.10758283e-02
  5 2.22200000e+00   6.11960100e-02
  6 9.59000000e-01   1.53071076e-01
  7 4.31400000e-01   2.94324162e-01
  8 1.96900000e-01   3.64270436e-01
  9 9.03300000e-02   2.58530924e-01
 10 4.06600000e-02   7.17053089e-02
P 10
  1 1.92500000e+02   1.03811900e-04
  2 4.56400000e+01   1.00297990e-03
  3 1.47500000e+01   4.40275030e-03
  4 5.50300000e+00   1.59590908e-02
  5 2.22200000e+00   1.40866372e-02
  6 9.59000000e-01   2.03602506e-01
  7 4.31400000e-01   6.40561072e-01
  8 1.96900000e-01   1.38043328e-02
  9 9.03300000e-02   -7.23955149e-01
 10 4.06600000e-02   -2.86154751e-01
P 10
  1 1.92500000e+02   3.90296000e-04
  2 4.56400000e+01   3.58723880e-03
  3 1.47500000e+01   1.81614638e-02
  4 5.50300000e+00   7.80319375e-02
  5 2.22200000e+00   2.45541210e-01
  6 9.59000000e-01   8.29640072e-01
  7 4.31400000e-01   -2.88444587e-01
  8 1.96900000e-01   -1.15757654e+00
  9 9.03300000e-02   5.47808333e-01
 10 4.06600000e-02   3.67890885e-01
D 5
  1 2.88600000e+00   1.24864080e-02
  2 1.26700000e+00   6.46166325e-02
  3 5.56000000e-01   3.52364034e-01
  4 2.44000000e-01   4.91827218e-01
  5 1.07000000e-01   2.48523180e-01
D 5
  1 2.88600000e+00   -4.49367119e-02
  2 1.26700000e+00   2.53698751e-01
  3 5.56000000e-01   8.65924761e-01
  4 2.44000000e-01   -4.51243401e-01
  5 1.07000000e-01   -5.80249322e-01
F 4
  1 1.65100000e+00   2.61389620e-02
  2 8.00200000e-01   3.04593559e-01
  3 3.87800000e-01   4.97810565e-01
  4 1.88000000e-01   3.21518619e-01
end
NewGTO C
S 16
  1 3.12100000e+05   5.68880000e-06
  2 4.67400000e+04   4.42122000e-05
```

```
  3 1.06400000e+04   2.32471700e-04
  4 3.01300000e+03   9.81398300e-04
  5 9.82800000e+02   3.56048580e-03
  6 3.54800000e+02   1.14692478e-02
  7 1.38400000e+02   3.30825487e-02
  8 5.73500000e+01   8.42739202e-02
  9 2.49200000e+01   1.81183521e-01
 10 1.12300000e+01   3.05872661e-01
 11 5.20100000e+00   3.42899662e-01
 12 2.42600000e+00   1.78520312e-01
 13 9.67300000e-01   1.74929057e-02
 14 4.45600000e-01   -6.93818560e-03
 15 1.97100000e-01   -4.58959260e-03
 16 8.63500000e-02   -1.77870100e-03
S 16
  1 3.12100000e+05   1.15170000e-06
  2 4.67400000e+04   8.94010000e-06
  3 1.06400000e+04   4.71067000e-05
  4 3.01300000e+03   1.98530500e-04
  5 9.82800000e+02   7.24115900e-04
  6 3.54800000e+02   2.33730310e-03
  7 1.38400000e+02   6.86927270e-03
  8 5.73500000e+01   1.79514067e-02
  9 2.49200000e+01   4.14871653e-02
 10 1.12300000e+01   7.95916232e-02
 11 5.20100000e+00   1.25824978e-01
 12 2.42600000e+00   1.06822824e-01
 13 9.67300000e-01   -1.28639081e-01
 14 4.45600000e-01   -4.39866541e-01
 15 1.97100000e-01   -4.46202571e-01
 16 8.63500000e-02   -1.39613780e-01
S 16
  1 3.12100000e+05   1.46150000e-06
  2 4.67400000e+04   1.13996000e-05
  3 1.06400000e+04   5.96491000e-05
  4 3.01300000e+03   2.53756100e-04
  5 9.82800000e+02   9.15187600e-04
  6 3.54800000e+02   2.99970750e-03
  7 1.38400000e+02   8.69022700e-03
  8 5.73500000e+01   2.33513200e-02
  9 2.49200000e+01   5.32963047e-02
 10 1.12300000e+01   1.07142134e-01
 11 5.20100000e+00   1.71571164e-01
 12 2.42600000e+00   1.81050614e-01
 13 9.67300000e-01   -6.61896970e-01
 14 4.45600000e-01   -9.41374990e-01
 15 1.97100000e-01   6.89663676e-01
 16 8.63500000e-02   7.07911640e-01
S 16
  1 3.12100000e+05   -2.07290000e-06
  2 4.67400000e+04   -1.52244000e-05
  3 1.06400000e+04   -8.68378000e-05
  4 3.01300000e+03   -3.29424800e-04
  5 9.82800000e+02   -1.37229620e-03
  6 3.54800000e+02   -3.77954040e-03
  7 1.38400000e+02   -1.36469384e-02
  8 5.73500000e+01   -2.90748718e-02
  9 2.49200000e+01   -9.39954201e-02
 10 1.12300000e+01   -1.27039889e-01
 11 5.20100000e+00   -3.90368865e-01
 12 2.42600000e+00   9.67690209e-02
 13 9.67300000e-01   2.18317118e+00
 14 4.45600000e-01   -1.63805873e+00
 15 1.97100000e-01   -1.36613182e+00
 16 8.63500000e-02   1.60111801e+00
P 10
  1 2.95200000e+02   1.42953600e-04
  2 6.99800000e+01   1.22489070e-03
  3 2.26400000e+01   6.35892390e-03
  4 8.48500000e+00   2.35884808e-02
  5 3.45900000e+00   6.96777731e-02
```

```
  6 1.50400000e+00   1.66446415e-01
  7 6.78300000e-01   2.93356499e-01
  8 3.08700000e-01   3.51201757e-01
  9 1.40000000e-01   2.56386887e-01
 10 6.17800000e-02   7.62169165e-02
P 10
  1 2.95200000e+02   1.64119100e-04
  2 6.99800000e+01   1.50145510e-03
  3 2.26400000e+01   7.37691910e-03
  4 8.48500000e+00   2.84317842e-02
  5 3.45900000e+00   6.74783713e-02
  6 1.50400000e+00   2.85759124e-01
  7 6.78300000e-01   5.58607497e-01
  8 3.08700000e-01   -8.98519083e-02
  9 1.40000000e-01   -6.54650765e-01
 10 6.17800000e-02   -2.60439145e-01
P 10
  1 2.95200000e+02   4.06304700e-04
  2 6.99800000e+01   3.59675900e-03
  3 2.26400000e+01   1.97550020e-02
  4 8.48500000e+00   8.25675839e-02
  5 3.45900000e+00   2.83930168e-01
  6 1.50400000e+00   7.77020255e-01
  7 6.78300000e-01   -4.43564826e-01
  8 3.08700000e-01   -1.03884454e+00
  9 1.40000000e-01   5.73532240e-01
 10 6.17800000e-02   3.86981582e-01
D 5
  1 4.54200000e+00   1.43889048e-02
  2 1.97900000e+00   7.13989420e-02
  3 8.62100000e-01   3.57076052e-01
  4 3.75600000e-01   4.80335413e-01
  5 1.63600000e-01   2.55117645e-01
D 5
  1 4.54200000e+00   -7.63546200e-04
  2 1.97900000e+00   3.07148921e-01
  3 8.62100000e-01   7.74419900e-01
  4 3.75600000e-01   -4.24221517e-01
  5 1.63600000e-01   -5.69104609e-01
F 4
  1 2.63100000e+00   3.34914704e-02
  2 1.25500000e+00   2.98354910e-01
  3 5.98800000e-01   5.16839650e-01
  4 2.85700000e-01   3.06133004e-01
end
NewGTO N
S 16
  1 4.32300000e+05   5.59360000e-06
  2 6.47000000e+04   4.35138000e-05
  3 1.47200000e+04   2.28928700e-04
  4 4.17000000e+03   9.65017100e-04
  5 1.36100000e+03   3.50219040e-03
  6 4.91200000e+02   1.12921163e-02
  7 1.91600000e+02   3.26128265e-02
  8 7.94100000e+01   8.32972697e-02
  9 3.45300000e+01   1.79985658e-01
 10 1.55800000e+01   3.05003513e-01
 11 7.23200000e+00   3.41159312e-01
 12 3.38200000e+00   1.77482697e-01
 13 1.36900000e+00   1.98840957e-02
 14 6.24800000e-01   -1.24663480e-03
 15 2.74700000e-01   1.04010390e-03
 16 1.19200000e-01   -1.26586000e-04
S 16
  1 4.32300000e+05   1.23770000e-06
  2 6.47000000e+04   9.61460000e-06
  3 1.47200000e+04   5.07014000e-05
  4 4.17000000e+03   2.13291100e-04
  5 1.36100000e+03   7.78459400e-04
  6 4.91200000e+02   2.51336340e-03
  7 1.91600000e+02   7.39724800e-03
```

```
  8 7.94100000e+01   1.93531475e-02
  9 3.45300000e+01   4.49446014e-02
 10 1.55800000e+01   8.62431945e-02
 11 7.23200000e+00   1.34250976e-01
 12 3.38200000e+00   1.06013878e-01
 13 1.36900000e+00   -1.38417092e-01
 14 6.24800000e-01   -4.39126760e-01
 15 2.74700000e-01   -4.31343581e-01
 16 1.19200000e-01   -1.50718609e-01
S 16
  1 4.32300000e+05   1.39480000e-06
  2 6.47000000e+04   1.09071000e-05
  3 1.47200000e+04   5.69709000e-05
  4 4.17000000e+03   2.42756300e-04
  5 1.36100000e+03   8.72395100e-04
  6 4.91200000e+02   2.87588280e-03
  7 1.91600000e+02   8.29427670e-03
  8 7.94100000e+01   2.25053948e-02
  9 3.45300000e+01   5.12093507e-02
 10 1.55800000e+01   1.04849727e-01
 11 7.23200000e+00   1.64429024e-01
 12 3.38200000e+00   1.71055872e-01
 13 1.36900000e+00   -5.97499064e-01
 14 6.24800000e-01   -9.10291398e-01
 15 2.74700000e-01   5.23452769e-01
 16 1.19200000e-01   8.20052144e-01
S 16
  1 4.32300000e+05   -2.07200000e-06
  2 6.47000000e+04   -1.53930000e-05
  3 1.47200000e+04   -8.65570000e-05
  4 4.17000000e+03   -3.34467200e-04
  5 1.36100000e+03   -1.35998010e-03
  6 4.91200000e+02   -3.86617150e-03
  7 1.91600000e+02   -1.34702049e-02
  8 7.94100000e+01   -3.00266728e-02
  9 3.45300000e+01   -9.25316587e-02
 10 1.55800000e+01   -1.38615171e-01
 11 7.23200000e+00   -3.82493166e-01
 12 3.38200000e+00   9.54870859e-02
 13 1.36900000e+00   2.04696644e+00
 14 6.24800000e-01   -1.30083367e+00
 15 2.74700000e-01   -1.61851336e+00
 16 1.19200000e-01   1.64264012e+00
P 10
  1 4.15900000e+02   1.49351500e-04
  2 9.86100000e+01   1.28527800e-03
  3 3.19200000e+01   6.74821670e-03
  4 1.20000000e+01   2.54480843e-02
  5 4.91900000e+00   7.55743643e-02
  6 2.14800000e+00   1.75828080e-01
  7 9.69600000e-01   2.96219513e-01
  8 4.39900000e-01   3.44109940e-01
  9 1.97800000e-01   2.50361577e-01
 10 8.60300000e-02   7.63931220e-02
P 10
  1 4.15900000e+02   1.95109000e-04
  2 9.86100000e+01   1.75701950e-03
  3 3.19200000e+01   8.99352330e-03
  4 1.20000000e+01   3.54017865e-02
  5 4.91900000e+00   9.70144192e-02
  6 2.14800000e+00   3.24562521e-01
  7 9.69600000e-01   5.07018547e-01
  8 4.39900000e-01   -1.45468019e-01
  9 1.97800000e-01   -6.20130974e-01
 10 8.60300000e-02   -2.42720658e-01
P 10
  1 4.15900000e+02   4.16269200e-04
  2 9.86100000e+01   3.64253630e-03
  3 3.19200000e+01   2.08775389e-02
  4 1.20000000e+01   8.69515693e-02
  5 4.91900000e+00   3.01954565e-01
```

```
  6 2.14800000e+00   7.48192432e-01
  7 9.69600000e-01   -5.36601609e-01
  8 4.39900000e-01   -9.62402874e-01
  9 1.97800000e-01   5.97938688e-01
 10 8.60300000e-02   3.81282295e-01
D 5
  1 6.71700000e+00   1.53532025e-02
  2 2.89600000e+00   7.39377840e-02
  3 1.24900000e+00   3.61114918e-01
  4 5.38000000e-01   4.78400826e-01
  5 2.32000000e-01   2.54877565e-01
D 5
  1 6.71700000e+00   1.33057296e-02
  2 2.89600000e+00   3.12470144e-01
  3 1.24900000e+00   7.43203706e-01
  4 5.38000000e-01   -3.99952197e-01
  5 2.32000000e-01   -5.77366313e-01
F 4
  1 3.82900000e+00   3.62155054e-02
  2 1.79500000e+00   3.00102167e-01
  3 8.41000000e-01   5.30147509e-01
  4 3.94000000e-01   2.93011802e-01
end
NewGTO O
S 16
  1 5.70800000e+05   5.54770000e-06
  2 8.54800000e+04   4.31114000e-05
  3 1.94600000e+04   2.26687800e-04
  4 5.51200000e+03   9.56446100e-04
  5 1.79800000e+03   3.47348060e-03
  6 6.48900000e+02   1.11986722e-02
  7 2.53100000e+02   3.23902914e-02
  8 1.04900000e+02   8.28666917e-02
  9 4.56500000e+01   1.79599915e-01
 10 2.06200000e+01   3.05252295e-01
 11 9.58700000e+00   3.40941604e-01
 12 4.49300000e+00   1.77462875e-01
 13 1.83700000e+00   2.04404056e-02
 14 8.34900000e-01   -1.12254760e-03
 15 3.65800000e-01   9.01550300e-04
 16 1.57000000e-01   -1.42089000e-04
S 16
  1 5.70800000e+05   1.26190000e-06
  2 8.54800000e+04   9.79420000e-06
  3 1.94600000e+04   5.16082000e-05
  4 5.51200000e+03   2.17376000e-04
  5 1.79800000e+03   7.93634000e-04
  6 6.48900000e+02   2.56368280e-03
  7 2.53100000e+02   7.55406490e-03
  8 1.04900000e+02   1.98252847e-02
  9 4.56500000e+01   4.62226113e-02
 10 2.06200000e+01   8.93623473e-02
 11 9.58700000e+00   1.38343462e-01
 12 4.49300000e+00   1.05181307e-01
 13 1.83700000e+00   -1.49165905e-01
 14 8.34900000e-01   -4.43538639e-01
 15 3.65800000e-01   -4.30421768e-01
 16 1.57000000e-01   -1.39284763e-01
S 16
  1 5.70800000e+05   -1.49560000e-06
  2 8.54800000e+04   -1.16782000e-05
  3 1.94600000e+04   -6.10049000e-05
  4 5.51200000e+03   -2.59966200e-04
  5 1.79800000e+03   -9.35972900e-04
  6 6.48900000e+02   -3.08166350e-03
  7 2.53100000e+02   -8.91883060e-03
  8 1.04900000e+02   -2.42145842e-02
  9 4.56500000e+01   -5.55592008e-02
 10 2.06200000e+01   -1.14527593e-01
 11 9.58700000e+00   -1.82885364e-01
 12 4.49300000e+00   -1.75395843e-01
```

```
 13 1.83700000e+00   7.13860208e-01
 14 8.34900000e-01   8.83104152e-01
 15 3.65800000e-01   -7.22845012e-01
 16 1.57000000e-01   -6.62903050e-01
S 16
  1 5.70800000e+05   -2.10120000e-06
  2 8.54800000e+04   -1.53046000e-05
  3 1.94600000e+04   -8.83188000e-05
  4 5.51200000e+03   -3.29606500e-04
  5 1.79800000e+03   -1.40118000e-03
  6 6.48900000e+02   -3.76970610e-03
  7 2.53100000e+02   -1.40318733e-02
  8 1.04900000e+02   -2.89525787e-02
  9 4.56500000e+01   -9.82062901e-02
 10 2.06200000e+01   -1.30740865e-01
 11 9.58700000e+00   -4.33993969e-01
 12 4.49300000e+00   1.77427381e-01
 13 1.83700000e+00   2.18658844e+00
 14 8.34900000e-01   -1.84886022e+00
 15 3.65800000e-01   -1.06263353e+00
 16 1.57000000e-01   1.46444115e+00
P 10
  1 5.25600000e+02   1.67663100e-04
  2 1.24600000e+02   1.44341600e-03
  3 4.03400000e+01   7.59732890e-03
  4 1.51800000e+01   2.88049894e-02
  5 6.24500000e+00   8.49419814e-02
  6 2.73200000e+00   1.89963117e-01
  7 1.22700000e+00   2.99047865e-01
  8 5.49200000e-01   3.32056996e-01
  9 2.41800000e-01   2.41992085e-01
 10 1.02500000e-01   8.36337245e-02
P 10
  1 5.25600000e+02   2.04132700e-04
  2 1.24600000e+02   1.79921620e-03
  3 4.03400000e+01   9.35497490e-03
  4 1.51800000e+01   3.61612981e-02
  5 6.24500000e+00   1.04785215e-01
  6 2.73200000e+00   3.13224909e-01
  7 1.22700000e+00   4.46483313e-01
  8 5.49200000e-01   -1.16219022e-01
  9 2.41800000e-01   -5.39812159e-01
 10 1.02500000e-01   -3.46845177e-01
P 10
  1 5.25600000e+02   3.39750900e-04
  2 1.24600000e+02   3.05971660e-03
  3 4.03400000e+01   1.66722601e-02
  4 1.51800000e+01   6.91106710e-02
  5 6.24500000e+00   2.24673930e-01
  6 2.73200000e+00   6.38777042e-01
  7 1.22700000e+00   -2.24450373e-01
  8 5.49200000e-01   -9.38196350e-01
  9 2.41800000e-01   1.16475904e-01
 10 1.02500000e-01   7.36644552e-01
D 5
  1 8.25300000e+00   2.04005719e-02
  2 3.59700000e+00   9.41369585e-02
  3 1.56800000e+00   3.96477091e-01
  4 6.84000000e-01   4.55360161e-01
  5 2.98000000e-01   2.20405730e-01
D 5
  1 8.25300000e+00   2.53040956e-02
  2 3.59700000e+00   3.69383220e-01
  3 1.56800000e+00   6.50550865e-01
  4 6.84000000e-01   -4.11645852e-01
  5 2.98000000e-01   -5.80497766e-01
F 4
  1 5.43000000e+00   3.35228290e-02
  2 2.41600000e+00   2.88431165e-01
  3 1.07500000e+00   5.27243780e-01
  4 4.78000000e-01   3.29660778e-01
```

```
end
NewGTO F
S 16
  1 7.23500000e+05   5.55530000e-06
  2 1.08400000e+05   4.31553000e-05
  3 2.46800000e+04   2.26888200e-04
  4 6.99000000e+03   9.57552400e-04
  5 2.28200000e+03   3.46841300e-03
  6 8.24600000e+02   1.11796501e-02
  7 3.21800000e+02   3.23122990e-02
  8 1.33500000e+02   8.27520288e-02
  9 5.81100000e+01   1.79778821e-01
 10 2.62800000e+01   3.05381571e-01
 11 1.22400000e+01   3.39966883e-01
 12 5.74700000e+00   1.76460081e-01
 13 2.36500000e+00   2.11818400e-02
 14 1.07100000e+00   2.01810000e-04
 15 4.68100000e-01   2.01228700e-03
 16 1.99400000e-01   1.86918000e-04
S 16
  1 7.23500000e+05   1.30260000e-06
  2 1.08400000e+05   1.01077000e-05
  3 2.46800000e+04   5.32435000e-05
  4 6.99000000e+03   2.24379700e-04
  5 2.28200000e+03   8.16840000e-04
  6 8.24600000e+02   2.63904770e-03
  7 3.21800000e+02   7.76806640e-03
  8 1.33500000e+02   2.04244295e-02
  9 5.81100000e+01   4.77452371e-02
 10 2.62800000e+01   9.24737947e-02
 11 1.22400000e+01   1.41959438e-01
 12 5.74700000e+00   1.04067345e-01
 13 2.36500000e+00   -1.57152841e-01
 14 1.07100000e+00   -4.45384340e-01
 15 4.68100000e-01   -4.28296901e-01
 16 1.99400000e-01   -1.33422900e-01
S 16
  1 7.23500000e+05   -1.58120000e-06
  2 1.08400000e+05   -1.23348000e-05
  3 2.46800000e+04   -6.44826000e-05
  4 6.99000000e+03   -2.74554700e-04
  5 2.28200000e+03   -9.87382600e-04
  6 8.24600000e+02   -3.24507940e-03
  7 3.21800000e+02   -9.40855790e-03
  8 1.33500000e+02   -2.55267961e-02
  9 5.81100000e+01   -5.90490461e-02
 10 2.62800000e+01   -1.21829593e-01
 11 1.22400000e+01   -1.97106235e-01
 12 5.74700000e+00   -1.74313349e-01
 13 2.36500000e+00   8.01133928e-01
 14 1.07100000e+00   8.43347803e-01
 15 4.68100000e-01   -8.43766683e-01
 16 1.99400000e-01   -5.56541527e-01
S 16
  1 7.23500000e+05   -2.21380000e-06
  2 1.08400000e+05   -1.57194000e-05
  3 2.46800000e+04   -9.39462000e-05
  4 6.99000000e+03   -3.34204000e-04
  5 2.28200000e+03   -1.50315470e-03
  6 8.24600000e+02   -3.74960300e-03
  7 3.21800000e+02   -1.52245069e-02
  8 1.33500000e+02   -2.82133583e-02
  9 5.81100000e+01   -1.08737960e-01
 10 2.62800000e+01   -1.21479459e-01
 11 1.22400000e+01   -5.12410504e-01
 12 5.74700000e+00   3.08944627e-01
 13 2.36500000e+00   2.31713096e+00
 14 1.07100000e+00   -2.43988488e+00
 15 4.68100000e-01   -4.17998152e-01
 16 1.99400000e-01   1.21555675e+00
P 10
```

```
  1 6.60000000e+02   1.78229200e-04
  2 1.56400000e+02   1.53635190e-03
  3 5.06400000e+01   8.12284340e-03
  4 1.90800000e+01   3.09453269e-02
  5 7.87200000e+00   9.08038263e-02
  6 3.44900000e+00   1.97614180e-01
  7 1.54500000e+00   3.00590618e-01
  8 6.86400000e-01   3.26422450e-01
  9 2.98600000e-01   2.39668526e-01
 10 1.24500000e-01   8.12040964e-02
P 10
  1 6.60000000e+02   2.34347800e-04
  2 1.56400000e+02   2.07242600e-03
  3 5.06400000e+01   1.08206813e-02
  4 1.90800000e+01   4.23975838e-02
  5 7.87200000e+00   1.25261742e-01
  6 3.44900000e+00   3.55755402e-01
  7 1.54500000e+00   4.26059781e-01
  8 6.86400000e-01   -1.96265343e-01
  9 2.98600000e-01   -5.53936553e-01
 10 1.24500000e-01   -2.62152264e-01
P 10
  1 6.60000000e+02   3.96333300e-04
  2 1.56400000e+02   3.38304610e-03
  3 5.06400000e+01   1.96902218e-02
  4 1.90800000e+01   7.73666944e-02
  5 7.87200000e+00   2.75392562e-01
  6 3.44900000e+00   6.85583994e-01
  7 1.54500000e+00   -4.98790106e-01
  8 6.86400000e-01   -8.78659405e-01
  9 2.98600000e-01   4.22153705e-01
 10 1.24500000e-01   5.25305084e-01
D 5
  1 1.05730000e+01   2.27863167e-02
  2 4.61300000e+00   1.01470167e-01
  3 2.01300000e+00   4.10653684e-01
  4 8.78000000e-01   4.49088535e-01
  5 3.83000000e-01   2.03354826e-01
D 5
  1 1.05730000e+01   3.38687384e-02
  2 4.61300000e+00   3.93252355e-01
  3 2.01300000e+00   6.20292128e-01
  4 8.78000000e-01   -4.43973787e-01
  5 3.83000000e-01   -5.56278968e-01
F 4
  1 7.56300000e+00   2.56618573e-02
  2 3.33000000e+00   2.72433403e-01
  3 1.46600000e+00   5.42962543e-01
  4 6.45000000e-01   3.33550324e-01
end
NewGTO Ne
S 16
  1 9.02400000e+05   5.50710000e-06
  2 1.35100000e+05   4.28234000e-05
  3 3.07500000e+04   2.25142500e-04
  4 8.71000000e+03   9.50160200e-04
  5 2.84200000e+03   3.44718850e-03
  6 1.02600000e+03   1.11254471e-02
  7 4.00100000e+02   3.22056752e-02
  8 1.65900000e+02   8.25989133e-02
  9 7.22100000e+01   1.79905642e-01
 10 3.26600000e+01   3.06052078e-01
 11 1.52200000e+01   3.40125584e-01
 12 7.14900000e+00   1.76168221e-01
 13 2.95700000e+00   2.10152784e-02
 14 1.33500000e+00   -5.07437900e-04
 15 5.81600000e-01   1.05785740e-03
 16 2.46300000e-01   -5.98773000e-05
S 16
  1 9.02400000e+05   1.29630000e-06
  2 1.35100000e+05   1.00690000e-05
```

```
   3 3.07500000e+04   5.30405000e-05
   4 8.71000000e+03   2.23517100e-04
   5 2.84200000e+03   8.15055900e-04
   6 1.02600000e+03   2.63688220e-03
   7 4.00100000e+02   7.77600830e-03
   8 1.65900000e+02   2.04887059e-02
   9 7.22100000e+01   4.80966546e-02
  10 3.26600000e+01   9.35683962e-02
  11 1.52200000e+01   1.43356072e-01
  12 7.14900000e+00   1.02155876e-01
  13 2.95700000e+00   -1.62934767e-01
  14 1.33500000e+00   -4.46676169e-01
  15 5.81600000e-01   -4.25862512e-01
  16 2.46300000e-01   -1.30306294e-01
S 16
   1 9.02400000e+05   -1.61150000e-06
   2 1.35100000e+05   -1.25800000e-05
   3 3.07500000e+04   -6.57939000e-05
   4 8.71000000e+03   -2.79967400e-04
   5 2.84200000e+03   -1.00926080e-03
   6 1.02600000e+03   -3.31830470e-03
   7 4.00100000e+02   -9.64836690e-03
   8 1.65900000e+02   -2.61882549e-02
   9 7.22100000e+01   -6.09198166e-02
  10 3.26600000e+01   -1.26041391e-01
  11 1.52200000e+01   -2.05488897e-01
  12 7.14900000e+00   -1.71923360e-01
  13 2.95700000e+00   8.48906100e-01
  14 1.33500000e+00   8.13169457e-01
  15 5.81600000e-01   -9.01853688e-01
  16 2.46300000e-01   -4.99925119e-01
S 16
   1 9.02400000e+05   -2.27750000e-06
   2 1.35100000e+05   -1.58878000e-05
   3 3.07500000e+04   -9.74546000e-05
   4 8.71000000e+03   -3.34413100e-04
   5 2.84200000e+03   -1.57289810e-03
   6 1.02600000e+03   -3.71614540e-03
   7 4.00100000e+02   -1.60993168e-02
   8 1.65900000e+02   -2.75130195e-02
   9 7.22100000e+01   -1.16504533e-01
  10 3.26600000e+01   -1.14236147e-01
  11 1.52200000e+01   -5.76611990e-01
  12 7.14900000e+00   4.17635713e-01
  13 2.95700000e+00   2.38840962e+00
  14 1.33500000e+00   -2.83819246e+00
  15 5.81600000e-01   4.97789435e-02
  16 2.46300000e-01   1.01516124e+00
P 10
   1 8.15600000e+02   1.84770500e-04
   2 1.93300000e+02   1.59410930e-03
   3 6.26000000e+01   8.46535310e-03
   4 2.36100000e+01   3.24030611e-02
   5 9.76200000e+00   9.47184717e-02
   6 4.28100000e+00   2.02624168e-01
   7 1.91500000e+00   3.01794970e-01
   8 8.47600000e-01   3.23166976e-01
   9 3.66000000e-01   2.37138589e-01
  10 1.51000000e-01   7.91176295e-02
P 10
   1 8.15600000e+02   2.55486400e-04
   2 1.93300000e+02   2.25791630e-03
   3 6.26000000e+01   1.18809731e-02
   4 2.36100000e+01   4.69683102e-02
   5 9.76200000e+00   1.40960018e-01
   6 4.28100000e+00   3.82289571e-01
   7 1.91500000e+00   4.04957116e-01
   8 8.47600000e-01   -2.48772519e-01
   9 3.66000000e-01   -5.50555989e-01
  10 1.51000000e-01   -2.14656548e-01
P 10
```

```
  1 8.15600000e+02   4.49349300e-04
  2 1.93300000e+02   3.64469480e-03
  3 6.26000000e+01   2.25385482e-02
  4 2.36100000e+01   8.42160085e-02
  5 9.76200000e+00   3.25411552e-01
  6 4.28100000e+00   7.22034611e-01
  7 1.91500000e+00   -7.51414382e-01
  8 8.47600000e-01   -7.61544619e-01
  9 3.66000000e-01   6.36069389e-01
 10 1.51000000e-01   3.27481185e-01
D 5
  1 1.33170000e+01   2.47658998e-02
  2 5.80300000e+00   1.06876555e-01
  3 2.52900000e+00   4.20540693e-01
  4 1.10200000e+00   4.44946874e-01
  5 4.80000000e-01   1.90871284e-01
D 5
  1 1.33170000e+01   4.02982534e-02
  2 5.80300000e+00   4.04860485e-01
  3 2.52900000e+00   5.99892024e-01
  4 1.10200000e+00   -4.61442784e-01
  5 4.80000000e-01   -5.44031357e-01
F 4
  1 1.03560000e+01   1.50197139e-02
  2 4.53800000e+00   2.51242680e-01
  3 1.98900000e+00   5.75622078e-01
  4 8.71000000e-01   3.19708463e-01
end
NewGTO Na
S 21
  1 1.91870000e+06   2.70880000e-06
  2 2.87270000e+05   2.10578000e-05
  3 6.53820000e+04   1.10786800e-04
  4 1.85230000e+04   4.67532600e-04
  5 6.04540000e+03   1.70132840e-03
  6 2.18420000e+03   5.51507460e-03
  7 8.53110000e+02   1.62308700e-02
  8 3.54690000e+02   4.31395655e-02
  9 1.55330000e+02   1.01486023e-01
 10 7.12340000e+01   1.99394305e-01
 11 3.38350000e+01   3.08728896e-01
 12 1.63490000e+01   3.06705230e-01
 13 8.00820000e+00   1.38088158e-01
 14 3.67130000e+00   2.28929786e-02
 15 1.71180000e+00   1.32950975e-02
 16 7.91470000e-01   1.36239139e-02
 17 3.54790000e-01   3.73867660e-03
 18 9.06640000e-02   7.44574000e-05
 19 4.43130000e-02   -9.52170000e-06
 20 2.19480000e-02   2.13706000e-05
 21 1.08210000e-02   0.00000000e+00
S 21
  1 1.91870000e+06   7.48500000e-07
  2 2.87270000e+05   5.81770000e-06
  3 6.53820000e+04   3.06178000e-05
  4 1.85230000e+04   1.29225900e-04
  5 6.04540000e+03   4.70970800e-04
  6 2.18420000e+03   1.53073460e-03
  7 8.53110000e+02   4.54125400e-03
  8 3.54690000e+02   1.22699314e-02
  9 1.55330000e+02   2.99593063e-02
 10 7.12340000e+01   6.33214284e-02
 11 3.38350000e+01   1.14295475e-01
 12 1.63490000e+01   1.54163786e-01
 13 8.00820000e+00   8.93386546e-02
 14 3.67130000e+00   -1.65977411e-01
 15 1.71180000e+00   -4.38423509e-01
 16 7.91470000e-01   -4.29104953e-01
 17 3.54790000e-01   -1.29316724e-01
 18 9.06640000e-02   4.40490600e-04
 19 4.43130000e-02   -2.47939490e-03
```

```
 20 2.19480000e-02   9.62642900e-04
 21 1.08210000e-02   -4.04101000e-04
S 21
  1 1.91870000e+06   1.09600000e-07
  2 2.87270000e+05   8.51300000e-07
  3 6.53820000e+04   4.48290000e-06
  4 1.85230000e+04   1.89095000e-05
  5 6.04540000e+03   6.89893000e-05
  6 2.18420000e+03   2.24129900e-04
  7 8.53110000e+02   6.66689500e-04
  8 3.54690000e+02   1.80444930e-03
  9 1.55330000e+02   4.44375000e-03
 10 7.12340000e+01   9.49314110e-03
 11 3.38350000e+01   1.76175702e-02
 12 1.63490000e+01   2.47238067e-02
 13 8.00820000e+00   1.50685595e-02
 14 3.67130000e+00   -3.26908857e-02
 15 1.71180000e+00   -9.05675951e-02
 16 7.91470000e-01   -1.27595009e-01
 17 3.54790000e-01   -1.22199518e-01
 18 9.06640000e-02   2.66615947e-01
 19 4.43130000e-02   5.62246346e-01
 20 2.19480000e-02   2.79758335e-01
 21 1.08210000e-02   1.84296293e-02
S 21
  1 1.91870000e+06   -9.12100000e-07
  2 2.87270000e+05   -7.05970000e-06
  3 6.53820000e+04   -3.73862000e-05
  4 1.85230000e+04   -1.56574600e-04
  5 6.04540000e+03   -5.77045300e-04
  6 2.18420000e+03   -1.85767720e-03
  7 8.53110000e+02   -5.64162320e-03
  8 3.54690000e+02   -1.53304856e-02
  9 1.55330000e+02   -4.00233925e-02
 10 7.12340000e+01   -8.43610950e-02
 11 3.38350000e+01   -1.52656528e-01
 12 1.63490000e+01   -2.12193144e-01
 13 8.00820000e+00   -1.08260808e-01
 14 3.67130000e+00   9.47407488e-01
 15 1.71180000e+00   7.69196129e-01
 16 7.91470000e-01   -9.53788381e-01
 17 3.54790000e-01   -5.97900103e-01
 18 9.06640000e-02   4.63503976e-02
 19 4.43130000e-02   2.18619967e-01
 20 2.19480000e-02   8.03942641e-02
 21 1.08210000e-02   9.83466700e-03
S 21
  1 1.91870000e+06   2.03180000e-06
  2 2.87270000e+05   1.61964000e-05
  3 6.53820000e+04   8.22300000e-05
  4 1.85230000e+04   3.64715300e-04
  5 6.04540000e+03   1.25846160e-03
  6 2.18420000e+03   4.47591370e-03
  7 8.53110000e+02   1.28756216e-02
  8 3.54690000e+02   4.44201519e-02
  9 1.55330000e+02   1.24991099e-01
 10 7.12340000e+01   2.83631891e-01
 11 3.38350000e+01   1.68789391e-01
 12 1.63490000e+01   1.87955419e-01
 13 8.00820000e+00   -8.57604112e-01
 14 3.67130000e+00   -1.25413242e+00
 15 1.71180000e+00   1.97935541e+00
 16 7.91470000e-01   4.18323562e-01
 17 3.54790000e-01   -1.26590249e+00
 18 9.06640000e-02   -2.17394423e-01
 19 4.43130000e-02   4.80560744e-01
 20 2.19480000e-02   1.03859080e-03
 21 1.08210000e-02   4.03416859e-02
P 15
  1 3.04420000e+03   2.81041000e-05
  2 7.20020000e+02   2.56512000e-04
```

```
  3 2.34420000e+02   1.41776520e-03
  4 8.96660000e+01   6.39168250e-03
  5 3.66950000e+01   2.31537137e-02
  6 1.62560000e+01   6.52632255e-02
  7 7.65930000e+00   1.44068059e-01
  8 3.71250000e+00   2.42657238e-01
  9 1.79920000e+00   3.06650146e-01
 10 8.62880000e-01   2.85522924e-01
 11 4.01040000e-01   1.56904919e-01
 12 1.77590000e-01   2.50223117e-02
 13 8.07000000e-02   9.79528500e-04
 14 3.67000000e-02   1.43310800e-04
 15 1.67000000e-02   3.82855000e-05
P 15
  1 3.04420000e+03   4.22160000e-05
  2 7.20020000e+02   3.89098500e-04
  3 2.34420000e+02   2.22141080e-03
  4 8.96660000e+01   1.03861947e-02
  5 3.66950000e+01   3.69679527e-02
  6 1.62560000e+01   1.02091536e-01
  7 7.65930000e+00   2.60145450e-01
  8 3.71250000e+00   4.51233315e-01
  9 1.79920000e+00   1.11611823e-01
 10 8.62880000e-01   -4.76591870e-01
 11 4.01040000e-01   -4.16152570e-01
 12 1.77590000e-01   -9.94949247e-02
 13 8.07000000e-02   -1.17309641e-02
 14 3.67000000e-02   -3.91016680e-03
 15 1.67000000e-02   2.09826000e-04
D 3
  1 2.35000000e+00   8.48536377e-01
  2 5.60000000e-01   2.80121913e-01
  3 1.40000000e-01   -1.51540850e-02
end
NewGTO Mg
S 21
  1 3.19910000e+06   1.79500000e-06
  2 4.79000000e+05   1.39619000e-05
  3 1.09010000e+05   7.34120000e-05
  4 3.08780000e+04   3.10361400e-04
  5 1.00750000e+04   1.12952010e-03
  6 3.63790000e+03   3.68251510e-03
  7 1.41950000e+03   1.09059255e-02
  8 5.89350000e+02   2.95927497e-02
  9 2.57530000e+02   7.21890156e-02
 10 1.17170000e+02   1.55264953e-01
 11 5.47620000e+01   2.72711082e-01
 12 2.62880000e+01   3.34448771e-01
 13 1.28600000e+01   2.19916844e-01
 14 6.26040000e+00   4.96777160e-02
 15 2.85140000e+00   2.76015740e-03
 16 1.30580000e+00   8.14324200e-04
 17 5.83310000e-01   7.60996000e-05
 18 1.68490000e-01   2.36754000e-05
 19 8.56460000e-02   -2.06013000e-05
 20 4.50770000e-02   1.10197000e-05
 21 2.19340000e-02   -2.70940000e-06
S 21
  1 3.19910000e+06   4.53700000e-07
  2 4.79000000e+05   3.52560000e-06
  3 1.09010000e+05   1.85673000e-05
  4 3.08780000e+04   7.83671000e-05
  5 1.00750000e+04   2.86187000e-04
  6 3.63790000e+03   9.32526900e-04
  7 1.41950000e+03   2.78865710e-03
  8 5.89350000e+02   7.63924590e-03
  9 2.57530000e+02   1.93013967e-02
 10 1.17170000e+02   4.38512116e-02
 11 5.47620000e+01   8.80107485e-02
 12 2.62880000e+01   1.37273971e-01
 13 1.28600000e+01   1.36976362e-01
```

```
 14 6.26040000e+00   -4.20379328e-02
 15 2.85140000e+00   -3.72787567e-01
 16 1.30580000e+00   -5.19014763e-01
 17 5.83310000e-01   -2.11210262e-01
 18 1.68490000e-01   -2.78178800e-03
 19 8.56460000e-02   -1.58740920e-03
 20 4.50770000e-02   1.42578440e-03
 21 2.19340000e-02   -4.18862200e-04
S 21
  1 3.19910000e+06   8.75000000e-08
  2 4.79000000e+05   6.78800000e-07
  3 1.09010000e+05   3.58080000e-06
  4 3.08780000e+04   1.50794000e-05
  5 1.00750000e+04   5.52299000e-05
  6 3.63790000e+03   1.79363900e-04
  7 1.41950000e+03   5.38857700e-04
  8 5.89350000e+02   1.47013010e-03
  9 2.57530000e+02   3.74429760e-03
 10 1.17170000e+02   8.48067780e-03
 11 5.47620000e+01   1.73277526e-02
 12 2.62880000e+01   2.71678567e-02
 13 1.28600000e+01   2.86523167e-02
 14 6.26040000e+00   -1.04754726e-02
 15 2.85140000e+00   -8.57137348e-02
 16 1.30580000e+00   -1.68548085e-01
 17 5.83310000e-01   -1.67989351e-01
 18 1.68490000e-01   2.25842419e-01
 19 8.56460000e-02   4.54327383e-01
 20 4.50770000e-02   3.90225285e-01
 21 2.19340000e-02   8.48474620e-02
S 21
  1 3.19910000e+06   2.80000000e-08
  2 4.79000000e+05   2.39500000e-07
  3 1.09010000e+05   1.09450000e-06
  4 3.08780000e+04   5.54920000e-06
  5 1.00750000e+04   1.60017000e-05
  6 3.63790000e+03   6.91337000e-05
  7 1.41950000e+03   1.45838200e-04
  8 5.89350000e+02   5.99166500e-04
  9 2.57530000e+02   9.15827800e-04
 10 1.17170000e+02   3.74560390e-03
 11 5.47620000e+01   3.36867780e-03
 12 2.62880000e+01   1.48488419e-02
 13 1.28600000e+01   -2.94189980e-03
 14 6.26040000e+00   2.03771606e-02
 15 2.85140000e+00   -7.55531699e-02
 16 1.30580000e+00   3.31803053e-02
 17 5.83310000e-01   -2.10262284e-01
 18 1.68490000e-01   8.01690086e-01
 19 8.56460000e-02   -2.13440679e+00
 20 4.50770000e-02   4.23794935e+00
 21 2.19340000e-02   -3.17425812e+00
S 21
  1 3.19910000e+06   6.47000000e-08
  2 4.79000000e+05   4.37600000e-07
  3 1.09010000e+05   2.80090000e-06
  4 3.08780000e+04   9.06210000e-06
  5 1.00750000e+04   4.57595000e-05
  6 3.63790000e+03   9.86988000e-05
  7 1.41950000e+03   4.76389300e-04
  8 5.89350000e+02   7.14558500e-04
  9 2.57530000e+02   3.59490610e-03
 10 1.17170000e+02   3.28477410e-03
 11 5.47620000e+01   1.91881365e-02
 12 2.62880000e+01   2.27814460e-03
 13 1.28600000e+01   5.66985070e-02
 14 6.26040000e+00   -7.72645697e-02
 15 2.85140000e+00   7.66595026e-02
 16 1.30580000e+00   -3.92945801e-01
 17 5.83310000e-01   3.20841673e-01
 18 1.68490000e-01   -2.18148721e+00
```

```
 19 8.56460000e-02   8.02815458e+00
 20 4.50770000e-02   -8.92239058e+00
 21 2.19340000e-02   3.15527468e+00
P 15
  1 4.15780000e+03   2.36086000e-05
  2 9.84530000e+02   2.17246800e-04
  3 3.20090000e+02   1.20606720e-03
  4 1.21730000e+02   5.61654260e-03
  5 4.98920000e+01   2.02158577e-02
  6 2.25420000e+01   5.66094004e-02
  7 1.07490000e+01   1.30038527e-01
  8 5.28990000e+00   2.29131731e-01
  9 2.59960000e+00   3.14520771e-01
 10 1.25180000e+00   3.05194501e-01
 11 5.92460000e-01   1.53594898e-01
 12 2.69410000e-01   2.25671416e-02
 13 1.22500000e-01   1.13636480e-03
 14 5.57000000e-02   2.59697200e-04
 15 2.53000000e-02   1.37661000e-05
P 15
  1 4.15780000e+03   1.41670000e-06
  2 9.84530000e+02   1.92918000e-05
  3 3.20090000e+02   7.35564000e-05
  4 1.21730000e+02   4.91881800e-04
  5 4.98920000e+01   1.25317580e-03
  6 2.25420000e+01   5.17293210e-03
  7 1.07490000e+01   7.55102310e-03
  8 5.28990000e+00   2.36912477e-02
  9 2.59960000e+00   1.20432758e-02
 10 1.25180000e+00   5.26499427e-02
 11 5.92460000e-01   -4.00496777e-02
 12 2.69410000e-01   8.31726078e-02
 13 1.22500000e-01   -3.81744210e-01
 14 5.57000000e-02   3.58175916e-01
 15 2.53000000e-02   -1.11581282e+00
D 3
  1 3.36000000e+00   2.16295093e-02
  2 8.00000000e-01   4.97381850e-03
  3 2.00000000e-01   9.96002292e-01
end
NewGTO Al
S 21
  1 3.65200000e+06   1.86370000e-06
  2 5.46800000e+05   1.44635000e-05
  3 1.24500000e+05   7.61824000e-05
  4 3.54400000e+04   3.15823500e-04
  5 1.18400000e+04   1.09738740e-03
  6 4.43400000e+03   3.36970980e-03
  7 1.81200000e+03   9.32218810e-03
  8 7.91500000e+02   2.37993006e-02
  9 3.61000000e+02   5.68193211e-02
 10 1.69500000e+02   1.22468847e-01
 11 8.16800000e+01   2.23897590e-01
 12 4.02800000e+01   3.13444677e-01
 13 2.02500000e+01   2.74977258e-01
 14 1.02300000e+01   1.10573391e-01
 15 4.80200000e+00   1.19196229e-02
 16 2.33900000e+00   6.36932200e-04
 17 1.16300000e+00   4.48769200e-04
 18 5.88200000e-01   -3.80120000e-05
 19 2.31100000e-01   5.93791000e-05
 20 1.02700000e-01   5.87612000e-05
 21 4.52100000e-02   2.61452000e-05
S 21
  1 3.65200000e+06   4.84800000e-07
  2 5.46800000e+05   3.76070000e-06
  3 1.24500000e+05   1.98230000e-05
  4 3.54400000e+04   8.21362000e-05
  5 1.18400000e+04   2.85993600e-04
  6 4.43400000e+03   8.78972800e-04
  7 1.81200000e+03   2.44968390e-03
```

```
  8 7.91500000e+02   6.31372180e-03
  9 3.61000000e+02   1.54946400e-02
 10 1.69500000e+02   3.49794080e-02
 11 8.16800000e+01   7.08150170e-02
 12 4.02800000e+01   1.19490376e-01
 13 2.02500000e+01   1.48928590e-01
 14 1.02300000e+01   5.90936008e-02
 15 4.80200000e+00   -2.16754778e-01
 16 2.33900000e+00   -4.76900610e-01
 17 1.16300000e+00   -3.76454511e-01
 18 5.88200000e-01   -8.70507052e-02
 19 2.31100000e-01   -1.25539970e-03
 20 1.02700000e-01   2.27551840e-03
 21 4.52100000e-02   5.97803400e-04
S 21
  1 3.65200000e+06   1.10700000e-07
  2 5.46800000e+05   8.59100000e-07
  3 1.24500000e+05   4.52550000e-06
  4 3.54400000e+04   1.87694000e-05
  5 1.18400000e+04   6.52773000e-05
  6 4.43400000e+03   2.00987800e-04
  7 1.81200000e+03   5.59222300e-04
  8 7.91500000e+02   1.44638300e-03
  9 3.61000000e+02   3.54559880e-03
 10 1.69500000e+02   8.06441740e-03
 11 8.16800000e+01   1.63827254e-02
 12 4.02800000e+01   2.82463510e-02
 13 2.02500000e+01   3.59202031e-02
 14 1.02300000e+01   1.51042507e-02
 15 4.80200000e+00   -6.05797083e-02
 16 2.33900000e+00   -1.50845870e-01
 17 1.16300000e+00   -2.19775043e-01
 18 5.88200000e-01   -9.45734630e-02
 19 2.31100000e-01   4.00909611e-01
 20 1.02700000e-01   6.09838548e-01
 21 4.52100000e-02   1.81242505e-01
S 21
  1 3.65200000e+06   1.49600000e-07
  2 5.46800000e+05   1.17350000e-06
  3 1.24500000e+05   6.08730000e-06
  4 3.54400000e+04   2.57771000e-05
  5 1.18400000e+04   8.71996000e-05
  6 4.43400000e+03   2.78364200e-04
  7 1.81200000e+03   7.39521600e-04
  8 7.91500000e+02   2.02563180e-03
  9 3.61000000e+02   4.64223630e-03
 10 1.69500000e+02   1.14436286e-02
 11 8.16800000e+01   2.11580673e-02
 12 4.02800000e+01   4.14650571e-02
 13 2.02500000e+01   4.35211688e-02
 14 1.02300000e+01   3.24891435e-02
 15 4.80200000e+00   -1.19650402e-01
 16 2.33900000e+00   -1.60003196e-01
 17 1.16300000e+00   -5.19835133e-01
 18 5.88200000e-01   1.66599052e-01
 19 2.31100000e-01   1.84383214e+00
 20 1.02700000e-01   -8.37723991e-01
 21 4.52100000e-02   -7.63734360e-01
S 21
  1 3.65200000e+06   2.06100000e-07
  2 5.46800000e+05   1.71530000e-06
  3 1.24500000e+05   8.14900000e-06
  4 3.54400000e+04   3.87797000e-05
  5 1.18400000e+04   1.11847200e-04
  6 4.43400000e+03   4.37120400e-04
  7 1.81200000e+03   8.87074100e-04
  8 7.91500000e+02   3.35299490e-03
  9 3.61000000e+02   5.17038220e-03
 10 1.69500000e+02   2.00182998e-02
 11 8.16800000e+01   2.08212154e-02
 12 4.02800000e+01   8.17328395e-02
```

```
 13 2.02500000e+01   1.53743546e-02
 14 1.02300000e+01   1.37609815e-01
 15 4.80200000e+00   -4.14141440e-01
 16 2.33900000e+00   2.14720916e-01
 17 1.16300000e+00   -2.09270766e+00
 18 5.88200000e-01   3.51390605e+00
 19 2.31100000e-01   -8.39589520e-01
 20 1.02700000e-01   -2.19068436e+00
 21 4.52100000e-02   1.83762412e+00
P 14
  1 2.88400000e+03   6.38513000e-05
  2 6.83200000e+02   5.63618100e-04
  3 2.22000000e+02   3.17209690e-03
  4 8.48200000e+01   1.32523750e-02
  5 3.58100000e+01   4.33836653e-02
  6 1.62200000e+01   1.12060310e-01
  7 7.70200000e+00   2.18014276e-01
  8 3.74100000e+00   3.11943174e-01
  9 1.83100000e+00   3.16943976e-01
 10 8.87800000e-01   1.78925133e-01
 11 3.98900000e-01   3.09903761e-02
 12 1.71800000e-01   1.20673500e-04
 13 7.29800000e-02   6.68944900e-04
 14 3.06900000e-02   -1.26314900e-04
P 14
  1 2.88400000e+03   1.20316000e-05
  2 6.83200000e+02   1.06831000e-04
  3 2.22000000e+02   5.99099500e-04
  4 8.48200000e+01   2.52558480e-03
  5 3.58100000e+01   8.29114610e-03
  6 1.62200000e+01   2.18958429e-02
  7 7.70200000e+00   4.30126547e-02
  8 3.74100000e+00   6.33327606e-02
  9 1.83100000e+00   6.77298350e-02
 10 8.87800000e-01   4.17050279e-02
 11 3.98900000e-01   -1.30263600e-01
 12 1.71800000e-01   -4.20265513e-01
 13 7.29800000e-02   -4.49846925e-01
 14 3.06900000e-02   -1.54440166e-01
P 14
  1 2.88400000e+03   1.42841000e-05
  2 6.83200000e+02   1.36056600e-04
  3 2.22000000e+02   7.11550200e-04
  4 8.48200000e+01   3.21614900e-03
  5 3.58100000e+01   9.73602490e-03
  6 1.62200000e+01   2.78786993e-02
  7 7.70200000e+00   4.98714840e-02
  8 3.74100000e+00   8.77150859e-02
  9 1.83100000e+00   7.07128658e-02
 10 8.87800000e-01   1.57537627e-01
 11 3.98900000e-01   -4.80977977e-01
 12 1.71800000e-01   -8.34821097e-01
 13 7.29800000e-02   7.14980598e-01
 14 3.06900000e-02   4.86129239e-01
P 14
  1 2.88400000e+03   2.38482000e-05
  2 6.83200000e+02   1.94281400e-04
  3 2.22000000e+02   1.18801140e-03
  4 8.48200000e+01   4.59894110e-03
  5 3.58100000e+01   1.63337058e-02
  6 1.62200000e+01   3.82089444e-02
  7 7.70200000e+00   9.42740455e-02
  8 3.74100000e+00   1.11042689e-01
  9 1.83100000e+00   1.90893453e-01
 10 8.87800000e-01   1.36933213e-01
 11 3.98900000e-01   -1.87225306e+00
 12 1.71800000e-01   1.65161071e+00
 13 7.29800000e-02   2.22042391e-01
 14 3.06900000e-02   -8.23645039e-01
D 5
  1 2.21430000e+00   1.78097303e-02
```

```
  2 9.44900000e-01   1.04024313e-02
  3 4.03200000e-01   2.59473672e-01
  4 1.72100000e-01   5.57049317e-01
  5 7.34300000e-02   3.06491357e-01
D 5
  1 2.21430000e+00   8.12809320e-03
  2 9.44900000e-01   -7.80708823e-02
  3 4.03200000e-01   8.75507330e-01
  4 1.72100000e-01   7.40558985e-02
  5 7.34300000e-02   -9.04595539e-01
F 4
  1 8.75600000e-01   -1.56499578e-02
  2 4.47200000e-01   2.90304534e-01
  3 2.28400000e-01   4.53639208e-01
  4 1.16700000e-01   3.91701193e-01
end
NewGTO Si
S 21
  1 4.46500000e+06   1.74730000e-06
  2 6.68500000e+05   1.35737000e-05
  3 1.52200000e+05   7.14074000e-05
  4 4.33000000e+04   2.97258300e-04
  5 1.44100000e+04   1.03828330e-03
  6 5.39400000e+03   3.17467540e-03
  7 2.21200000e+03   8.73249580e-03
  8 9.68100000e+02   2.23831560e-02
  9 4.41200000e+02   5.37277388e-02
 10 2.07100000e+02   1.16649627e-01
 11 9.98000000e+01   2.15980191e-01
 12 4.92400000e+01   3.09568372e-01
 13 2.47400000e+01   2.83949358e-01
 14 1.24700000e+01   1.22238608e-01
 15 5.79500000e+00   1.41879507e-02
 16 2.83000000e+00   2.86092300e-04
 17 1.40700000e+00   5.80965400e-04
 18 6.99500000e-01   -1.36262400e-04
 19 3.08300000e-01   1.13232400e-04
 20 1.38500000e-01   5.83027000e-05
 21 6.14500000e-02   3.34760000e-05
S 21
  1 4.46500000e+06   4.65600000e-07
  2 6.68500000e+05   3.61540000e-06
  3 1.52200000e+05   1.90351000e-05
  4 4.33000000e+04   7.91894000e-05
  5 1.44100000e+04   2.77208300e-04
  6 5.39400000e+03   8.48109500e-04
  7 2.21200000e+03   2.35032400e-03
  8 9.68100000e+02   6.07697460e-03
  9 4.41200000e+02   1.49870123e-02
 10 2.07100000e+02   3.40090641e-02
 11 9.98000000e+01   6.95329490e-02
 12 4.92400000e+01   1.19129204e-01
 13 2.47400000e+01   1.53814185e-01
 14 1.24700000e+01   7.05550699e-02
 15 5.79500000e+00   -2.13382841e-01
 16 2.83000000e+00   -4.92337286e-01
 17 1.40700000e+00   -3.79518818e-01
 18 6.99500000e-01   -7.60495636e-02
 19 3.08300000e-01   2.91889700e-04
 20 1.38500000e-01   2.26994070e-03
 21 6.14500000e-02   7.92093300e-04
S 21
  1 4.46500000e+06   1.17400000e-07
  2 6.68500000e+05   9.11700000e-07
  3 1.52200000e+05   4.79790000e-06
  4 4.33000000e+04   1.99725000e-05
  5 1.44100000e+04   6.98666000e-05
  6 5.39400000e+03   2.14013100e-04
  7 2.21200000e+03   5.92574000e-04
  8 9.68100000e+02   1.53611170e-03
  9 4.41200000e+02   3.78886050e-03
```

```
 10 2.07100000e+02   8.65221810e-03
 11 9.98000000e+01   1.77881630e-02
 12 4.92400000e+01   3.10894097e-02
 13 2.47400000e+01   4.11817036e-02
 14 1.24700000e+01   1.98252138e-02
 15 5.79500000e+00   -6.61548777e-02
 16 2.83000000e+00   -1.80055889e-01
 17 1.40700000e+00   -2.58650133e-01
 18 6.99500000e-01   -6.34512383e-02
 19 3.08300000e-01   4.45814763e-01
 20 1.38500000e-01   5.86713523e-01
 21 6.14500000e-02   1.71335593e-01
S 21
  1 4.46500000e+06   1.54400000e-07
  2 6.68500000e+05   1.20970000e-06
  3 1.52200000e+05   6.28680000e-06
  4 4.33000000e+04   2.66189000e-05
  5 1.44100000e+04   9.10384000e-05
  6 5.39400000e+03   2.87271400e-04
  7 2.21200000e+03   7.65629100e-04
  8 9.68100000e+02   2.08105590e-03
  9 4.41200000e+02   4.85934250e-03
 10 2.07100000e+02   1.18522651e-02
 11 9.98000000e+01   2.26106642e-02
 12 4.92400000e+01   4.38278795e-02
 13 2.47400000e+01   5.04887020e-02
 14 1.24700000e+01   3.62050687e-02
 15 5.79500000e+00   -1.27092864e-01
 16 2.83000000e+00   -2.00125671e-01
 17 1.40700000e+00   -5.74217932e-01
 18 6.99500000e-01   3.05577965e-01
 19 3.08300000e-01   1.79870833e+00
 20 1.38500000e-01   -8.74698722e-01
 21 6.14500000e-02   -7.51939794e-01
S 21
  1 4.46500000e+06   2.05000000e-07
  2 6.68500000e+05   1.64730000e-06
  3 1.52200000e+05   8.24790000e-06
  4 4.33000000e+04   3.67054000e-05
  5 1.44100000e+04   1.17408700e-04
  6 5.39400000e+03   4.04067900e-04
  7 2.21200000e+03   9.61205500e-04
  8 9.68100000e+02   3.00051730e-03
  9 4.41200000e+02   5.95155910e-03
 10 2.07100000e+02   1.75729444e-02
 11 9.98000000e+01   2.67914016e-02
 12 4.92400000e+01   6.91625792e-02
 13 2.47400000e+01   5.04674095e-02
 14 1.24700000e+01   8.44721410e-02
 15 5.79500000e+00   -2.94216400e-01
 16 2.83000000e+00   -1.27235735e-01
 17 1.40700000e+00   -1.59825158e+00
 18 6.99500000e-01   3.50880490e+00
 19 3.08300000e-01   -1.09920288e+00
 20 1.38500000e-01   -2.24128054e+00
 21 6.14500000e-02   1.90711389e+00
P 14
  1 3.57200000e+03   5.98101000e-05
  2 8.46000000e+02   5.29019700e-04
  3 2.74800000e+02   2.99272640e-03
  4 1.05000000e+02   1.26203482e-02
  5 4.43500000e+01   4.18585003e-02
  6 2.00800000e+01   1.10129727e-01
  7 9.53000000e+00   2.18574670e-01
  8 4.63400000e+00   3.17480603e-01
  9 2.28000000e+00   3.19330889e-01
 10 1.11600000e+00   1.71414596e-01
 11 4.99100000e-01   2.72061913e-02
 12 2.25400000e-01   -3.24650000e-04
 13 1.00100000e-01   8.25436900e-04
 14 4.33200000e-02   -1.20198300e-04
```

```
P 14
  1 3.57200000e+03   1.30489000e-05
  2 8.46000000e+02   1.15334100e-04
  3 2.74800000e+02   6.54562100e-04
  4 1.05000000e+02   2.76796380e-03
  5 4.43500000e+01   9.28172850e-03
  6 2.00800000e+01   2.48181605e-02
  7 9.53000000e+00   5.04241847e-02
  8 4.63400000e+00   7.44168937e-02
  9 2.28000000e+00   8.17489341e-02
 10 1.11600000e+00   3.08043509e-02
 11 4.99100000e-01   -1.78172493e-01
 12 2.25400000e-01   -4.29742808e-01
 13 1.00100000e-01   -4.11530218e-01
 14 4.33200000e-02   -1.31675147e-01
P 14
  1 3.57200000e+03   1.57401000e-05
  2 8.46000000e+02   1.44740100e-04
  3 2.74800000e+02   7.90095900e-04
  4 1.05000000e+02   3.47530840e-03
  5 4.43500000e+01   1.11422064e-02
  6 2.00800000e+01   3.10943218e-02
  7 9.53000000e+00   6.07776749e-02
  8 4.63400000e+00   9.78451850e-02
  9 2.28000000e+00   9.62138762e-02
 10 1.11600000e+00   1.23953056e-01
 11 4.99100000e-01   -6.35775221e-01
 12 2.25400000e-01   -6.60206529e-01
 13 1.00100000e-01   7.63210791e-01
 14 4.33200000e-02   4.37019177e-01
P 14
  1 3.57200000e+03   3.00647000e-05
  2 8.46000000e+02   2.01963100e-04
  3 2.74800000e+02   1.50816700e-03
  4 1.05000000e+02   4.88658490e-03
  5 4.43500000e+01   2.17118781e-02
  6 2.00800000e+01   4.16318863e-02
  7 9.53000000e+00   1.35924311e-01
  8 4.63400000e+00   9.44419852e-02
  9 2.28000000e+00   3.17748880e-01
 10 1.11600000e+00   -2.72994296e-01
 11 4.99100000e-01   -1.64660972e+00
 12 2.25400000e-01   1.94010667e+00
 13 1.00100000e-01   -9.99669875e-02
 14 4.33200000e-02   -7.31844930e-01
D 5
  1 3.23860000e+00   1.97751361e-02
  2 1.37670000e+00   1.50954672e-02
  3 5.85300000e-01   2.57236119e-01
  4 2.48800000e-01   5.56047232e-01
  5 1.05800000e-01   3.08114757e-01
D 5
  1 3.23860000e+00   4.73247960e-03
  2 1.37670000e+00   -9.63416463e-02
  3 5.85300000e-01   8.70203551e-01
  4 2.48800000e-01   1.00273984e-01
  5 1.05800000e-01   -9.21418942e-01
F 4
  1 1.35100000e+00   -1.31724073e-02
  2 6.60000000e-01   2.47459738e-01
  3 3.22500000e-01   5.29316438e-01
  4 1.57500000e-01   3.56207230e-01
end
NewGTO P
S 21
  1 5.38400000e+06   1.64570000e-06
  2 8.06200000e+05   1.27815000e-05
  3 1.83600000e+05   6.72065000e-05
  4 5.22500000e+04   2.79710900e-04
  5 1.73900000e+04   9.76699800e-04
  6 6.52300000e+03   2.96834920e-03
```

```
  7 2.68700000e+03   8.12399770e-03
  8 1.17800000e+03   2.09200106e-02
  9 5.36200000e+02   5.05590328e-02
 10 2.51500000e+02   1.10479103e-01
 11 1.21300000e+02   2.06956912e-01
 12 5.98800000e+01   3.04737341e-01
 13 3.00500000e+01   2.92952311e-01
 14 1.51200000e+01   1.35560712e-01
 15 7.01000000e+00   1.73207816e-02
 16 3.44100000e+00   -3.51698000e-05
 17 1.71200000e+00   8.03262000e-04
 18 8.33700000e-01   -2.43839500e-04
 19 3.91200000e-01   9.87220000e-05
 20 1.77700000e-01   -5.12279000e-05
 21 7.93900000e-02   1.00911000e-05
S 21
  1 5.38400000e+06   4.46900000e-07
  2 8.06200000e+05   3.46950000e-06
  3 1.83600000e+05   1.82569000e-05
  4 5.22500000e+04   7.59386000e-05
  5 1.73900000e+04   2.65723600e-04
  6 6.52300000e+03   8.08045300e-04
  7 2.68700000e+03   2.22732830e-03
  8 1.17800000e+03   5.78331990e-03
  9 5.36200000e+02   1.43437812e-02
 10 2.51500000e+02   3.27060584e-02
 11 1.21300000e+02   6.73715266e-02
 12 5.98800000e+01   1.17647137e-01
 13 3.00500000e+01   1.57279812e-01
 14 1.51200000e+01   8.38541752e-02
 15 7.01000000e+00   -1.99717185e-01
 16 3.44100000e+00   -4.98603034e-01
 17 1.71200000e+00   -3.89817123e-01
 18 8.33700000e-01   -7.43425810e-02
 19 3.91200000e-01   -7.36775200e-04
 20 1.77700000e-01   -9.26189400e-04
 21 7.93900000e-02   0.00000000e+00
S 21
  1 5.38400000e+06   1.23300000e-07
  2 8.06200000e+05   9.57000000e-07
  3 1.83600000e+05   5.03560000e-06
  4 5.22500000e+04   2.09489000e-05
  5 1.73900000e+04   7.32948000e-05
  6 6.52300000e+03   2.22987100e-04
  7 2.68700000e+03   6.14700400e-04
  8 1.17800000e+03   1.59827480e-03
  9 5.36200000e+02   3.96991650e-03
 10 2.51500000e+02   9.09371040e-03
 11 1.21300000e+02   1.88701405e-02
 12 5.98800000e+01   3.35159167e-02
 13 3.00500000e+01   4.61471119e-02
 14 1.51200000e+01   2.56075836e-02
 15 7.01000000e+00   -6.73395749e-02
 16 3.44100000e+00   -2.03809441e-01
 17 1.71200000e+00   -2.88303275e-01
 18 8.33700000e-01   -4.07361705e-02
 19 3.91200000e-01   4.71307689e-01
 20 1.77700000e-01   5.65966007e-01
 21 7.93900000e-02   1.70269360e-01
S 21
  1 5.38400000e+06   1.52600000e-07
  2 8.06200000e+05   1.19460000e-06
  3 1.83600000e+05   6.21140000e-06
  4 5.22500000e+04   2.62588000e-05
  5 1.73900000e+04   8.99315000e-05
  6 6.52300000e+03   2.81455000e-04
  7 2.68700000e+03   7.48131000e-04
  8 1.17800000e+03   2.03487350e-03
  9 5.36200000e+02   4.80185600e-03
 10 2.51500000e+02   1.17025222e-02
 11 1.21300000e+02   2.26723683e-02
```

```
 12 5.98800000e+01   4.43417480e-02
 13 3.00500000e+01   5.42824195e-02
 14 1.51200000e+01   4.13471455e-02
 15 7.01000000e+00   -1.27458698e-01
 16 3.44100000e+00   -2.16143117e-01
 17 1.71200000e+00   -5.95219793e-01
 18 8.33700000e-01   3.71262375e-01
 19 3.91200000e-01   1.72720559e+00
 20 1.77700000e-01   -8.07508990e-01
 21 7.93900000e-02   -8.02768702e-01
S 21
  1 5.38400000e+06   2.01700000e-07
  2 8.06200000e+05   1.59010000e-06
  3 1.83600000e+05   8.18220000e-06
  4 5.22500000e+04   3.50779000e-05
  5 1.73900000e+04   1.17913900e-04
  6 6.52300000e+03   3.78250500e-04
  7 2.68700000e+03   9.74014800e-04
  8 1.17800000e+03   2.75608110e-03
  9 5.36200000e+02   6.22510530e-03
 10 2.51500000e+02   1.60328102e-02
 11 1.21300000e+02   2.93865348e-02
 12 5.98800000e+01   6.24183676e-02
 13 3.00500000e+01   6.94862147e-02
 14 1.51200000e+01   6.23982686e-02
 15 7.01000000e+00   -2.22276309e-01
 16 3.44100000e+00   -3.27768605e-01
 17 1.71200000e+00   -1.26599615e+00
 18 8.33700000e-01   3.35803976e+00
 19 3.91200000e-01   -1.01939561e+00
 20 1.77700000e-01   -2.46072372e+00
 21 7.93900000e-02   1.99964963e+00
P 14
  1 4.55200000e+03   5.19530000e-05
  2 1.07800000e+03   4.60402400e-04
  3 3.50100000e+02   2.62081640e-03
  4 1.33800000e+02   1.11872441e-02
  5 5.65200000e+01   3.78228974e-02
  6 2.55800000e+01   1.02116397e-01
  7 1.21400000e+01   2.10313745e-01
  8 5.90200000e+00   3.17382380e-01
  9 2.91000000e+00   3.27164036e-01
 10 1.43500000e+00   1.77686982e-01
 11 6.57000000e-01   2.93588218e-02
 12 3.00500000e-01   -7.52283000e-05
 13 1.34000000e-01   6.79589100e-04
 14 5.78300000e-02   -1.25946100e-04
P 14
  1 4.55200000e+03   1.25053000e-05
  2 1.07800000e+03   1.10641500e-04
  3 3.50100000e+02   6.32449500e-04
  4 1.33800000e+02   2.70513400e-03
  5 5.65200000e+01   9.25645860e-03
  6 2.55800000e+01   2.54006335e-02
  7 1.21400000e+01   5.37628786e-02
  8 5.90200000e+00   8.25945281e-02
  9 2.91000000e+00   9.26223095e-02
 10 1.43500000e+00   3.09116256e-02
 11 6.57000000e-01   -1.93095245e-01
 12 3.00500000e-01   -4.36362552e-01
 13 1.34000000e-01   -4.00880059e-01
 14 5.78300000e-02   -1.22049293e-01
P 14
  1 4.55200000e+03   1.55755000e-05
  2 1.07800000e+03   1.42293500e-04
  3 3.50100000e+02   7.88608800e-04
  4 1.33800000e+02   3.48358910e-03
  5 5.65200000e+01   1.15313768e-02
  6 2.55800000e+01   3.27400156e-02
  7 1.21400000e+01   6.76387623e-02
  8 5.90200000e+00   1.10002175e-01
```

```
  9 2.91000000e+00   1.11837996e-01
 10 1.43500000e+00   1.08244664e-01
 11 6.57000000e-01   -6.88491510e-01
 12 3.00500000e-01   -5.89511985e-01
 13 1.34000000e-01   7.74697719e-01
 14 5.78300000e-02   4.12884245e-01
P 14
  1 4.55200000e+03   2.99529000e-05
  2 1.07800000e+03   1.99674200e-04
  3 3.50100000e+02   1.51486450e-03
  4 1.33800000e+02   4.95677510e-03
  5 5.65200000e+01   2.26188204e-02
  6 2.55800000e+01   4.52278279e-02
  7 1.21400000e+01   1.49569116e-01
  8 5.90200000e+00   1.16058996e-01
  9 2.91000000e+00   3.29017516e-01
 10 1.43500000e+00   -3.92408581e-01
 11 6.57000000e-01   -1.53159372e+00
 12 3.00500000e-01   1.96339207e+00
 13 1.34000000e-01   -1.68662441e-01
 14 5.78300000e-02   -7.13412188e-01
D 5
  1 4.30080000e+00   2.12636418e-02
  2 1.83460000e+00   1.88050678e-02
  3 7.82600000e-01   2.64797630e-01
  4 3.33900000e-01   5.53510347e-01
  5 1.42400000e-01   3.00434000e-01
D 5
  1 4.30080000e+00   -1.01265740e-03
  2 1.83460000e+00   -1.03476132e-01
  3 7.82600000e-01   8.91446808e-01
  4 3.33900000e-01   6.94034624e-02
  5 1.42400000e-01   -9.13145622e-01
F 4
  1 1.81600000e+00   -1.26043063e-02
  2 8.80600000e-01   2.45954440e-01
  3 4.27000000e-01   5.49126285e-01
  4 2.07000000e-01   3.36333481e-01
end
NewGTO S
S 21
  1 6.29700000e+06   1.59210000e-06
  2 9.43100000e+05   1.23599000e-05
  3 2.14900000e+05   6.49159000e-05
  4 6.12500000e+04   2.69332600e-04
  5 2.04500000e+04   9.34603400e-04
  6 7.71900000e+03   2.80814930e-03
  7 3.19800000e+03   7.67362780e-03
  8 1.40200000e+03   1.98887182e-02
  9 6.37200000e+02   4.82561629e-02
 10 2.98900000e+02   1.05750417e-01
 11 1.44300000e+02   2.00209679e-01
 12 7.12100000e+01   3.00704756e-01
 13 3.57300000e+01   2.98654879e-01
 14 1.79700000e+01   1.46326707e-01
 15 8.34100000e+00   2.01597202e-02
 16 4.11200000e+00   -1.02704800e-04
 17 2.04500000e+00   1.10109070e-03
 18 9.77000000e-01   -3.10142600e-04
 19 4.76600000e-01   -1.74322800e-04
 20 2.18500000e-01   -4.19145700e-04
 21 9.75900000e-02   -9.12518000e-05
S 21
  1 6.29700000e+06   4.38200000e-07
  2 9.43100000e+05   3.40010000e-06
  3 2.14900000e+05   1.78707000e-05
  4 6.12500000e+04   7.41041000e-05
  5 2.04500000e+04   2.57663900e-04
  6 7.71900000e+03   7.74651600e-04
  7 3.19800000e+03   2.13129540e-03
  8 1.40200000e+03   5.56892110e-03
```

```
  9 6.37200000e+02   1.38537158e-02
 10 2.98900000e+02   3.16453111e-02
 11 1.44300000e+02   6.56714009e-02
 12 7.12100000e+01   1.16366754e-01
 13 3.57300000e+01   1.59128876e-01
 14 1.79700000e+01   9.41285220e-02
 15 8.34100000e+00   -1.86945575e-01
 16 4.11200000e+00   -5.01734920e-01
 17 2.04500000e+00   -3.95479956e-01
 18 9.77000000e-01   -7.32558993e-02
 19 4.76600000e-01   -6.16003380e-03
 20 2.18500000e-01   -8.64194040e-03
 21 9.75900000e-02   -2.10110990e-03
S 21
  1 6.29700000e+06   1.35000000e-07
  2 9.43100000e+05   1.04730000e-06
  3 2.14900000e+05   5.50540000e-06
  4 6.12500000e+04   2.28258000e-05
  5 2.04500000e+04   7.93897000e-05
  6 7.71900000e+03   2.38649600e-04
  7 3.19800000e+03   6.57099800e-04
  8 1.40200000e+03   1.71758370e-03
  9 6.37200000e+02   4.28269700e-03
 10 2.98900000e+02   9.81182580e-03
 11 1.44300000e+02   2.05253893e-02
 12 7.12100000e+01   3.68719010e-02
 13 3.57300000e+01   5.19727886e-02
 14 1.79700000e+01   3.18049495e-02
 15 8.34100000e+00   -6.92984997e-02
 16 4.11200000e+00   -2.29930204e-01
 17 2.04500000e+00   -3.16948205e-01
 18 9.77000000e-01   -1.59749150e-02
 19 4.76600000e-01   4.95799807e-01
 20 2.18500000e-01   5.50219898e-01
 21 9.75900000e-02   1.58102388e-01
S 21
  1 6.29700000e+06   1.51500000e-07
  2 9.43100000e+05   1.18640000e-06
  3 2.14900000e+05   6.15540000e-06
  4 6.12500000e+04   2.59766000e-05
  5 2.04500000e+04   8.82303000e-05
  6 7.71900000e+03   2.73765800e-04
  7 3.19800000e+03   7.23791000e-04
  8 1.40200000e+03   1.98836830e-03
  9 6.37200000e+02   4.68848970e-03
 10 2.98900000e+02   1.14963962e-02
 11 1.44300000e+02   2.23354902e-02
 12 7.12100000e+01   4.45622770e-02
 13 3.57300000e+01   5.56857997e-02
 14 1.79700000e+01   4.65569275e-02
 15 8.34100000e+00   -1.26255521e-01
 16 4.11200000e+00   -2.17545259e-01
 17 2.04500000e+00   -6.28301634e-01
 18 9.77000000e-01   4.24188658e-01
 19 4.76600000e-01   1.74505122e+00
 20 2.18500000e-01   -8.97041429e-01
 21 9.75900000e-02   -7.68350182e-01
S 21
  1 6.29700000e+06   2.17500000e-07
  2 9.43100000e+05   1.70140000e-06
  3 2.14900000e+05   8.83650000e-06
  4 6.12500000e+04   3.72408000e-05
  5 2.04500000e+04   1.26734500e-04
  6 7.71900000e+03   3.92329400e-04
  7 3.19800000e+03   1.04101410e-03
  8 1.40200000e+03   2.85121380e-03
  9 6.37200000e+02   6.76792040e-03
 10 2.98900000e+02   1.65725289e-02
 11 1.44300000e+02   3.26736745e-02
 12 7.12100000e+01   6.51357654e-02
 13 3.57300000e+01   8.50063621e-02
```

```
 14 1.79700000e+01   6.24786338e-02
 15 8.34100000e+00   -2.34497907e-01
 16 4.11200000e+00   -4.09053361e-01
 17 2.04500000e+00   -1.25281008e+00
 18 9.77000000e-01   3.93868724e+00
 19 4.76600000e-01   -1.96343163e+00
 20 2.18500000e-01   -1.83559477e+00
 21 9.75900000e-02   1.78276513e+00
P 14
  1 5.26600000e+03   5.22613000e-05
  2 1.24700000e+03   4.63522300e-04
  3 4.05000000e+02   2.64098570e-03
  4 1.54800000e+02   1.13168621e-02
  5 6.53800000e+01   3.84702429e-02
  6 2.95900000e+01   1.04338980e-01
  7 1.40400000e+01   2.15682941e-01
  8 6.82400000e+00   3.25258367e-01
  9 3.36900000e+00   3.26179695e-01
 10 1.66600000e+00   1.63147292e-01
 11 7.68100000e-01   2.38968197e-02
 12 3.50400000e-01   4.74148300e-04
 13 1.55600000e-01   7.36633300e-04
 14 6.68100000e-02   1.98570000e-05
P 14
  1 5.26600000e+03   1.34501000e-05
  2 1.24700000e+03   1.18880900e-04
  3 4.05000000e+02   6.81566000e-04
  4 1.54800000e+02   2.92224110e-03
  5 6.53800000e+01   1.00822376e-02
  6 2.95900000e+01   2.77819341e-02
  7 1.40400000e+01   5.93004576e-02
  8 6.82400000e+00   9.11250782e-02
  9 3.36900000e+00   1.00494729e-01
 10 1.66600000e+00   1.22559698e-02
 11 7.68100000e-01   -2.37663445e-01
 12 3.50400000e-01   -4.37452406e-01
 13 1.55600000e-01   -3.65271350e-01
 14 6.68100000e-02   -1.13754627e-01
P 14
  1 5.26600000e+03   1.54652000e-05
  2 1.24700000e+03   1.40879100e-04
  3 4.05000000e+02   7.84589200e-04
  4 1.54800000e+02   3.46750170e-03
  5 6.53800000e+01   1.16099085e-02
  6 2.95900000e+01   3.31206035e-02
  7 1.40400000e+01   6.93083831e-02
  8 6.82400000e+00   1.10584668e-01
  9 3.36900000e+00   1.08493300e-01
 10 1.66600000e+00   7.73246928e-02
 11 7.68100000e-01   -7.01546916e-01
 12 3.50400000e-01   -4.72302903e-01
 13 1.55600000e-01   6.91338286e-01
 14 6.68100000e-02   4.65832381e-01
P 14
  1 5.26600000e+03   2.61265000e-05
  2 1.24700000e+03   1.83114200e-04
  3 4.05000000e+02   1.32375180e-03
  4 1.54800000e+02   4.55960340e-03
  5 6.53800000e+01   1.99287095e-02
  6 2.95900000e+01   4.28388835e-02
  7 1.40400000e+01   1.31020473e-01
  8 6.82400000e+00   1.14102100e-01
  9 3.36900000e+00   2.82487282e-01
 10 1.66600000e+00   -3.61975733e-01
 11 7.68100000e-01   -1.29224861e+00
 12 3.50400000e-01   1.43716297e+00
 13 1.55600000e-01   4.00730908e-01
 14 6.68100000e-02   -1.00149722e+00
D 5
  1 5.07550000e+00   2.51450138e-02
  2 2.18330000e+00   2.47353053e-02
```

```
  3 9.39200000e-01   2.92276387e-01
  4 4.04000000e-01   5.46121626e-01
  5 1.73800000e-01   2.74586046e-01
D 5
  1 5.07550000e+00   -3.42198330e-03
  2 2.18330000e+00   -6.85076016e-02
  3 9.39200000e-01   9.01076877e-01
  4 4.04000000e-01   -1.18517043e-02
  5 1.73800000e-01   -8.84972741e-01
F 4
  1 1.32220000e+00   7.48384273e-02
  2 7.31900000e-01   4.16550600e-01
  3 4.05100000e-01   3.45168543e-01
  4 2.24300000e-01   3.00279115e-01
end
NewGTO Cl
S 20
  1 6.41000000e+06   1.81340000e-06
  2 9.59600000e+05   1.41111000e-05
  3 2.18300000e+05   7.42369000e-05
  4 6.18100000e+04   3.14115200e-04
  5 2.01400000e+04   1.14636230e-03
  6 7.26400000e+03   3.73869090e-03
  7 2.83200000e+03   1.10940777e-02
  8 1.17500000e+03   3.01135997e-02
  9 5.12600000e+02   7.39105226e-02
 10 2.33000000e+02   1.58249381e-01
 11 1.09500000e+02   2.74735095e-01
 12 5.28600000e+01   3.34036462e-01
 13 2.58400000e+01   2.17559780e-01
 14 1.21700000e+01   4.57431155e-02
 15 6.03000000e+00   -2.93391000e-05
 16 3.01200000e+00   1.83502750e-03
 17 1.51100000e+00   -4.44532700e-04
 18 6.60400000e-01   -7.76684000e-05
 19 2.92600000e-01   -4.58712400e-04
 20 1.25400000e-01   -9.40595000e-05
S 20
  1 6.41000000e+06   5.31400000e-07
  2 9.59600000e+05   4.13560000e-06
  3 2.18300000e+05   2.17541000e-05
  4 6.18100000e+04   9.21099000e-05
  5 2.01400000e+04   3.36357500e-04
  6 7.26400000e+03   1.10046940e-03
  7 2.83200000e+03   3.28485880e-03
  8 1.17500000e+03   9.05738090e-03
  9 5.12600000e+02   2.29428466e-02
 10 2.33000000e+02   5.25801453e-02
 11 1.09500000e+02   1.04288359e-01
 12 5.28600000e+01   1.65462254e-01
 13 2.58400000e+01   1.54021839e-01
 14 1.21700000e+01   -7.38403143e-02
 15 6.03000000e+00   -4.64015406e-01
 16 3.01200000e+00   -5.67479308e-01
 17 1.51100000e+00   -1.90508562e-01
 18 6.60400000e-01   1.23140436e-01
 19 2.92600000e-01   1.81415144e-01
 20 1.25400000e-01   5.72888245e-02
S 20
  1 6.41000000e+06   3.00000000e-09
  2 9.59600000e+05   2.42000000e-08
  3 2.18300000e+05   1.22700000e-07
  4 6.18100000e+04   5.44400000e-07
  5 2.01400000e+04   1.86150000e-06
  6 7.26400000e+03   6.49150000e-06
  7 2.83200000e+03   1.71666000e-05
  8 1.17500000e+03   4.96124000e-05
  9 5.12600000e+02   9.06845000e-05
 10 2.33000000e+02   1.67295600e-04
 11 1.09500000e+02   -1.52020100e-04
 12 5.28600000e+01   -1.18701400e-03
```

```
 13 2.58400000e+01   -3.57029940e-03
 14 1.21700000e+01   4.45877520e-03
 15 6.03000000e+00   4.23049009e-02
 16 3.01200000e+00   1.72928718e-01
 17 1.51100000e+00   7.99121551e-02
 18 6.60400000e-01   -4.30562797e-01
 19 2.92600000e-01   -5.80076876e-01
 20 1.25400000e-01   -1.83332217e-01
S 20
  1 6.41000000e+06   1.69800000e-07
  2 9.59600000e+05   1.30200000e-06
  3 2.18300000e+05   6.99600000e-06
  4 6.18100000e+04   2.88036000e-05
  5 2.01400000e+04   1.08950000e-04
  6 7.26400000e+03   3.41548300e-04
  7 2.83200000e+03   1.07404200e-03
  8 1.17500000e+03   2.78925910e-03
  9 5.12600000e+02   7.62512340e-03
 10 2.33000000e+02   1.61014859e-02
 11 1.09500000e+02   3.62533789e-02
 12 5.28600000e+01   5.04717507e-02
 13 2.58400000e+01   6.67371854e-02
 14 1.21700000e+01   -6.17185781e-02
 15 6.03000000e+00   -1.24932458e-01
 16 3.01200000e+00   -6.04647456e-01
 17 1.51100000e+00   -1.78307776e-01
 18 6.60400000e-01   2.18904089e+00
 19 2.92600000e-01   -8.54423151e-01
 20 1.25400000e-01   -8.25303523e-01
S 20
  1 6.41000000e+06   -2.45400000e-07
  2 9.59600000e+05   -1.70380000e-06
  3 2.18300000e+05   -1.05333000e-05
  4 6.18100000e+04   -3.58464000e-05
  5 2.01400000e+04   -1.71046400e-04
  6 7.26400000e+03   -3.99562600e-04
  7 2.83200000e+03   -1.76898390e-03
  8 1.17500000e+03   -2.99905090e-03
  9 5.12600000e+02   -1.33610420e-02
 10 2.33000000e+02   -1.49230657e-02
 11 1.09500000e+02   -7.14207934e-02
 12 5.28600000e+01   -2.38172309e-02
 13 2.58400000e+01   -2.06192769e-01
 14 1.21700000e+01   3.57897053e-01
 15 6.03000000e+00   -3.57728043e-01
 16 3.01200000e+00   2.69247013e+00
 17 1.51100000e+00   -3.95409694e+00
 18 6.60400000e-01   6.26233179e-01
 19 2.92600000e-01   2.58435997e+00
 20 1.25400000e-01   -1.90447989e+00
P 12
  1 2.54800000e+03   2.35701700e-04
  2 6.03700000e+02   2.05157990e-03
  3 1.95600000e+02   1.11543331e-02
  4 7.41500000e+01   4.39815972e-02
  5 3.09400000e+01   1.29994232e-01
  6 1.36900000e+01   2.72959183e-01
  7 6.22900000e+00   3.83690452e-01
  8 2.87800000e+00   2.91869453e-01
  9 1.28200000e+00   7.04461278e-02
 10 5.64100000e-01   1.28677700e-03
 11 2.34800000e-01   1.82967110e-03
 12 9.31200000e-02   1.63527000e-05
P 12
  1 2.54800000e+03   6.39499000e-05
  2 6.03700000e+02   5.56986200e-04
  3 1.95600000e+02   3.04748690e-03
  4 7.41500000e+01   1.21470785e-02
  5 3.09400000e+01   3.68757425e-02
  6 1.36900000e+01   7.96346992e-02
  7 6.22900000e+00   1.17986470e-01
```

```
  8 2.87800000e+00   8.71432235e-02
  9 1.28200000e+00   -1.42101282e-01
 10 5.64100000e-01   -4.27685723e-01
 11 2.34800000e-01   -4.43417262e-01
 12 9.31200000e-02   -1.62396124e-01
P 12
  1 2.54800000e+03   7.92628000e-05
  2 6.03700000e+02   7.42637900e-04
  3 1.95600000e+02   3.78255490e-03
  4 7.41500000e+01   1.63312278e-02
  5 3.09400000e+01   4.60944074e-02
  6 1.36900000e+01   1.11998718e-01
  7 6.22900000e+00   1.29275347e-01
  8 2.87800000e+00   1.72551149e-01
  9 1.28200000e+00   -4.62888419e-01
 10 5.64100000e-01   -7.98744967e-01
 11 2.34800000e-01   6.41053120e-01
 12 9.31200000e-02   5.32811061e-01
P 12
  1 2.54800000e+03   1.07726600e-04
  2 6.03700000e+02   1.02722500e-03
  3 1.95600000e+02   5.16452550e-03
  4 7.41500000e+01   2.28840699e-02
  5 3.09400000e+01   6.51495253e-02
  6 1.36900000e+01   1.62079215e-01
  7 6.22900000e+00   1.60873768e-01
  8 2.87800000e+00   2.85444809e-01
  9 1.28200000e+00   -1.72569936e+00
 10 5.64100000e-01   1.13370702e+00
 11 2.34800000e-01   7.41597631e-01
 12 9.31200000e-02   -1.04751067e+00
D 4
  1 2.50000000e-01   3.72890783e-01
  2 6.18000000e-01   5.73676911e-01
  3 1.52900000e+00   1.71976094e-01
  4 3.78100000e+00   3.71180574e-02
D 4
  1 2.50000000e-01   -1.03878704e+00
  2 6.18000000e-01   4.42872368e-01
  3 1.52900000e+00   6.54921272e-01
  4 3.78100000e+00   -8.95377154e-02
F 3
  1 3.20000000e-01   3.30473066e-01
  2 6.56000000e-01   5.38809829e-01
  3 1.34500000e+00   2.56779382e-01
end
NewGTO Ar
S 21
  1 9.14900000e+06   1.34270000e-06
  2 1.37000000e+06   1.04386000e-05
  3 3.11900000e+05   5.48567000e-05
  4 8.86500000e+04   2.29582800e-04
  5 2.93300000e+04   8.10333800e-04
  6 1.09300000e+04   2.48534130e-03
  7 4.48000000e+03   6.83691730e-03
  8 1.96200000e+03   1.76198815e-02
  9 8.94100000e+02   4.28751718e-02
 10 4.19600000e+02   9.54852872e-02
 11 2.02300000e+02   1.85064004e-01
 12 9.98400000e+01   2.89041537e-01
 13 5.00700000e+01   3.10165596e-01
 14 2.51400000e+01   1.72183219e-01
 15 1.18100000e+01   2.85227179e-02
 16 5.88200000e+00   -5.75738100e-04
 17 2.93900000e+00   1.18121500e-03
 18 1.40500000e+00   -4.80534900e-04
 19 6.96300000e-01   1.88849400e-04
 20 3.18800000e-01   -9.72509000e-05
 21 1.41000000e-01   1.74327000e-05
S 21
  1 9.14900000e+06   3.81300000e-07
```

```
  2 1.37000000e+06   2.96330000e-06
  3 3.11900000e+05   1.55803000e-05
  4 8.86500000e+04   6.51880000e-05
  5 2.93300000e+04   2.30432200e-04
  6 1.09300000e+04   7.07458600e-04
  7 4.48000000e+03   1.95729630e-03
  8 1.96200000e+03   5.08562250e-03
  9 8.94100000e+02   1.26528350e-02
 10 4.19600000e+02   2.93064513e-02
 11 2.02300000e+02   6.17712186e-02
 12 9.98400000e+01   1.12540721e-01
 13 5.00700000e+01   1.62293160e-01
 14 2.51400000e+01   1.18411976e-01
 15 1.18100000e+01   -1.46147512e-01
 16 5.88200000e+00   -4.97751505e-01
 17 2.93900000e+00   -4.34239643e-01
 18 1.40500000e+00   -8.90745104e-02
 19 6.96300000e-01   -5.38050000e-04
 20 3.18800000e-01   -2.04036030e-03
 21 1.41000000e-01   2.80176000e-05
S 21
  1 9.14900000e+06   1.19600000e-07
  2 1.37000000e+06   9.29500000e-07
  3 3.11900000e+05   4.88840000e-06
  4 8.86500000e+04   2.04455000e-05
  5 2.93300000e+04   7.23150000e-05
  6 1.09300000e+04   2.21909700e-04
  7 4.48000000e+03   6.14754500e-04
  8 1.96200000e+03   1.59709840e-03
  9 8.94100000e+02   3.98653340e-03
 10 4.19600000e+02   9.25777920e-03
 11 2.02300000e+02   1.97068769e-02
 12 9.98400000e+01   3.64106866e-02
 13 5.00700000e+01   5.43600462e-02
 14 2.51400000e+01   4.12101400e-02
 15 1.18100000e+01   -5.57198596e-02
 16 5.88200000e+00   -2.38979099e-01
 17 2.93900000e+00   -3.48066057e-01
 18 1.40500000e+00   -1.14046784e-02
 19 6.96300000e-01   5.08891467e-01
 20 3.18800000e-01   5.48407888e-01
 21 1.41000000e-01   1.56444137e-01
S 21
  1 9.14900000e+06   -1.36700000e-07
  2 1.37000000e+06   -1.07400000e-06
  3 3.11900000e+05   -5.55650000e-06
  4 8.86500000e+04   -2.37565000e-05
  5 2.93300000e+04   -8.16261000e-05
  6 1.09300000e+04   -2.60209600e-04
  7 4.48000000e+03   -6.86131100e-04
  8 1.96200000e+03   -1.89503990e-03
  9 8.94100000e+02   -4.40774940e-03
 10 4.19600000e+02   -1.11231815e-02
 11 2.02300000e+02   -2.15812668e-02
 12 9.98400000e+01   -4.50890638e-02
 13 5.00700000e+01   -5.82069373e-02
 14 2.51400000e+01   -6.07658119e-02
 15 1.18100000e+01   1.16719219e-01
 16 5.88200000e+00   2.03465012e-01
 17 2.93900000e+00   7.21530023e-01
 18 1.40500000e+00   -4.38597272e-01
 19 6.96300000e-01   -1.84490573e+00
 20 3.18800000e-01   1.02026461e+00
 21 1.41000000e-01   7.09466695e-01
S 21
  1 9.14900000e+06   2.02700000e-07
  2 1.37000000e+06   1.59170000e-06
  3 3.11900000e+05   8.24540000e-06
  4 8.86500000e+04   3.51960000e-05
  5 2.93300000e+04   1.21200400e-04
  6 1.09300000e+04   3.85346100e-04
```

```
  7 4.48000000e+03   1.02026250e-03
  8 1.96200000e+03   2.80766860e-03
  9 8.94100000e+02   6.57909810e-03
 10 4.19600000e+02   1.65651285e-02
 11 2.02300000e+02   3.26566656e-02
 12 9.98400000e+01   6.82935031e-02
 13 5.00700000e+01   9.31850677e-02
 14 2.51400000e+01   8.92905674e-02
 15 1.18100000e+01   -2.51036339e-01
 16 5.88200000e+00   -3.89771465e-01
 17 2.93900000e+00   -1.51218152e+00
 18 1.40500000e+00   4.58982859e+00
 19 6.96300000e-01   -2.72890640e+00
 20 3.18800000e-01   -1.32480766e+00
 21 1.41000000e-01   1.59409222e+00
P 14
  1 7.05000000e+03   5.01843000e-05
  2 1.66900000e+03   4.45365000e-04
  3 5.42100000e+02   2.54799730e-03
  4 2.07100000e+02   1.10155237e-02
  5 8.75200000e+01   3.78489764e-02
  6 3.96100000e+01   1.04355193e-01
  7 1.87800000e+01   2.19335151e-01
  8 9.13000000e+00   3.34615307e-01
  9 4.51600000e+00   3.26771020e-01
 10 2.24500000e+00   1.48153015e-01
 11 1.06500000e+00   1.92586150e-02
 12 4.88500000e-01   5.51936800e-04
 13 2.16600000e-01   1.76924600e-04
 14 9.25500000e-02   -1.68555000e-05
P 14
  1 7.05000000e+03   1.41163000e-05
  2 1.66900000e+03   1.24932700e-04
  3 5.42100000e+02   7.18915000e-04
  4 2.07100000e+02   3.11348210e-03
  5 8.75200000e+01   1.08614712e-02
  6 3.96100000e+01   3.05213102e-02
  7 1.87800000e+01   6.64087472e-02
  8 9.13000000e+00   1.04335520e-01
  9 4.51600000e+00   1.10481227e-01
 10 2.24500000e+00   -1.32896226e-02
 11 1.06500000e+00   -2.78221541e-01
 12 4.88500000e-01   -4.34865574e-01
 13 2.16600000e-01   -3.37575208e-01
 14 9.25500000e-02   -9.52338864e-02
P 14
  1 7.05000000e+03   1.81269000e-05
  2 1.66900000e+03   1.60467400e-04
  3 5.42100000e+02   9.24138300e-04
  4 2.07100000e+02   4.00947730e-03
  5 8.75200000e+01   1.40247320e-02
  6 3.96100000e+01   3.96644574e-02
  7 1.87800000e+01   8.83804352e-02
  8 9.13000000e+00   1.32531330e-01
  9 4.51600000e+00   1.41996113e-01
 10 2.24500000e+00   -1.51877420e-03
 11 1.06500000e+00   -8.79227120e-01
 12 4.88500000e-01   -2.13830610e-01
 13 2.16600000e-01   7.84225468e-01
 14 9.25500000e-02   2.86621342e-01
P 14
  1 7.05000000e+03   3.43626000e-05
  2 1.66900000e+03   2.03057600e-04
  3 5.42100000e+02   1.74641850e-03
  4 2.07100000e+02   5.17887170e-03
  5 8.75200000e+01   2.70262790e-02
  6 3.96100000e+01   5.07693530e-02
  7 1.87800000e+01   1.93375640e-01
  8 9.13000000e+00   1.01572651e-01
  9 4.51600000e+00   4.35592857e-01
 10 2.24500000e+00   -9.85508099e-01
```

```
 11 1.06500000e+00   -8.43210270e-01
 12 4.88500000e-01   2.02126894e+00
 13 2.16600000e-01   -6.68903866e-01
 14 9.25500000e-02   -4.77495424e-01
D 5
  1 7.63270000e+00   2.79057812e-02
  2 3.28760000e+00   2.93683713e-02
  3 1.41600000e+00   3.03094373e-01
  4 6.09900000e-01   5.42279927e-01
  5 2.62700000e-01   2.64001541e-01
D 5
  1 7.63270000e+00   -3.28925500e-03
  2 3.28760000e+00   -7.01912500e-02
  3 1.41600000e+00   9.23579371e-01
  4 6.09900000e-01   -6.16176285e-02
  5 2.62700000e-01   -8.64872079e-01
F 4
  1 3.05820000e+00   -1.06008451e-02
  2 1.52920000e+00   3.04583459e-01
  3 7.64700000e-01   5.42344318e-01
  4 3.82400000e-01   2.77175008e-01
end
```

S8 References

S1. Neese, F.; Valeev, E. F. Revisiting the Atomic Natural Orbital Approach for Basis Sets: Robust Systematic Basis Sets for Explicitly Correlated and Conventional Correlated *ab initio* Methods? *J. Chem. Theory Comput.* **2011,** *7*, 33–43.

S2. Huber, K.-P.; Herzberg, G., *Molecular Spectra and Molecular Structure IV. Constants of Diatomic Molecules*. Van Nostrand Reinhold Co.: New York, NY, 1979.

S3. Graham, W. R. M.; Weltner, W., Jr. B atoms, B_2 and H_2BO molecules: ESR and optical spectra at 4 °K. *J. Chem. Phys.* **1976,** *65*, 1516–1521.

S4. Bender, C. F.; Davidson, E. R. Electronic Structure of the B_2 Molecule. *J. Chem. Phys.* **1967,** *46*, 3313–3319.

S5. Schwenke, D. W. The extrapolation of one-electron basis sets in electronic structure calculations: How it should work and how it can be made to work. *J. Chem. Phys.* **2005,** *122*, 014107.
